# 1,4-Benzenedimethanethiol (1,4-BDMT) as a scavenger for greener peptide resin cleavages†

**DOI:** 10.1039/c9ra08553j

**Published:** 2019-11-28

**Authors:** Jan Pawlas, Thomas Svensson, Jon H. Rasmussen

**Affiliations:** PolyPeptide Group Limhamnsvägen 108, PO BOX 30089 20061 Limhamn Sweden jan.pawlas@polypeptide.com

## Abstract

Aiming at elevating the environmental profile of the solid-phase peptide synthesis (SPPS) methodology by improving the quality of crude peptides that SPPS provides we assessed a series of benzylthiols (BTs) as scavengers for global deprotection/TFA cleavage of exenatide peptide resin accessed by Fmoc SPPS. In these studies we identified 1,4-BDMT as a scavenger that affords the peptide in higher quality than the standard aliphatic thiol reagents, not least in terms of the content of critical peptide impurities in the crude material. Further, 1,4-BDMT exhibited favorable UV detectability as well as stability and solubility in TFA. Finally, based on the MS assessment of the crude exenatide products herein we propose that thiol scavengers in the cleavage of Trp containing peptide resins do not minimize the content of Trp oxidants by means of inhibiting Trp oxidation but rather by forming a peptide–thiol adduct *via* a mechanism involving an attack of a thiol on an oxindolylalanine (Oia) impurity present in the crude material.

## Abstract 2

1,4-BDMT is a benign, non-odorous scavenger for peptide resin cleavages which provides crude peptides in higher quality than the aliphatic thiols used for this purpose.

## Graphical abstract

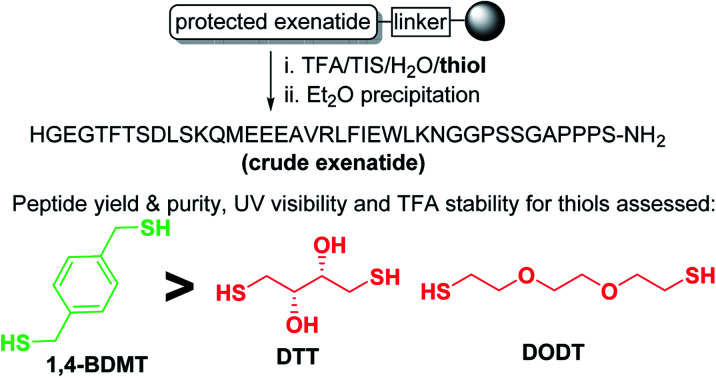

## Introduction

Over the past 13 decades the art of peptide synthesis has evolved to encompass numerous reliable methods and protocols^1^ for the preparation of a broad spectrum of peptides whose applications span a wide range of fields including pharmaceuticals,^2^ cosmetics^3^ and materials science.^4^ Nevertheless, despite the intense scrutiny that greenness in the chemical sciences in general^5^ and in peptide synthesis in particular^6^ has received in recent years, sustainable synthesis and purification of peptides has remained challenging.^7^ For example, it has been noted that within the realms of pharmaceutical peptide manufacturing^8^ production of one kg of an active pharmaceutical ingredient (API) typically generates 3000–15 000 kg of waste containing highly hazardous reagents and solvents.^9^

As Merrifield's solid-phase peptide synthesis (SPPS)^10^ is the most widely used method to synthesize peptides^11^ substantial inroads have been made towards greening of this technique,^12^ including efforts to access more complex peptides in a greener manner.^13^ Nevertheless, while significant attention has been paid to the greening of the assembly of polypeptide chains on solid supports considerably less effort has been dedicated to improving the environmental profile of the subsequent process stages, *i.e.* removal of protecting groups (PGs)/cleavage of the target molecule off the resin^14^ and isolation of the crude material by antisolvent precipitation.^13*a*,15^ From sustainability standpoint a crucial role during PG removal/cleavage of a peptide off a polymer support is played by scavengers. These reagents serve to minimize formation of a wide range of byproducts capable of derailing the ensuing downstream processing (DSP) of the crude product. As Merrifield's original Boc SPPS^16^ hinges on the use of the undesirable HF for removal of peptides from solid supports Fmoc SPPS,^17^ which requires the less hazardous TFA for the final cleavage, has emerged as the preferred peptide synthesis method.^18^ In fact, numerous scavengers and scavenger cocktails effective in mitigating the formation of a wide range of cleavage related impurities were developed for final TFA cleavages of peptide resins accessed by Fmoc SPPS.^19^ With respect to scavenging efficiency of the scavengers in the art aliphatic thiols such as EDT,^20^ DTT^21^ and DODT^22^ have been found to constitute particularly effective reagents for minimizing side reactions during peptide resin cleavages. Nevertheless, aliphatic thiols are often malodorous, can form byproducts by reacting with peptides^23^ and, due to the lack of chromophores, can form impurities that can be difficult to detect by UV during DSP of crude peptide products.^24^ On the other hand, aromatic thiols which are easier to detect are also less reactive than aliphatic thiols and thereby less effective as scavengers.^25^

## Results & discussion

As a part of a program aimed at an advancement of greener practices in manufacturing of therapeutic peptides^26^ we set out to develop effective scavengers for peptide resin cleavages which would address the aforementioned shortcomings of aliphatic thiols. Specifically, we reasoned that BTs could constitute suitable scavengers for cleavages of peptide resins by the virtue of combining the high reactivity of aliphatic thiols with the UV visibility of aromatic compounds. According to SciFinder more than 490 000 BTs are known >8000 of which are commercially available. Aiming at scavengers useful for large scale manufacturing applications we focused on BTs that would be relatively simple and thus could be made cost efficiently while not containing any sensitive functional groups not compatible with conc. TFA used in peptide resin cleavages.

**Fig. 1:**
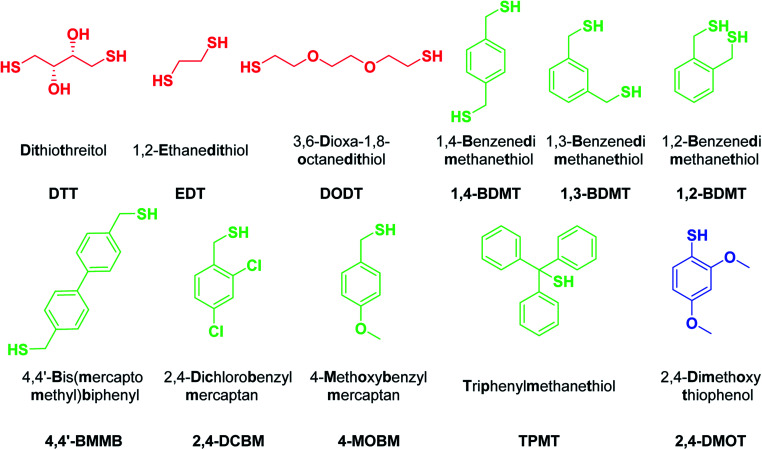
Structures, chemical names and abbreviations for thiols evaluated as scavengers in peptide resin TFA cleavages. Aliphatic, benzylic and aromatic thiols in red, green and blue, respectively.

To evaluate the impact of the scavenger structure on the outcome of peptide resin cleavages we selected a series of BTs varying in electronic and steric properties (Fig. 1). The three BDMT isomers and the biphenyl based 4,4′-BMMB were chosen as BT counterparts of the aliphatic dithiols DTT and DODT. Moreover, the electron withdrawing group (EWG) containing 2,4-DCBM, the electron donating group (EDG) based 4-MOBM and the highly sterically congested α-diphenyl substituted TPMT were examined as well. Aliphatic DTT, EDT and DODT as well as the aromatic 2,4-DMOT were used as benchmarks and the 39-mer antidiabetic exenatide (1)^27^ was selected as the model peptide.

HGEGTFTSDLSKQMEEEAVRLFIEWLKNGGPSSGAPPPS-NH_2_1

**Scheme 1:**
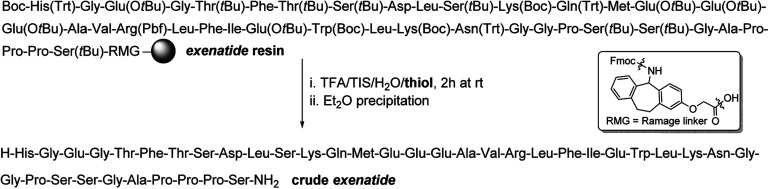
TFA cleavages of exenatide resin using thiol scavengers shown in Fig. 1.

The 39-mer 1 was chosen as the substrate as it contains a plethora of sensitive amino acids (AAs)^28^ which can undergo various side reactions during the cleavage of the peptide off the resin, for example by forming adducts with the species liberated from side chain PGs. An exenatide resin was synthesized by standard Fmoc SPPS^18^ and the cleavages of this resin were carried out for 2 h at rt using TFA/TIS/H_2_O (95 : 3 : 2, v/v/v) in the presence of Fig. 1 thiol scavengers (Scheme 1). Furthermore, a control experiment in the absence of a thiol was carried out as well.

**Fig. 2:**
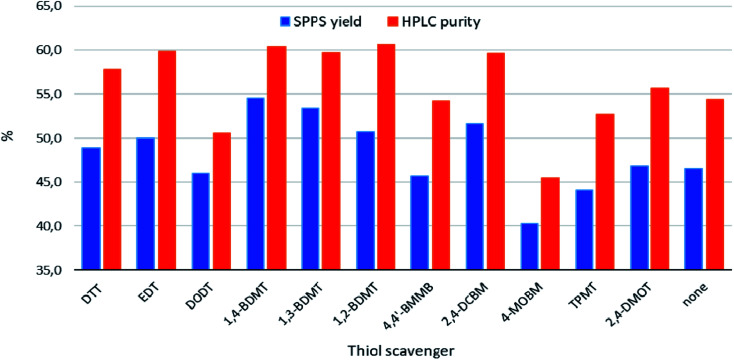
Chemical yields and HPLC purities of crude products obtained from TFA cleavages of exenatide resin (Scheme 1).

The yields and HPLC purities of the isolated crude peptides were determined (Fig. 2), see Table S1 in the Section 3 of the ESI† for the details of these experiments. These analyses revealed that cleavages using DODT, 4,4′-BMMB, the EDG containing 4-MOBM, bulky TPMT, aromatic 2,4-DMOT as well as no thiol were all inferior to the cleavages using the standard alkylthiols DTT and EDT. On the other hand, cleavages containing the three BDMT isomers as well as the EWG based 2,4-DCBM compared favorably to those carried out in the presence of the benchmark aliphatic thiols.

HPLC profiles of the crude peptides were fairly comparable and only differed in the levels of the cleavage related impurities (*vide infra*). Notable exceptions were the crudes from TFA cleavages using DODT, 4-MOBM and 2,4-DMOT as scavengers which contained significant peaks not found in the other crudes (see Section 5 of the ESI†). Specifically, according to LC-HRMS analyses for the DODT crude this impurity was the expected product of monoalkylation of methionine with *t*-butyl ethyl sulphide (+117 Da).^23^ On the other hand, in the crudes from cleavages using the EDG containing 4-MOBM and 2,4-DMOT we detected peptide–scavenger adducts accompanied by a loss of H_2_S. As the malodorous liquids EDT and 1,3-BDMT as well as toxic 2,4-DCBM are undesirable from the environment, health and safety (EHS) standpoints DTT, 1,2- and 1,4-BDMT were deemed as overall best scavengers among the thiols tested.

Next, we set out to investigate the content of specific cleavage related impurities by LC-HRMS. A peptide of exenatide length often contains several byproducts at a given retention time (*R*_t_) in a UV chromatogram rendering quantifications of the amounts of impurities by simple peak area integrations unreliable. We therefore opted to assess the crude materials by extracted ion chromatography-mass spectrometry (EIC-MS) in which only peaks pertaining to the specific impurities are extracted and integrated, giving a more accurate view of the content of specific impurities. Thus, we examined the content of some of the common cleavage related impurities which could be expected in crude peptides based on the AAs present in exenatide and PGs used for protection of the side chains. Specifically, we assessed the content of (i) *t*-Bu (+56 Da) adducts formed either by incomplete *t-*Bu removals and/or migrations of *t*-Bu cations from side chains to other positions in the peptide^19^ (ii) SO_3_ adducts (+80 Da)^25^ stemming from the Pbf group^29^ (iii) Pbf adducts (+252 Da) formed either by incomplete Pbf removals and/or migrations of the Pbf group from side chains to other positions in the peptide^30^ (iv) Met to Met(O) oxidation (+16 Da)^28^ (v) Met to HCys demethylation (−14 Da)^31^ (vi) primary Trp oxidants (+16 Da) *i.e.* 5-hydroxytryptophan (5-HTP) and oxindolylalanine (Oia).^32^

**Fig. 3:**
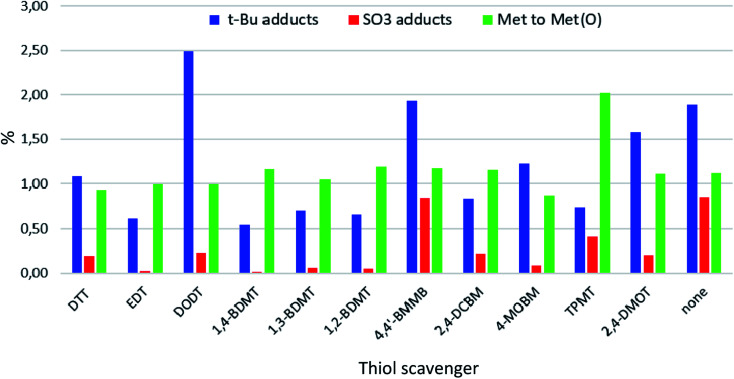
EIC-MS contents of *t*-Bu and SO_3_ adducts and Met to Met(O) oxidations in exenatide crudes obtained from TFA cleavages of exenatide resin (Scheme 1).

We summarized the EIC-MS results for all impurities examined (see Fig. 3 and Tables S27 and S28 in the ESI†) and found that with regards to minimizing the formation of peptide-*t-*Bu adducts 1,4-BDMT proved to be the most efficient (0.54%). As *t-*Bu adducts (+56 Da) are frequently encountered critical impurities in peptide medicines^33^ the fact that 1,4-BDMT was the best scavenger in terms of reducing the content of this byproduct class illustrates the value of this reagent for sustainable peptide manufacturing. In other words, replacing the currently used scavengers with 1,4-BDMT would result in more DSP amenable crude peptide products thereby allowing for the use of simpler, greener purification processes. Further, EDT, TPMT, 1,2- and 1,3-BDMT were slightly inferior (0.61–0.74%) to 1,4-BDMT while the content of *t*-Bu adducts in all remaining crudes was >0.8%. With respect to suppressing the content of *t-*Bu adducts it is worth noting that crude peptides stemming from cleavages of Lys(Boc) containing peptide resins bear +56 Da impurities formed by an intramolecular *N*-Boc- > *N-t-*Bu migration.^34^ Such +56 Da impurities are not amenable to minimization by altering the composition of the cleavage cocktail and are best addressed by replacing the Lys(Boc) moieties in the resin with Lys(Trt) counterparts.^35^

**Fig. 4:**
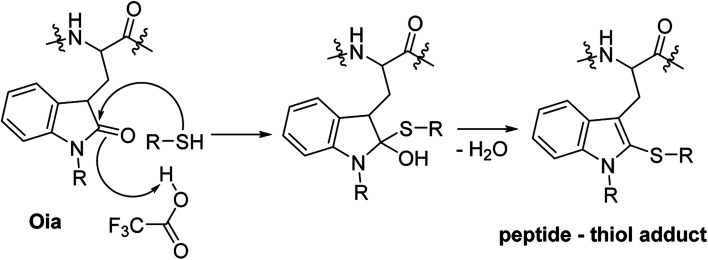
A schematic representation of the proposed mechanism of formation of peptide–thiol adduct during TFA cleavage of a Trp containing peptide resin *via* a thiol attack on Oia impurity. R = Boc, CO_2_H or H.

Furthermore, we found that 1,4-BDMT and EDT were the best scavengers in terms of minimizing the sulfonation of the peptide (0.02%). 4-MOBM, 1,2- and 1,3-BDMT were only slightly worse (0.05–0.07%) while the sulfonation content in all others crudes was >0.2%. On the other hand, with the exception of TPMT (∼2%) the Met(O) content was roughly the same for all other crudes (∼1%) showing that none of the thiols tested minimized the Met(O) content appreciably. We therefore propose that suppressing Met(O) in TFA cleavages is best accomplished by using some of the scavengers developed specifically for this side reaction.^28,36^ Further, we found that the content of add on Pbf and Met to HomoCys impurities was negligible (<0.1%) for all crudes examined. Finally, we examined the content of primary Trp oxidation impurities which have the same MW as Met(O) but are easily distinguishable from the latter by means of a large *R*_t_ difference (see ESI† Section 6). Thus, the content of 5-HTP + Oia oxidants for all crudes was determined to be 0.28–0.43%. Interestingly, the content of these +16 Da Trp oxidants was actually higher for cleavages containing some of the thiol scavengers (*e.g.* 0.38% for DTT) than for the cleavage carried in the absence of a thiol reagent (0.32%). This is in contrast with reports stating that during peptide resin TFA cleavages Trp is susceptible to oxidation with thiol scavenger such as DTT acting as a suppressant of the oxidative process.^37^ We reasoned that perhaps a thiol in a TFA cleavage of a peptide resin could play a different role in altering the 5-HTP + Oia content than preventing the Trp oxidation. To examine this hypothesis we carried out an EIC-MS analysis on the crude exenatide materials searching for masses of adducts of the peptide with thiol scavengers (MW of exenatide + MW of RSH − 2 Da). Remarkably, for cleavages containing EDT, DODT, 1,3- and 1,4-BDMT, 2,4-DMOT, 2,4-DCBM and 4-MOBM minute amounts (0.02–0.13%) of the peptide–thiol adducts were detected while no such compounds were seen in the crudes from cleavages using the bulky TPMT, poorly TFA soluble 4,4′-BMMB and oxidation susceptible DTT and 1,2-BDMT. While formation of peptide–thiol adducts during cleavages of Cys containing peptide resins is well precedented,^38^ this side reaction is less known in Cys free peptides, albeit formation of dithioacetal adducts during cleavages of peptides containing a keto function has been reported.^39^ To shed light on the formation of these unusual peptide–thiol adducts we carried out a tandem mass spectrometry (MS/MS) analysis on one of these peptide–thiol species (exenatide–DCBM adduct, see ESI† Section 7) and determined that the DCBM function resides on the Trp residue. While electrophilic attack on Trp during peptide resin TFA cleavages is well documented^40^ the reaction of a R–SH nucleophile with the electron rich Trp moiety is much less likely. We therefore propose that these peptide–thiol adducts are formed *via* an attack of a thiol on the keto function of the Oia impurity in the crude peptide followed by a loss of water to provide the peptide–thiol adduct (Fig. 4). To our knowledge this reaction is not known in peptide synthesis *per se*, albeit addition of thiols to simple oxindole systems followed by aromatization has nonetheless been reported.^41^ In other words we suggest that thiol scavengers in TFA cleavages of peptide resins do not prevent Trp oxidation but rather undergo a reaction with Oia impurity in which sense the thiol appears to suppress oxidation. In fact, although we did not observe an exenatide–DTT adduct (+152 Da) in the crude peptide from the cleavage of exenatide resin containing DTT we have previously detected formation of peptide–DTT adducts in TFA cleavages of Trp containing peptide resins, in particular when large amounts of DTT as a scavenger was used (data not shown). It is worth noting that these peptide–DTT adducts always eluted very close to the product peak and thus posed a significant challenge during purification of crude peptide products. On the other hand, the exenatide–BT adducts observed herein always eluted far from the main peak (see ESI† Section 6) and as such do not have a negative impact on DSP amenability.

**Fig. 5:**
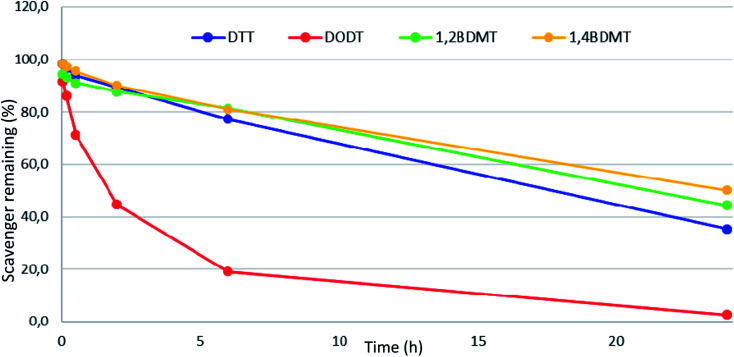
Stability of DTT, DODT, 1,2-BDMT and 1,4-BDMT in TFA/TIS/H_2_O (95 : 3 : 2, v/v/v).

Having evaluated the attributes of a series of BT scavengers in cleavages of exenatide peptide resin we examined properties of the most promising BT compounds (1,4- and 1,2-BDMT) under the conditions of peptide resin TFA cleavages. It has been shown that aliphatic thiols such as DTT decompose during TFA cleavages which decreases their scavenging ability while also forming poorly UV visible scavenger adducts.^24^ We therefore evaluated stability of 1,4 and 1,2-BDMT in a conc. TFA solution together with alkylthiols DTT and DODT as benchmarks in which we found that the order of stability was 1,4-BDMT > 1,2-BDMT > DTT ⋙ DODT (Fig. 5). Conceivably, the higher stability of 1,4-BDMT compared to the other thiols contributed to its scavenging efficiency in the TFA cleavage of exenatide resin (*vide supra*). Furthermore, regarding reagent solubility we found that adequate solubility (5% w/v) of 1,4-BDMT in TFA could be attained while 1,2-BDMT in TFA at 5% w/v remained only partially soluble even after 24 h of vigorous shaking. Again, it is conceivable that the higher solubility in TFA contributed to the better characteristics of 1,4-BDMT as a scavenger in the TFA cleavage of the exenatide resin. With respect to solubility it is worth noting that a precipitation was observed during TFA cleavages carried out in the presence of some of the BTs we examined. In fact, we isolated these precipitates from TFA cleavages of exenatide peptide resin using 1,2-, 1,3- and 1,4-BDMT and found that these materials were not BTs but rather adducts of BTs with PGs^42^ liberated from the peptide resin (see ESI† Section 9). These poorly TFA soluble compounds were easily filtered off together with the spent resin after the cleavage.

## Conclusions

In summary we assessed a series of BTs as scavengers for TFA cleavages of peptide resins and found that this class of thiols encompasses useful alternatives for aliphatic thiols currently used as state-of-the-art scavengers for peptide resin cleavages. Specifically, we found that 1,4-benzendimethanethiol (1,4-BDMT) used as a scavenger in a cleavage of exenatide peptide resin furnished the crude product in improved yield and purity compared to the cleavages carried out in the presence of standard aliphatic thiol reagents.

Importantly, 1,4-BDMT was the best thiol tested in terms of minimizing the content of critical impurities, which vastly improves crude peptide DSP amenability and thereby has a positive effect on the overall sustainability of synthetic peptides accessed by SPPS.

1,4-BDMT is easily UV detectable, exhibits suitable solubility and stability in TFA and can be sourced cost efficiently on scales required for GMP production of therapeutic peptides. Studies are underway aiming at implementing 1,4-BDMT to commercial manufacturing as well as examining additional BTs as scavengers and peptide resins as substrates.

Finally, we report that during TFA cleavages of Trp containing peptide resins an unprecedented formation of peptide–thiol adducts takes place, conceivably *via* a thiol attack on an Oia impurity in the crude peptide. We propose that this formation of peptide–thiol adducts may have been previously mistakenly interpreted as a thiol suppressing Trp oxidation during peptide resin cleavages. As Trp oxidants are commonly encountered critical impurities in therapeutic peptides^33^ further work aiming at improved understanding of factors governing Trp oxidation in peptide chemistry is underway.

## Experimental

### Materials

All reagents, reactants and solvents were from standard suppliers of raw materials for peptide manufacturing and were used as such.

### Methods

#### SPPS of exenatide resin

The SPPS of exenatide peptide resin was carried out using standard Fmoc/*t-*Bu SPPS methods,^17,18^ see ESI† Section 2 for details.

#### TFA cleavages of exenatide resin

All cleavages of the exenatide peptide resin herein were carried out as follows: 200 mg of the resin was weighed into a fritted PTFE syringe followed by addition of 60 μL triisopropylsilane (TIS), 40 μL water and a thiol amount of which is specified in Table S1.† Next, 1.84 mL of trifluoroacetic acid was added, and the resulting reaction mixture was shaken (320 rpm) for 2 h at rt. The supernate containing the crude peptide was then passed through the frit and into 30 mL of ice cold diethyl ether (DEE). The precipitated product was centrifuged for 10 min at 4000 rpm upon which the supernate was decanted off, additional 30 mL DEE was added, the resulting slurry was shaken vigorously for 2 min after which the centrifugation was repeated. The supernate was decanted off again and the residual off-white crude peptide was dried to constant weight *in vacuo*.

#### Assessment of thiol stability in TFA/TIS/H_2_O

The thiol (0.39 mmol) was added to TFA (1.84 mL), TIS (60 μL) and water (40 μL) and the resulting mixture was shaken at rt (300 rpm). 50 μL aliquots were taken out from the reaction mixture over time, diluted with MeCN (1.0 mL) and analyzed by HPLC.

## Conflicts of interest

There are no conflicts to declare.

## Supplementary Material

RA-009-C9RA08553J-s001
