# Supplementary material for: 1,4-Benzenedimethanethiol (1,4-BDMT) as a scavenger for greener peptide resin cleavages
Source: RSC Adv. 2019 Nov 28;9(67):38928–34. doi: 10.1039/c9ra08553j (PMC9075962; doi:10.1039/c9ra08553j)
Supplement: RA-009-C9RA08553J-s001 [file RA-009-C9RA08553J-s001.pdf]

Electronic Supplementary Material (ESI) for RSC Advances.
This journal is © The Royal Society of Chemistry 2019

**Electronic Supplementary Information (ESI)**

**1,4-Benzenedimethanethiol (1,4-BDMT) as a scavenger for greener peptide resin cleavages**

Jan Pawlas,* Thomas Svensson and Jon H. Rasmussen
PolyPeptide Group, Limhamnsvägen 108, PO BOX 30089, 20061 Limhamn, Sweden
*Corresponding author. E-mail: jan.pawlas@polypeptide.com

Table of Contents

1. General information …………………………………………………………………………… S2
2. SPPS………….. …………………………………………………………………………… S3
3. TFA cleavages……………………………………………………………………………….. S3
4. HPLC analyses of exenatide crudes……………….. ……………………………...………..... S5
5. LC-HRMS analyses of exenatide crudes ………...……………………………………..……. S18
6. EIC-MS analyses of exenatide crudes ……………………………………………….……. S48
7. MS-MS analyses of exenatide from TFA cleavage using 2,4-DCBM as scavenger…..…. S133
8. Assessment of thiol stability in TFA/TIS/$H_2O$…………...……………………………..… S137
9. Assessment of precipitation during TFA cleavages using BTs as scavengers…………… S140
10. Assessment of UV visibility (220 nm) of DTT and 1,4-BDMT……………………...…….S143

## 1. General information

All reagents, reactants and solvents were from standard suppliers of raw materials for peptide synthesis and were used as such. All HPLC analyses were carried out on an Agilent 1100 or a Waters Alliance instruments. MS analyses were carried out an Agilent Q-TOF mass spectrometer, (Agilent, Santa Clara, CA, USA). All LC-MS analyses were performed on a tandem liquid chromatography mass spectrometry system consisting of an Agilent 1290, 1200 bar system with DAD, connected to an Agilent quadrupole time-of-flight (Q-TOF) mass spectrometer. The mass spectrometry system was operated in a positive mode using electron spray ionization (ESI), mass range 20-3200, mass accuracy at 0.02 u, resolution up to 20000 ppm. The following source settings were used: gas temp 300 $^{o}$C, gas flow 8 l/min, nebulizer 30psig, sheat gas temperature 350 $^{o}$C and sheat gas flow 7.5 l/min. Analytical separations were achieved using a Waters Acquity UPLC instrument. SPPS of exenatide resin was carried out in a glass SPPS reactor equipped with an overhead mechanical stirrer. All peptide yield determinations were carried out by HPLC methods employing sample of the purified peptides as reference standards. The product yields were not corrected for the peptide content in the isolated materials.

## 2. SPPS

The synthesis of the exenatide resin was carried out at room temperature throughout and commenced with 200 mmol of 0.55 M DEG AM[1] resin (364 g). All Fmoc removals were carried out using piperidine in DMF (20% v/v, 10 + 20 min each) and all AA couplings were performed in DMF using HOBtx$H_2O$[2] and DIC[3] as coupling agents. Following starting materials were used for the couplings in the synthesis: 1) Ramage linker;[4] 2) Fmoc-Ser(*t*-Bu)-OH; 3) Fmoc-Pro-OH; 4) Fmoc-Pro-OH; 5) Fmoc-Pro-OH; 6) Fmoc-Ala-OHx$H_2O$; 7) Fmoc-Gly-OH; 8) Fmoc-Ser(*t*-Bu)-OH; 9) Fmoc-Ser(*t*-Bu)-OH; 10) Fmoc-Pro-OH; 11) Fmoc-Gly-OH; 12) Fmoc-Gly-OH; 13) Fmoc-Asn(Trt)-OH; 14) Fmoc-Lys(Boc)-OH; 15) Fmoc-Leu-OH; 16) Fmoc-Trp(Boc)-OH; 17) Fmoc-Glu(OtBu)-OHx$H_2O$; 18) Fmoc-Ile-OH; 19) Fmoc-Phe-OH; 20) Fmoc-Leu-OH; 21) Fmoc-Arg(Pbf)-OH; 22) Fmoc-Val-OH; 23) Fmoc-Ala-OHx$H_2O$; 24) Fmoc-Glu(O*t*-Bu)-OH; 25) Fmoc-Glu(O*t*-Bu)-OH; 26) Fmoc-Glu(O*t*-Bu)-OH; 27) Fmoc-Met-OH; 28) Fmoc-Gln(Trt)-OH; 29) Fmoc-Lys(Boc)-OH; 30) Fmoc-Ser(*t*-Bu)-OH; 31) Fmoc-Leu-OH; 32) Fmoc-Asp(O*t*-Bu)-OH; 33) Fmoc-Ser(*t*-Bu)-OH; 34) Fmoc-Thr(*t*-Bu)-OH; 35) Fmoc-Phe-OH; 36) Fmoc-Thr(*t*-Bu)-OH; 37) Fmoc-Gly-OH; 38) Fmoc-Glu(O*t*-Bu)-OH; 39) Boc-His(Trt)-Gly-OH. 2 – 3 equiv of the AAs and coupling reagents (vs the base resin) were used throughout and the couplings were allowed to proceed until completion was attained according to qualitative color tests[5-6] upon which the coupling solutions were capped using acetic acid anhydride ($Ac_2O$) an N-methylmorpholine (NMM). After each coupling and Fmoc removal the intermediate peptide resin was thoroughly washed with DMF. Upon the completion of the synthesis the final exenatide peptide resin was washed with isopropanol (*i*-PrOH) and dried *en vacuo* which afforded 1381 g of the title resin. As the scale of the synthesis was 200 mmol the theoretically attainable amount exenatide per gram resin was determined to be 200/1381 = 0.145 mmol ~ 608 mg.

## 3. TFA cleavages

For the amounts of the isolated exenatide crudes see Table S1. The yield of each experiment was calculated as follows: based on the HPLC analyses carried out for all exenatide crudes prepared (see below) and using a sample of authentic exenatide API as a reference the amount of exenatide in the isolated crude peptide was calculated. The yield of each experiment in Scheme 1 was then calculated as ((amount of exenatide obtained per 200 mg resin)/(theoretically attainable amount of exenatide per 200 mg resin))*100, (see Table S1). The reproducibility of the TFA cleavage results shown in Table S1 was verified by carrying out selected cleavages in duplicate (run 1, DTT; run 6, 1,2-BDMT and run 8 2,4-DCBM) and, for run 4 (1,4-BDMT), in quadruplicate. In all cases the amounts of the isolated peptides were within 0.5 mg (~1%) of the values stated in Table S1.

**Table S1.** Overview of thiols used, isolated amounts and yields and purities of exenatide crudes for TFA cleavages shown in Scheme 1.

| run | thiol used | | | | | | | exenatide product obtained | | | |
|---|---|---|---|---|---|---|---|---|---|---|---|
| | scavenger | cas nr | amount (mg) | amount (mmol) | MW | density (g/mL) | amount (µL) | isolated amount of crude peptide (mg) | amount of exenatide formed (mg)[1] | yield (%)[2] | HPLC purity (%)[3] |
| 1 | DTT | 3483-12-03 | 60,0 | 0,39 | 154,25 | n.d. | n.d. | 101 | 59,29 | 48,86 | 57,77 |
| 2 | EDT | 540-63-6 | 36,7 | 0,39 | 94,20 | 1,123 | 32,7 | 100 | 60,68 | 50,00 | 59,91 |
| 3 | DODT | 14970-87-7 | 71,1 | 0,39 | 182,30 | 1,12 | 63,5 | 107 | 55,86 | 46,04 | 50,59 |
| 4 | 1,4-BDMT | 105-09-9 | 66,4 | 0,39 | 170,29 | n.d. | n.d. | 106 | 68,68 | 56,60 | 60,53 |
| 5 | 1,3-BDMT | 41563-69-3 | 66,4 | 0,39 | 170,29 | 1,15 | 57,8 | 104 | 66,77 | 55,03 | 59,67 |
| 6 | 1,2-BDMT | 41383-84-0 | 66,4 | 0,39 | 170,29 | n.d. | n.d. | 100 | 61,49 | 50,67 | 60,34 |
| 7 | 4,4'-BMMB | 43012-19-7 | 96,1 | 0,39 | 246,39 | n.d. | n.d. | 102 | 55,48 | 45,72 | 54,22 |
| 8 | 2,4-DCBM | 59293-67-3 | 150,6 | 0,78 | 193,10 | 1,36 | 110,7 | 104 | 62,66 | 51,64 | 59,62 |
| 9 | 4-MOBM | 6258-60-2 | 120,3 | 0,78 | 154,23 | 1,112 | 108,2 | 109 | 48,86 | 40,27 | 45,27 |
| 10 | TPMT | 3695-77-0 | 215,6 | 0,78 | 276,40 | n.d. | n.d. | 94 | 53,46 | 44,06 | 52,97 |
| 11 | 2,4-DMOT | 18906-37-1 | 132,8 | 0,78 | 170,23 | 1,186 | 112,0 | 100 | 56,79 | 46,80 | 55,67 |
| 12 | none | none | none | none | none | none | none | 94 | 56,43 | 46,51 | 54,36 |

[1]amount of crude peptide x content of exenatide in crude (%w/w); [2]((amount of exenatide formed from 200 mg peptide resin)/(theoretically attainable amount of exenatide from 200 mg peptide resin))x100; [3]See section 4 of this ESI for details.

## 4. HPLC analyses of exenatide crudes

All HPLC analyses and yield quantifications were carried out as follows: 10.0 mg of each crude peptide was dissolved in 10.0 mL of AcOH/$H_2O$/MeCN (10:40:50). The resulting homogeneous solutions were shaken at 40 °C for 30 min to decarboxylate the Trp residue of the peptide which is partially in its carbamate form[7] after TFA cleavage/DEE precipitation. After the decarboxylation HPLC analyses on all exenatide crudes were carried out using the following analytical system: column: Waters XSelect CSH130 C18 2.5 µm 4.6x150mm; detection wavelength: 220 nm, column temperature: 30 °C; injection volume: 5 µL for the yield quantifycations, then varying to produce an HPLC overlay in which main peaks of all crudes are comparable; sampler temperature:10°C; flow: 0.5 ml/min; mobile phase A: 0.1 % TFA in water, mobile phase B: 0.08 % TFA in 90% MeCN/10 %water. Gradient (Time(min), %B): 0, 0; 40, 100; 54, 100; 55, 0; 62, 0. Main peak at retention time (Rt) 22 min, integrated area 18 – 30 min. HPLC analyses for selected exenatide crudes (Table S1 runs 1, 4, 6 and 8), were carried in duplicate and in all cases the two chromatograms obtained for the same crude peptide were unduistingshable.

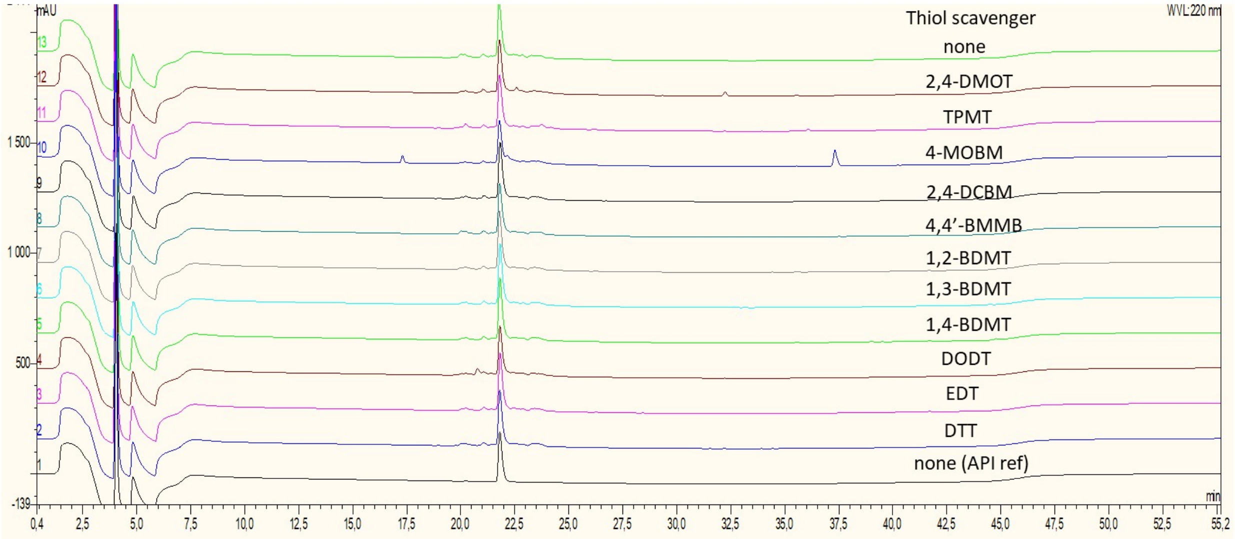


**Figure S1.** Overlay of HPLC chromatograms of exenatide crudes prepared using different thiols as scavengers in TFA cleavages of exenatide peptide resin.

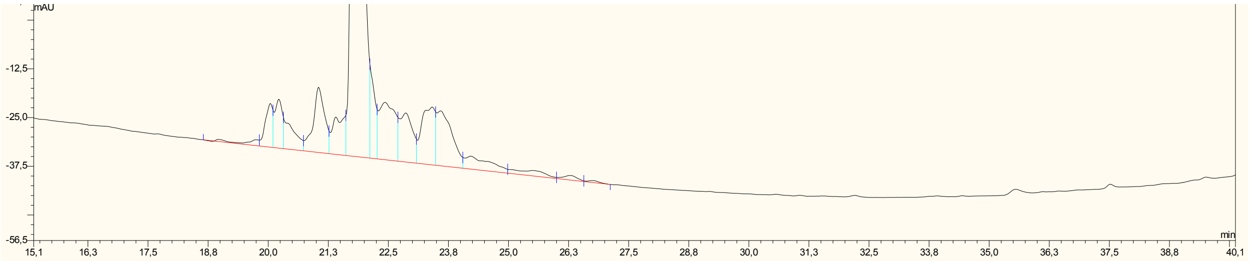

**Figure S2.** HPLC chromatogram of exenatide crude prepared using DTT as thiol scavenger in TFA cleavage of exenatide peptide resin.

**Table S2.** Area% for integrated peaks.

| No. | Peakname | Ret.Time min | Area mAU*min | Amount | Type | Height mAU | Rel.Area % | Resolution |
|---|---|---|---|---|---|---|---|---|
| 1 | n.a. | 19,783 | 0,4913 | n.a. | BM * | 1,506 | 0,51 | n.a. |
| 2 | n.a. | 20,050 | 1,9268 | n.a. | M * | 11,206 | 2,00 | n.a. |
| 3 | n.a. | 20,217 | 2,2878 | n.a. | M * | 12,552 | 2,38 | n.a. |
| 4 | n.a. | 20,333 | 2,0557 | n.a. | M * | 7,299 | 2,14 | n.a. |
| 5 | n.a. | 21,050 | 4,6484 | n.a. | M * | 16,736 | 4,83 | n.a. |
| 6 | n.a. | 21,617 | 2,8965 | n.a. | M * | 10,291 | 3,01 | n.a. |
| *7* | *Main peak* | *21,800* | *55,5918* | *n.a.* | *M ** | *255,917* | *57,77* | *n.a.* |
| 8 | n.a. | 22,133 | 2,6973 | n.a. | M * | 22,391 | 2,80 | n.a. |
| 9 | n.a. | 22,433 | 5,7592 | n.a. | M * | 14,732 | 5,99 | n.a. |
| 10 | n.a. | 22,867 | 3,9836 | n.a. | M * | 12,598 | 4,14 | n.a. |
| 11 | n.a. | 23,417 | 4,7995 | n.a. | M * | 14,839 | 4,99 | n.a. |
| 12 | n.a. | 23,583 | 5,4207 | n.a. | M * | 14,095 | 5,63 | n.a. |
| 13 | n.a. | 24,217 | 2,1445 | n.a. | M * | 3,325 | 2,23 | n.a. |
| 14 | n.a. | 25,533 | 0,9939 | n.a. | M * | 1,411 | 1,03 | n.a. |
| 15 | n.a. | 26,300 | 0,3922 | n.a. | M * | 1,186 | 0,41 | 1,05 |
| 16 | n.a. | 26,783 | 0,1322 | n.a. | MB* | 0,512 | 0,14 | n.a. |
| **Total:** | | | **96,2213** | **0,0000** | | **400,596** | **100,00** | |

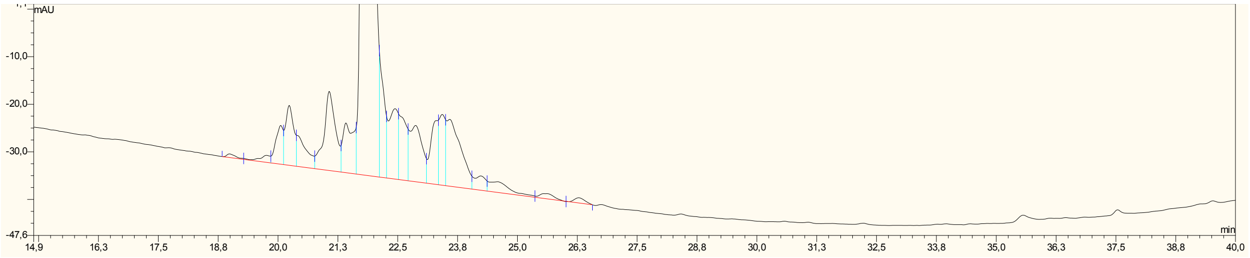

**Figure S3.** HPLC chromatogram of exenatide crude prepared using EDT as thiol scavenger in TFA cleavage of exenatide peptide resin.

**Table S3.** Area% for integrated peaks.

| No. | Peakname | Ret.Time min | Area mAU*min | Amount | Type | Height mAU | Rel.Area % | Resolution |
|---|---|---|---|---|---|---|---|---|
| 1 | n.a. | 19,000 | 0,1677 | n.a. | BM * | 0,761 | 0,17 | n.a. |
| 2 | n.a. | 19,767 | 0,3720 | n.a. | M * | 1,473 | 0,39 | n.a. |
| 3 | n.a. | 20,050 | 1,4413 | n.a. | M * | 8,130 | 1,50 | n.a. |
| 4 | n.a. | 20,233 | 2,5289 | n.a. | M * | 12,606 | 2,64 | n.a. |
| 5 | n.a. | 20,400 | 1,6244 | n.a. | M * | 6,482 | 1,69 | n.a. |
| 6 | n.a. | 21,067 | 4,6770 | n.a. | M * | 16,640 | 4,88 | n.a. |
| 7 | n.a. | 21,417 | 2,7819 | n.a. | M * | 10,475 | 2,90 | n.a. |
| 8 | Main peak | 21,817 | 57,4617 | n.a. | M * | 264,322 | 59,91 | n.a. |
| 9 | n.a. | 22,133 | 2,8644 | n.a. | M * | 24,035 | 2,99 | n.a. |
| 10 | n.a. | 22,450 | 3,3966 | n.a. | M * | 14,797 | 3,54 | n.a. |
| 11 | n.a. | 22,533 | 2,5207 | n.a. | M * | 13,555 | 2,63 | n.a. |
| 12 | n.a. | 22,883 | 3,6933 | n.a. | M * | 11,823 | 3,85 | n.a. |
| 13 | n.a. | 23,350 | 2,5164 | n.a. | M * | 13,550 | 2,62 | n.a. |
| 14 | n.a. | 23,433 | 2,1561 | n.a. | M * | 14,882 | 2,25 | n.a. |
| 15 | n.a. | 23,583 | 5,0835 | n.a. | M * | 14,043 | 5,30 | n.a. |
| 16 | n.a. | 24,233 | 0,8313 | n.a. | M * | 3,009 | 0,87 | n.a. |
| 17 | n.a. | 24,600 | 1,1452 | n.a. | M * | 2,252 | 1,19 | n.a. |
| 18 | n.a. | 25,633 | 0,3668 | n.a. | M * | 1,107 | 0,38 | 1,34 |
| 19 | n.a. | 26,283 | 0,2773 | n.a. | MB* | 1,096 | 0,29 | n.a. |
| **Total:** | | | **95,9063** | **0,0000** | | **435,038** | **100,00** | |

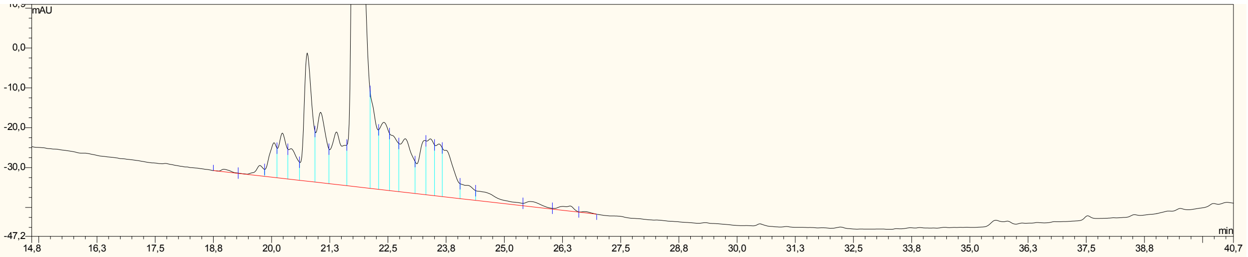


**Figure S4.** HPLC chromatogram of exenatide crude prepared using DODT as thiol scavenger in TFA cleavage of exenatide peptide resin.

**Table S4.** Area% for integrated peaks.

| No. | Peakname | Ret.Time min | Area mAU*min | Amount | Type | Height mAU | Rel.Area % | Resolution |
|---|---|---|---|---|---|---|---|---|
| 1 | n.a. | 18,983 | 0,1390 | n.a. | BM * | 0,639 | 0,14 | n.a. |
| 2 | n.a. | 19,750 | 0,4966 | n.a. | M * | 2,606 | 0,51 | n.a. |
| 3 | n.a. | 20,050 | 1,5724 | n.a. | M * | 8,704 | 1,61 | n.a. |
| 4 | n.a. | 20,233 | 2,1761 | n.a. | M * | 11,367 | 2,23 | n.a. |
| 5 | n.a. | 20,417 | 1,6455 | n.a. | M * | 7,686 | 1,68 | n.a. |
| 6 | n.a. | 20,767 | 6,3261 | n.a. | M * | 32,172 | 6,47 | n.a. |
| 7 | n.a. | 21,050 | 4,1501 | n.a. | M * | 17,669 | 4,25 | n.a. |
| 8 | n.a. | 21,400 | 4,0661 | n.a. | M * | 13,178 | 4,16 | n.a. |
| 9 | *Main peak* | *21,817* | *49,4428* | *n.a.* | *M ** | *225,629* | *50,59* | *n.a.* |
| 10 | n.a. | 22,133 | 3,4293 | n.a. | M * | 22,858 | 3,51 | n.a. |
| 11 | n.a. | 22,417 | 3,7277 | n.a. | M * | 16,967 | 3,81 | n.a. |
| 12 | n.a. | 22,550 | 2,6752 | n.a. | M * | 14,061 | 2,74 | n.a. |
| 13 | n.a. | 22,867 | 4,0145 | n.a. | M * | 13,445 | 4,11 | n.a. |
| 14 | n.a. | 23,283 | 2,4941 | n.a. | M * | 13,579 | 2,55 | n.a. |
| 15 | n.a. | 23,417 | 2,5033 | n.a. | M * | 14,149 | 2,56 | n.a. |
| 16 | n.a. | 23,600 | 2,1313 | n.a. | M * | 13,104 | 2,18 | n.a. |
| 17 | n.a. | 23,683 | 3,2772 | n.a. | M * | 11,897 | 3,35 | n.a. |
| 18 | n.a. | 24,067 | 1,1064 | n.a. | M * | 3,680 | 1,13 | n.a. |
| 19 | n.a. | 24,400 | 1,3850 | n.a. | M * | 2,396 | 1,42 | n.a. |
| 20 | n.a. | 25,600 | 0,5078 | n.a. | M * | 1,299 | 0,52 | 1,31 |
| 21 | n.a. | 26,417 | 0,4110 | n.a. | M * | 1,298 | 0,42 | 0,78 |
| 22 | n.a. | 26,767 | 0,0642 | n.a. | MB* | 0,312 | 0,07 | n.a. |
| **Total:** | | | **97,7417** | **0,0000** | | **448,695** | **100,00** | |

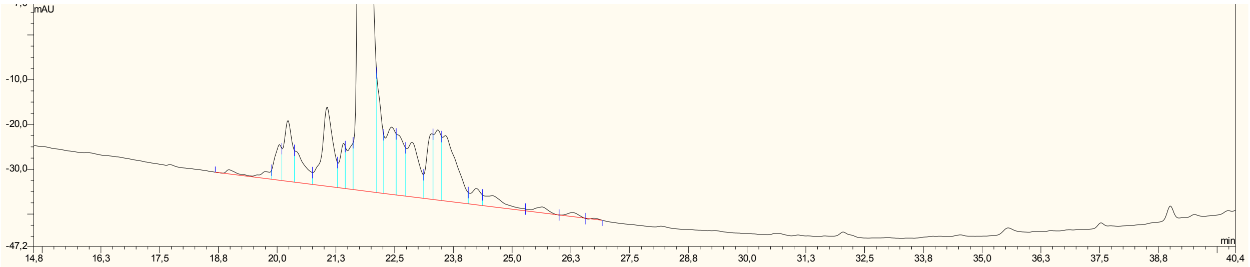


**Figure S5.** HPLC chromatogram of exenatide crude prepared using 1,4-BDMT as thiol scavenger in TFA cleavage of exenatide peptide resin.

**Table S5.** Area% for integrated peaks.

| No. | Peakname | Ret.Time min | Area mAU*min | Amount | Type | Height mAU | Rel.Area % | Resolution |
|---|---|---|---|---|---|---|---|---|
| 1 | n.a. | 19,883 | 0,6638 | n.a. | BM * | 2,032 | 0,65 | n.a. |
| 2 | n.a. | 20,050 | 1,2813 | n.a. | M * | 7,965 | 1,26 | n.a. |
| 3 | n.a. | 20,233 | 2,7023 | n.a. | M * | 13,569 | 2,67 | n.a. |
| 4 | n.a. | 20,383 | 1,7874 | n.a. | M * | 6,956 | 1,76 | n.a. |
| 5 | n.a. | 21,067 | 4,8134 | n.a. | M * | 17,658 | 4,75 | n.a. |
| 6 | n.a. | 21,417 | 1,3186 | n.a. | M * | 10,055 | 1,30 | n.a. |
| 7 | n.a. | 21,617 | 1,5354 | n.a. | M * | 10,453 | 1,51 | n.a. |
| *8* | *Main peak* | *21,817* | *61,3609* | *n.a.* | *M ** | *284,682* | *60,53* | *n.a.* |
| 9 | n.a. | 22,133 | 2,9074 | n.a. | M * | 24,452 | 2,87 | n.a. |
| 10 | n.a. | 22,433 | 3,6992 | n.a. | M * | 15,027 | 3,65 | n.a. |
| 11 | n.a. | 22,550 | 2,5114 | n.a. | M * | 13,497 | 2,48 | n.a. |
| 12 | n.a. | 22,867 | 3,6919 | n.a. | M * | 12,214 | 3,64 | n.a. |
| 13 | n.a. | 23,283 | 2,2277 | n.a. | M * | 14,629 | 2,20 | n.a. |
| 14 | n.a. | 23,417 | 2,7577 | n.a. | M * | 15,671 | 2,72 | n.a. |
| 15 | n.a. | 23,583 | 5,2699 | n.a. | M * | 14,600 | 5,20 | n.a. |
| 16 | n.a. | 24,233 | 0,8891 | n.a. | M * | 3,639 | 0,88 | n.a. |
| 17 | n.a. | 24,583 | 1,2416 | n.a. | M * | 2,478 | 1,22 | n.a. |
| 18 | n.a. | 25,650 | 0,4499 | n.a. | M * | 1,325 | 0,44 | 1,37 |
| 19 | n.a. | 26,317 | 0,2398 | n.a. | M * | 0,911 | 0,24 | 1,24 |
| 20 | n.a. | 26,767 | 0,0300 | n.a. | MB* | 0,242 | 0,03 | n.a. |
| **Total:** | | | **101,3788** | **0,0000** | | **472,055** | **100,00** | |

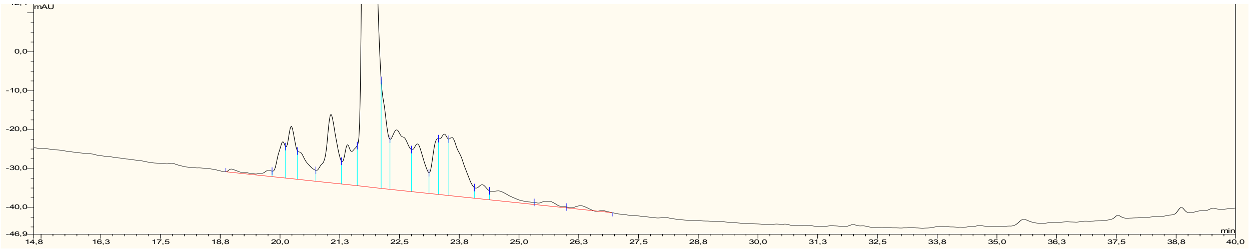

**Figure S6.** HPLC chromatogram of exenatide crude prepared using 1,3-BDMT as thiol scavenger in TFA cleavage of exenatide peptide resin.

**Table S6.** Area% for integrated peaks.

| No. | Peakname | Ret.Time min | Area mAU*min | Amount | Type | Height mAU | Rel.Area % | Resolution |
|---|---|---|---|---|---|---|---|---|
| 1 | n.a. | 19,767 | 0,5603 | n.a. | BM * | 1,505 | 0,55 | n.a. |
| 2 | n.a. | 20,067 | 1,6227 | n.a. | M * | 9,229 | 1,59 | n.a. |
| 3 | n.a. | 20,233 | 2,6089 | n.a. | M * | 13,445 | 2,56 | n.a. |
| 4 | n.a. | 20,383 | 1,8763 | n.a. | M * | 7,096 | 1,84 | n.a. |
| 5 | n.a. | 21,067 | 4,8558 | n.a. | M * | 17,618 | 4,77 | n.a. |
| 6 | n.a. | 21,617 | 2,8957 | n.a. | M * | 10,297 | 2,84 | n.a. |
| 7 | Main peak | 21,817 | 60,7964 | n.a. | M * | 279,629 | 59,67 | n.a. |
| 8 | n.a. | 22,133 | 3,4689 | n.a. | M * | 25,270 | 3,40 | n.a. |
| 9 | n.a. | 22,433 | 6,1194 | n.a. | M * | 15,448 | 6,01 | n.a. |
| 10 | n.a. | 22,883 | 3,5867 | n.a. | M * | 12,409 | 3,52 | n.a. |
| 11 | n.a. | 23,317 | 2,1002 | n.a. | M * | 14,325 | 2,06 | n.a. |
| 12 | n.a. | 23,433 | 3,2344 | n.a. | M * | 15,666 | 3,17 | n.a. |
| 13 | n.a. | 23,600 | 5,0669 | n.a. | M * | 15,008 | 4,97 | n.a. |
| 14 | n.a. | 24,233 | 0,9668 | n.a. | M * | 3,679 | 0,95 | n.a. |
| 15 | n.a. | 24,583 | 1,3356 | n.a. | M * | 2,537 | 1,31 | n.a. |
| 16 | n.a. | 25,633 | 0,4686 | n.a. | M * | 1,241 | 0,46 | 1,31 |
| 17 | n.a. | 26,300 | 0,3167 | n.a. | MB* | 0,946 | 0,31 | n.a. |
| Total: | | | 101,8803 | 0,0000 | | 445,348 | 100,00 | |

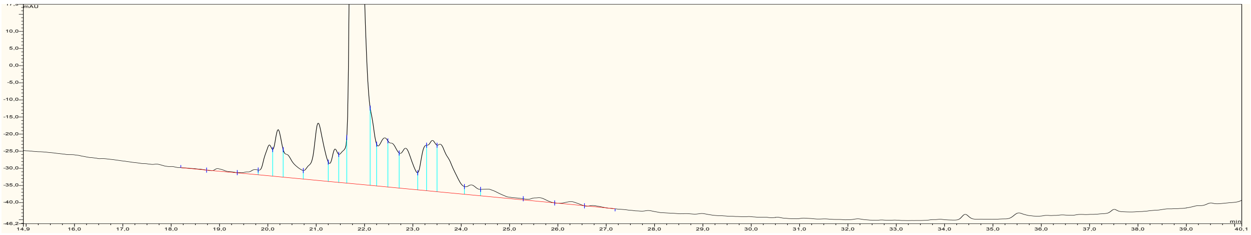

**Figure S7.** HPLC chromatogram of exenatide crude prepared using 1,2-BDMT as thiol scavenger in TFA cleavage of exenatide peptide resin.

**Table S7.** Area% for integrated peaks.

| No. | Peakname | Ret.Time min | Area mAU*min | Amount | Type | Height mAU | Rel.Area % | Resolution |
|---|---|---|---|---|---|---|---|---|
| 1 | n.a. | 18,233 | 0,0323 | n.a. | BM * | 0,012 | 0,03 | 3,80 |
| 2 | n.a. | 18,967 | 0,1611 | n.a. | bM * | 0,733 | 0,17 | n.a. |
| 3 | n.a. | 19,750 | 0,2994 | n.a. | M * | 1,434 | 0,31 | n.a. |
| 4 | n.a. | 20,033 | 1,6695 | n.a. | M * | 9,042 | 1,74 | n.a. |
| 5 | n.a. | 20,217 | 2,3812 | n.a. | M * | 13,745 | 2,48 | n.a. |
| 6 | n.a. | 20,333 | 1,9760 | n.a. | M * | 7,377 | 2,06 | n.a. |
| 7 | n.a. | 21,050 | 4,5035 | n.a. | M * | 16,730 | 4,68 | n.a. |
| 8 | n.a. | 21,400 | 1,6659 | n.a. | M * | 9,623 | 1,73 | n.a. |
| 9 | n.a. | 21,633 | 1,5785 | n.a. | M * | 13,306 | 1,64 | n.a. |
| *10* | *Main peak* | *21,783* | *58,0195* | *n.a.* | *M ** | *267,110* | *60,34* | *n.a.* |
| 11 | n.a. | 22,133 | 2,2961 | n.a. | M * | 21,267 | 2,39 | n.a. |
| 12 | n.a. | 22,417 | 3,0710 | n.a. | M * | 14,272 | 3,19 | n.a. |
| 13 | n.a. | 22,500 | 2,8346 | n.a. | M * | 13,174 | 2,95 | n.a. |
| 14 | n.a. | 22,850 | 3,5381 | n.a. | M * | 11,832 | 3,68 | n.a. |
| 15 | n.a. | 23,283 | 1,8078 | n.a. | M * | 13,289 | 1,88 | n.a. |
| 16 | n.a. | 23,400 | 3,0466 | n.a. | M * | 14,841 | 3,17 | n.a. |
| 17 | n.a. | 23,567 | 4,8084 | n.a. | M * | 13,965 | 5,00 | n.a. |
| 18 | n.a. | 24,217 | 0,8057 | n.a. | M * | 2,936 | 0,84 | n.a. |
| 19 | n.a. | 24,567 | 0,9904 | n.a. | M * | 2,183 | 1,03 | n.a. |
| 20 | n.a. | 25,633 | 0,3756 | n.a. | M * | 1,088 | 0,39 | 1,28 |
| 21 | n.a. | 26,283 | 0,2280 | n.a. | M * | 0,833 | 0,24 | 1,10 |
| 22 | n.a. | 26,750 | 0,0657 | n.a. | MB* | 0,319 | 0,07 | n.a. |
| **Total:** | | | **96,1546** | **0,0000** | | **449,111** | **100,00** | |

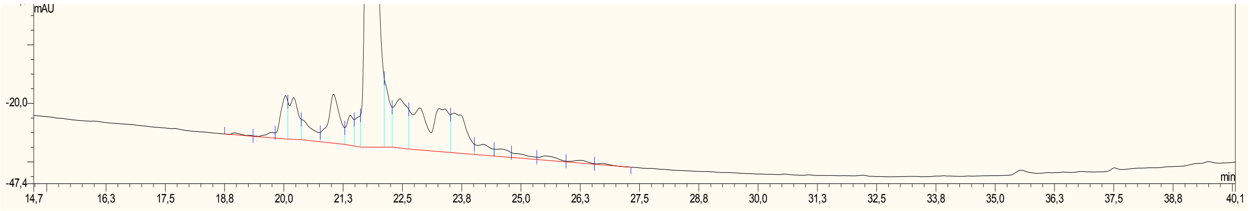


**Figure S8.** HPLC chromatogram of exenatide crude prepared using 4,4'-BMMB as thiol scavenger in TFA cleavage of exenatide peptide resin.

**Table S8.** Area% for integrated peaks.

| No. | Peakname | Ret.Time min | Area mAU*min | Amount | Type | Height mAU | Rel.Area % | Resolution |
|---|---|---|---|---|---|---|---|---|
| 1 | n.a. | 18,967 | 0,1490 | n.a. | BM * | 0,642 | 0,16 | n.a. |
| 2 | n.a. | 19,750 | 0,4015 | n.a. | M * | 1,825 | 0,42 | n.a. |
| 3 | n.a. | 20,033 | 2,4163 | n.a. | M * | 14,863 | 2,53 | n.a. |
| 4 | n.a. | 20,200 | 3,2014 | n.a. | M * | 14,211 | 3,36 | n.a. |
| 5 | n.a. | 20,383 | 1,8520 | n.a. | M * | 6,826 | 1,94 | n.a. |
| 6 | n.a. | 21,050 | 4,7202 | n.a. | M * | 16,756 | 4,95 | n.a. |
| 7 | n.a. | 21,400 | 1,7223 | n.a. | M * | 10,244 | 1,81 | n.a. |
| 8 | n.a. | 21,617 | 1,3201 | n.a. | M * | 10,868 | 1,38 | n.a. |
| *9* | *Main peak* | *21,800* | *51,6896* | *n.a.* | *M ** | *234,148* | *54,22* | *n.a.* |
| 10 | n.a. | 22,133 | 2,9680 | n.a. | M * | 22,081 | 3,11 | n.a. |
| 11 | n.a. | 22,450 | 5,3217 | n.a. | M * | 16,811 | 5,58 | n.a. |
| 12 | n.a. | 23,283 | 10,6612 | n.a. | M * | 14,681 | 11,18 | n.a. |
| 13 | n.a. | 23,600 | 5,0411 | n.a. | M * | 13,502 | 5,29 | n.a. |
| 14 | n.a. | 24,217 | 1,3088 | n.a. | M * | 3,685 | 1,37 | n.a. |
| 15 | n.a. | 24,600 | 0,8598 | n.a. | M * | 2,600 | 0,90 | n.a. |
| 16 | n.a. | 24,817 | 0,6265 | n.a. | M * | 1,533 | 0,66 | n.a. |
| 17 | n.a. | 25,533 | 0,6197 | n.a. | M * | 1,490 | 0,65 | n.a. |
| 18 | n.a. | 26,267 | 0,3353 | n.a. | M * | 0,948 | 0,35 | 0,97 |
| 19 | n.a. | 26,733 | 0,1195 | n.a. | MB* | 0,515 | 0,13 | n.a. |
| **Total:** | | | **95,3339** | **0,0000** | | **388,229** | **100,00** | |

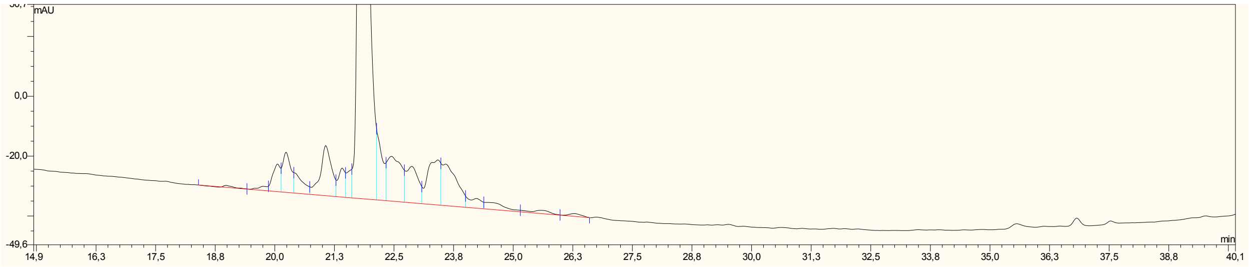


**Figure S9.** HPLC chromatogram of exenatide crude prepared using 2,4-DCBM as thiol scavenger in TFA cleavage of exenatide peptide resin.

**Table S9.** Area% for integrated peaks.

| No. | Peakname | Ret.Time min | Area mAU*min | Amount | Type | Height mAU | Rel.Area % | Resolution |
|---|---|---|---|---|---|---|---|---|
| 1 | n.a. | 18,983 | 0,1107 | n.a. | BM * | 0,656 | 0,12 | n.a. |
| 2 | n.a. | 19,867 | 0,3340 | n.a. | M * | 1,521 | 0,35 | n.a. |
| 3 | n.a. | 20,050 | 1,7190 | n.a. | M * | 9,207 | 1,80 | n.a. |
| 4 | n.a. | 20,233 | 2,6882 | n.a. | M * | 13,426 | 2,81 | n.a. |
| 5 | n.a. | 20,417 | 1,4779 | n.a. | M * | 6,768 | 1,54 | n.a. |
| 6 | n.a. | 21,067 | 4,5789 | n.a. | M * | 16,753 | 4,78 | n.a. |
| 7 | n.a. | 21,417 | 1,5734 | n.a. | M * | 9,685 | 1,64 | n.a. |
| 8 | n.a. | 21,617 | 1,1704 | n.a. | M * | 9,623 | 1,22 | n.a. |
| 9 | Main peak | 21,817 | 57,0613 | n.a. | M * | 260,434 | 59,62 | n.a. |
| 10 | n.a. | 22,150 | 3,2776 | n.a. | M * | 22,177 | 3,42 | n.a. |
| 11 | n.a. | 22,450 | 5,1383 | n.a. | M * | 14,971 | 5,37 | n.a. |
| 12 | n.a. | 22,867 | 3,6771 | n.a. | M * | 12,173 | 3,84 | n.a. |
| 13 | n.a. | 23,417 | 4,6902 | n.a. | M * | 15,063 | 4,90 | n.a. |
| 14 | n.a. | 23,583 | 5,2100 | n.a. | M * | 13,876 | 5,44 | n.a. |
| 15 | n.a. | 24,017 | 1,0980 | n.a. | M * | 3,451 | 1,15 | n.a. |
| 16 | n.a. | 24,583 | 1,1189 | n.a. | M * | 2,299 | 1,17 | n.a. |
| 17 | n.a. | 25,633 | 0,5090 | n.a. | M * | 1,126 | 0,53 | 1,22 |
| 18 | n.a. | 26,300 | 0,2830 | n.a. | MB* | 0,940 | 0,30 | n.a. |
| Total: | | | 95,7159 | 0,0000 | | 414,149 | 100,00 | |

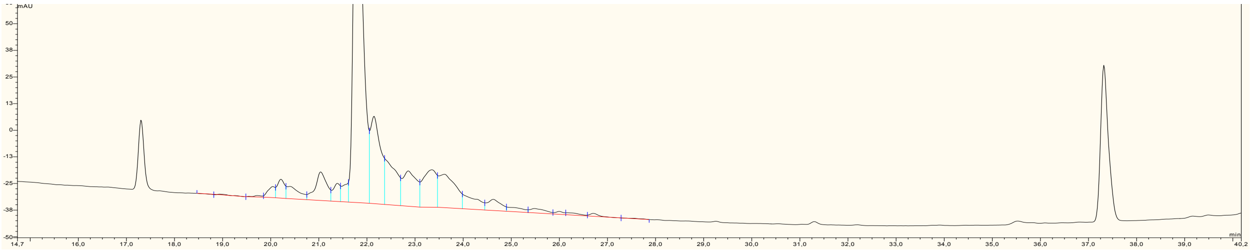

**Figure S10.** HPLC chromatogram of exenatide crude prepared using 4-MOBM as thiol scavenger in TFA cleavage of exenatide peptide resin.

**Table S10.** Area% for integrated peaks.

| No. | Peakname | Ret.Time min | Area mAU*min | Amount | Type | Height mAU | Rel.Area % | Resolution |
|---|---|---|---|---|---|---|---|---|
| 1 | n.a. | 18,817 | 0,0273 | n.a. | BM * | 0,179 | 0,03 | n.a. |
| 2 | n.a. | 18,983 | 0,0619 | n.a. | Mb* | 0,303 | 0,07 | n.a. |
| 3 | n.a. | 19,717 | 0,1422 | n.a. | bM * | 0,678 | 0,15 | n.a. |
| 4 | n.a. | 20,050 | 0,8912 | n.a. | M * | 5,190 | 0,95 | n.a. |
| 5 | n.a. | 20,217 | 1,5433 | n.a. | M * | 8,858 | 1,65 | n.a. |
| 6 | n.a. | 20,400 | 1,7751 | n.a. | M * | 5,760 | 1,89 | n.a. |
| 7 | n.a. | 21,033 | 3,7116 | n.a. | M * | 13,415 | 3,96 | n.a. |
| 8 | n.a. | 21,383 | 1,4016 | n.a. | M * | 8,510 | 1,49 | n.a. |
| 9 | n.a. | 21,617 | 1,3232 | n.a. | M * | 9,249 | 1,41 | n.a. |
| *10* | *Main peak* | *21,783* | *42,4517* | *n.a.* | *M ** | *199,295* | *45,27* | *n.a.* |
| 11 | n.a. | 22,150 | 10,2395 | n.a. | M * | 41,024 | 10,92 | n.a. |
| 12 | n.a. | 22,383 | 5,8430 | n.a. | M * | 21,217 | 6,23 | n.a. |
| 13 | n.a. | 22,867 | 5,6574 | n.a. | M * | 16,566 | 6,03 | n.a. |
| 14 | n.a. | 23,350 | 5,6743 | n.a. | M * | 17,570 | 6,05 | n.a. |
| 15 | n.a. | 23,617 | 6,5273 | n.a. | M * | 15,696 | 6,96 | n.a. |
| 16 | n.a. | 24,000 | 2,3368 | n.a. | M * | 6,614 | 2,49 | n.a. |
| 17 | n.a. | 24,633 | 1,7098 | n.a. | M * | 5,230 | 1,82 | n.a. |
| 18 | n.a. | 24,917 | 0,7684 | n.a. | M * | 1,965 | 0,82 | n.a. |
| 19 | n.a. | 25,483 | 0,7670 | n.a. | M * | 2,042 | 0,82 | n.a. |
| 20 | n.a. | 26,000 | 0,2687 | n.a. | M * | 1,362 | 0,29 | n.a. |
| 21 | n.a. | 26,283 | 0,3241 | n.a. | M * | 0,986 | 0,35 | n.a. |
| 22 | n.a. | 26,717 | 0,2698 | n.a. | M * | 1,364 | 0,29 | n.a. |
| 23 | n.a. | 27,300 | 0,0495 | n.a. | MB* | 0,027 | 0,05 | n.a. |
| **Total:** | | | **93,7645** | **0,0000** | | **383,100** | **100,00** | |

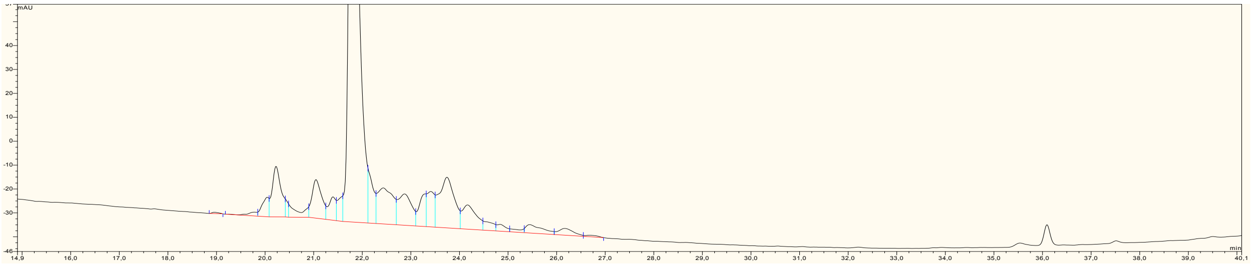

**Figure S11.** HPLC chromatogram of exenatide crude prepared using TPMT as thiol scavenger in TFA cleavage of exenatide peptide resin.

**Table S11.** Area% for integrated peaks.

| No. | Peakname | Ret.Time min | Area mAU*min | Amount | Type | Height mAU | Rel.Area % | Resolution |
|---|---|---|---|---|---|---|---|---|
| 1 | n.a. | 18,967 | 0,0872 | n.a. | BMB* | 0,551 | 0,09 | n.a. |
| 2 | n.a. | 19,850 | 0,3677 | n.a. | BM * | 1,691 | 0,36 | n.a. |
| 3 | n.a. | 20,050 | 1,2922 | n.a. | M * | 8,167 | 1,27 | n.a. |
| 4 | n.a. | 20,217 | 4,4905 | n.a. | M * | 21,067 | 4,42 | n.a. |
| 5 | n.a. | 20,433 | 0,4390 | n.a. | M * | 7,097 | 0,43 | n.a. |
| 6 | n.a. | 20,500 | 1,2932 | n.a. | M * | 5,021 | 1,27 | n.a. |
| 7 | n.a. | 21,050 | 3,7084 | n.a. | M * | 16,144 | 3,65 | n.a. |
| 8 | n.a. | 21,400 | 1,6793 | n.a. | M * | 9,797 | 1,65 | n.a. |
| 9 | n.a. | 21,600 | 1,2607 | n.a. | M * | 10,718 | 1,24 | n.a. |
| *10* | *Main peak* | *21,800* | *53,8602* | *n.a.* | *M ** | *244,781* | *52,97* | *n.a.* |
| 11 | n.a. | 22,133 | 2,8105 | n.a. | M * | 21,614 | 2,76 | n.a. |
| 12 | n.a. | 22,433 | 5,5421 | n.a. | M * | 15,082 | 5,45 | n.a. |
| 13 | n.a. | 22,867 | 4,2397 | n.a. | M * | 13,103 | 4,17 | n.a. |
| 14 | n.a. | 23,317 | 2,2630 | n.a. | M * | 13,673 | 2,23 | n.a. |
| 15 | n.a. | 23,417 | 2,6082 | n.a. | M * | 14,814 | 2,56 | n.a. |
| 16 | n.a. | 23,750 | 7,7977 | n.a. | M * | 21,179 | 7,67 | n.a. |
| 17 | n.a. | 24,167 | 3,4634 | n.a. | M * | 10,152 | 3,41 | n.a. |
| 18 | n.a. | 24,500 | 0,8834 | n.a. | M * | 3,695 | 0,87 | n.a. |
| 19 | n.a. | 24,833 | 0,6234 | n.a. | M * | 2,840 | 0,61 | n.a. |
| 20 | n.a. | 25,333 | 0,3488 | n.a. | M * | 1,728 | 0,34 | n.a. |
| 21 | n.a. | 25,450 | 1,4234 | n.a. | M * | 3,506 | 1,40 | 1,07 |
| 22 | n.a. | 26,167 | 1,0211 | n.a. | M * | 2,829 | 1,00 | n.a. |
| 23 | n.a. | 26,700 | 0,1859 | n.a. | MB* | 0,650 | 0,18 | n.a. |
| **Total:** | | | **101,6891** | **0,0000** | | **449,899** | **100,00** | |

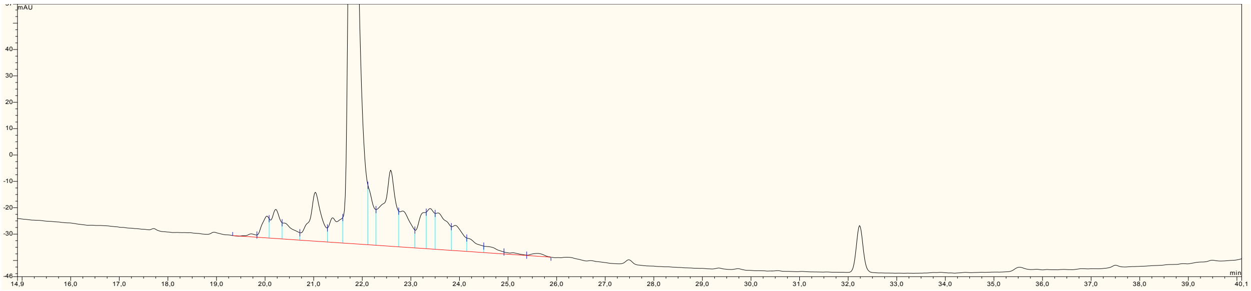

**Figure S12.** HPLC chromatogram of exenatide crude prepared using 2,4-DMOT as thiol scavenger in TFA cleavage of exenatide peptide resin.

**Table S12.** Area% for integrated peaks.

| No. | Peakname | Ret.Time min | Area mAU*min | Amount | Type | Height mAU | Rel.Area % | Resolution |
|---|---|---|---|---|---|---|---|---|
| 1 | n.a. | 19,717 | 0,2136 | n.a. | BM * | 1,134 | 0,22 | n.a. |
| 2 | n.a. | 20,033 | 1,3146 | n.a. | M * | 8,267 | 1,36 | n.a. |
| 3 | n.a. | 20,217 | 2,2768 | n.a. | M * | 11,048 | 2,36 | n.a. |
| 4 | n.a. | 20,400 | 1,6459 | n.a. | M * | 6,091 | 1,70 | n.a. |
| 5 | n.a. | 21,033 | 5,2255 | n.a. | M * | 18,559 | 5,41 | n.a. |
| 6 | n.a. | 21,600 | 2,6073 | n.a. | M * | 9,694 | 2,70 | n.a. |
| 7 | Main peak | 21,783 | 53,7868 | n.a. | M * | 244,790 | 55,67 | n.a. |
| 8 | n.a. | 22,133 | 2,8653 | n.a. | M * | 21,491 | 2,97 | n.a. |
| 9 | n.a. | 22,583 | 8,7127 | n.a. | M * | 28,847 | 9,02 | n.a. |
| 10 | n.a. | 22,833 | 3,7214 | n.a. | M * | 13,679 | 3,85 | n.a. |
| 11 | n.a. | 23,317 | 2,5889 | n.a. | M * | 13,828 | 2,68 | n.a. |
| 12 | n.a. | 23,400 | 2,6674 | n.a. | M * | 15,305 | 2,76 | n.a. |
| 13 | n.a. | 23,567 | 3,9540 | n.a. | M * | 13,854 | 4,09 | n.a. |
| 14 | n.a. | 23,917 | 2,4872 | n.a. | M * | 9,594 | 2,57 | n.a. |
| 15 | n.a. | 24,167 | 1,2792 | n.a. | M * | 5,009 | 1,32 | n.a. |
| 16 | n.a. | 24,517 | 0,7518 | n.a. | M * | 2,411 | 0,78 | n.a. |
| 17 | n.a. | 25,117 | 0,2156 | n.a. | M * | 0,681 | 0,22 | n.a. |
| 18 | n.a. | 25,633 | 0,3018 | n.a. | MB* | 1,064 | 0,31 | n.a. |
| **Total:** | | | **96,6159** | **0,0000** | | **425,346** | **100,00** | |

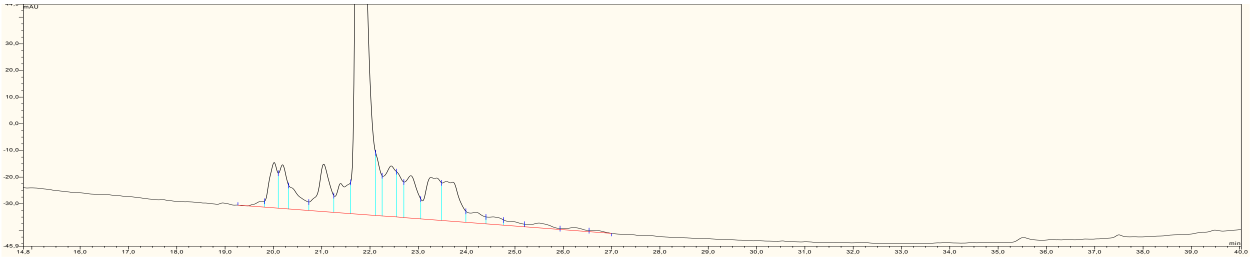

**Figure S13.** HPLC chromatogram of exenatide crude prepared without thiol scavenger in TFA cleavage of exenatide peptide resin.

**Table S13.** Area% for integrated peaks.

| No. | Peakname | Ret.Time min | Area mAU*min | Amount | Type | Height mAU | Rel.Area % | Resolution |
|---|---|---|---|---|---|---|---|---|
| 1 | n.a. | 19,817 | 0,4051 | n.a. | BM * | 2,191 | 0,39 | n.a. |
| 2 | n.a. | 20,017 | 3,2333 | n.a. | M * | 17,052 | 3,09 | n.a. |
| 3 | n.a. | 20,183 | 2,9534 | n.a. | M * | 16,385 | 2,82 | n.a. |
| 4 | n.a. | 20,333 | 2,3212 | n.a. | M * | 8,383 | 2,22 | n.a. |
| 5 | n.a. | 21,033 | 4,9414 | n.a. | M * | 17,793 | 4,73 | n.a. |
| 6 | n.a. | 21,600 | 3,3456 | n.a. | M * | 11,762 | 3,20 | n.a. |
| 7 | Main peak | 21,783 | 56,8519 | n.a. | M * | 256,434 | 54,36 | n.a. |
| 8 | n.a. | 22,133 | 2,5313 | n.a. | M * | 22,512 | 2,42 | n.a. |
| 9 | n.a. | 22,433 | 5,1359 | n.a. | M * | 18,974 | 4,91 | n.a. |
| 10 | n.a. | 22,567 | 2,2638 | n.a. | M * | 16,698 | 2,16 | n.a. |
| 11 | n.a. | 22,850 | 4,5512 | n.a. | M * | 15,927 | 4,35 | n.a. |
| 12 | n.a. | 23,267 | 5,7319 | n.a. | M * | 15,888 | 5,48 | n.a. |
| 13 | n.a. | 23,583 | 5,8012 | n.a. | M * | 14,760 | 5,55 | n.a. |
| 14 | n.a. | 24,200 | 1,4736 | n.a. | M * | 4,029 | 1,41 | n.a. |
| 15 | n.a. | 24,567 | 0,9499 | n.a. | M * | 2,807 | 0,91 | n.a. |
| 16 | n.a. | 24,783 | 0,6314 | n.a. | M * | 1,803 | 0,60 | n.a. |
| 17 | n.a. | 25,500 | 0,8466 | n.a. | M * | 1,805 | 0,81 | n.a. |
| 18 | n.a. | 26,267 | 0,4195 | n.a. | M * | 1,083 | 0,40 | n.a. |
| 19 | n.a. | 26,717 | 0,1879 | n.a. | MB* | 0,666 | 0,18 | n.a. |
| **Total:** | | | **104,5761** | **0,0000** | | **446,952** | **100,00** | |

## 5. LC-HRMS analyses of exenatide crudes

Experimental conditions: column: Waters peptide CSH C18, 2.1x150mm, 1.7um, 130Å; column temperature: 55°C; injection volume: varying (~1 µL), adjusted to provide exenatide main peak of comparable size in all crudes; sampler temperature: 10°C; MS mode: positive 50-3200; DAD: 220 nm; data rate: 5Hz; detector cell: standard cell 1uL; flow: 0.2 ml/min; jet weaver: v380 mixer; mobile phase A: 0.1 % TFA in water, mobile phase B: 0.10 % TFA in MeCN. Gradient (Time(min), %B): 0, 11; 1, 11; 5, 33; 50, 37; 54, 60; 56, 90; 58, 90; 58.1, 11; 70, 11. For runs in which appreciable peaks were detected which were not observed with the other scavengers (DODT, 4-MOBM and 2,4-DMOT) MS identities of such peaks are provided. For all runs EIC MS analyses were carried out for the specific cleavage related impurities investigated herein, see section 6 of this Electronic Supporting Information.

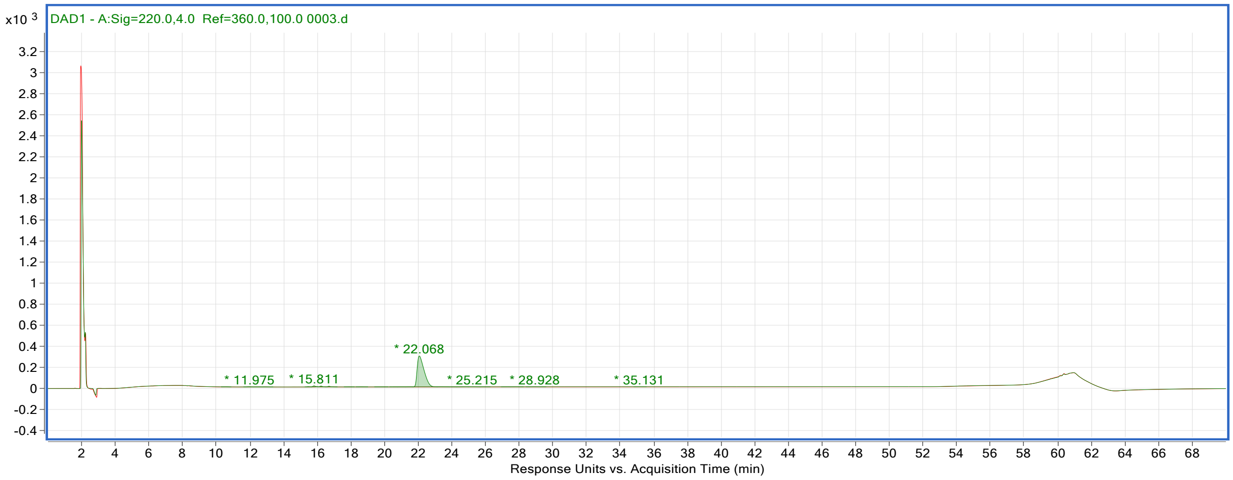


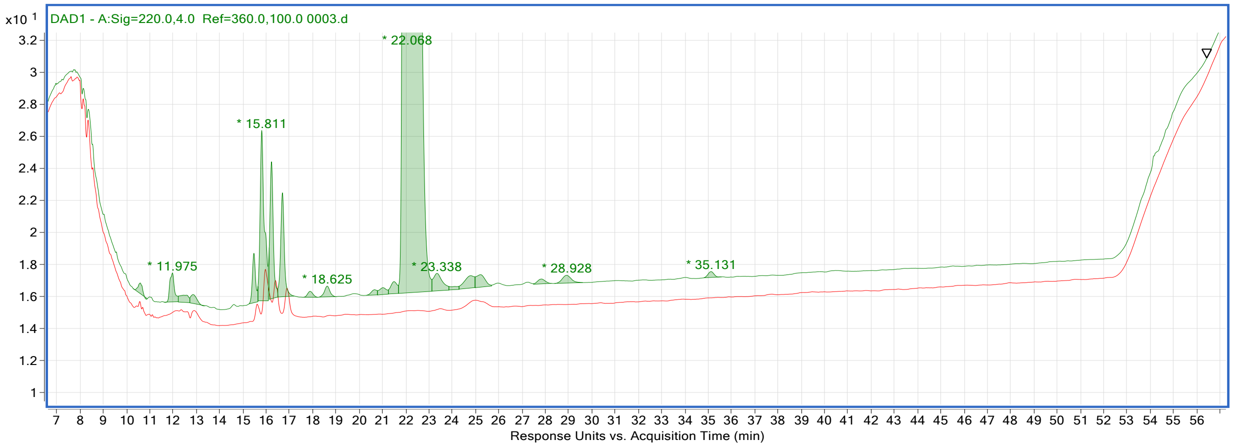


**Figure S14**. UV chromatogram from LC-HRMS analysis of exenatide API reference. The peaks at 15 – 17 min are artefacts present in all chromatograms as well as in the blank (in red) and were not integrated.

**Table S14.** Area% for integrated peaks.

| Peak | Rt | Area | RRT | Area % |
|---|---|---|---|---|
| 1 | 10,59 | 8,090 | 0,480 | 0,09 |
| 2 | 11,98 | 25,350 | 0,543 | 0,30 |
| 3 | 12,45 | 12,250 | 0,564 | 0,14 |
| 4 | 12,86 | 9,730 | 0,583 | 0,11 |
| 5 | 17,89 | 6,020 | 0,810 | 0,07 |
| 6 | 18,63 | 11,050 | 0,844 | 0,13 |
| 7 | 20,67 | 5,490 | 0,937 | 0,06 |
| 8 | 21,02 | 9,630 | 0,952 | 0,11 |
| 9 | 21,50 | 14,900 | 0,974 | 0,18 |
| 10 | 22,07 | 8304,620 | 1,000 | 97,55 |
| 11 | 23,34 | 30,110 | 1,058 | 0,35 |
| 12 | 24,29 | 5,160 | 1,101 | 0,06 |
| 13 | 24,81 | 22,890 | 1,124 | 0,27 |
| 14 | 25,22 | 21,220 | 1,143 | 0,25 |
| 15 | 27,84 | 6,970 | 1,261 | 0,08 |
| 16 | 28,928 | 13,2 | 1,311 | 0,16 |
| 17 | 35,131 | 6,56 | 1,592 | 0,08 |

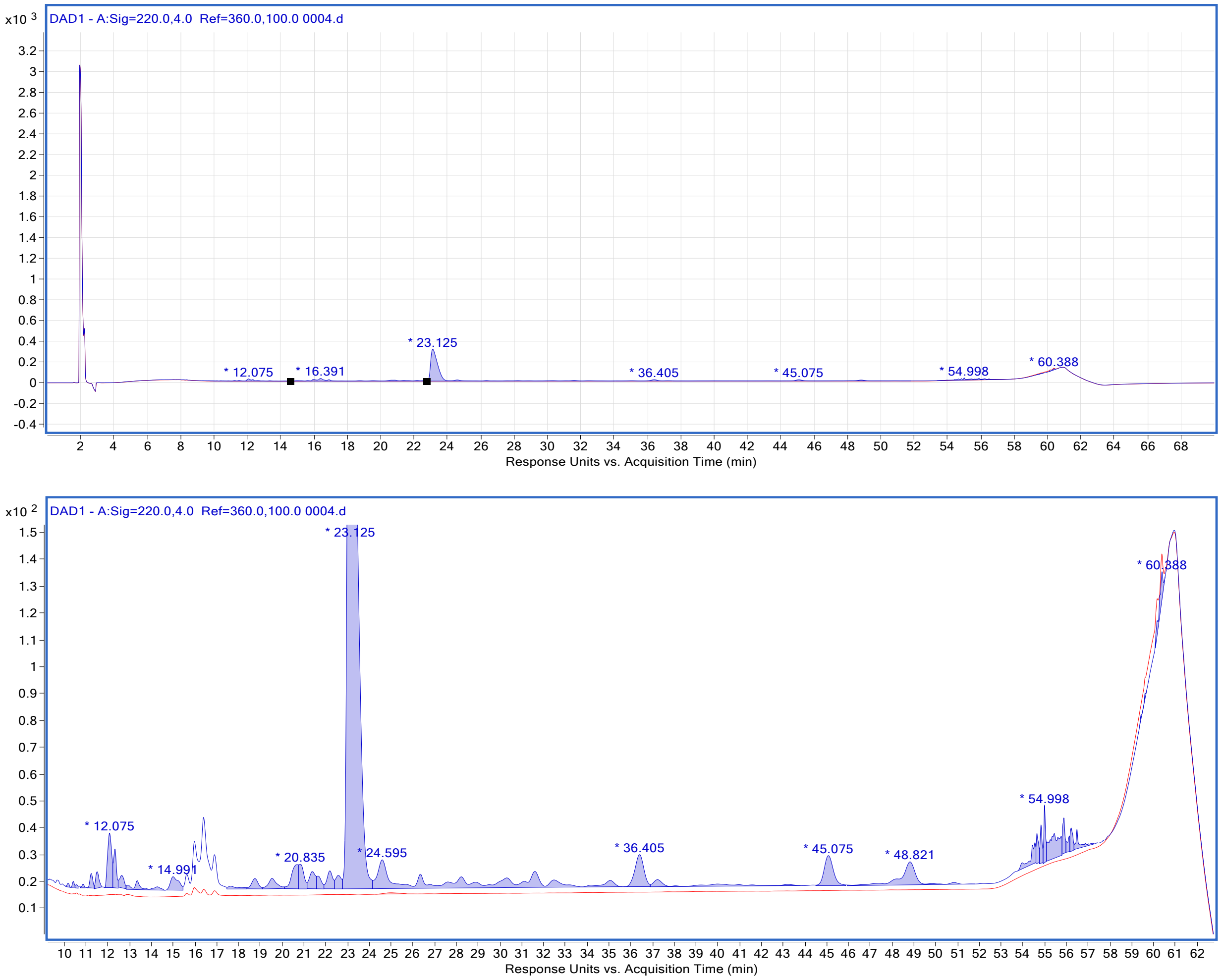


**Figure S15**. UV chromatogram from LC-HRMS analysis of exenatide crude prepared using DTT as thiol scavenger in TFA cleavage of exenatide peptide resin.

**Table S15.** Area% for integrated peaks.

| Peak | Rt | Area | RRT | Area % |
|---|---|---|---|---|
| 1 | 10,17 | 9,45 | 0,440 | 0,07 |
| 2 | 10,41 | 13,54 | 0,450 | 0,09 |
| 3 | 10,55 | 9,12 | 0,456 | 0,06 |
| 4 | 10,87 | 9,96 | 0,470 | 0,07 |
| 5 | 11,24 | 48,45 | 0,486 | 0,33 |
| 6 | 11,51 | 66,50 | 0,498 | 0,46 |
| 7 | 12,08 | 264,61 | 0,522 | 1,83 |
| 8 | 12,33 | 137,19 | 0,533 | 0,95 |
| 9 | 12,63 | 70,15 | 0,546 | 0,48 |
| 10 | 13,35 | 98,15 | 0,577 | 0,68 |
| 11 | 14,99 | 133,62 | 0,648 | 0,92 |
| 12 | 17,63 | 36,23 | 0,762 | 0,25 |
| 13 | 18,75 | 80,28 | 0,811 | 0,55 |
| 14 | 19,54 | 105,35 | 0,845 | 0,73 |
| 15 | 20,68 | 184,62 | 0,894 | 1,28 |
| 16 | 20,84 | 156,04 | 0,901 | 1,08 |
| 17 | 21,39 | 128,66 | 0,925 | 0,89 |
| 18 | 21,65 | 69,40 | 0,936 | 0,48 |
| 19 | 22,19 | 122,22 | 0,959 | 0,84 |
| 20 | 22,58 | 95,19 | 0,976 | 0,66 |
| 21 | 23,13 | 8810,55 | 1,000 | 60,89 |
| 22 | 24,60 | 1369,00 | 1,064 | 9,46 |
| 23 | 36,41 | 342,13 | 1,574 | 2,36 |
| 24 | 37,23 | 196,57 | 1,610 | 1,36 |
| 25 | 45,08 | 325,47 | 1,949 | 2,25 |
| 26 | 48,82 | 419,75 | 2,111 | 2,90 |
| 27 | 54,56 | 132,12 | 2,359 | 0,91 |
| 28 | 54,65 | 78,20 | 2,363 | 0,54 |
| 29 | 54,84 | 91,34 | 2,371 | 0,63 |
| 30 | 55,00 | 115,62 | 2,378 | 0,80 |
| 31 | 55,45 | 311,44 | 2,398 | 2,15 |
| 32 | 55,89 | 98,06 | 2,417 | 0,68 |
| 33 | 56,13 | 47,75 | 2,427 | 0,33 |
| 34 | 56,22 | 71,96 | 2,431 | 0,50 |
| 35 | 56,49 | 75,60 | 2,443 | 0,52 |
| 36 | 59,70 | 13,33 | 2,581 | 0,09 |
| 37 | 60,39 | 132,05 | 2,611 | 0,91 |

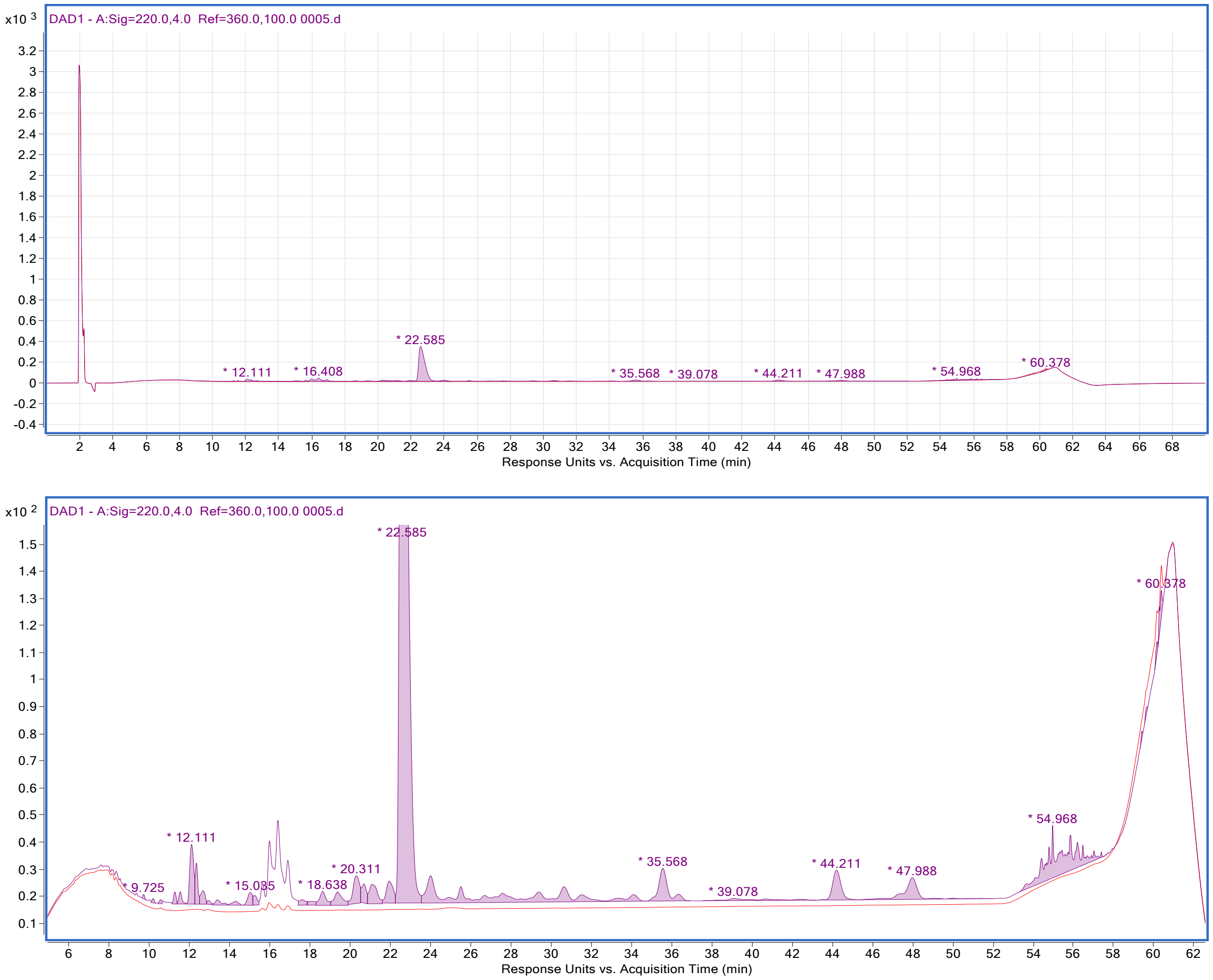


**Figure S16**. UV chromatogram from LC-HRMS analysis of exenatide crude prepared using EDT as thiol scavenger in TFA cleavage of exenatide peptide resin.

**Table S16.** Area% for integrated peaks.

| Peak | Rt | Area | RRT | Area % |
|---|---|---|---|---|
| 1 | 9,73 | 10,450 | 0,431 | 0,07 |
| 2 | 10,20 | 12,580 | 0,452 | 0,09 |
| 3 | 10,57 | 12,940 | 0,468 | 0,09 |
| 4 | 11,28 | 38,840 | 0,499 | 0,27 |
| 5 | 11,55 | 48,810 | 0,511 | 0,34 |
| 6 | 12,11 | 300,180 | 0,536 | 2,07 |
| 7 | 12,35 | 132,960 | 0,547 | 0,92 |
| 8 | 12,68 | 77,660 | 0,561 | 0,54 |
| 9 | 12,96 | 16,970 | 0,574 | 0,12 |
| 10 | 13,40 | 75,000 | 0,593 | 0,52 |
| 11 | 15,04 | 74,670 | 0,666 | 0,52 |
| 12 | 15,26 | 50,990 | 0,676 | 0,35 |
| 13 | 17,62 | 44,460 | 0,780 | 0,31 |
| 14 | 18,06 | 33,770 | 0,799 | 0,23 |
| 15 | 18,64 | 117,940 | 0,825 | 0,81 |
| 16 | 19,38 | 144,010 | 0,858 | 0,99 |
| 17 | 20,31 | 227,880 | 0,899 | 1,57 |
| 18 | 20,69 | 118,450 | 0,916 | 0,82 |
| 19 | 21,10 | 206,330 | 0,934 | 1,42 |
| 20 | 21,95 | 195,620 | 0,972 | 1,35 |
| 21 | 22,59 | 8765,400 | 1,000 | 60,50 |
| 22 | 35,57 | 1724,390 | 1,575 | 11,90 |
| 23 | 39,08 | 95,580 | 1,730 | 0,66 |
| 24 | 44,21 | 317,680 | 1,958 | 2,19 |
| 25 | 47,99 | 364,770 | 2,125 | 2,52 |
| 26 | 54,97 | 1120,790 | 2,434 | 7,74 |
| 27 | 59,41 | 11,830 | 2,630 | 0,08 |
| 28 | 59,71 | 15,500 | 2,644 | 0,11 |
| 29 | 60,16 | 30,880 | 2,664 | 0,21 |
| 30 | 60,38 | 101,050 | 2,673 | 0,70 |

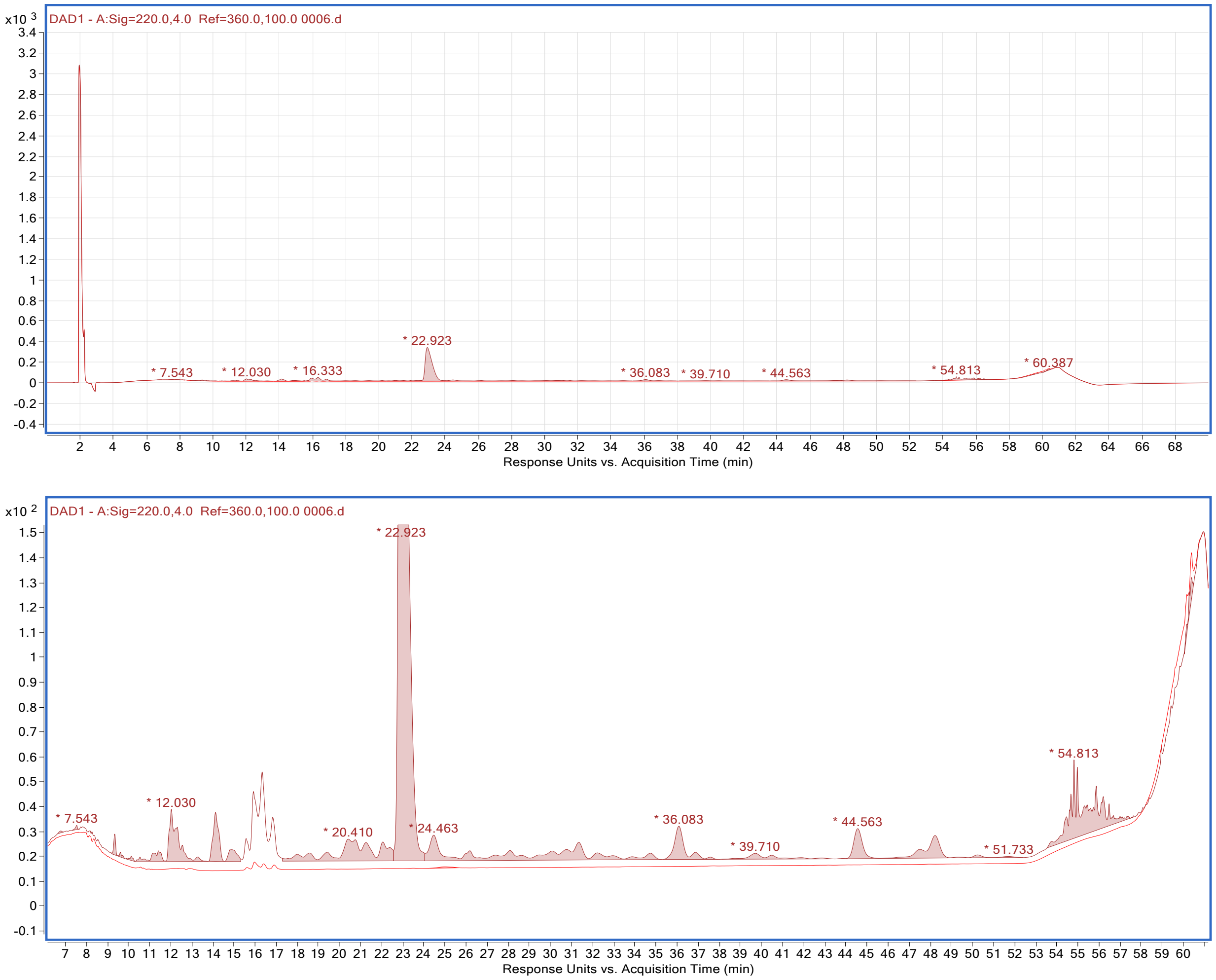


**Figure S17**. UV chromatogram from LC-HRMS analysis of exenatide crude prepared using DODT as thiol scavenger in TFA cleavage of exenatide peptide resin.

**Table S17.** Area% for integrated peaks.

| Peak | Rt | Area | RRT | Area % |
|---|---|---|---|---|
| 1 | 6,787 | 7,17 | 0,296 | 0,04 |
| 2 | 7,543 | 11,55 | 0,329 | 0,07 |
| 3 | 9,343 | 66,77 | 0,408 | 0,4 |
| 4 | 10,130 | 17,59 | 0,442 | 0,11 |
| 5 | 12,030 | 741,45 | 0,525 | 4,44 |
| 6 | 14,123 | 369,86 | 0,616 | 2,21 |
| 7 | 14,843 | 138,73 | 0,648 | 0,83 |
| 8 | 20,410 | 1149,27 | 0,890 | 6,88 |
| 9 | 22,923 | 9504,31 | 1,000 | 56,86 |
| 10 | 24,463 | 1535,17 | 1,067 | 9,18 |
| 11 | 36,083 | 468,29 | 1,574 | 2,8 |
| 12 | 39,710 | 186,18 | 1,732 | 1,11 |
| 13 | 44,563 | 826,38 | 1,944 | 4,94 |
| 14 | 51,733 | 17,00 | 2,257 | 0,1 |
| 15 | 54,813 | 1559,76 | 2,391 | 9,33 |
| 16 | 60,207 | 26,10 | 2,626 | 0,16 |
| 17 | 60,387 | 90,67 | 2,634 | 0,54 |

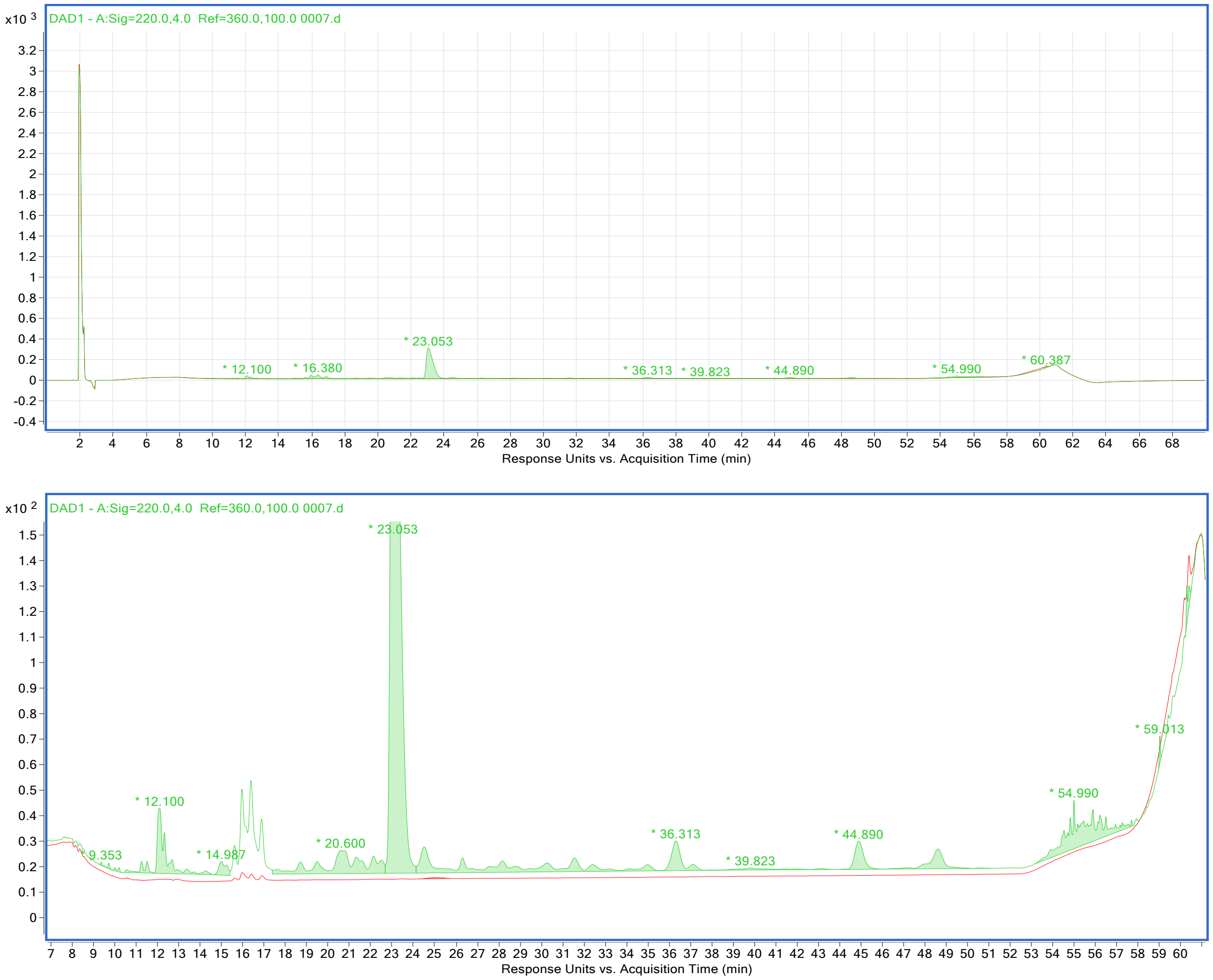


**Figure S18**. UV chromatogram from LC-HRMS analysis of exenatide crude prepared using 1,4-BDMT as thiol scavenger in TFA cleavage of exenatide peptide resin.

**Table S18.** Area% for integrated peaks.

| Peak | Rt | Area | RRT | Area % |
|---|---|---|---|---|
| 1 | 9,35 | 18,850 | 0,406 | 0,13 |
| 2 | 10,19 | 18,010 | 0,442 | 0,12 |
| 3 | 12,10 | 762,300 | 0,525 | 5,26 |
| 4 | 14,99 | 137,010 | 0,650 | 0,95 |
| 5 | 20,60 | 1095,710 | 0,894 | 7,56 |
| 6 | 23,05 | 8565,080 | 1,000 | 59,1 |
| 7 | 36,31 | 1751,960 | 1,575 | 12,09 |
| 8 | 39,82 | 102,250 | 1,727 | 0,71 |
| 9 | 44,89 | 672,290 | 1,947 | 4,64 |
| 10 | 54,99 | 1230,520 | 2,385 | 8,49 |
| 11 | 59,01 | 37,800 | 2,560 | 0,26 |
| 12 | 60,39 | 99,610 | 2,619 | 0,69 |

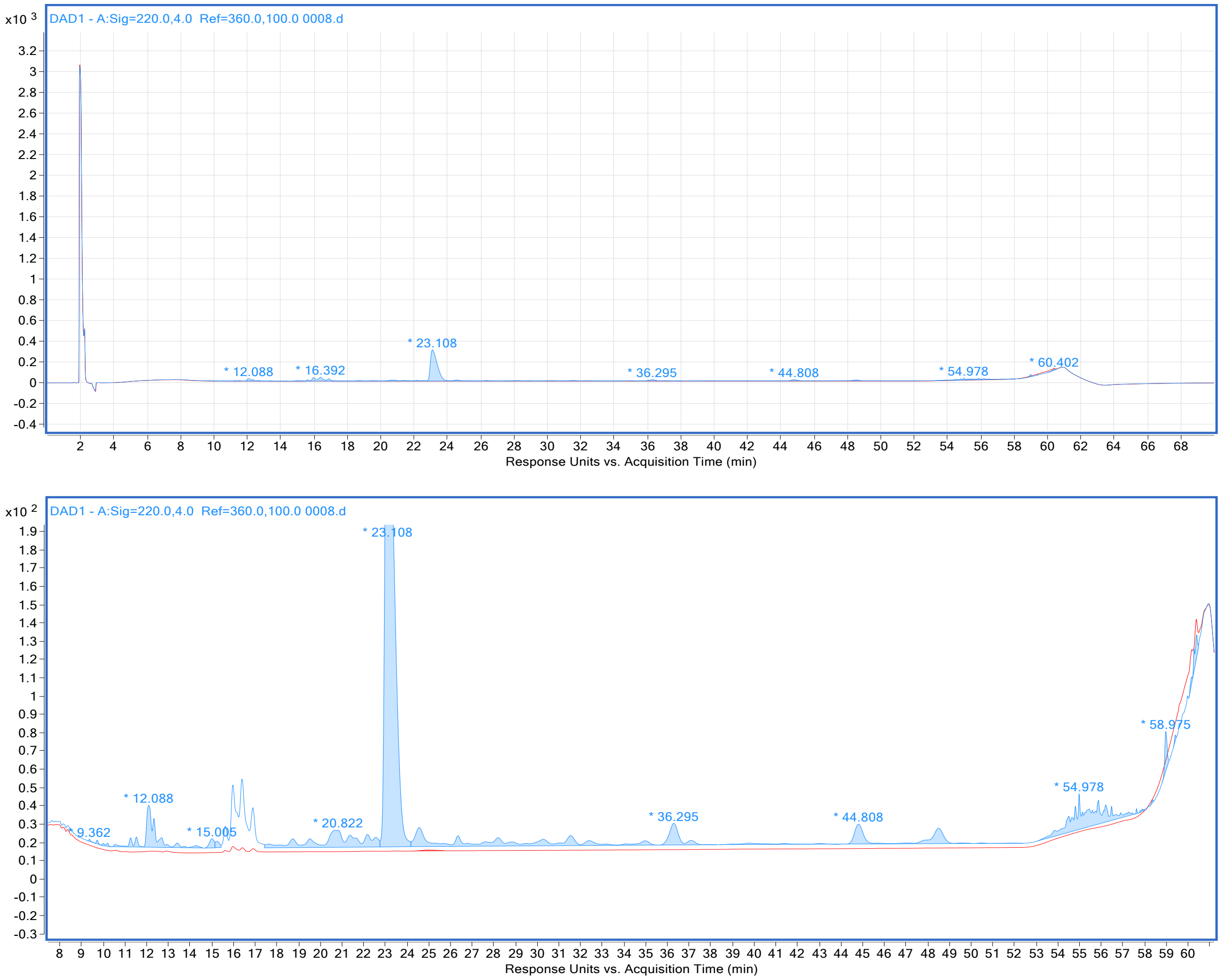


**Figure S19**. UV chromatogram from LC-HRMS analysis of exenatide crude prepared using 1,3-BDMT as thiol scavenger in TFA cleavage of exenatide peptide resin.

**Table S19.** Area% for integrated peaks.

| Peak | Rt | Area | RRT | Area % |
|---|---|---|---|---|
| 1 | 9,36 | 17,410 | 0,405 | 0,12 |
| 2 | 10,195 | 21,35 | 0,441 | 0,140 |
| 3 | 12,088 | 735,96 | 0,523 | 4,980 |
| 4 | 15,005 | 77,06 | 0,649 | 0,520 |
| 5 | 15,248 | 45,96 | 0,660 | 0,310 |
| 6 | 20,822 | 1152,99 | 0,901 | 7,800 |
| 7 | 23,108 | 8619,09 | 1,000 | 58,340 |
| 8 | 36,295 | 1759,73 | 1,571 | 11,910 |
| 9 | 44,808 | 761,85 | 1,939 | 5,160 |
| 10 | 54,978 | 1245,37 | 2,379 | 8,430 |
| 11 | 57,885 | 9,78 | 2,505 | 0,070 |
| 12 | 58,378 | 6,24 | 2,526 | 0,040 |
| 13 | 58,975 | 158,52 | 2,552 | 1,070 |
| 14 | 59,415 | 12,40 | 2,571 | 0,080 |
| 15 | 60,402 | 149,90 | 2,614 | 1,010 |

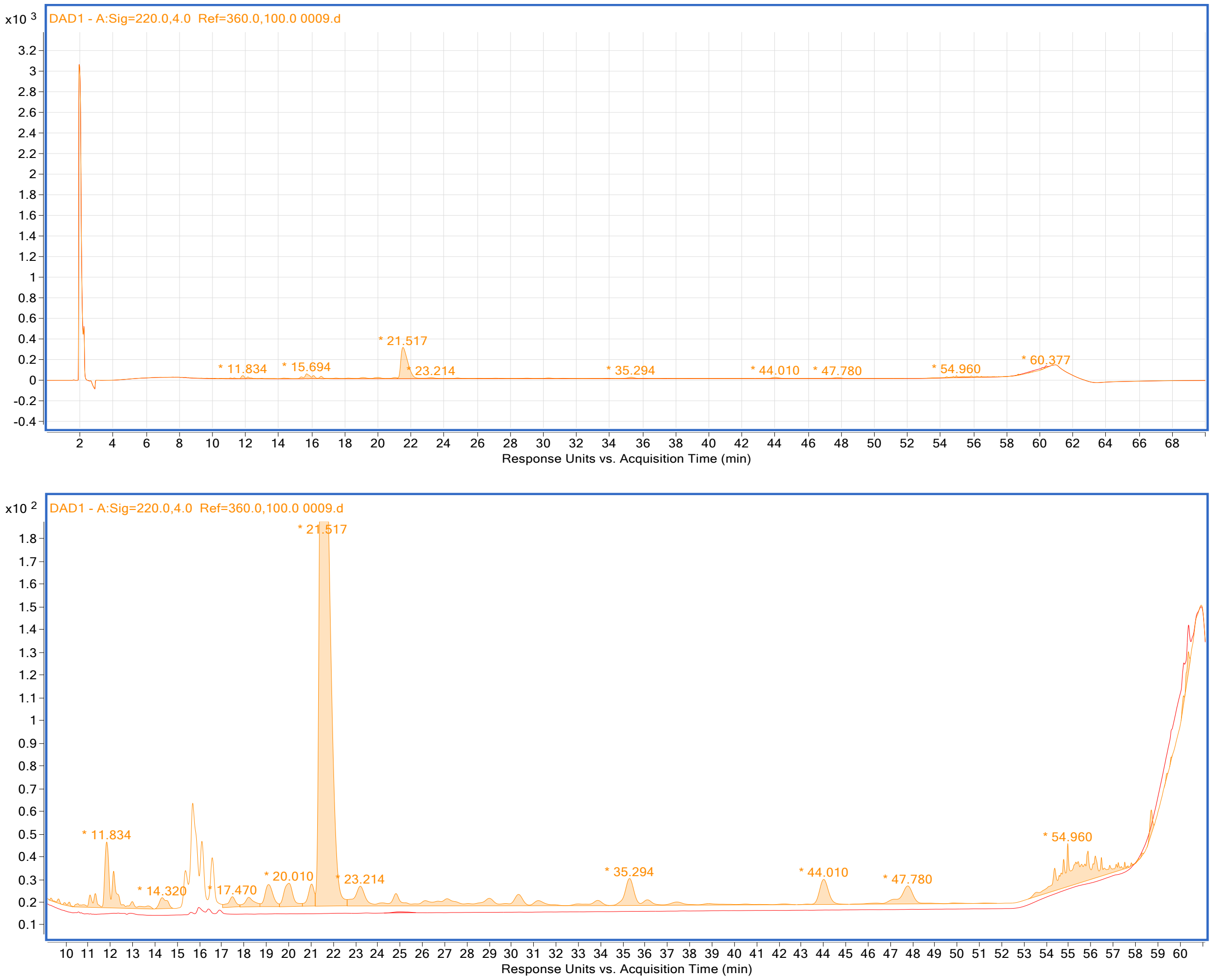


**Figure S20**. UV chromatogram from LC-HRMS analysis of exenatide crude prepared using 1,2-BDMT as thiol scavenger in TFA cleavage of exenatide peptide resin, UV chromatogram overview.

**Table S20.** Area% for integrated peaks.

| Peak | Rt | Area | RRT | Area % |
|---|---|---|---|---|
| 1 | 9,33 | 23,780 | 0,433 | 0,16 |
| 2 | 10,154 | 21,65 | 0,472 | 0,1500 |
| 3 | 11,834 | 824,38 | 0,550 | 5,6500 |
| 4 | 14,32 | 118,87 | 0,666 | 0,8100 |
| 5 | 17,47 | 107,51 | 0,812 | 0,7400 |
| 6 | 18,207 | 124,23 | 0,846 | 0,8500 |
| 7 | 19,097 | 251,91 | 0,888 | 1,7300 |
| 8 | 20,01 | 296,96 | 0,930 | 2,0400 |
| 9 | 21,027 | 180,25 | 0,977 | 1,2400 |
| 10 | 21,517 | 8730,14 | 1,000 | 59,8400 |
| 11 | 23,214 | 1213,52 | 1,079 | 8,3200 |
| 12 | 35,294 | 550,5 | 1,640 | 3,7700 |
| 13 | 44,01 | 317,3 | 2,045 | 2,1700 |
| 14 | 47,78 | 366,13 | 2,221 | 2,5100 |
| 15 | 54,96 | 1241,41 | 2,554 | 8,5100 |
| 16 | 57,814 | 5,03 | 2,687 | 0,0300 |
| 17 | 58,137 | 5,02 | 2,702 | 0,0300 |
| 18 | 58,387 | 5,77 | 2,714 | 0,0400 |
| 19 | 58,71 | 85,89 | 2,729 | 0,5900 |
| 20 | 59,45 | 7,3 | 2,763 | 0,0500 |
| 21 | 59,637 | 5,72 | 2,772 | 0,0400 |
| 22 | 60,377 | 105,92 | 2,806 | 0,7300 |

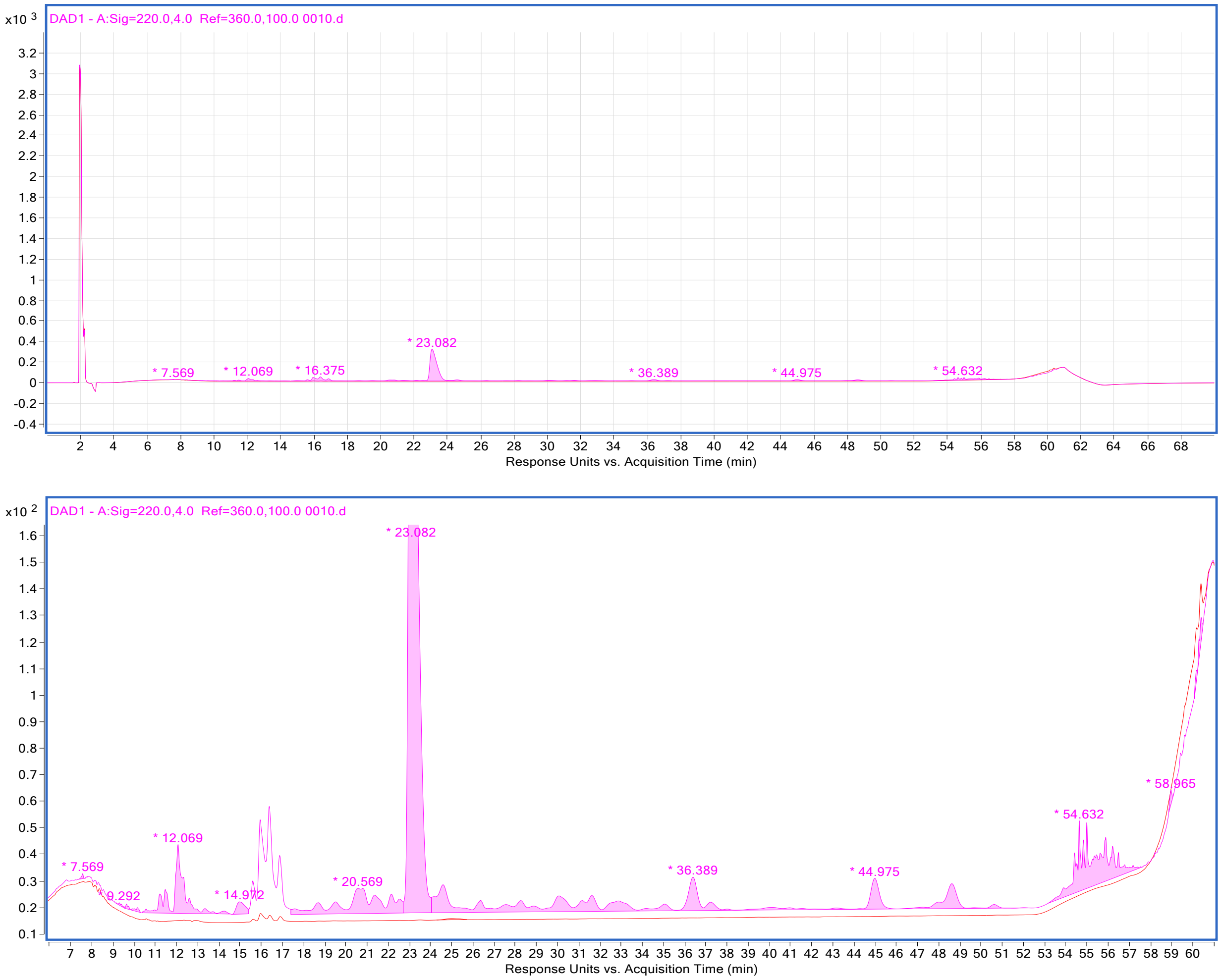


**Figure S21**. UV chromatogram from LC-HRMS analysis of exenatide crude prepared using 4,4'-BMMB as thiol scavenger in TFA cleavage of exenatide peptide resin, UV chromatogram overview.

**Table S21.** Area% for integrated peaks.

| Peak | Rt | Area | RRT | Area % |
|---|---|---|---|---|
| 1 | 7,57 | 10,490 | 0,328 | 0,06 |
| 2 | 9,29 | 10,800 | 0,403 | 0,07 |
| 3 | 9,64 | 33,810 | 0,417 | 0,21 |
| 4 | 12,07 | 913,590 | 0,523 | 5,63 |
| 5 | 14,97 | 132,900 | 0,649 | 0,82 |
| 6 | 20,57 | 1236,150 | 0,891 | 7,61 |
| 7 | 23,08 | 8932,960 | 1,000 | 55,02 |
| 8 | 36,39 | 2288,100 | 1,577 | 14,09 |
| 9 | 44,98 | 908,420 | 1,948 | 5,59 |
| 10 | 54,63 | 1619,840 | 2,367 | 9,98 |
| 11 | 58,97 | 36,570 | 2,555 | 0,23 |
| 12 | 60,39 | 113,210 | 2,616 | 0,7 |

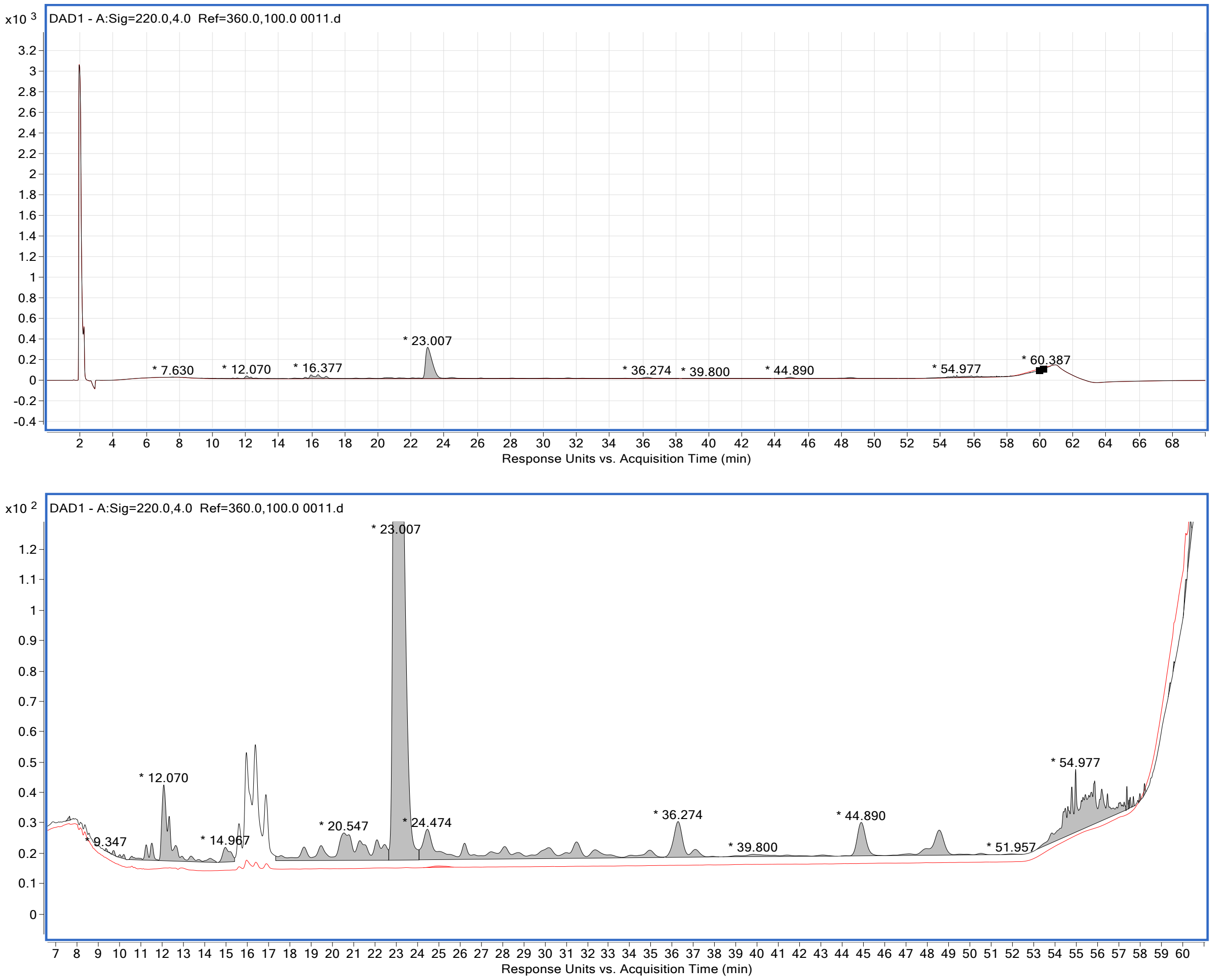


**Figure S22**. UV chromatogram from LC-HRMS analysis of exenatide crude prepared using 2,4-DCBM as thiol scavenger in TFA cleavage of exenatide peptide resin, UV chromatogram overview.

**Table S22.** Area% for integrated peaks.

| Peak | Rt | Area | RRT | Area % |
|---|---|---|---|---|
| 1 | 7,63 | 8,340 | 0,332 | 0,06 |
| 2 | 9,347 | 15,99 | 0,406 | 0,11 |
| 3 | 10,184 | 16,09 | 0,443 | 0,11 |
| 4 | 12,07 | 769,64 | 0,525 | 5,18 |
| 5 | 14,967 | 124,23 | 0,651 | 0,84 |
| 6 | 20,547 | 1092,28 | 0,893 | 7,36 |
| 7 | 23,007 | 8619,92 | 1,000 | 58,04 |
| 8 | 24,474 | 1421,43 | 1,064 | 9,57 |
| 9 | 36,274 | 409,17 | 1,577 | 2,76 |
| 10 | 39,8 | 100,92 | 1,730 | 0,68 |
| 11 | 44,89 | 676,04 | 1,951 | 4,55 |
| 12 | 51,957 | 5,38 | 2,258 | 0,04 |
| 13 | 54,977 | 1414,35 | 2,390 | 9,52 |
| 14 | 57,39 | 26,45 | 2,494 | 0,18 |
| 15 | 57,487 | 8,9 | 2,499 | 0,06 |
| 16 | 57,547 | 12,56 | 2,501 | 0,08 |
| 17 | 57,697 | 9,67 | 2,508 | 0,07 |
| 18 | 58,004 | 9,65 | 2,521 | 0,06 |
| 19 | 58,22 | 13,82 | 2,531 | 0,09 |
| 20 | 59,467 | 7,71 | 2,585 | 0,05 |
| 21 | 59,607 | 5,72 | 2,591 | 0,04 |
| 22 | 60,217 | 21,35 | 2,617 | 0,14 |
| 23 | 60,387 | 61,04 | 2,625 | 0,41 |

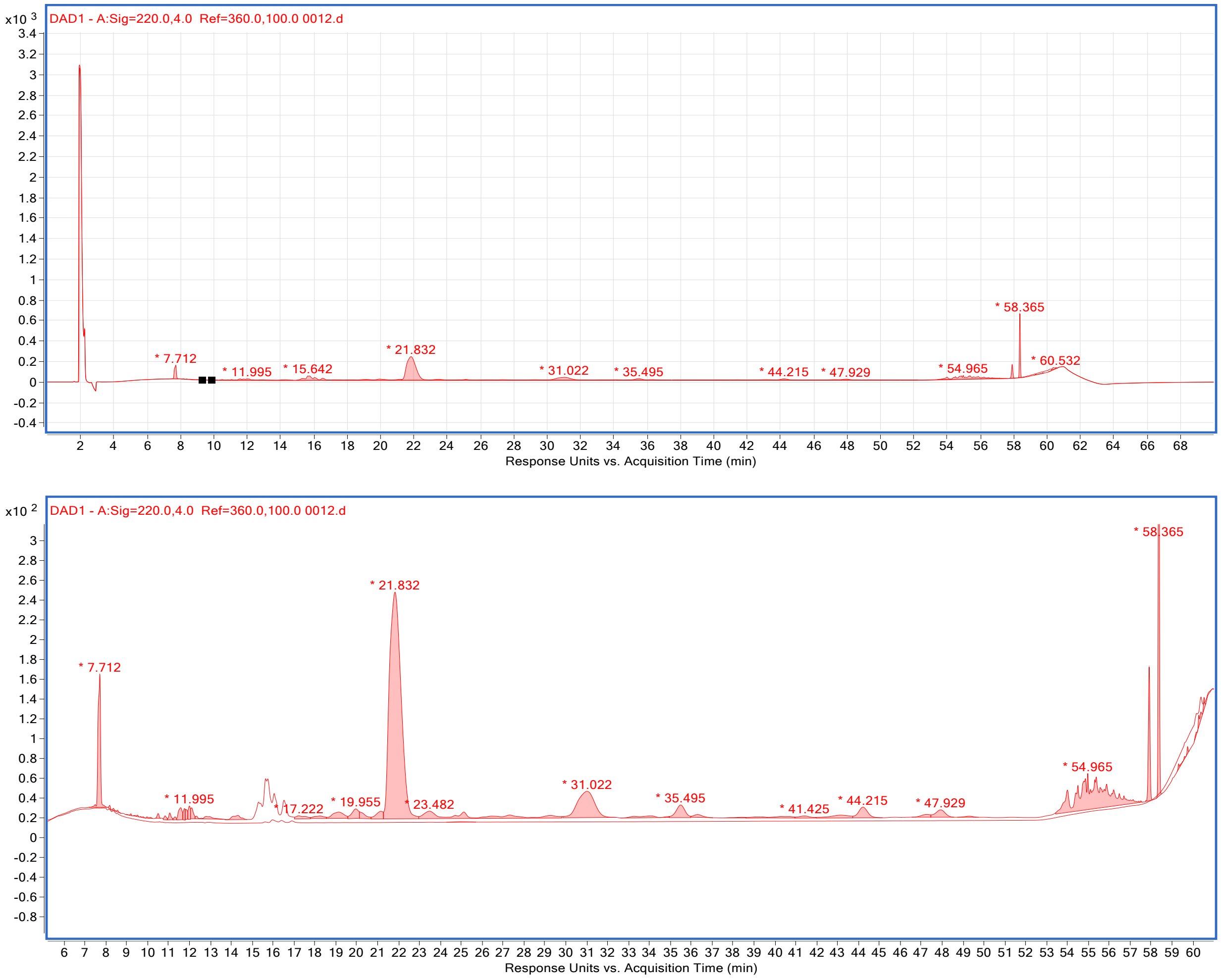


**Figure S23**. UV chromatogram from LC-HRMS analysis of exenatide crude prepared using 4-MOBM as thiol scavenger in TFA cleavage of exenatide peptide resin, UV chromatogram overview.

**Table S23.** Area% for integrated peaks.

| Peak | Rt | Area | RRT | Area % |
|---|---|---|---|---|
| 1 | 7,71 | 1143,480 | 0,353 | 5,08 |
| 2 | 8,179 | 16,98 | 0,375 | 0,08 |
| 3 | 8,355 | 22,39 | 0,383 | 0,1 |
| 4 | 9,195 | 9,73 | 0,421 | 0,04 |
| 5 | 9,515 | 18,51 | 0,436 | 0,08 |
| 6 | 9,965 | 15,03 | 0,456 | 0,07 |
| 7 | 10,502 | 30,97 | 0,481 | 0,14 |
| 8 | 10,835 | 31,81 | 0,496 | 0,14 |
| 9 | 11,059 | 55,15 | 0,507 | 0,24 |
| 10 | 11,309 | 21,57 | 0,518 | 0,1 |
| 11 | 11,579 | 151,9 | 0,530 | 0,67 |
| 12 | 11,762 | 70,6 | 0,539 | 0,31 |
| 13 | 11,795 | 69,85 | 0,540 | 0,31 |
| 14 | 11,995 | 91,09 | 0,549 | 0,4 |
| 15 | 12,105 | 97,33 | 0,554 | 0,43 |
| 16 | 12,335 | 99,29 | 0,565 | 0,44 |
| 17 | 14,315 | 120,25 | 0,656 | 0,53 |
| 18 | 17,222 | 105,75 | 0,789 | 0,47 |
| 19 | 18,245 | 89,46 | 0,836 | 0,4 |
| 20 | 19,139 | 248,06 | 0,877 | 1,1 |
| 21 | 19,955 | 218,93 | 0,914 | 0,97 |
| 22 | 20,129 | 127,12 | 0,922 | 0,56 |
| 23 | 21,162 | 184,07 | 0,969 | 0,82 |
| 24 | 21,832 | 8891,5 | 1,000 | 39,47 |
| 25 | 23,482 | 952,57 | 1,076 | 4,23 |
| 26 | 31,022 | 1522,11 | 1,421 | 6,76 |
| 27 | 34,059 | 136,05 | 1,560 | 0,6 |
| 28 | 35,495 | 385,47 | 1,626 | 1,71 |
| 29 | 36,309 | 84,72 | 1,663 | 0,38 |
| 30 | 38,422 | 25,04 | 1,760 | 0,11 |
| 31 | 39,175 | 60,55 | 1,794 | 0,27 |
| 32 | 40,299 | 99,32 | 1,846 | 0,44 |
| 33 | 41,425 | 87,63 | 1,897 | 0,39 |
| 34 | 43,132 | 199,46 | 1,976 | 0,89 |
| 35 | 44,215 | 336,47 | 2,025 | 1,49 |
| 36 | 47,255 | 83,79 | 2,164 | 0,37 |
| 37 | 47,929 | 245,79 | 2,195 | 1,09 |
| 38 | 49,309 | 31,31 | 2,259 | 0,14 |
| 39 | 54,965 | 3054 | 2,518 | 13,56 |
| 40 | 57,905 | 748,84 | 2,652 | 3,32 |
| 41 | 58,365 | 2331,21 | 2,673 | 10,35 |
| 42 | 59,742 | 60,36 | 2,736 | 0,27 |
| 43 | 60,532 | 150,78 | 2,773 | 0,67 |

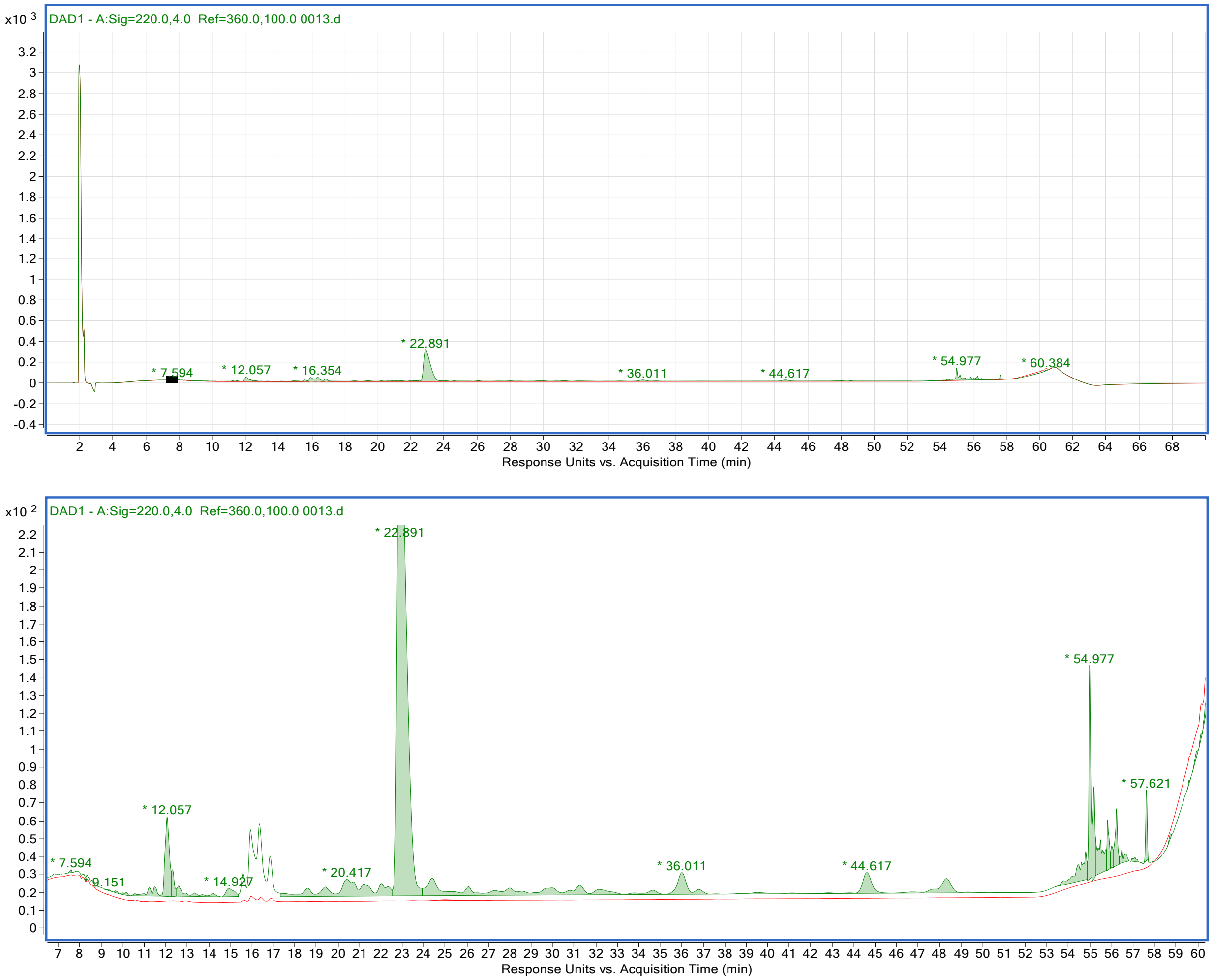


**Figure S24**. UV chromatogram from LC-HRMS analysis of exenatide crude prepared using TPMT as thiol scavenger in TFA cleavage of exenatide peptide resin, UV chromatogram overview.

**Table S24.** Area% for integrated peaks.

| Peak | Rt | Area | RRT | Area % |
|---|---|---|---|---|
| 1 | 7,59 | 9,080 | 0,332 | 0,05 |
| 2 | 9,151 | 16,74 | 0,400 | 0,1 |
| 3 | 10,157 | 22,13 | 0,444 | 0,13 |
| 4 | 11,481 | 146,86 | 0,502 | 0,88 |
| 5 | 12,057 | 638,99 | 0,527 | 3,81 |
| 6 | 12,311 | 121,08 | 0,538 | 0,72 |
| 7 | 12,591 | 190,73 | 0,550 | 1,14 |
| 8 | 14,927 | 125,1 | 0,652 | 0,75 |
| 9 | 20,417 | 1186,96 | 0,892 | 7,08 |
| 10 | 22,891 | 8557,01 | 1,000 | 51,07 |
| 11 | 36,011 | 1989,38 | 1,573 | 11,87 |
| 12 | 44,617 | 838,29 | 1,949 | 5 |
| 13 | 54,797 | 422,13 | 2,394 | 2,52 |
| 14 | 54,977 | 719,47 | 2,402 | 4,29 |
| 15 | 55,177 | 362,08 | 2,410 | 2,16 |
| 16 | 55,244 | 422,34 | 2,413 | 2,52 |
| 17 | 55,817 | 195,15 | 2,438 | 1,16 |
| 18 | 56,007 | 91,53 | 2,447 | 0,55 |
| 19 | 56,231 | 251,22 | 2,456 | 1,5 |
| 20 | 56,477 | 97,21 | 2,467 | 0,58 |
| 21 | 57,087 | 29 | 2,494 | 0,17 |
| 22 | 57,621 | 192,29 | 2,517 | 1,15 |
| 23 | 58,747 | 17,03 | 2,566 | 0,1 |
| 24 | 59,657 | 7,27 | 2,606 | 0,04 |
| 25 | 60,037 | 29,51 | 2,623 | 0,18 |
| 26 | 60,161 | 17,85 | 2,628 | 0,11 |
| 27 | 60,384 | 59,84 | 2,638 | 0,36 |

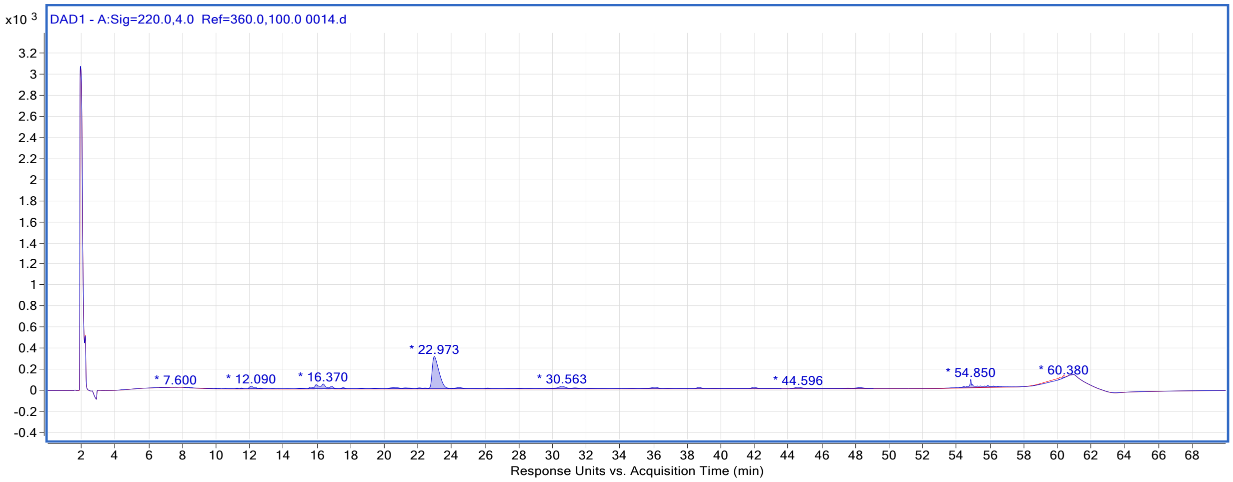


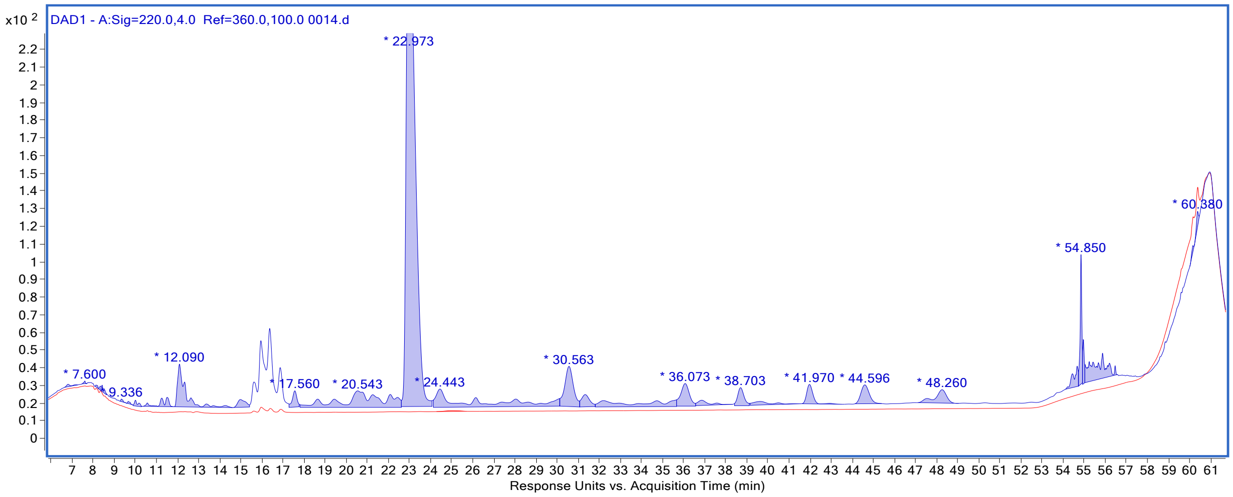


**Figure S25**. UV chromatogram from LC-HRMS analysis of exenatide crude prepared using 2,4-DMOT as thiol scavenger in TFA cleavage of exenatide peptide resin, UV chromatogram overview.

**Table S25.** Area% for integrated peaks.

| Peak | Rt | Area | RRT | Area % |
|---|---|---|---|---|
| 1 | 6,81 | 18,150 | 0,296 | 0,11 |
| 2 | 7,6 | 6,6 | 0,331 | 0,04 |
| 3 | 8,136 | 46,2 | 0,354 | 0,29 |
| 4 | 9,336 | 17,83 | 0,406 | 0,11 |
| 5 | 10,003 | 38,08 | 0,435 | 0,24 |
| 6 | 12,09 | 742,61 | 0,526 | 4,59 |
| 7 | 14,98 | 116,8 | 0,652 | 0,72 |
| 8 | 17,56 | 137,74 | 0,764 | 0,85 |
| 9 | 20,543 | 1161,3 | 0,894 | 7,17 |
| 10 | 22,973 | 8518,71 | 1,000 | 52,61 |
| 11 | 24,443 | 1015,48 | 1,064 | 6,27 |
| 12 | 30,563 | 654,86 | 1,330 | 4,04 |
| 13 | 31,333 | 189,32 | 1,364 | 1,17 |
| 14 | 35,666 | 505,35 | 1,553 | 3,12 |
| 15 | 36,073 | 371,51 | 1,570 | 2,29 |
| 16 | 36,86 | 105,51 | 1,604 | 0,65 |
| 17 | 38,703 | 216,62 | 1,685 | 1,34 |
| 18 | 39,64 | 113,65 | 1,726 | 0,7 |
| 19 | 41,97 | 229,61 | 1,827 | 1,42 |
| 20 | 44,596 | 295,38 | 1,941 | 1,82 |
| 21 | 48,26 | 291,26 | 2,101 | 1,8 |
| 22 | 54,666 | 181 | 2,380 | 1,12 |
| 23 | 54,85 | 398,23 | 2,388 | 2,46 |
| 24 | 54,96 | 115,48 | 2,392 | 0,71 |
| 25 | 55,87 | 624,33 | 2,432 | 3,86 |
| 26 | 60,156 | 21,87 | 2,619 | 0,14 |
| 27 | 60,38 | 57,18 | 2,628 | 0,35 |

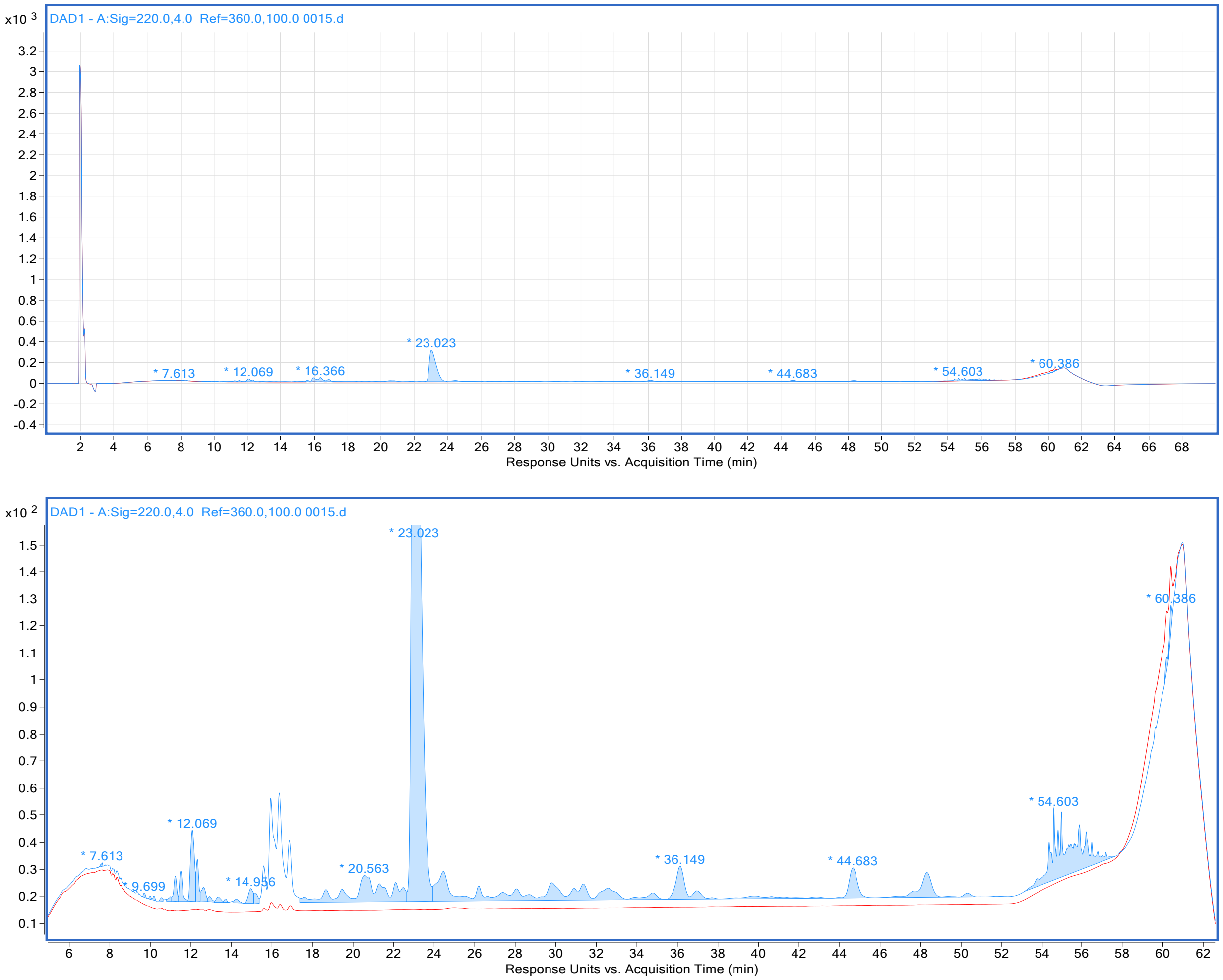


**Figure S26**. UV chromatogram from LC-HRMS analysis of exenatide crude prepared without a thiol scavenger in TFA cleavage of exenatide peptide resin, UV chromatogram overview.

**Table S26.** Area% for integrated peaks.

| Peak | Rt | Area | RRT | Area % |
|---|---|---|---|---|
| 1 | 7,61 | 7,77 | 0,331 | 0,05 |
| 2 | 9,70 | 10,97 | 0,421 | 0,07 |
| 3 | 9,99 | 18,27 | 0,434 | 0,12 |
| 4 | 10,55 | 11,32 | 0,458 | 0,07 |
| 5 | 11,00 | 18,58 | 0,478 | 0,12 |
| 6 | 11,23 | 94,56 | 0,488 | 0,60 |
| 7 | 11,50 | 119,50 | 0,500 | 0,76 |
| 8 | 12,07 | 355,24 | 0,524 | 2,26 |
| 9 | 12,32 | 139,43 | 0,535 | 0,89 |
| 10 | 12,62 | 82,44 | 0,548 | 0,52 |
| 11 | 12,92 | 25,46 | 0,561 | 0,16 |
| 12 | 13,35 | 32,33 | 0,580 | 0,21 |
| 13 | 13,71 | 11,62 | 0,596 | 0,07 |
| 14 | 14,24 | 21,46 | 0,618 | 0,14 |
| 15 | 14,96 | 86,17 | 0,650 | 0,55 |
| 16 | 15,10 | 50,50 | 0,656 | 0,32 |
| 17 | 20,56 | 1144,24 | 0,893 | 7,27 |
| 18 | 23,02 | 8610,68 | 1,000 | 54,73 |
| 19 | 36,15 | 2404,98 | 1,570 | 15,29 |
| 20 | 44,68 | 738,90 | 1,941 | 4,70 |
| 21 | 54,60 | 1632,59 | 2,372 | 10,38 |
| 22 | 60,26 | 43,19 | 2,618 | 0,27 |
| 23 | 60,39 | 72,82 | 2,623 | 0,46 |

## MS identities of peaks only observed for specific scavengers

1) DODT as scavenger

MS spectrum for t-Butyl ethyl sulphide adduct:

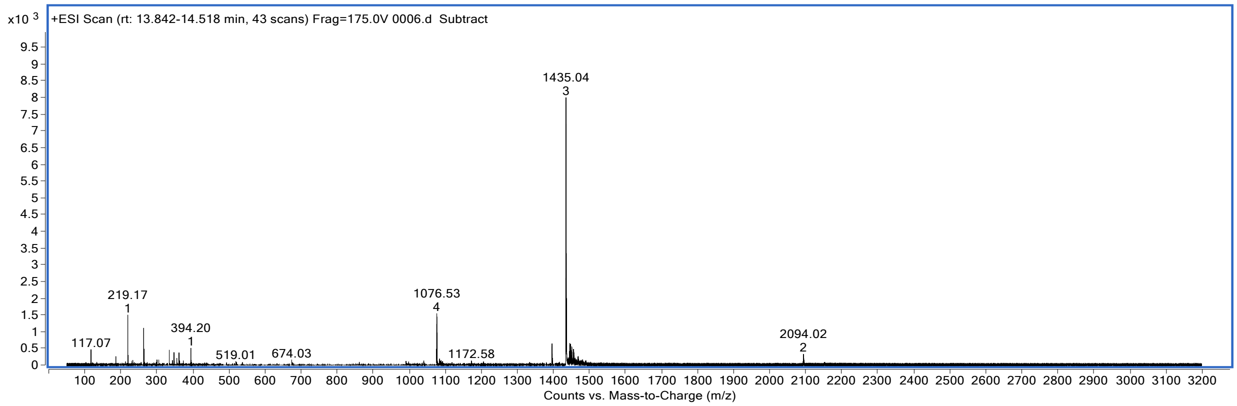


Identity:

| Peak | Rt | Area | most(m+z)/z | z | decon. | diff | | RRT | Area % | identity |
|---|---|---|---|---|---|---|---|---|---|---|
| 1 | 14,123 | 369,86 | 1434,3700 | 3 | 4300,0882 | 116,0612 | | 0,616 | 0,76 | t-butyl ethyl sulphide adduct[1] |

[1]The difference in MW between exenatide and this byproduct is 117 Da. Nevertheless, exenatide is protonated and thus detected as $M+H^+$. On the other hand the *t*-butyl ethyl sulphide adduct is detected in its sulfonium form for which $M^+$ is observed.

2) 4-MOBM as scavenger

MS spectrum for 165 Da non-peptide impurity:

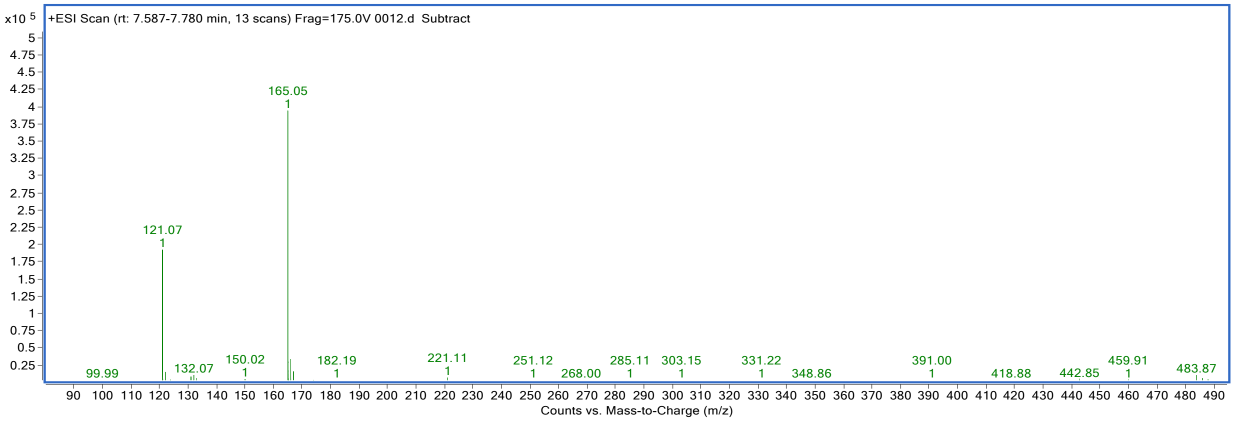


MS spectrum for 4-methylanisole adduct:

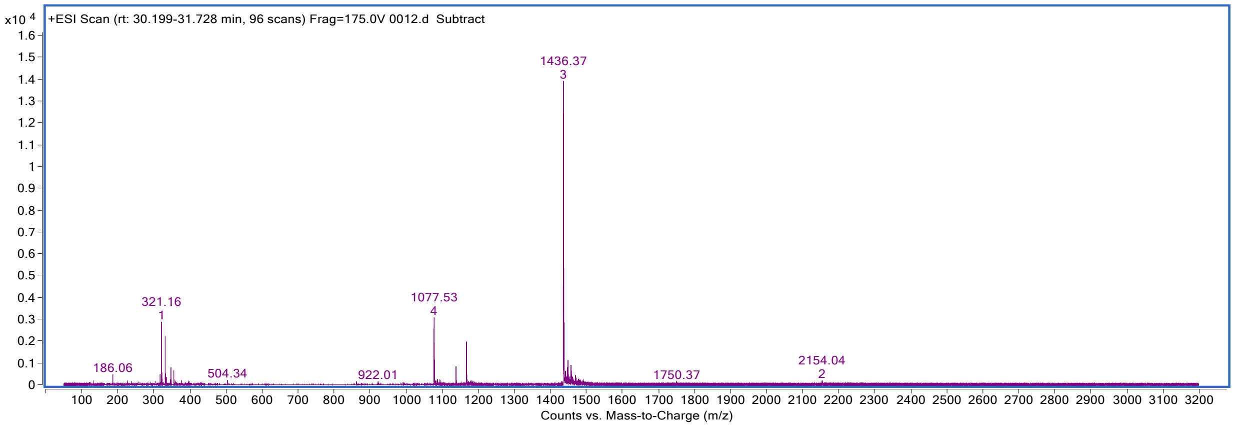


## Identitities:

| Peak | Rt | Area | most(m+z)/z | z | decon. | diff | RRT | Area % | identity |
|---|---|---|---|---|---|---|---|---|---|
| 1 | 7,71 | 1143,480 | 165,0500 | 1 | 164,0427 | -4019,9843 | 0,353 | 4,71 | non-peptide impurity |
| 2 | 31,02 | 1522,11 | 1435,7 | 3 | 4304,0782 | 120,0512 | 1,421 | 6,27 | + 120 Da (4-methylanisole adduct) |
| 3 | 57,91 | 748,84 | 121,07 | 1 | 120,0627 | -4063,9643 | 2,652 | 3,08 | non-peptide impurity |
| 4 | 58,37 | 2331,21 | 121,07 | 1 | 120,0627 | -4063,9643 | 2,673 | 9,60 | non-peptide impurity |

3) 2,4-DMOT as scavenger

MS spectrum for +1,3-dimethoxybenzene

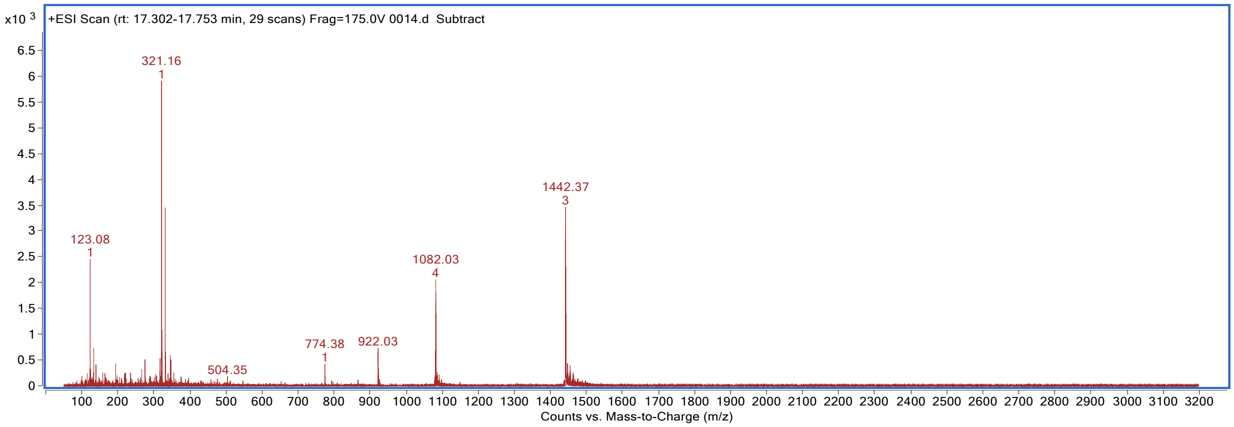


MS spectrum for +1,3-dimethoxybenzene/+t-Bu

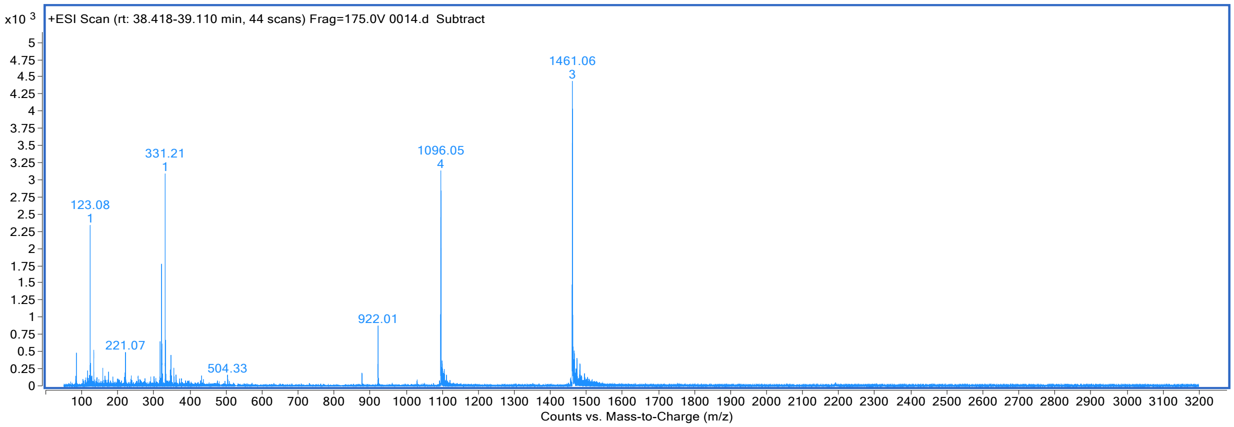


MS spectrum for +1,3-dimethoxybenzene/+t-Bu

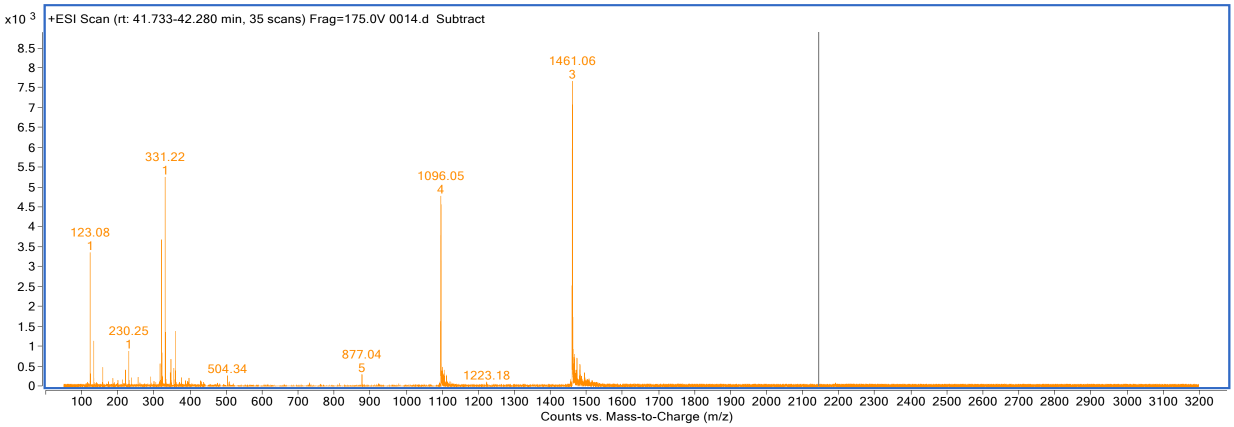

MS spectrum for adduct with 1,3-dimethoxybenzene/+t-Bu

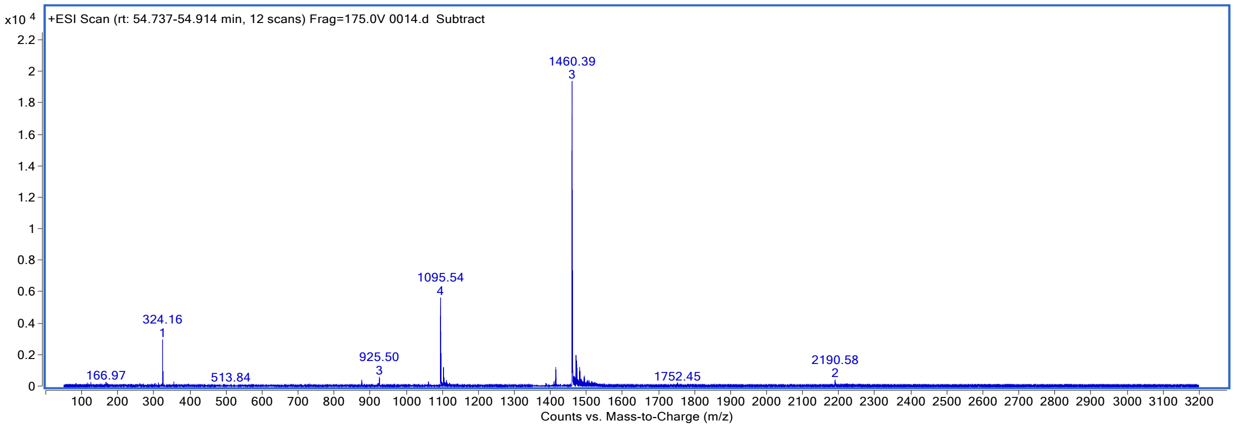


# Identitities:

| Peak | Rt | Area | most(m+z)/z | z | decon. | diff | RRT | Area % | identity |
|---|---|---|---|---|---|---|---|---|---|
| 1 | 17,56 | 137,74 | 1442,37 | 3 | 4322,0800 | 138,0080 | 0,764 | 0,85 | +1,3-Dimethoxybenzene |
| 2 | 38,703 | 216,62 | 1461,06 | 3 | 4378,1500 | 194,0780 | 1,685 | 1,34 | +1,3-Dimethoxybenzene/+t-Bu |
| 3 | 41,97 | 229,61 | 1461,06 | 3 | 4378,1500 | 194,0780 | 1,827 | 1,42 | +1,3-Dimethoxybenzene/+t-Bu |
| 4 | 54,85 | 398,23 | 1460,39 | 3 | 4376,1400 | 192,0680 | 2,388 | 2,46 | Adduct with 1,3-dimethoxybenzene/+t-Bu |

## 6. EIC-MS analyses of exenatide crudes

Upon performing LC-HRMS analyses on all crude exenatide samples as described above selected extracted ion chromatograms (EICs) were obtained by inspecting the original chromatograms at appropriate m/z values, resulting in the EICs pertaining to the specific cleavage related exenatide impurities investigated herein. To eliminate the risk that some of the peaks detected by EIC-MS were either other peptides and/or artefacts all peak assignments were a result of a two-step process: first, an EIC-MS analysis was carried out searching for the most abundant mass of a given impurity (z=+3) with the mass window of ±1 Da. Second, the mass spectra for the EIC-MS peaks thus obtained were inspected manually one by one and only the peaks for which the mass spectra were in full agreement with the expected mass spectrum of a given impurity were included as actual impurities. The peaks that were identified as hits in the initial screening round but subsequently did not fit with the expected mass spectrum were disregarded. The areas of these EICs were integrated and thus formed the basis for the comparison of the amounts of the specific impurities present in the different exenatide crudes. The EIC areas thus obtained were recalculated to UV areas as follows: the EIC area of the Met(O) impurity in the crude exenatide obtained from cleavage of exenatide peptide resin employing EDT as the scavenger was used as the reference.
EIC areas for following impurities were determined: i) add on *t*-Bu; ii) add on Pbf; iii) add on $SO_3$; iv) +16 Da Trp oxidation; v) Met to HCys demethylation; vi) Met to Met(O) oxidation; vii) scavenger adducts. The EIC areas for all the above impurities are summarized in the section 6.8 of this Electronic Supporting Information, the recalculation to the UV% areas is also included therein.

### 6.1 EIC-MS analysis of the content of add on *t*-Bu (+56 Da) byproducts

The original chromatograms were inspected at the m/z values corresponding to the add on *t*-Bu impurities: 4241,1 (z=+1) and the most abundant 1415,0 (z=+3).

#### 6.1.1 EIC-MS analysis of the content of add on *t*-Bu (+56 Da) byproducts in the crude exenatide from the cleavage using DTT as scavenger

EIC-MS from the initial m/z 1415 evaluation:

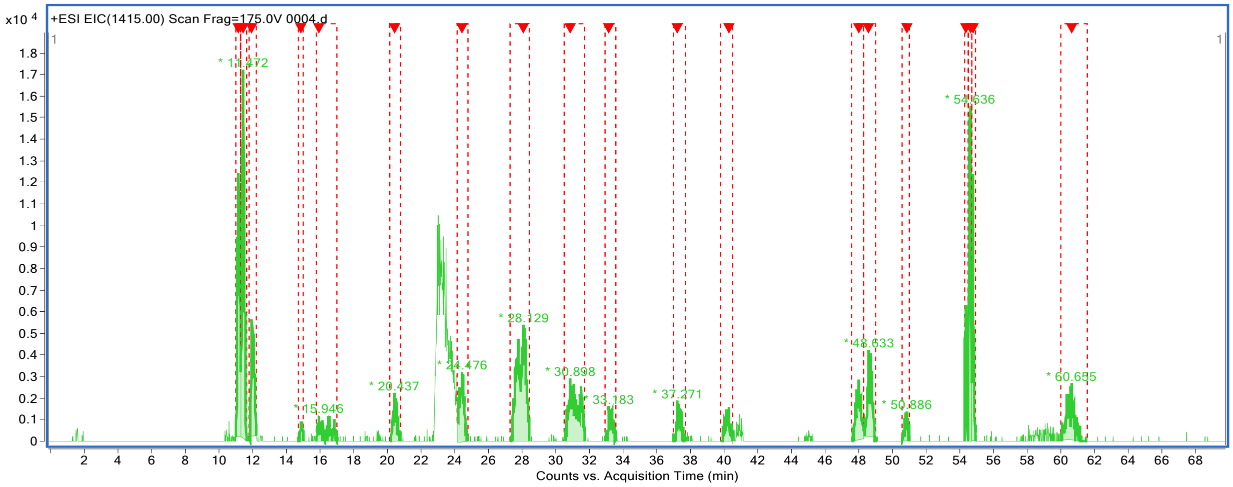

After evaluating MS spectra of the peaks identified in the initial EIC-MS following peaks were deemed as add on t-Bu byproducts:

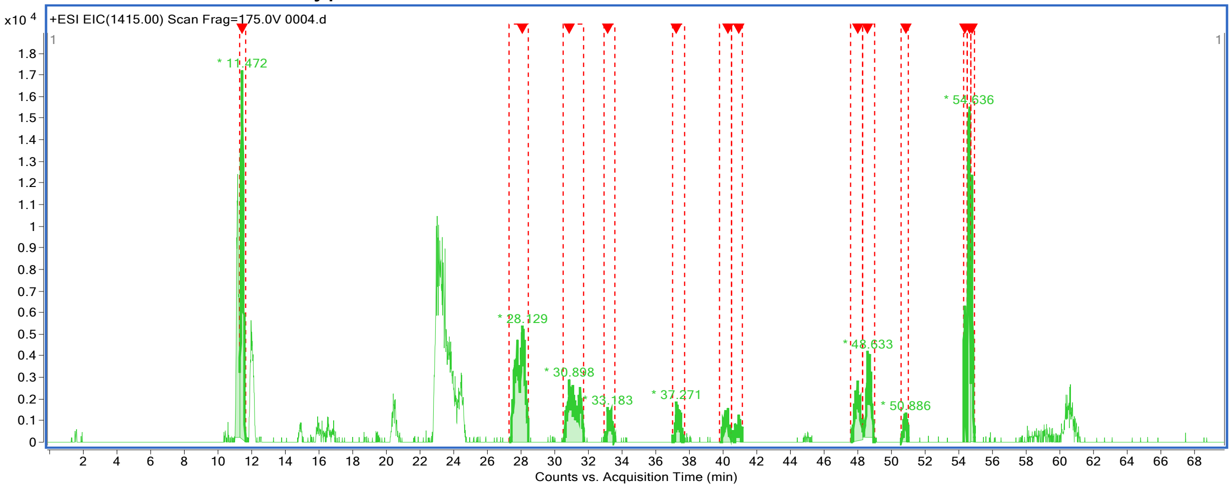


Summary of EIC areas for the identified add on *t*-Bu byproducts

| Peak | Rt | EIC area |
|---|---|---|
| 1 | 11,20 | 111515,00 |
| 2 | 11,47 | 162011,00 |
| 3 | 28,13 | 180823,00 |
| 4 | 30,90 | 106940,00 |
| 5 | 33,18 | 25668,00 |
| 6 | 37,27 | 25043,00 |
| 7 | 40,31 | 23660,00 |
| 8 | 40,96 | 13499,00 |
| 9 | 48,01 | 51142,00 |
| 10 | 48,63 | 60442,00 |
| 11 | 50,89 | 15435,00 |
| 12 | 54,41 | 37717,00 |
| 13 | 54,64 | 110143,00 |
| 14 | 54,81 | 64102,00 |
| $\sum$ | | **988140,00** |

### 6.1.2 EIC-MS analysis of the content of add on *t*-Bu (+56 Da) byproducts in the crude exenatide from the cleavage using EDT as scavenger

EIC-MS peaks identified as add on t-Bu byproducts: upper EIC-MS for DTT in the cleavage, lower EIC-MS for EDT in the cleavage

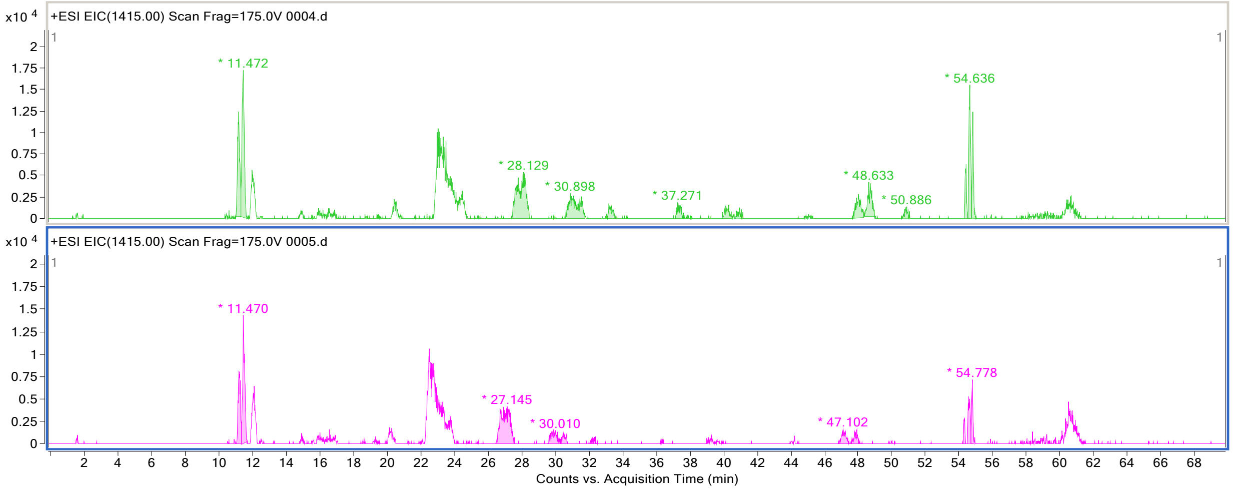


Summary of EIC areas for the identified add on *t*-Bu byproducts

| Peak | Rt | EIC area |
|---|---|---|
| 1 | 11,21 | 78379,69 |
| 2 | 11,47 | 116754,46 |
| 3 | 27,15 | 152099,08 |
| 4 | 30,01 | 49682,60 |
| 5 | 32,41 | 7231,24 |
| 6 | 47,10 | 26364,32 |
| 7 | 47,92 | 19714,42 |
| 8 | 54,31 | 15066,47 |
| 9 | 54,54 | 45924,86 |
| 10 | 54,78 | 37654,64 |
| $\sum$ | | **548871,78** |

**6.1.3 EIC-MS analysis of the content of add on *t*-Bu (+56 Da) byproducts in the crude exenatide from the cleavage using DODT as scavenger**

EIC-MS peaks identified as add on t-Bu byproducts: upper EIC-MS for DTT in the cleavage, lower EIC-MS for DODT in the cleavage

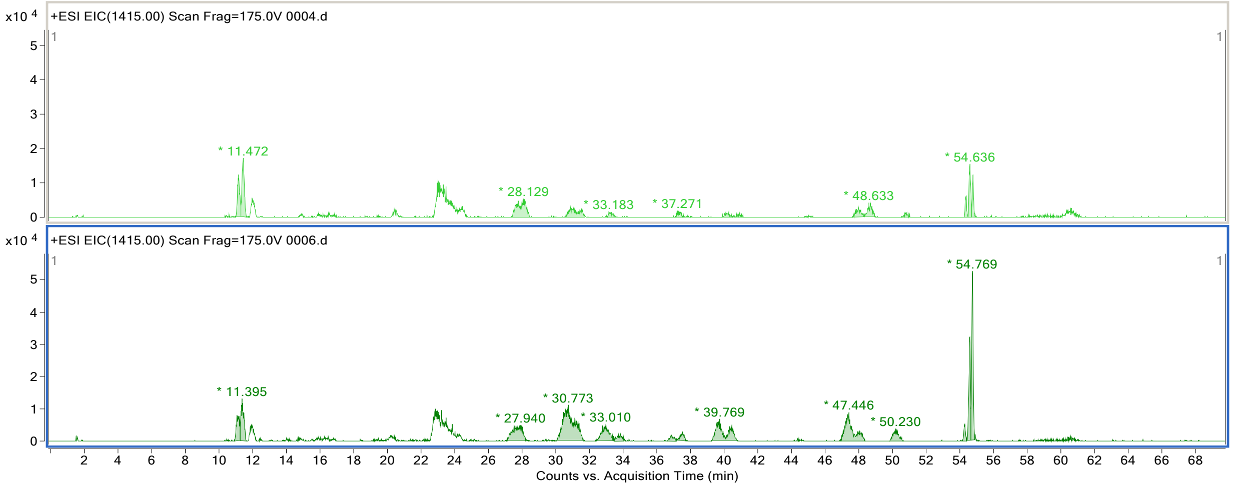


Summary of EIC areas for the identified add on *t*-Bu byproducts

| Peak | Rt | EIC area |
|---|---|---|
| 1 | 11,09 | 98708,69 |
| 2 | 11,40 | 143844,95 |
| 3 | 27,94 | 180199,10 |
| 4 | 30,77 | 459678,75 |
| 5 | 33,01 | 133923,10 |
| 6 | 33,88 | 41198,27 |
| 7 | 36,92 | 29480,51 |
| 8 | 37,55 | 35631,31 |
| 9 | 39,77 | 224957,74 |
| 10 | 47,45 | 270924,16 |
| 11 | 50,23 | 75591,61 |
| 12 | 54,30 | 35212,70 |
| 13 | 54,62 | 217325,96 |
| 14 | 54,77 | 310760,36 |
| $\sum$ | | **2257437,21** |

### 6.1.4 EIC-MS analysis of the content of add on *t*-Bu (+56 Da) byproducts in the crude exenatide from the cleavage using 1,4-BDMT as scavenger

EIC-MS peaks identified as add on t-Bu byproducts: upper EIC-MS for DTT in the cleavage, lower EIC-MS for 1,4-BDMT in the cleavage

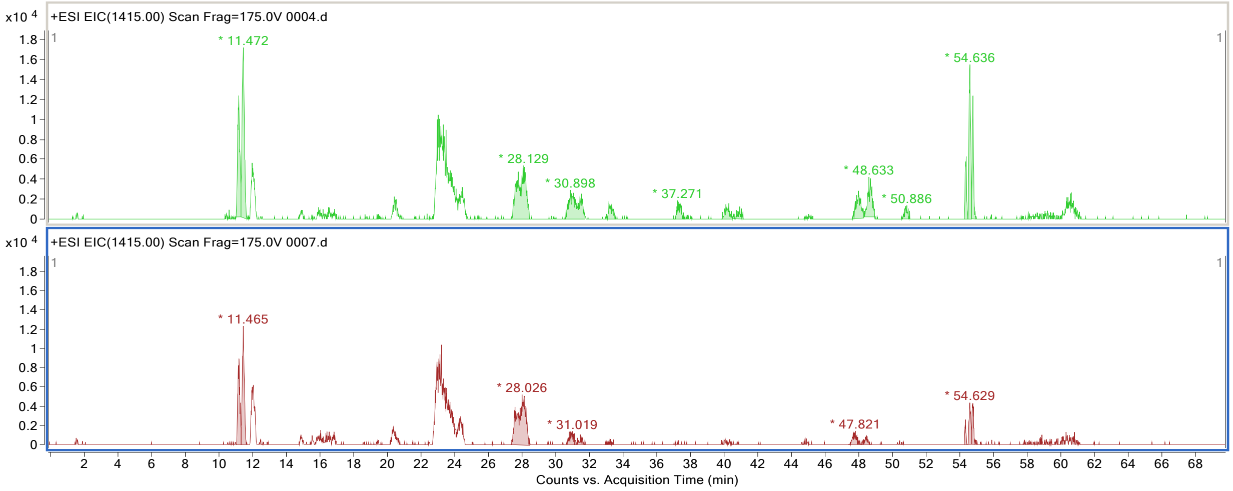


Summary of EIC areas for the identified add on *t*-Bu byproducts

| Peak | Rt | EIC area |
|---|---|---|
| 1 | 11,21 | 76851,79 |
| 2 | 11,47 | 101135,35 |
| 3 | 28,03 | 172163,46 |
| 4 | 31,02 | 20846,74 |
| 5 | 31,49 | 9236,80 |
| 6 | 33,27 | 4278,49 |
| 7 | 47,82 | 20785,60 |
| 8 | 48,48 | 11186,74 |
| 9 | 54,37 | 11017,46 |
| 10 | 54,63 | 33664,21 |
| 11 | 54,81 | 26015,87 |
| Σ | | **487182,51** |

### 6.1.5 EIC-MS analysis of the content of add on *t*-Bu (+56 Da) byproducts in the crude exenatide from the cleavage using 1,3-BDMT as scavenger

EIC-MS peaks identified as add on t-Bu byproducts upper EIC-MS for DTT in the cleavage, lower EIC-MS for 1,3-BDMT in the cleavage

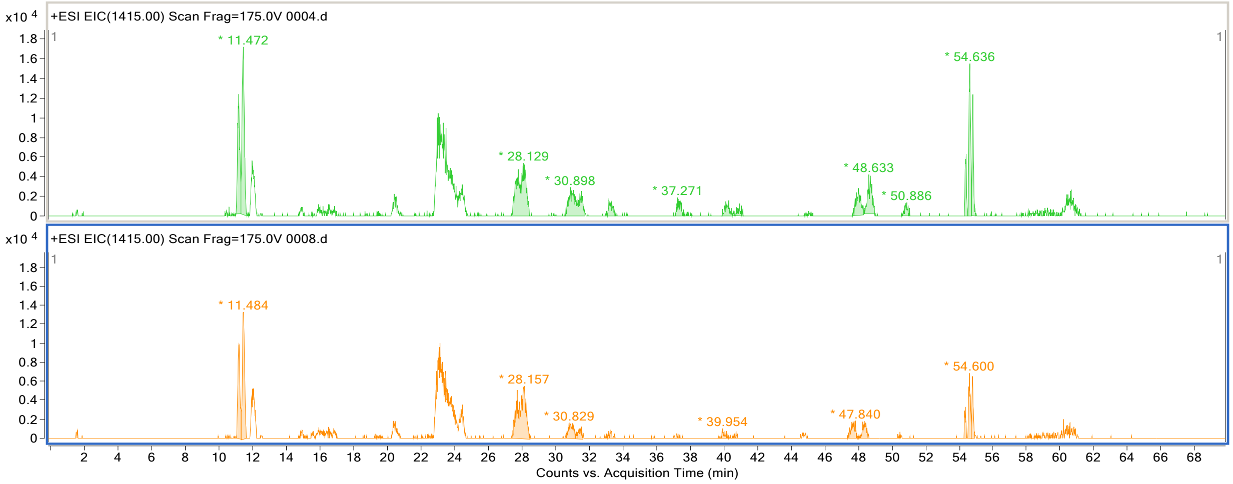


Summary of EIC areas for the identified add on *t*-Bu byproducts

| Peak | Rt | EIC area |
|---|---|---|
| 1 | 11,21 | 91183,77 |
| 2 | 11,48 | 125741,73 |
| 3 | 28,16 | 173585,87 |
| 4 | 30,83 | 52721,95 |
| 5 | 33,26 | 10086,12 |
| 6 | 37,23 | 4104,19 |
| 7 | 39,95 | 14521,22 |
| 8 | 47,84 | 32051,21 |
| 9 | 48,36 | 26394,17 |
| 10 | 54,36 | 18339,17 |
| 11 | 54,60 | 48150,91 |
| 12 | 54,78 | 37290,07 |
| $\sum$ | | **634170,38** |

### 6.1.6 EIC-MS analysis of the content of add on *t*-Bu (+56 Da) byproducts in the crude exenatide from the cleavage using 1,2-BDMT as scavenger

EIC-MS peaks identified as add on t-Bu byproducts: upper EIC-MS for DTT in the cleavage, lower EIC-MS for 1,2-BDMT in the cleavage

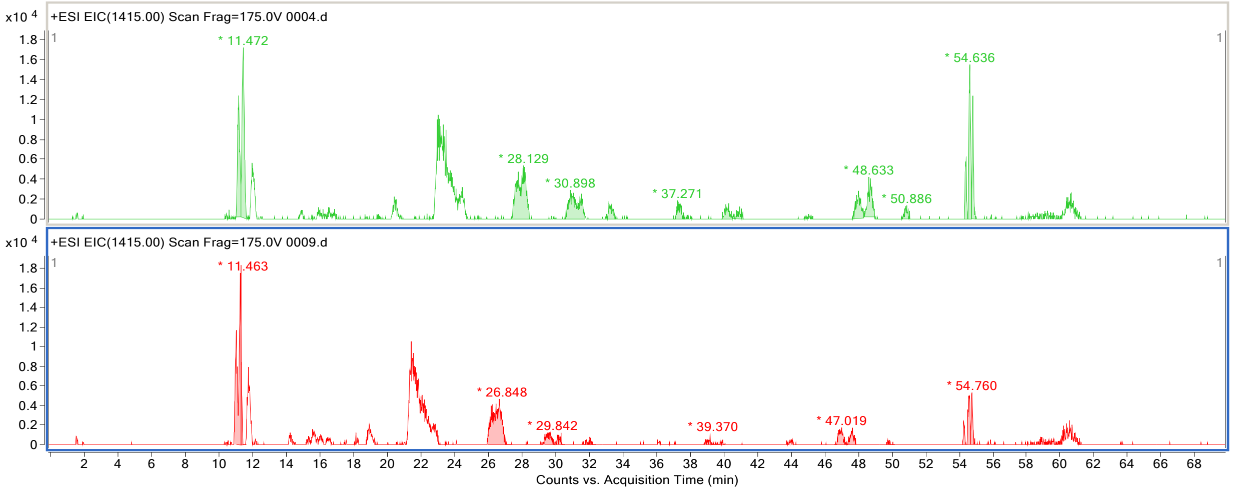


Summary of EIC areas for the identified add on *t*-Bu byproducts

| Peak | Rt | EIC area |
|---|---|---|
| 1 | 11,24 | 99326,99 |
| 2 | 11,46 | 136189,79 |
| 3 | 26,85 | 164178,39 |
| 4 | 29,84 | 36952,86 |
| 5 | 32,22 | 6002,95 |
| 6 | 39,37 | 10115,41 |
| 7 | 47,02 | 49981,44 |
| 8 | 49,76 | 2872,27 |
| 9 | 54,26 | 13366,61 |
| 10 | 54,60 | 42990,91 |
| 11 | 54,76 | 30980,50 |
| $\Sigma$ | | **592958,12** |

### 6.1.7 EIC-MS analysis of the content of add on *t*-Bu (+56 Da) byproducts in the crude exenatide from the cleavage using 4,4'-BMMB as scavenger

EIC-MS peaks identified as add on t-Bu byproducts: upper EIC-MS for DTT in the cleavage, lower EIC-MS for 4,4'-BMMB in the cleavage

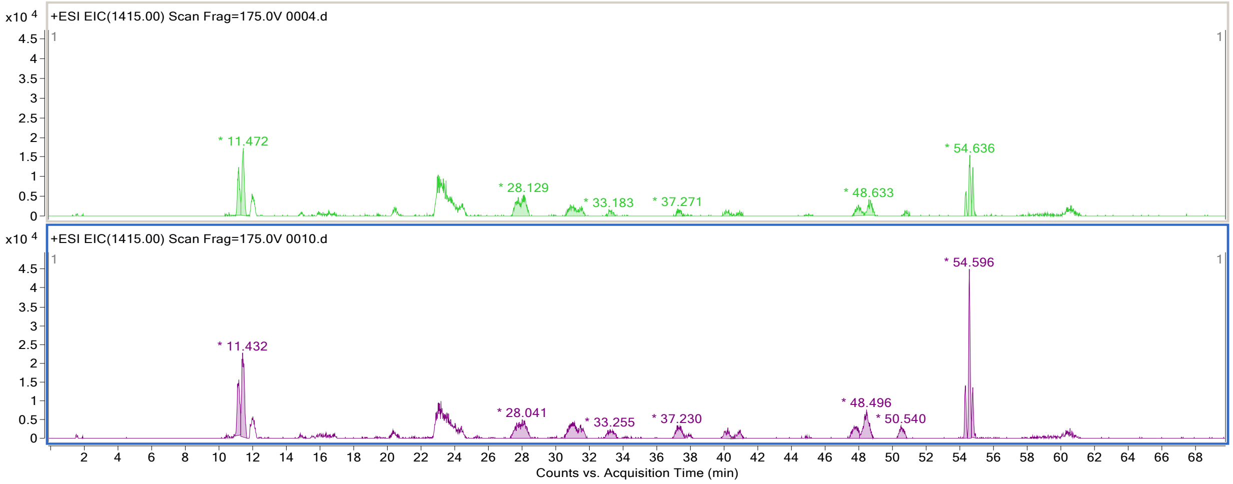


Summary of EIC areas for the identified add on *t*-Bu byproducts

| Peak | Rt | EIC area |
|---|---|---|
| 1 | 11,21 | 161324,35 |
| 2 | 11,43 | 257609,00 |
| 3 | 28,04 | 185125,12 |
| 4 | 31,03 | 185213,48 |
| 5 | 33,26 | 57628,15 |
| 6 | 37,23 | 74717,16 |
| 7 | 40,26 | 45069,43 |
| 8 | 41,00 | 37127,24 |
| 9 | 47,76 | 77485,22 |
| 10 | 48,50 | 155804,90 |
| 11 | 50,54 | 57858,29 |
| 12 | 54,37 | 90034,26 |
| 13 | 54,60 | 282357,27 |
| 14 | 54,81 | 82691,88 |
| Σ | | **1750045,75** |

### 6.1.8 EIC-MS analysis of the content of add on *t*-Bu (+56 Da) byproducts in the crude exenatide from the cleavage using 2,4-DCBM as scavenger

EIC-MS peaks identified as add on t-Bu byproducts: upper EIC-MS for DTT in the cleavage, lower EIC-MS for 2,4-DCBM in the cleavage

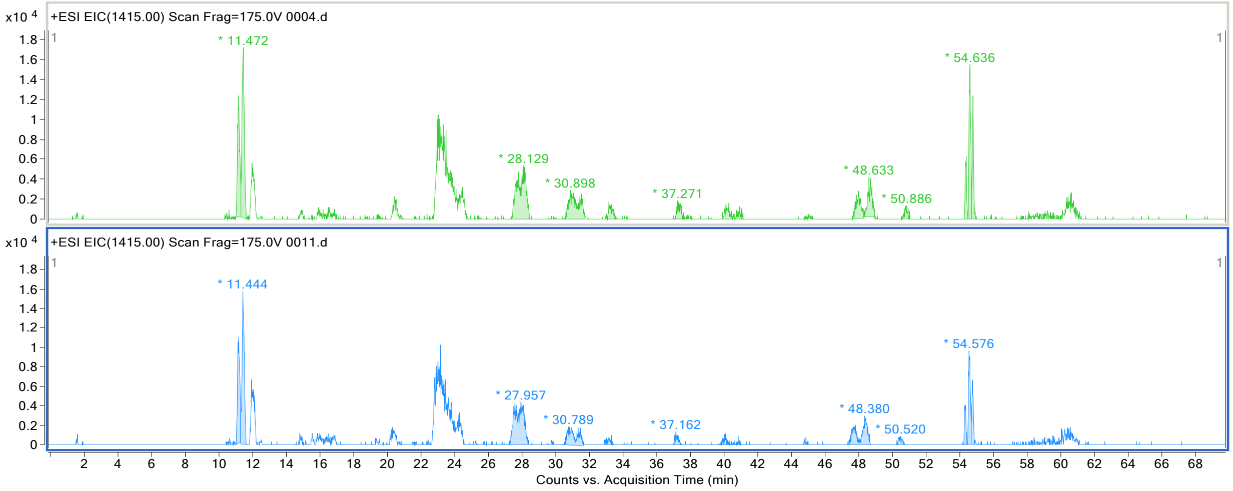


Summary of EIC areas for the identified add on *t*-Bu byproducts

| Peak | Rt | EIC area |
|---|---|---|
| 1 | 11,20 | 103012,25 |
| 2 | 11,44 | 144333,85 |
| 3 | 27,96 | 163589,57 |
| 4 | 30,79 | 66420,66 |
| 5 | 33,35 | 10181,53 |
| 6 | 37,16 | 13537,65 |
| 7 | 40,11 | 16808,64 |
| 8 | 48,38 | 88630,96 |
| 9 | 50,52 | 10453,06 |
| 10 | 54,37 | 26772,29 |
| 11 | 54,58 | 74326,34 |
| 12 | 54,79 | 40725,12 |
| Σ | | **758791,92** |

**6.1.9 EIC-MS analysis of the content of add on *t*-Bu (+56 Da) byproducts in the crude exenatide from the cleavage using 4-MOBM as scavenger**

EIC-MS peaks identified as add on t-Bu byproducts: upper EIC-MS for DTT in the cleavage, lower EIC-MS for 4-MOBM in the cleavage

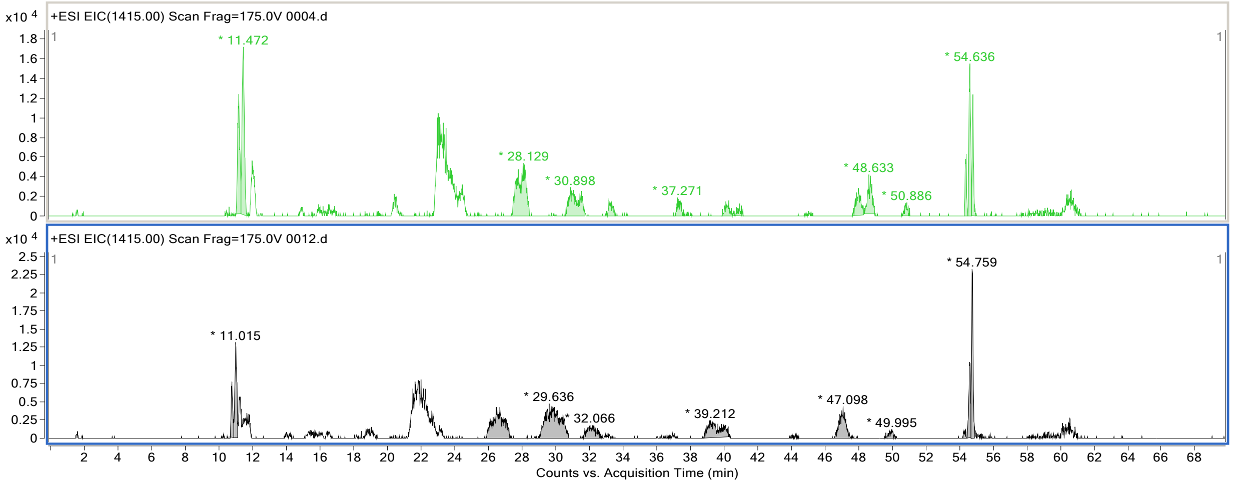


Summary of EIC areas for the identified add on *t*-Bu byproducts

| Peak | Rt | EIC area |
|---|---|---|
| 1 | 10,79 | 50192,05 |
| 2 | 11,02 | 99498,12 |
| 3 | 26,53 | 183232,21 |
| 4 | 29,64 | 260471,55 |
| 5 | 32,07 | 63527,32 |
| 6 | 39,21 | 109360,83 |
| 7 | 47,10 | 102623,79 |
| 8 | 50,00 | 19927,39 |
| 9 | 54,36 | 8867,64 |
| 10 | 54,61 | 77818,71 |
| 11 | 54,76 | 140306,70 |
| $\sum$ | | **1115826,31** |

### 6.1.10 EIC-MS analysis of the content of add on *t*-Bu (+56 Da) byproducts in the crude exenatide from the cleavage using TPMT as scavenger

EIC-MS peaks identified as add on t-Bu byproducts upper EIC-MS for DTT in the cleavage, lower EIC-MS for TPMT in the cleavage

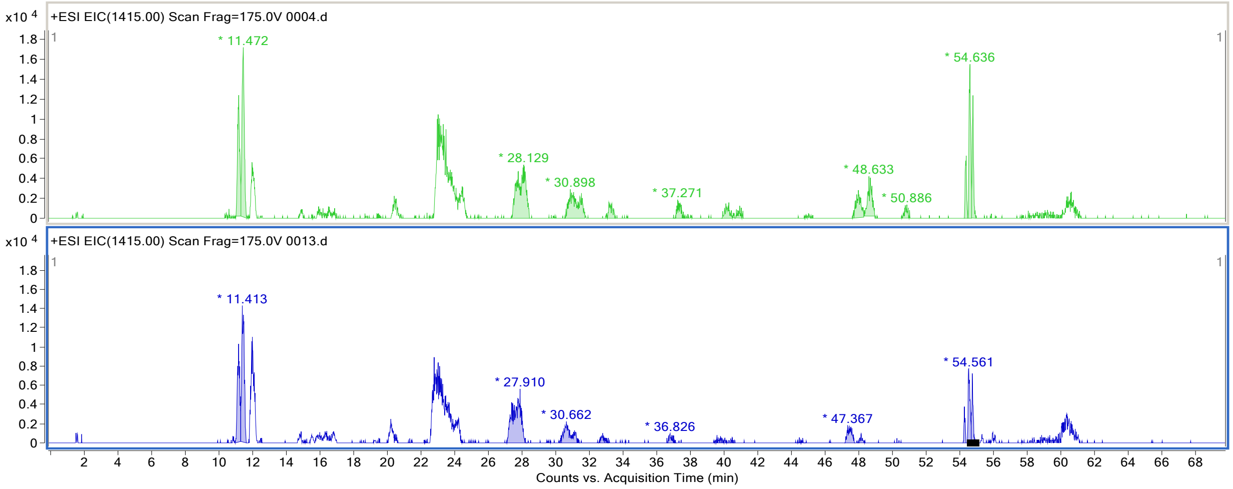


Summary of EIC areas for the identified add on *t*-Bu byproducts

| Peak | Rt | EIC area |
|---|---|---|
| 1 | 11,19 | 97054,74 |
| 2 | 11,41 | 147365,58 |
| 3 | 27,91 | 168298,40 |
| 4 | 30,66 | 60467,62 |
| 5 | 32,82 | 12367,06 |
| 6 | 36,83 | 11600,21 |
| 7 | 39,61 | 14758,29 |
| 8 | 44,50 | 3305,84 |
| 9 | 47,37 | 31061,76 |
| 10 | 48,17 | 7327,38 |
| 11 | 54,30 | 16986,00 |
| 12 | 54,56 | 61979,04 |
| 13 | 54,77 | 37587,14 |
| ∑ | | **670159,06** |

### 6.1.11 EIC-MS analysis of the content of add on *t*-Bu (+56 Da) byproducts in the crude exenatide from the cleavage using 2,4-DMOT as scavenger

EIC-MS peaks identified as add on t-Bu byproducts: upper EIC-MS using DTT in the cleavage, lower EIC-MS using 2,4-DMOT in the cleavage

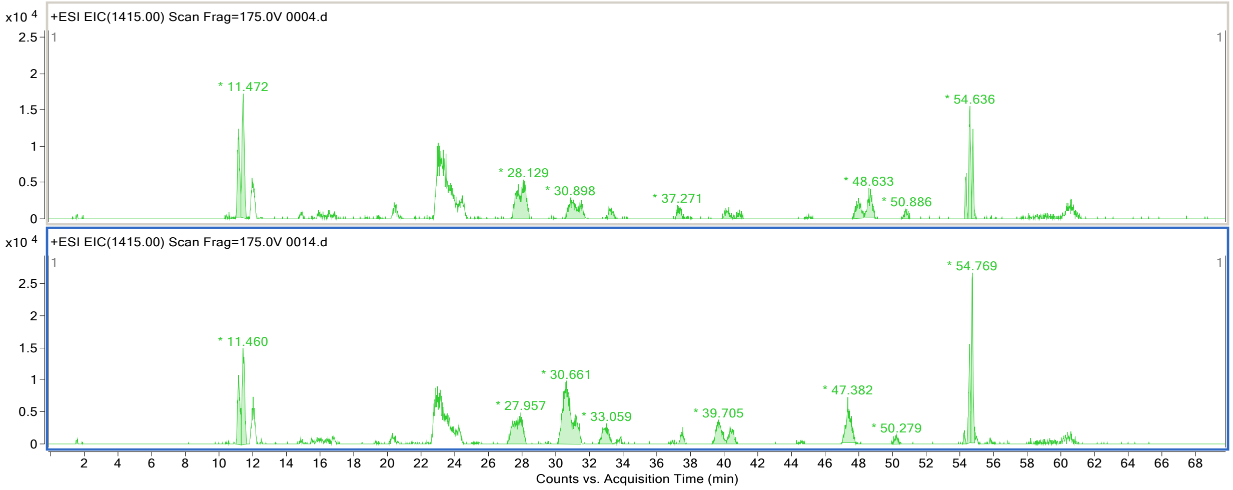


Summary of EIC areas for the identified add on *t*-Bu byproducts

| Peak | Rt | EIC area |
|---|---|---|
| 1 | 11,19 | 110155,25 |
| 2 | 11,46 | 153404,24 |
| 3 | 27,96 | 166365,59 |
| 4 | 30,66 | 361562,22 |
| 5 | 33,06 | 71181,99 |
| 6 | 37,58 | 23601,66 |
| 7 | 39,71 | 134090,46 |
| 8 | 47,38 | 115379,34 |
| 9 | 50,28 | 18394,36 |
| 10 | 54,30 | 11447,84 |
| 11 | 54,61 | 106585,39 |
| 12 | 54,77 | 158999,17 |
| Σ | | **1431167,51** |

### 6.1.12 EIC-MS analysis of the content of add on *t*-Bu (+56 Da) byproducts in the crude exenatide from the cleavage not using a thiol scavenger

EIC-MS peaks identified as add on t-Bu byproducts: in green using DTT in the cleavage, in in pink not using thiol in the cleavage

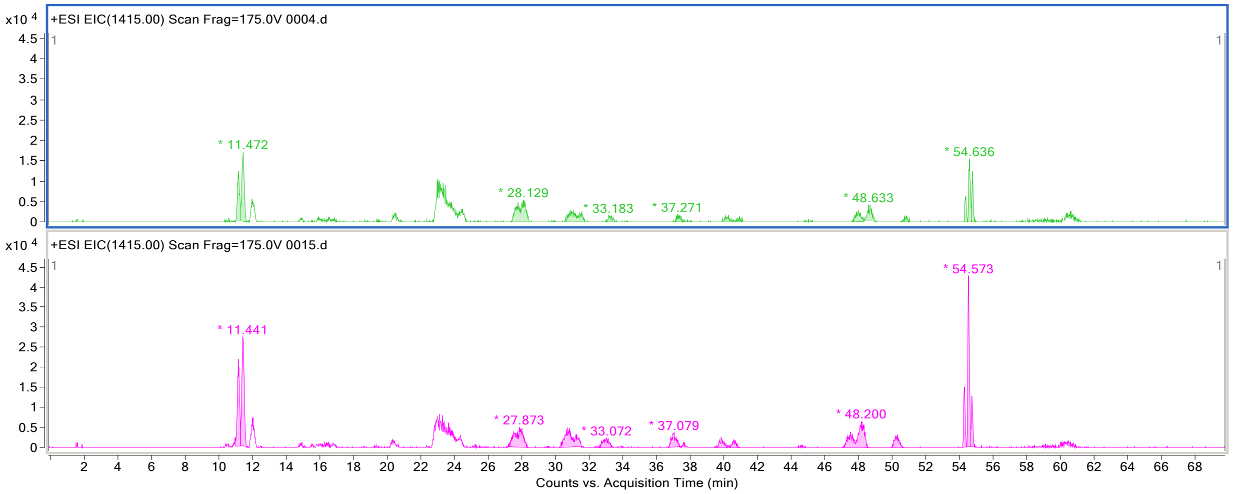


Summary of EIC areas for the identified add on *t*-Bu byproducts

| **Peak** | **Rt** | **EIC area** |
|---|---|---|
| 1 | 11,18 | 190592,87 |
| 2 | 11,44 | 269128,94 |
| 3 | 27,87 | 166144,69 |
| 4 | 30,75 | 167140,54 |
| 5 | 33,07 | 57250,02 |
| 6 | 37,08 | 68003,74 |
| 7 | 39,86 | 66201,73 |
| 8 | 48,20 | 225498,85 |
| 9 | 50,21 | 54445,45 |
| 10 | 54,33 | 91981,94 |
| 11 | 54,57 | 276337,13 |
| 12 | 54,77 | 76157,49 |
| ∑ | | **1708883,39** |

**6.2 EIC-MS analysis of the content of add on Pbf (+252 Da) byproducts**

The original chromatograms were inspected at the m/z values corresponding to the add on Pbf impurities: 4437,1 (z=+1) and the most abundant 1480,4 (z=+3).

**6.2.1 EIC-MS analysis of the content of add on Pbf (+252 Da) byproducts in the crude exenatide from the cleavage using DTT as scavenger**

EIC-MS peaks identified as add on Pbf byproducts

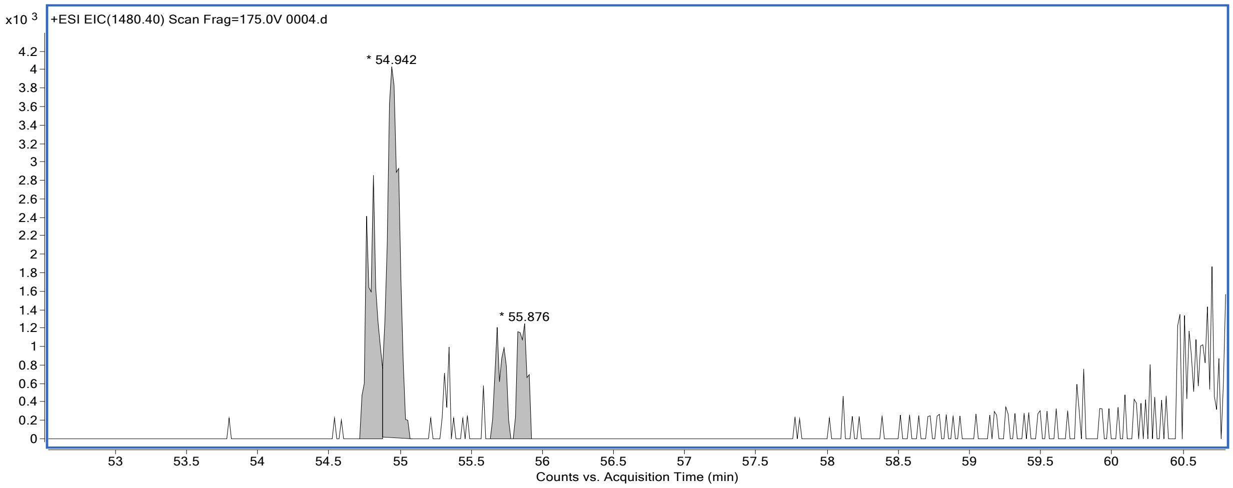


Summary of EIC areas for the identified add on Pbf byproducts

| Peak | Rt | EIC area |
|---|---|---|
| 1 | 54,81 | 13373,04 |
| 2 | 54,94 | 22694,05 |
| 3 | 55,68 | 5387,88 |
| 4 | 55,88 | 6016,72 |
| $\sum$ | | **47471,69** |

### 6.2.2 EIC-MS analysis of the content of add on Pbf (+252 Da) byproducts in the crude exenatide from the cleavage using EDT as scavenger

EIC-MS peaks identified as add on Pbf byproducts: upper EIC-MS using DTT in the cleavage, lower EIC-MS using EDT in the cleavage

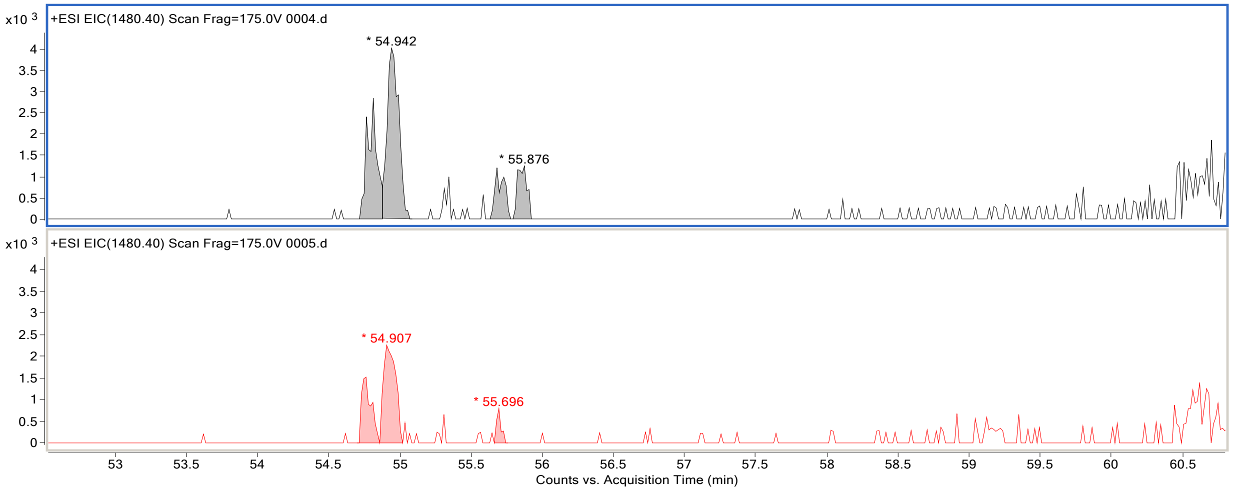


Summary of EIC areas for the identified add on Pbf byproducts

| Peak | Rt | EIC area |
|---|---|---|
| 1 | 54,76 | 7236,02 |
| 2 | 54,91 | 13762,84 |
| 3 | 55,70 | 1773,94 |
| $\sum$ | | **22772,80** |

**6.2.3 EIC-MS analysis of the content of add on Pbf (+252 Da) byproducts in the crude exenatide from the cleavage using DODT as scavenger**

EIC-MS peaks identified as add on Pbf byproducts: upper EIC-MS using DTT in the cleavage, lower EIC-MS using DODT in the cleavage

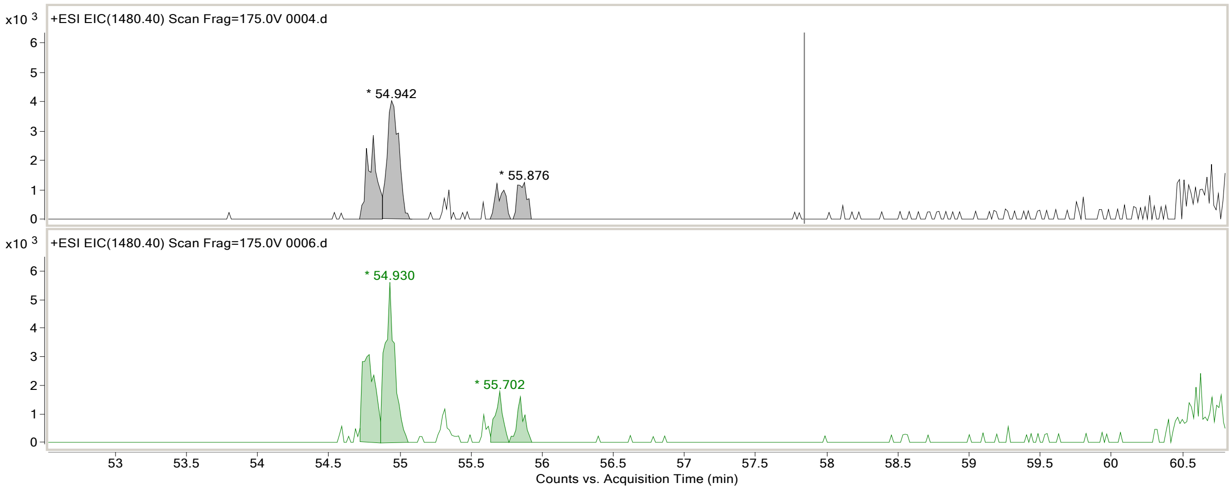


Summary of EIC areas for the identified add on Pbf byproducts

| Peak | Rt | EIC area |
|---|---|---|
| 1 | 54,79 | 19312,79 |
| 2 | 54,93 | 26641,65 |
| 3 | 55,70 | 6467,65 |
| 4 | 55,85 | 5762,26 |
| Σ | | **58184,35** |

### 6.2.4 EIC-MS analysis of the content of add on Pbf (+252 Da) byproducts in the crude exenatide from the cleavage using 1,4-BDMT as scavenger

EIC-MS peaks identified as add on Pbf byproducts: upper EIC-MS using DTT in the cleavage, lower EIC-MS using 1,4-BDMT in the cleavage

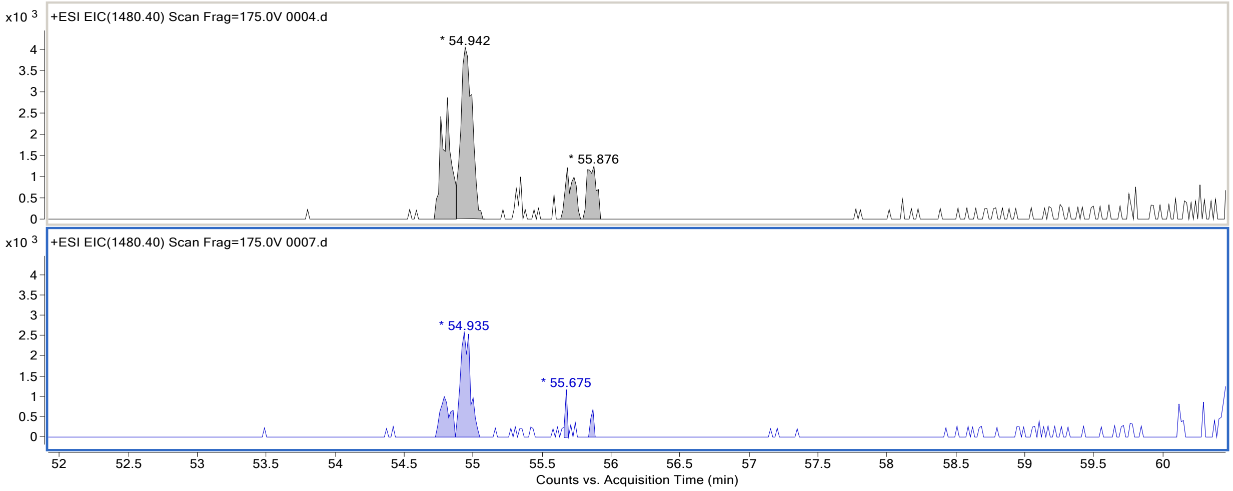


Summary of EIC areas for the identified add on Pbf byproducts

| Peak | Rt | EIC area |
|---|---|---|
| 1 | 54,79 | 5107,70 |
| 2 | 54,94 | 13111,00 |
| 3 | 55,68 | 1142,78 |
| 4 | 55,87 | 1101,41 |
| $\Sigma$ | | **20462,89** |

### 6.2.5 EIC-MS analysis of the content of add on Pbf (+252 Da) byproducts in the crude exenatide from the cleavage using 1,3-BDMT as scavenger

EIC-MS peaks identified as add on Pbf byproducts: upper EIC-MS using DTT in the cleavage, lower EIC-MS using 1,3-BDMT in the cleavage

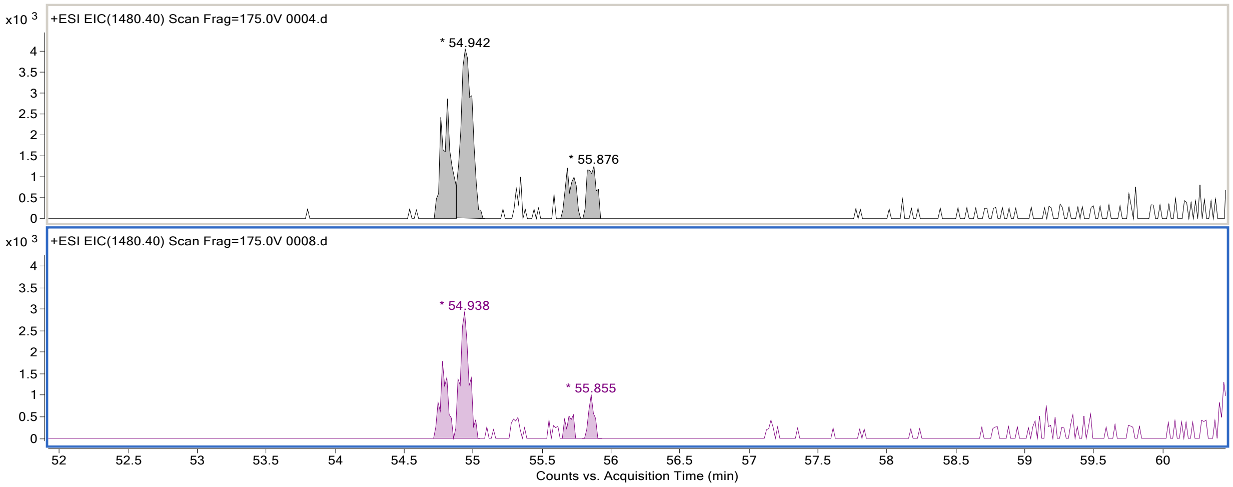


Summary of EIC areas for the identified add on Pbf byproducts

| Peak | Rt | EIC area |
|---|---|---|
| 1 | 54,78 | 6935,65 |
| 2 | 54,94 | 13500,43 |
| 3 | 55,73 | 2094,36 |
| 4 | 55,86 | 2826,10 |
| ∑ | | **25356,54** |

### 6.2.6 EIC-MS analysis of the content of add on Pbf (+252 Da) byproducts in the crude exenatide from the cleavage using 1,2-BDMT as scavenger

EIC-MS peaks identified as add on Pbf byproducts: upper EIC-MS using DTT in the cleavage, lower EIC-MS using 1,2-BDMT in the cleavage

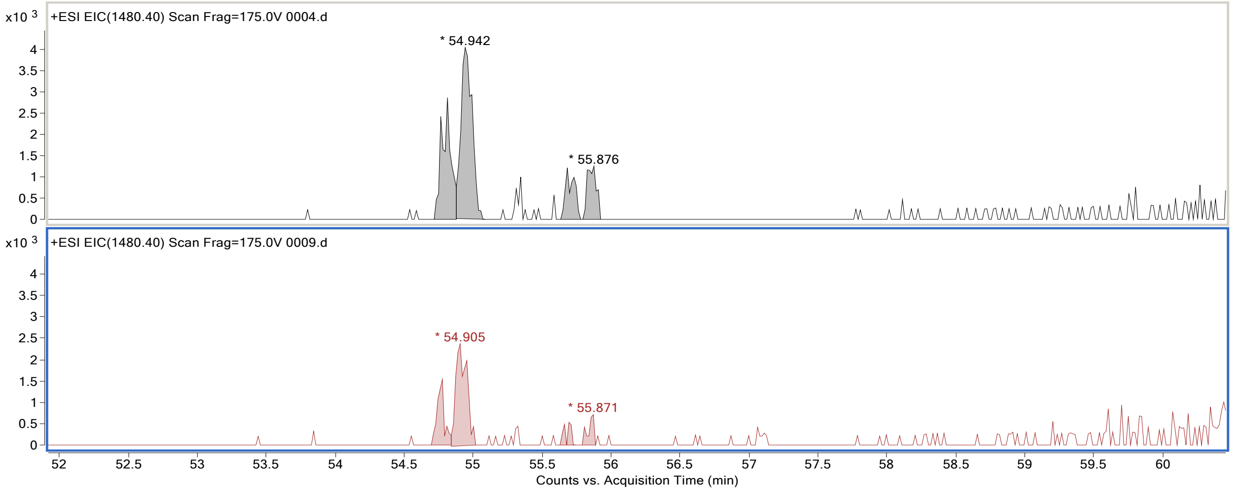


Summary of EIC areas for the identified add on Pbf byproducts

| Peak | Rt | EIC area |
|---|---|---|
| 1 | 54,78 | 5546,82 |
| 2 | 54,91 | 13520,07 |
| 3 | 55,69 | 1698,89 |
| 4 | 55,87 | 2126,16 |
| ∑ | | **22891,94** |

**6.2.7 EIC-MS analysis of the content of add on Pbf (+252 Da) byproducts in the crude exenatide from the cleavage using 4,4'-BMMB as scavenger**

EIC-MS peaks identified as add on Pbf byproducts: upper EIC-MS using DTT in the cleavage, lower EIC-MS using 4,4'-BMMB in the cleavage

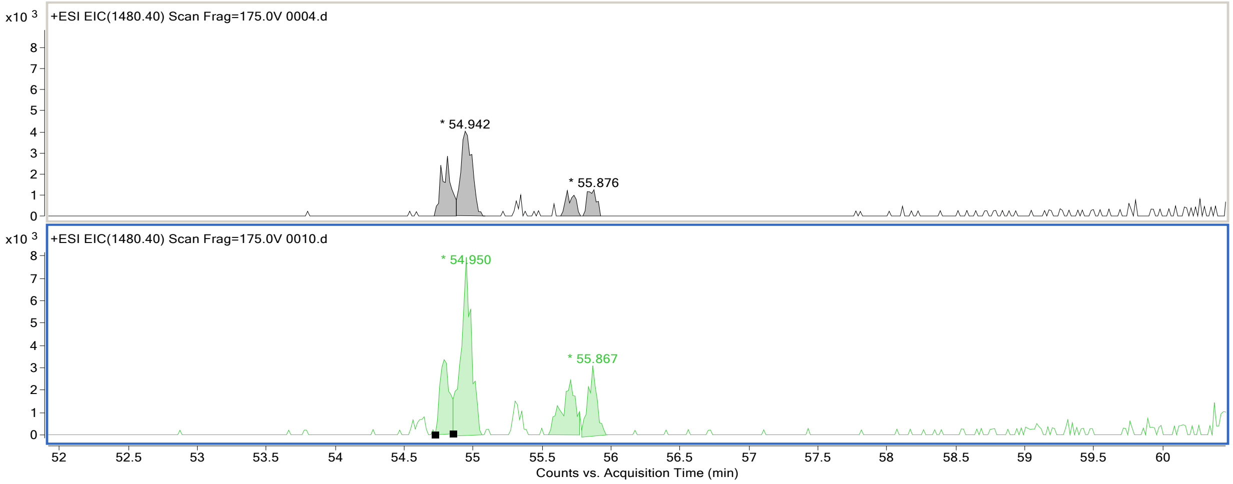


Summary of EIC areas for the identified add on Pbf byproducts

| Peak | Rt | EIC area |
|---|---|---|
| 1 | 54,79 | 16243,55 |
| 2 | 54,95 | 41087,91 |
| 3 | 55,71 | 17279,74 |
| 4 | 55,87 | 13906,32 |
| Σ | | **88517,52** |

### 6.2.8 EIC-MS analysis of the content of add on Pbf (+252 Da) byproducts in the crude exenatide from the cleavage using 2,4-DCBM as scavenger

EIC-MS peaks identified as add on Pbf byproducts: upper EIC-MS using DTT in the cleavage, lower EIC-MS using 2,4-DCBM in the cleavage

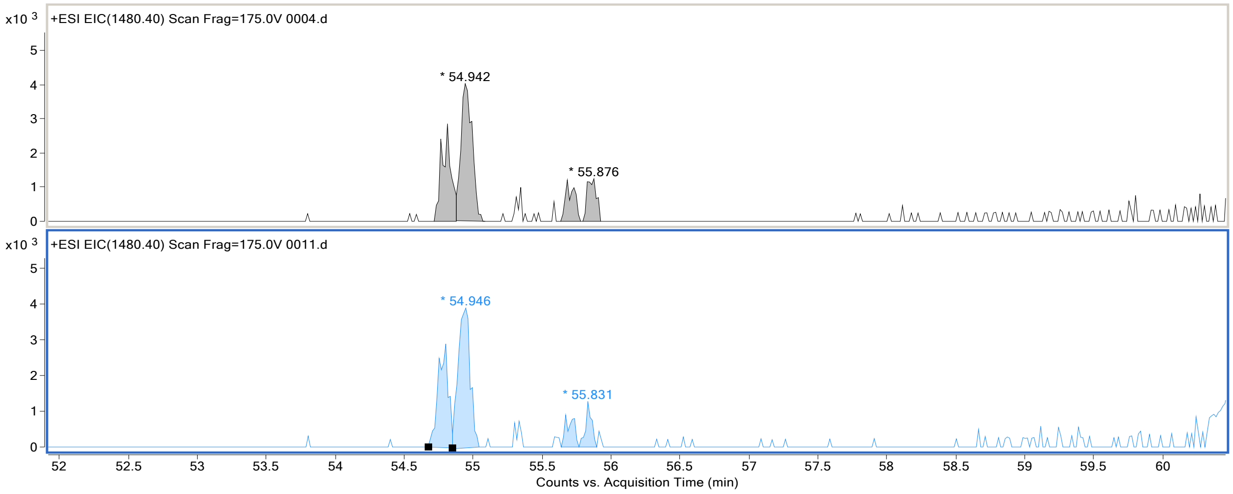


Summary of EIC areas for the identified add on Pbf byproducts

| | | |
|---|---|---|
| 1 | 54,80 | 14986,98 |
| 2 | 54,95 | 23916,09 |
| 3 | 55,67 | 3872,38 |
| 4 | 55,83 | 3895,57 |
| Σ | | **46671,02** |

### 6.2.9 EIC-MS analysis of the content of add on Pbf (+252 Da) byproducts in the crude exenatide from the cleavage using 4-MOBM as scavenger

EIC-MS peaks identified as add on Pbf byproducts: upper EIC-MS using DTT in the cleavage, lower EIC-MS using 4-MOBM in the cleavage

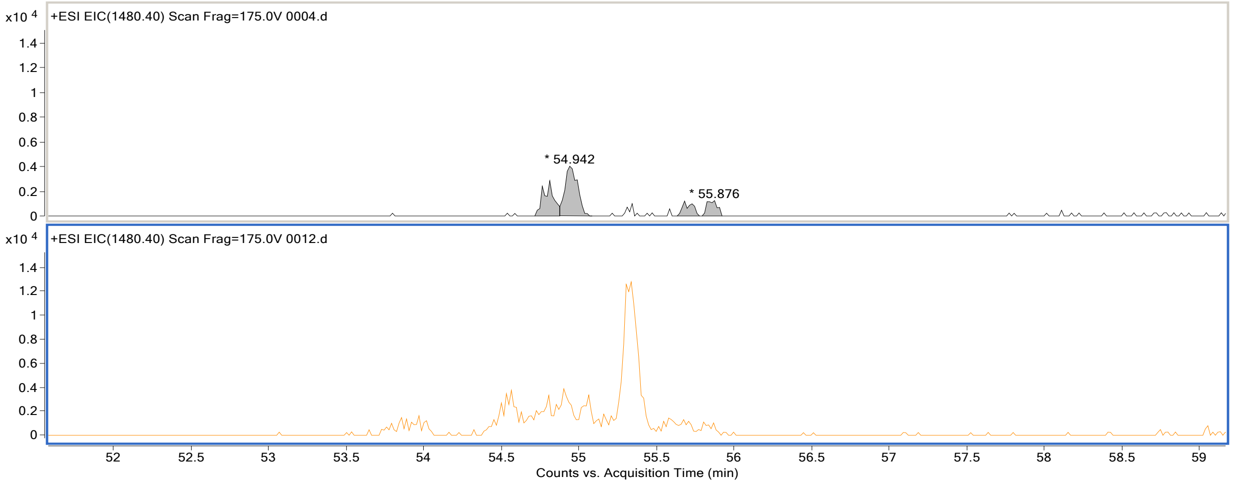


Summary of EIC areas for the identified add on Pbf byproducts

No matches for Pbf in the EIC-MS using 4-MOBM in the cleavage

### 6.2.10 EIC-MS analysis of the content of add on Pbf (+252 Da) byproducts in the crude exenatide from the cleavage using TPMT as scavenger

EIC-MS peaks identified as add on Pbf byproducts: upper EIC-MS using DTT in the cleavage, lower EIC-MS using TPMT in the cleavage

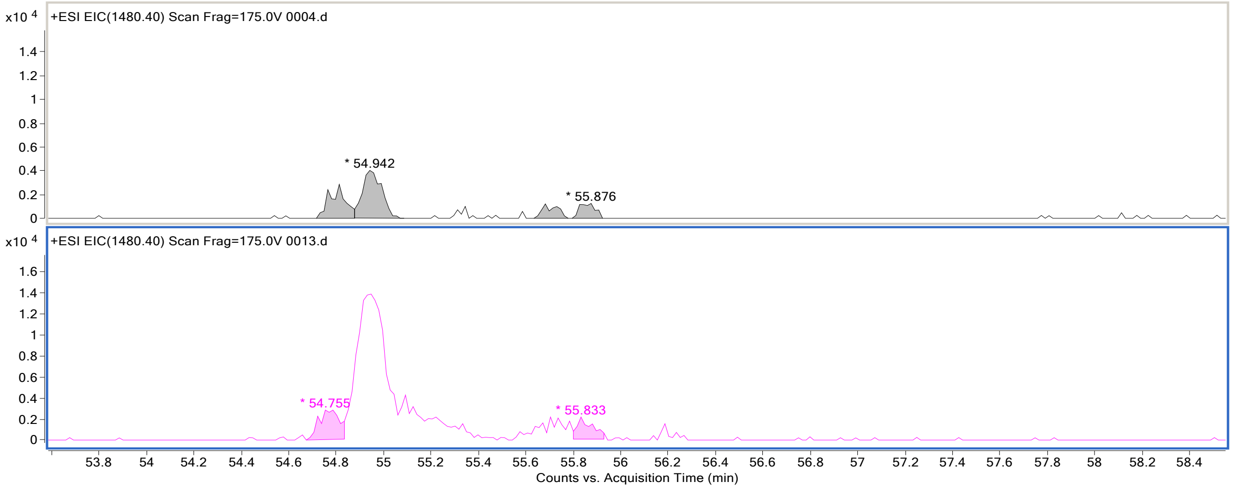


Summary of EIC areas for the identified add on Pbf byproducts

| Peak | Rt | EIC area |
|---|---|---|
| 1 | 54,75 | 17830,95 |
| 2 | 55,83 | 9307,29 |
| ∑ | | **27138,24** |

**6.2.11 EIC-MS analysis of the content of add on Pbf (+252 Da) byproducts in the crude exenatide from the cleavage using 2,4-DMOT as scavenger**

EIC-MS peaks identified as add on Pbf byproducts: upper EIC-MS using DTT in the cleavage, lower EIC-MS using 2,4-DMOT in the cleavage

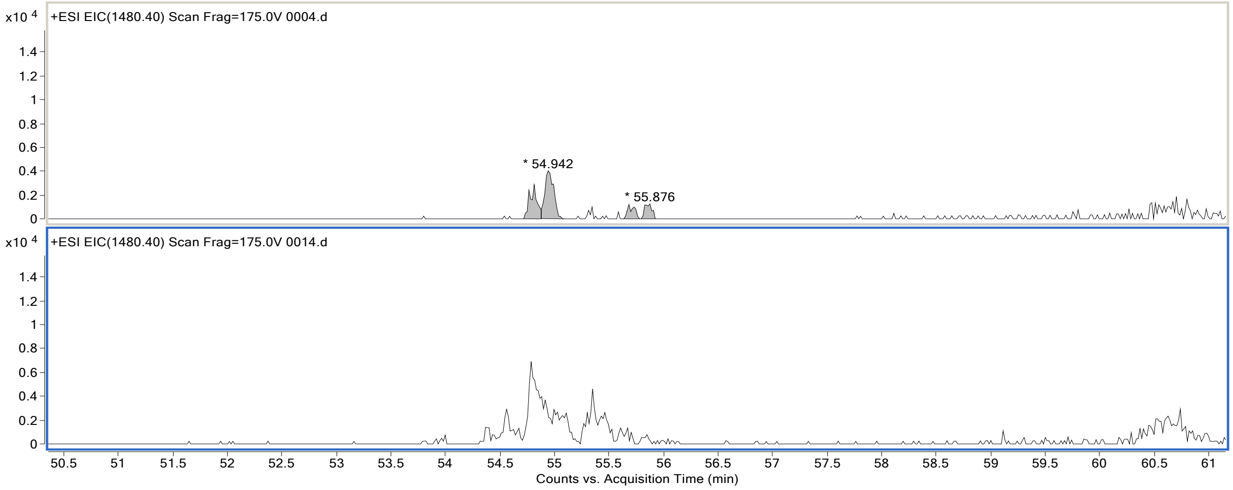


Summary of EIC areas for the identified add on Pbf byproducts

No matches for Pbf in the EIC-MS using 2,4-DMOT in the cleavage

### 6.2.12 EIC-MS analysis of the content of add on Pbf (+252 Da) byproducts in the crude exenatide from the cleavage not using a thiol scavenger

EIC-MS peaks identified as add on Pbf byproducts: upper EIC-MS using DTT in the cleavage, lower EIC-MS not using a thiol in the cleavage

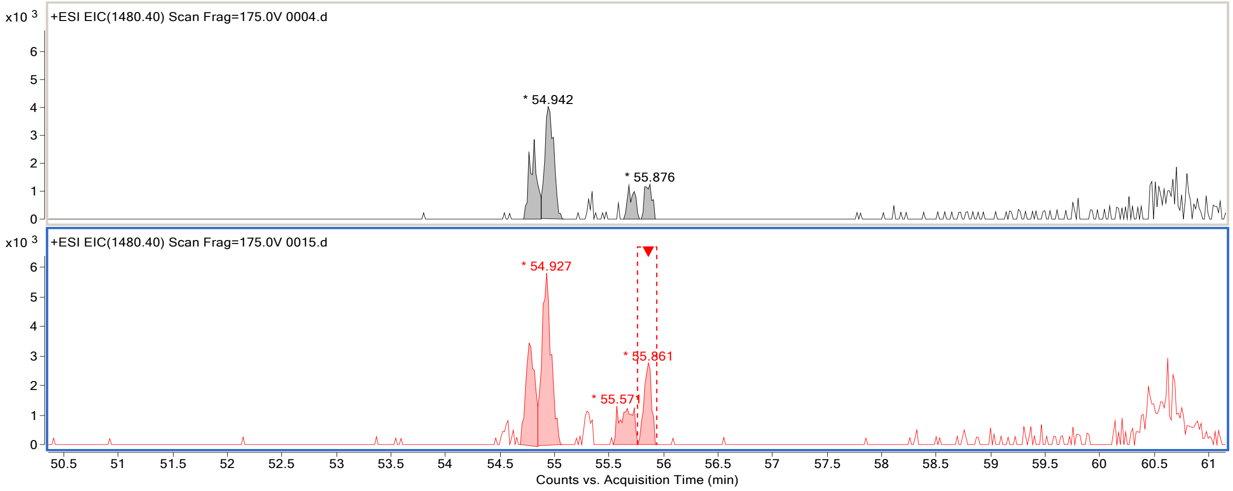


Summary of EIC areas for the identified add on Pbf byproducts

| Peak | Rt | EIC area |
|---|---|---|
| 1 | 54,77 | 20892,33 |
| 2 | 54,93 | 33926,52 |
| 3 | 55,57 | 11571,86 |
| 4 | 55,86 | 13448,82 |
| $\Sigma$ | | **79839,53** |

### 6.3 EIC-MS analysis of the content of add on $SO_3$ (+80 Da) byproducts

The original chromatograms were inspected at the m/z values corresponding to the add on $SO_3$ impurities: 4265,0 (z=+1) and the most abundant 1423,0 (z=+3).

#### 6.3.1 EIC-MS analysis of the content of add on $SO_3$ (+80 Da) byproducts in the crude exenatide from the cleavage using DTT as scavenger

EIC-MS peaks identified as add on $SO_3$ byproducts

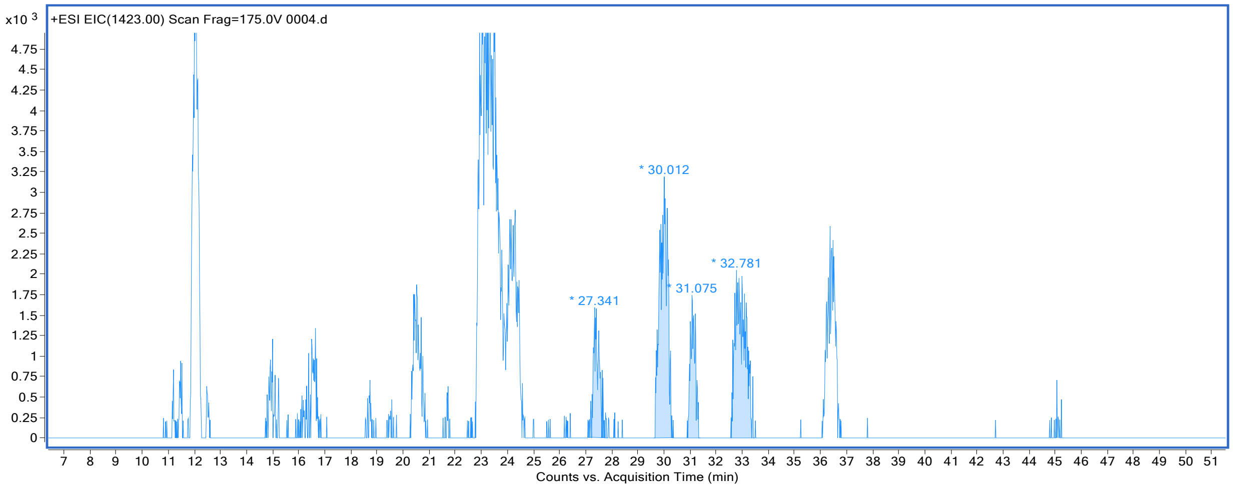


Summary of EIC areas for the identified add on $SO_3$ byproducts

| Peak | Rt | EIC area |
|---|---|---|
| 1 | 27,34 | 24414,47 |
| 2 | 30,01 | 65761,19 |
| 3 | 31,08 | 22507,87 |
| 4 | 32,78 | 56517,00 |
| ∑ | | **169200,53** |

### 6.3.2 EIC-MS analysis of the content of add $SO_3$ (+80 Da) byproducts in the crude exenatide from the cleavage using EDT as scavenger

EIC-MS peaks identified as add on $SO_3$ byproducts: upper EIC-MS using DTT in the cleavage, lower EIC-MS using EDT in the cleavage

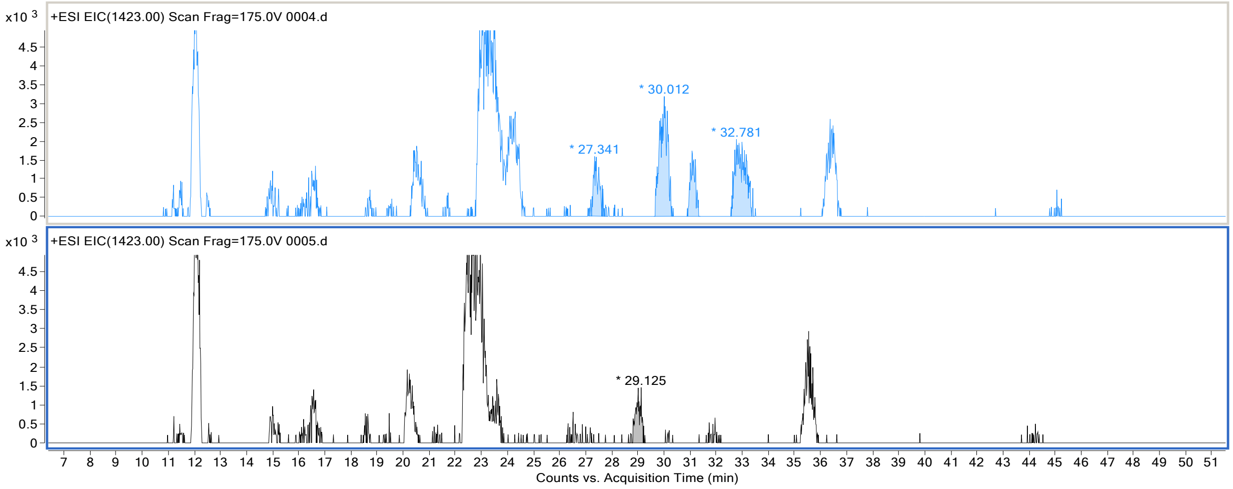


Summary of EIC areas for the identified add on $SO_3$ byproducts

| Peak | Rt | EIC area |
|---|---|---|
| 1 | 29,13 | 20629,38 |
| Σ | | **20629,38** |

### 6.3.3 EIC-MS analysis of the content of add on $SO_3$ (+80 Da) byproducts in the crude exenatide from the cleavage using DODT as scavenger

EIC-MS peaks identified as add on $SO_3$ byproducts: upper EIC-MS using DTT in the cleavage, lower EIC-MS using DODT in the cleavage

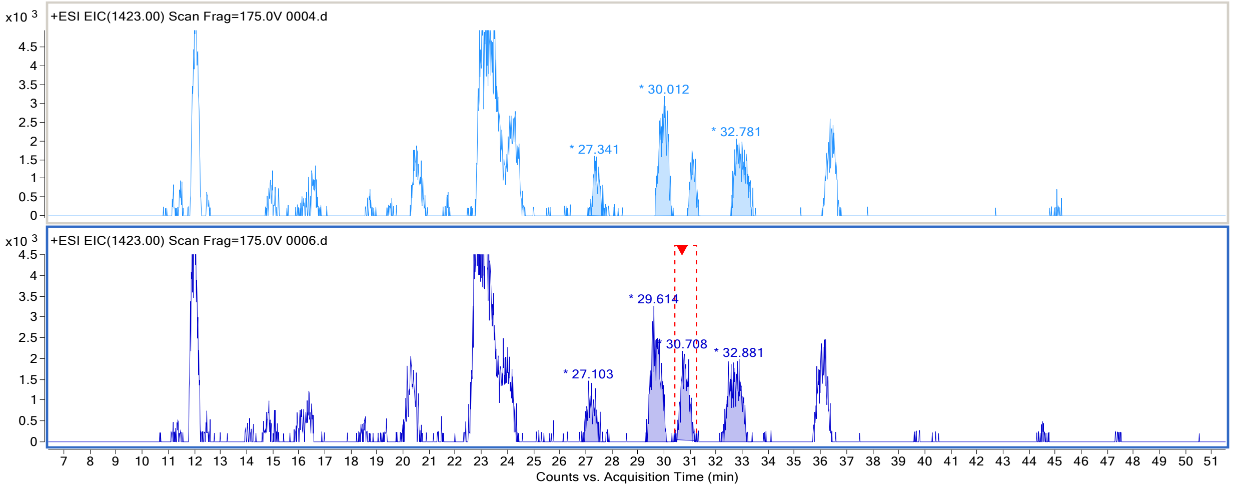


Summary of EIC areas for the identified add on $SO_3$ byproducts

| Peak | Rt | EIC area |
|---|---|---|
| 1 | 27,10 | 26629,30 |
| 2 | 29,61 | 70278,99 |
| 3 | 30,71 | 42419,04 |
| 4 | 32,88 | 58684,68 |
| $\Sigma$ | | **198012,01** |

**6.3.4 EIC-MS analysis of the content of add on $SO_3$ (+80 Da) byproducts in the crude exenatide from the cleavage using 1,4-BDMT as scavenger**

EIC-MS peaks identified as add on $SO_3$ byproducts: upper EIC-MS using DTT in the cleavage, lower EIC-MS using 1,4-BDMT in the cleavage

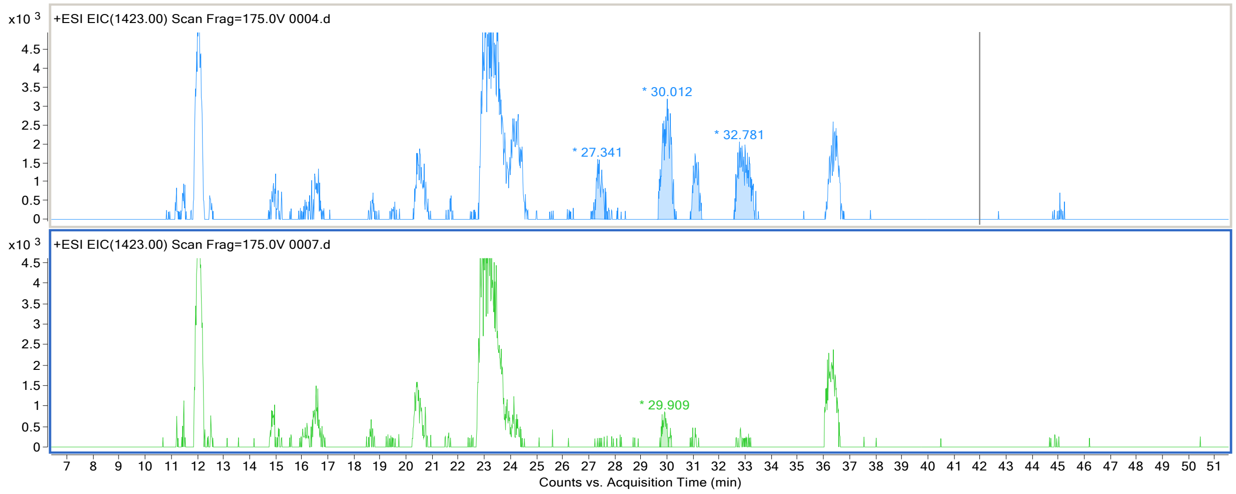


Summary of EIC areas for the identified add on $SO_3$ byproducts

| Peak | Rt | EIC area |
|---|---|---|
| 1 | 29,91 | 11120,22 |
| 2 | 30,99 | 2124,05 |
| 3 | 32,82 | 4176,27 |
| | | |
| ∑ | | **17420,54** |

### 6.3.5 EIC-MS analysis of the content of add on $SO_3$ (+80 Da) byproducts in the crude exenatide from the cleavage using 1,3-BDMT as scavenger

EIC-MS peaks identified as add on $SO_3$ byproducts: upper EIC-MS using DTT in the cleavage, lower EIC-MS using 1,3-BDMT in the cleavage

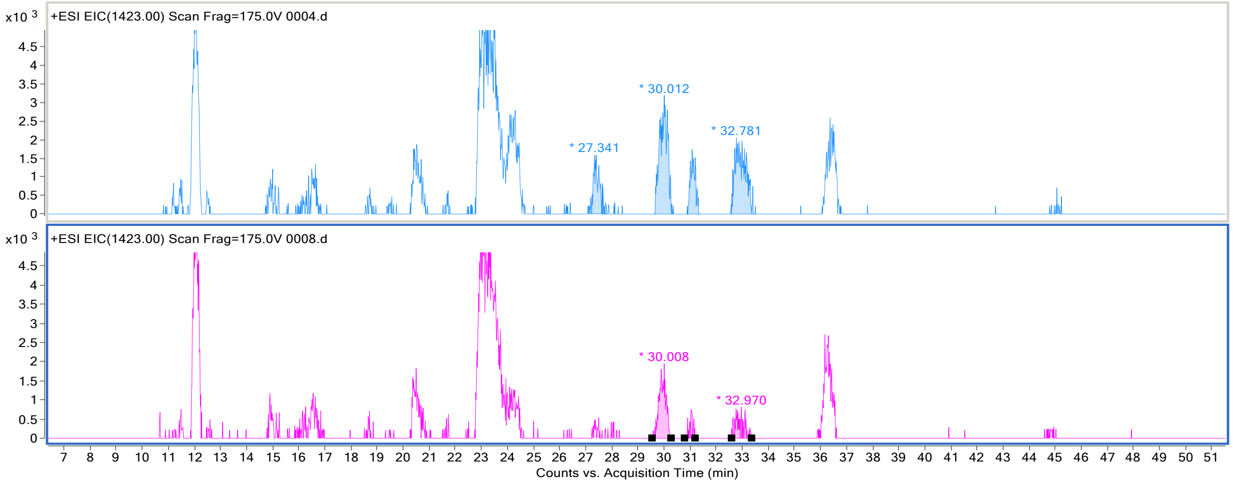


Summary of EIC areas for the identified add on $SO_3$ byproducts

| Peak | Rt | EIC area |
|---|---|---|
| 1 | 30,01 | 31872,30 |
| 2 | 31,04 | 6127,68 |
| 3 | 32,97 | 13595,85 |
| | | |
| Σ | | **51595,83** |

### 6.3.6 EIC-MS analysis of the content of add on $SO_3$ (+80 Da) byproducts in the crude exenatide from the cleavage using 1,2-BDMT as scavenger

EIC-MS peaks identified as add on $SO_3$ byproducts: upper EIC-MS using DTT in the cleavage, lower EIC-MS using 1,2-BDMT in the cleavage

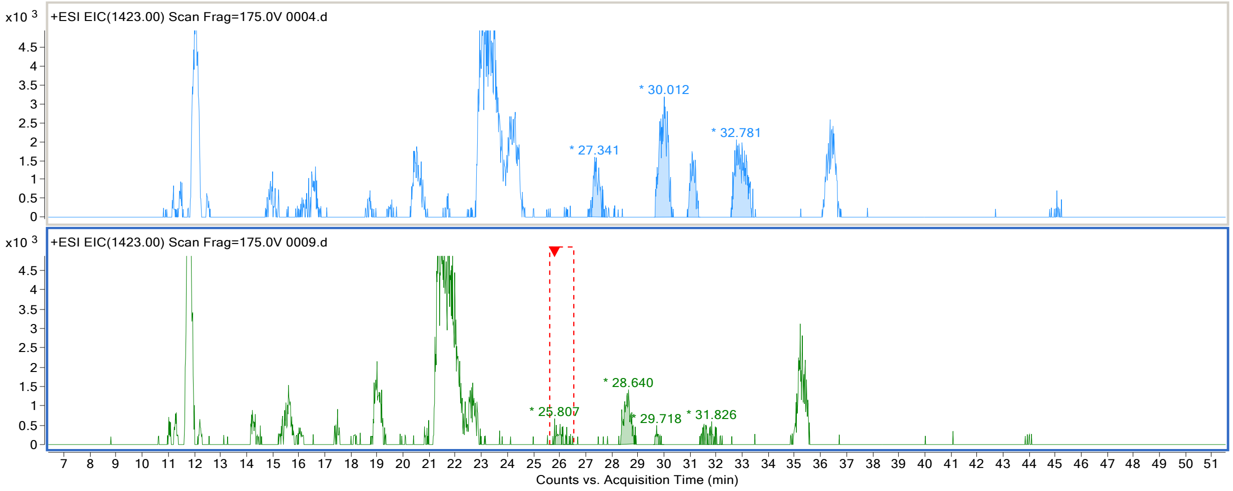


Summary of EIC areas for the identified add on $SO_3$ byproducts

| Peak | Rt | EIC area |
|---|---|---|
| 1 | 25,81 | 7777,70 |
| 2 | 28,64 | 23509,39 |
| 3 | 29,72 | 2938,27 |
| 4 | 31,83 | 9625,62 |
| ∑ | | **43850,98** |

**6.3.7 EIC-MS analysis of the content of add on $SO_3$ (+80 Da) byproducts in the crude exenatide from the cleavage using 4,4'-BMMB as scavenger**

EIC-MS peaks identified as add on $SO_3$ byproducts: upper EIC-MS using DTT in the cleavage, lower EIC-MS using 4,4'-BMMB in the cleavage

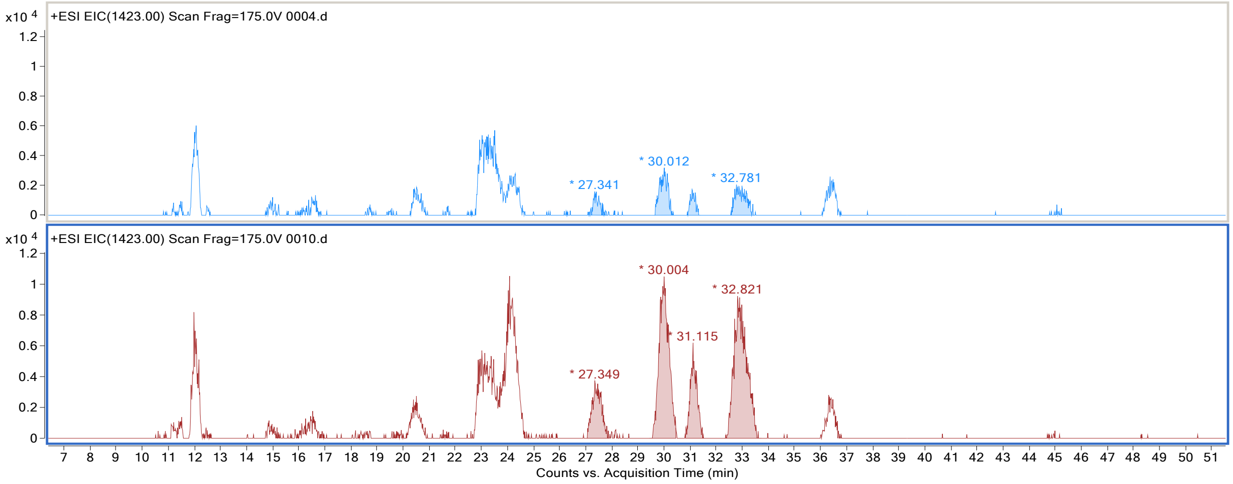


Summary of EIC areas for the identified add on $SO_3$ byproducts

| **Peak** | **Rt** | **EIC area** |
|---|---|---|
| 1 | 27,35 | 88931,32 |
| 2 | 30,00 | 271409,88 |
| 3 | 31,12 | 100601,03 |
| 4 | 32,82 | 305885,63 |
| $\Sigma$ | | **766827,86** |

**6.3.8 EIC-MS analysis of the content of add on $SO_3$ (+80 Da) byproducts in the crude exenatide from the cleavage using 2,4-DCBM as scavenger**

EIC-MS peaks identified as add on $SO_3$ byproducts: upper EIC-MS using DTT in the cleavage, lower EIC-MS using 2,4-DCBM in the cleavage

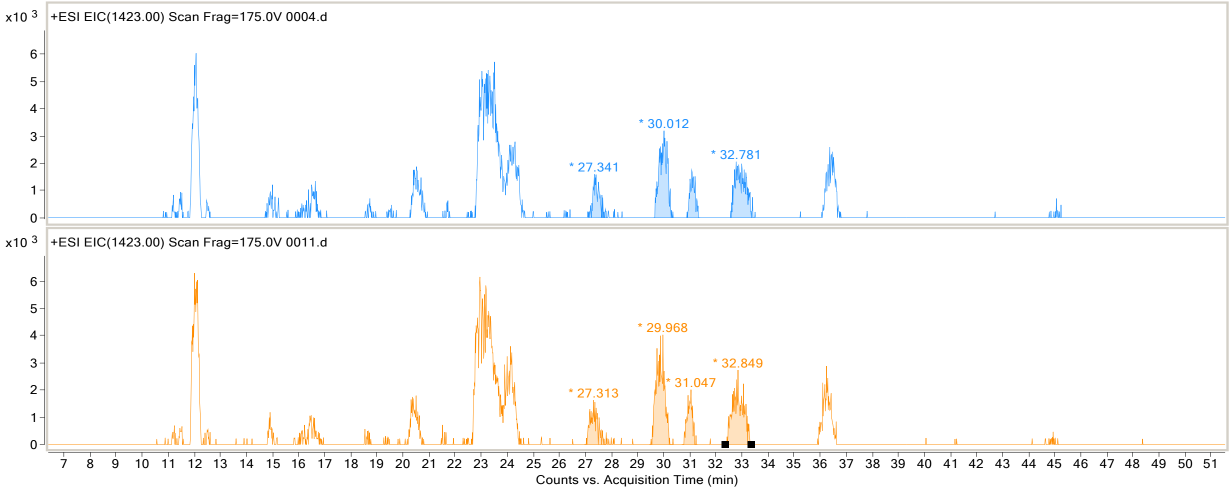


Summary of EIC areas for the identified add on $SO_3$ byproducts

| Peak | Rt | EIC area |
|---|---|---|
| 1 | 27,31 | 28636,10 |
| 2 | 29,97 | 77878,09 |
| 3 | 31,05 | 25959,94 |
| 4 | 32,85 | 63866,84 |
| $\sum$ | | **196340,97** |

**6.3.9 EIC-MS analysis of the content of add on $SO_3$ (+80 Da) byproducts in the crude exenatide from the cleavage using 4-MOBM as scavenger**

EIC-MS peaks identified as add on $SO_3$ byproducts: upper EIC-MS using DTT in the cleavage, lower EIC-MS using 4-MOBM in the cleavage

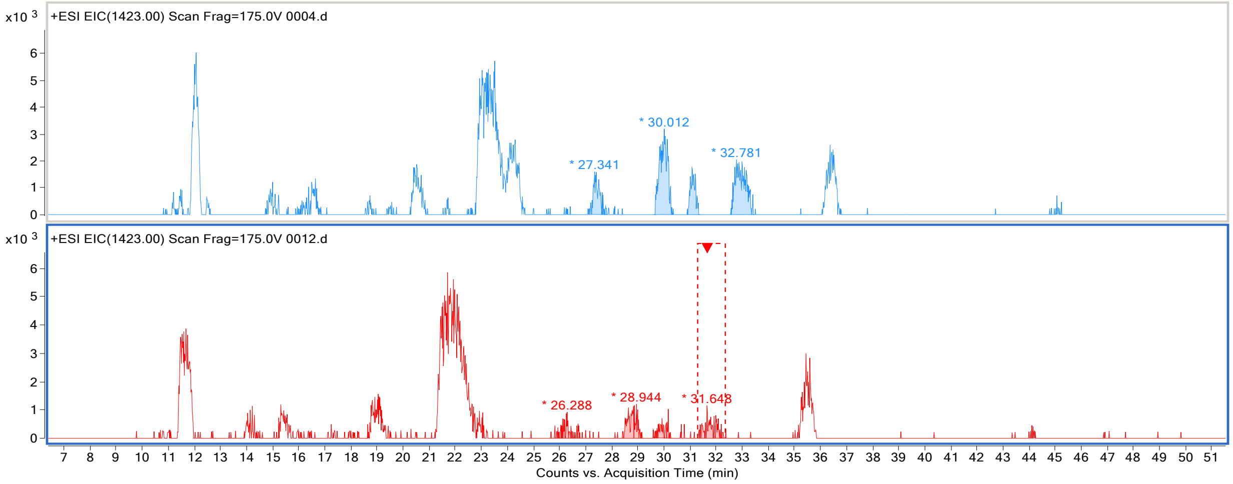


Summary of EIC areas for the identified add on $SO_3$ byproducts

| Peak | Rt | EIC area |
|---|---|---|
| 1 | 26,29 | 13583,72 |
| 2 | 28,94 | 23194,92 |
| 3 | 30,17 | 10514,33 |
| 4 | 31,65 | 20244,96 |
| $\sum$ | | **67537,93** |

### 6.3.10 EIC-MS analysis of the content of add on $SO_3$ (+80 Da) byproducts in the crude exenatide from the cleavage using TPMT as scavenger

EIC-MS peaks identified as add on $SO_3$ byproducts: upper EIC-MS using DTT in the cleavage, lower EIC-MS using TPMT in the cleavage

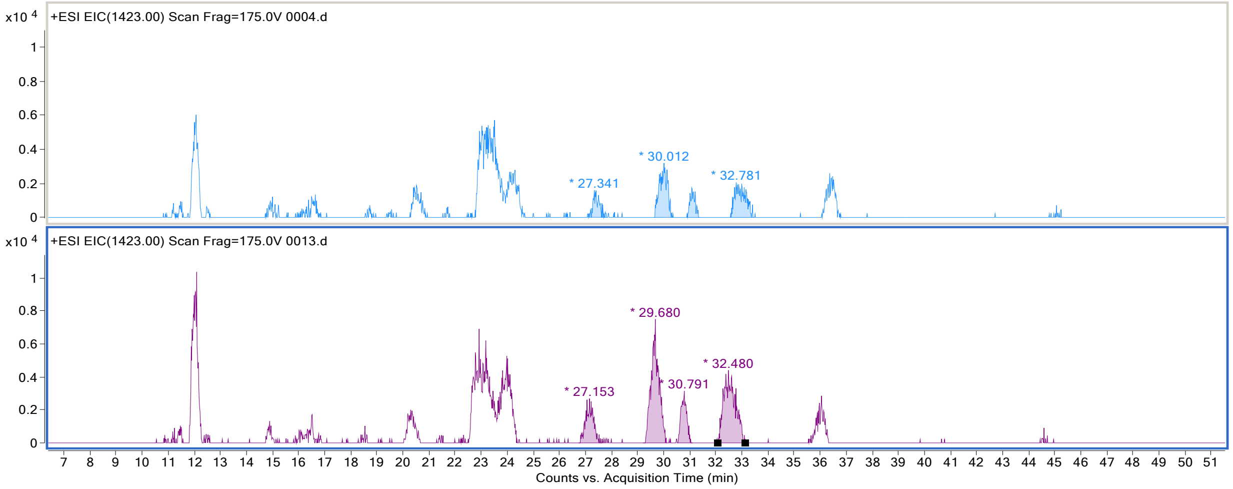


Summary of EIC areas for the identified add on $SO_3$ byproducts

| Peak | Rt | EIC area |
|---|---|---|
| 1 | 27,15 | 51136,35 |
| 2 | 29,68 | 146709,92 |
| 3 | 30,791 | 45334,77 |
| 4 | 32,48 | 132274,11 |
| ∑ | | **375455,15** |

### 6.3.11 EIC-MS analysis of the content of add on $SO_3$ (+80 Da) byproducts in the crude exenatide from the cleavage using 2,4-DMOT as scavenger

EIC-MS peaks identified as add on $SO_3$ byproducts: upper EIC-MS using DTT in the cleavage, lower EIC-MS using 2,4-DMOT in the cleavage

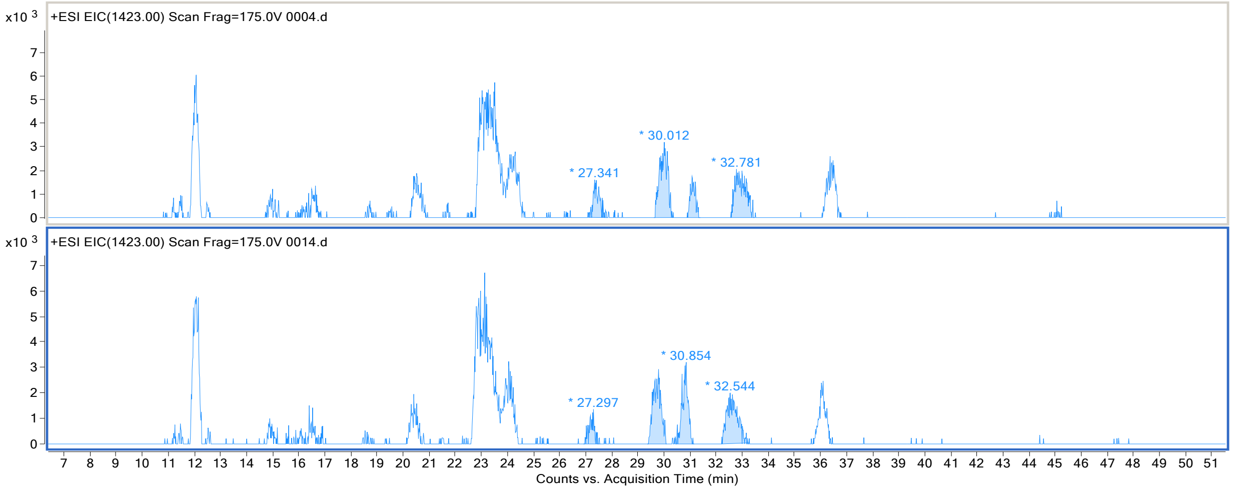


Summary of EIC areas for the identified add on $SO_3$ byproducts

| Peak | Rt | EIC area |
|---|---|---|
| 1 | 27,30 | 16544,60 |
| 2 | 29,79 | 57896,31 |
| 3 | 30,85 | 47491,91 |
| 4 | 32,54 | 54859,30 |
| $\sum$ | | **176792,12** |

### 6.3.12 EIC-MS analysis of the content of add on $SO_3$ (+80 Da) byproducts in the crude exenatide from the cleavage not using a thiol scavenger

EIC-MS peaks identified as add on $SO_3$ byproducts: upper EIC-MS using DTT in the cleavage, lower EIC-MS not using a thiol in the cleavage

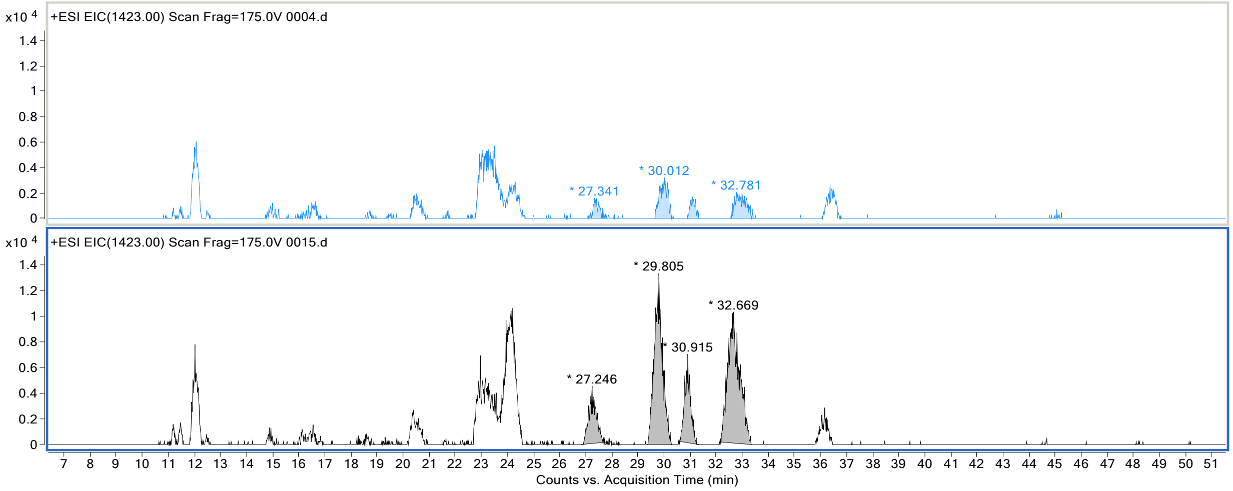


Summary of EIC areas for the identified add on $SO_3$ byproducts

| Peak | Rt | EIC area |
|---|---|---|
| 1 | 27,25 | 80849,34 |
| 2 | 29,81 | 283071,40 |
| 3 | 30,92 | 97376,28 |
| 4 | 32,67 | 314584,57 |
| ∑ | | **775881,59** |

### 6.4 EIC-MS analysis of the content of Trp ox (+16 Da) byproducts

The original chromatograms were inspected at the m/z values corresponding to the Trp ox impurities: 4201,0 (z=+1) and the most abundant 1401,7 (z=+3). Both 5-hydroxytryptophan and oxindolylalanine forms[8] of Trp +16 Da oxidants were evaluated, no attempts were made to discern which form is related to which peak in the EIC MS spectra.

#### 6.4.1 EIC-MS analysis of the content of Trp ox (+16 Da) byproducts in the crude exenatide from the cleavage using DTT as scavenger

EIC-MS peaks identified as Trp ox (+16 Da) byproducts

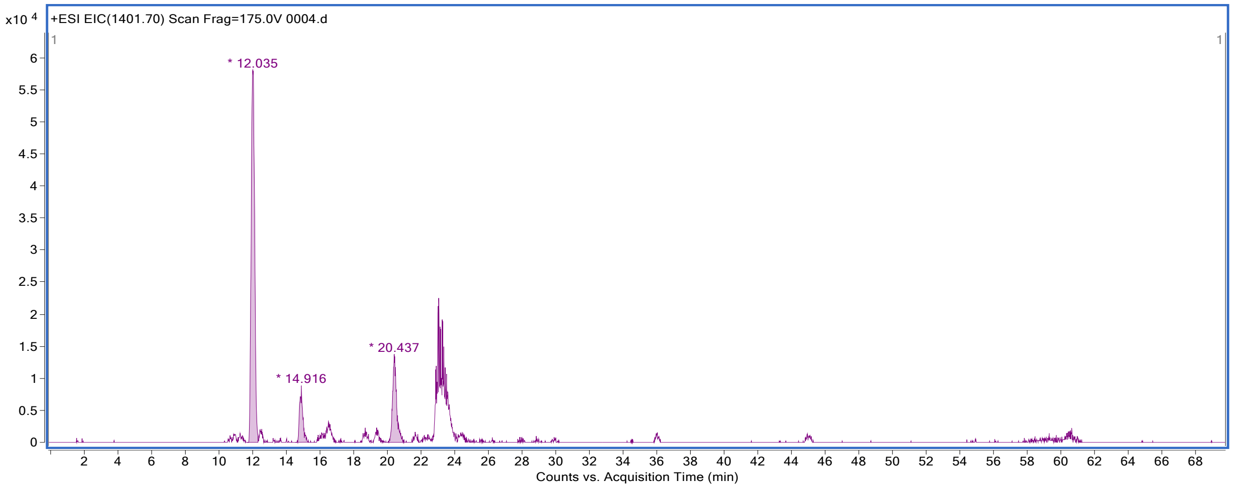


Summary of EIC areas for the identified Trp ox (+16 Da) byproducts

| Peak | Rt | EIC area |
|---|---|---|
| 1 | 14,92 | 115316,35 |
| 2 | 20,44 | 225142,79 |
| Σ | | **340459,14** |

### 6.4.2 EIC-MS analysis of the content of Trp ox (+16 Da) byproducts in the crude exenatide from the cleavage using EDT as scavenger

EIC-MS peaks identified as Trp ox (+16 Da) byproducts: upper EIC-MS using DTT in the cleavage, lower EIC-MS using EDT in the cleavage

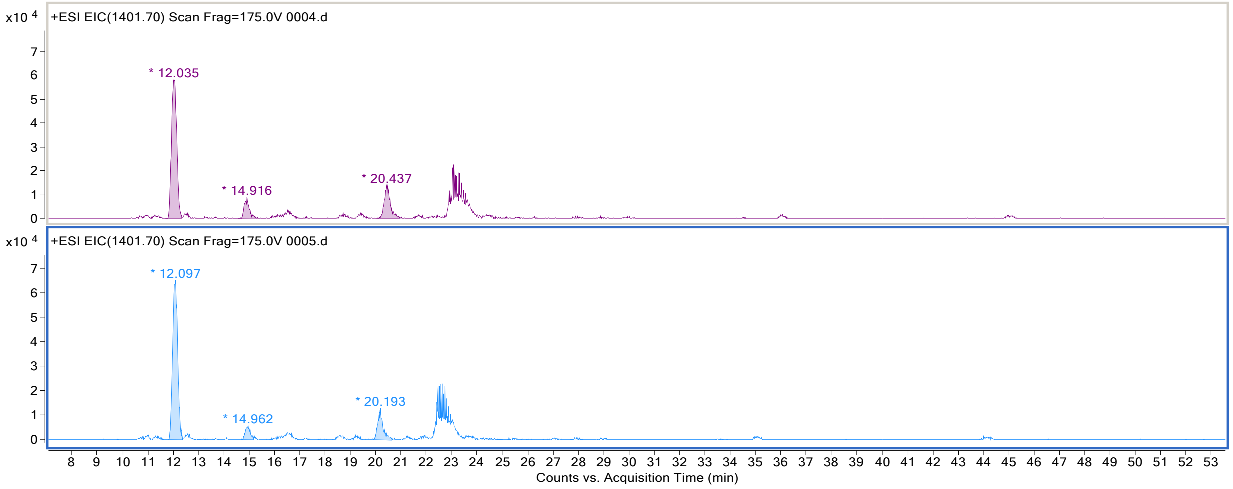


Summary of EIC areas for the identified Trp ox (+16 Da) byproducts

| Peak | Rt | EIC area |
|---|---|---|
| 1 | 14,96 | 76562,78 |
| 2 | 20,19 | 178311,31 |
| $\sum$ | | **254874,09** |

### 6.4.3 EIC-MS analysis of the content of Trp ox (+16 Da) byproducts in the crude exenatide from the cleavage using DODT as scavenger

EIC-MS peaks identified as Trp ox (+16 Da) byproducts: upper EIC-MS using DTT in the cleavage, lower EIC-MS using DODT in the cleavage

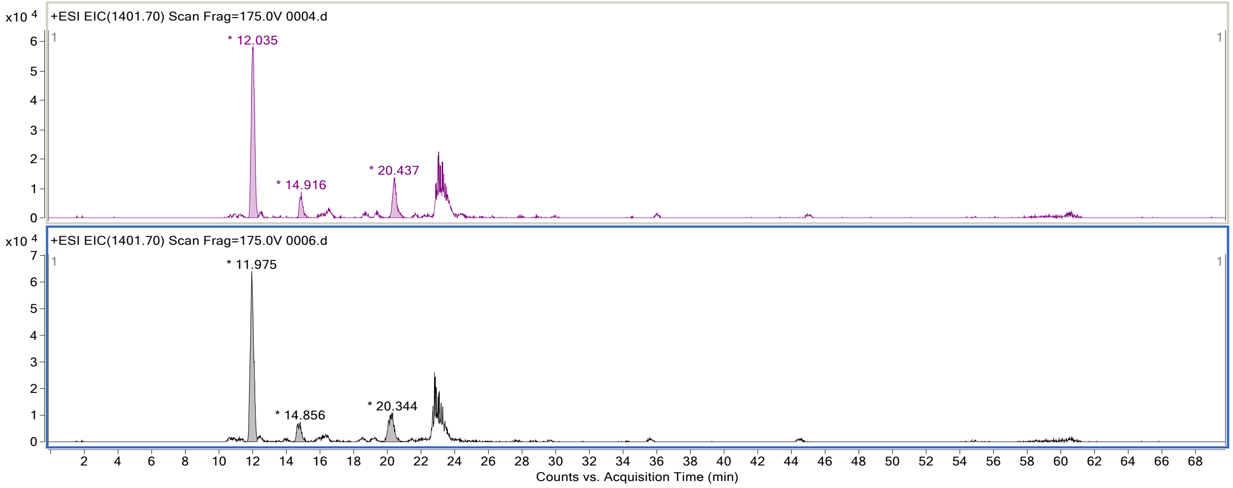


Summary of EIC areas for the identified Trp ox (+16 Da) byproducts

| Peak | Rt | EIC area |
|---|---|---|
| 2 | 14,86 | 134955,50 |
| 3 | 20,34 | 254463,66 |
| ∑ | | **389419,16** |

### 6.4.4 EIC-MS analysis of the content of Trp ox (+16 Da) byproducts in the crude exenatide from the cleavage using 1,4-BDMT as scavenger

EIC-MS peaks identified as Trp ox (+16 Da) byproducts: upper EIC-MS using DTT in the cleavage, lower EIC-MS using 1,4-BDMT in the cleavage

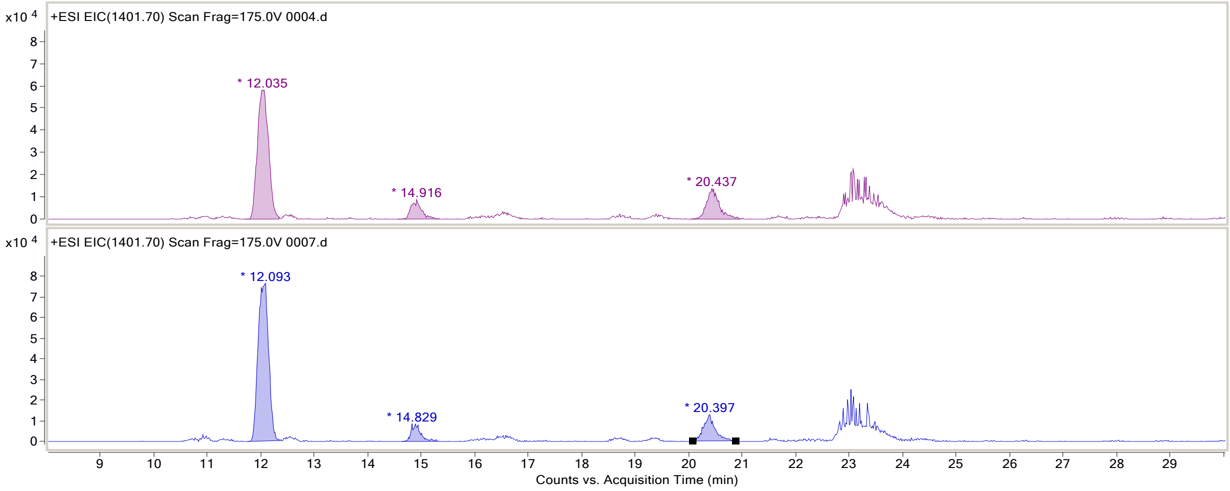


Summary of EIC areas for the identified Trp ox (+16 Da) byproducts

| Peak | Rt | EIC area |
|---|---|---|
| 1 | 14,83 | 104495,31 |
| 2 | 20,40 | 186430,47 |
| | | |
| Σ | | **290925,78** |

### 6.4.5 EIC-MS analysis of the content of Trp ox (+16 Da) byproducts in the crude exenatide from the cleavage using 1,3-BDMT as scavenger

EIC-MS peaks identified as Trp ox (+16 Da) byproducts: upper EIC-MS using DTT in the cleavage, lower EIC-MS using 1,3-BDMT in the cleavage

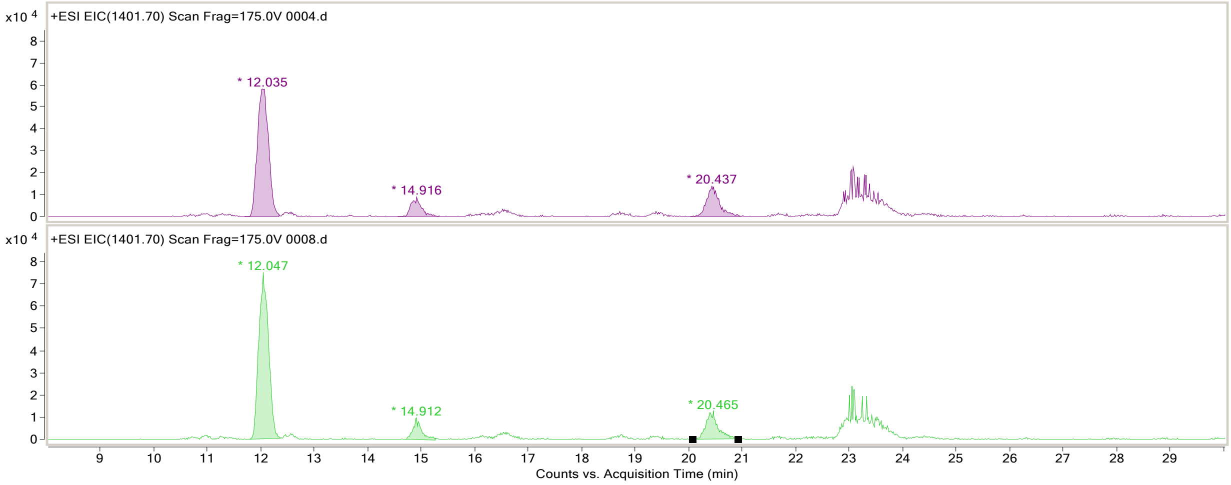


Summary of EIC areas for the identified Trp ox (+16 Da) byproducts

| Peak | Rt | EIC area |
|---|---|---|
| 2 | 14,91 | 107039,64 |
| 3 | 20,46 | 191283,37 |
| | | |
| Σ | | **298323,01** |

### 6.4.6 EIC-MS analysis of the content of Trp ox (+16 Da) byproducts in the crude exenatide from the cleavage using 1,2-BDMT as scavenger

EIC-MS peaks identified as Trp ox (+16 Da) byproducts: upper EIC-MS using DTT in the cleavage, lower EIC-MS using 1,2-BDMT in the cleavage

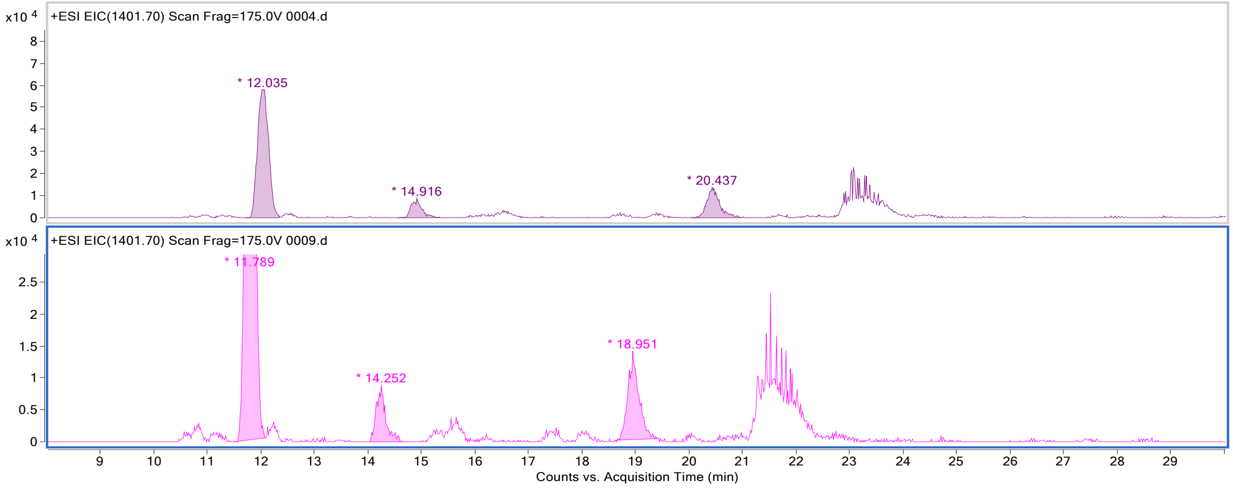


Summary of EIC areas for the identified Trp ox (+16 Da) byproducts

| Peak | Rt | EIC area |
|---|---|---|
| 1 | 14,25 | 106006,38 |
| 2 | 18,95 | 193503,57 |
| | | |
| Σ | | **299509,95** |

### 6.4.7 EIC-MS analysis of the content of Trp ox (+16 Da) byproducts in the crude exenatide from the cleavage using 4,4'-BMMB as scavenger

EIC-MS peaks identified as Trp ox (+16 Da) byproducts: upper EIC-MS using DTT in the cleavage, lower EIC-MS using 4,4'-BMMB in the cleavage

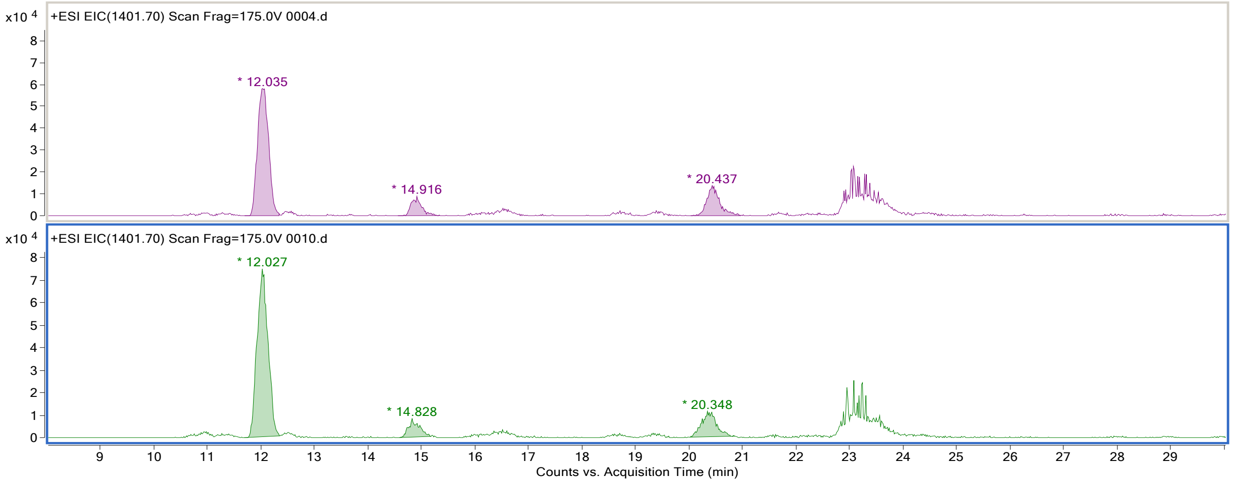


Summary of EIC areas for the identified Trp ox (+16 Da) byproducts

| Peak | Rt | EIC area |
|---|---|---|
| 1 | 14,83 | 111611,73 |
| 2 | 20,35 | 212884,78 |
| | | |
| Σ | | **324496,51** |

### 6.4.8 EIC-MS analysis of the content of Trp ox (+16 Da) byproducts in the crude exenatide from the cleavage using 2,4-DCBM as scavenger

EIC-MS peaks identified as Trp ox (+16 Da) byproducts: upper EIC-MS using DTT in the cleavage, lower EIC-MS using 2,4-DCBM in the cleavage

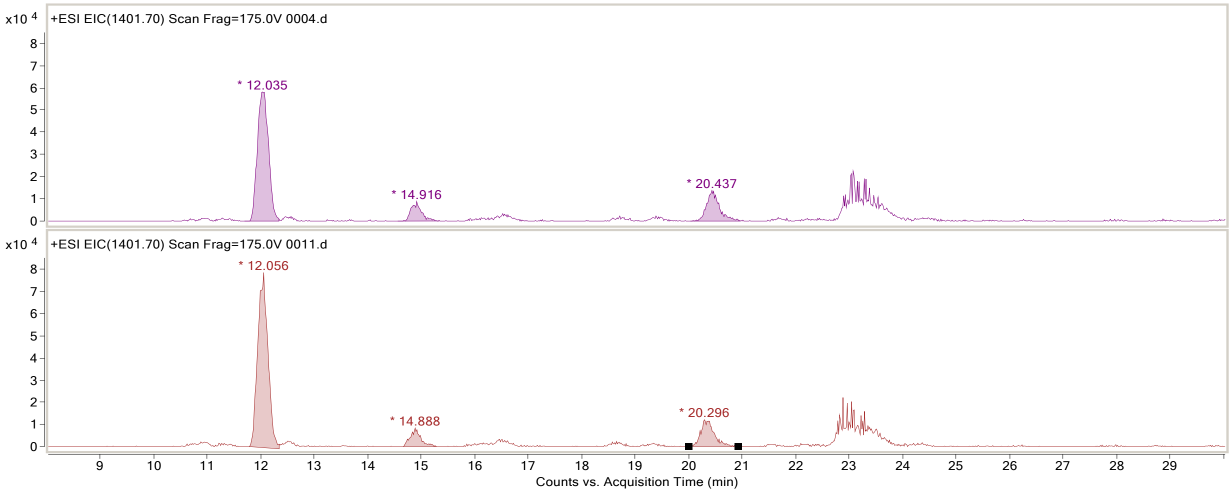


Summary of EIC areas for the identified Trp ox (+16 Da) byproducts

| Peak | Rt | EIC area |
|---|---|---|
| 1 | 14,89 | 109839,85 |
| 2 | 20,30 | 209197,29 |
| | | |
| ∑ | | **319037,14** |

### 6.4.9 EIC-MS analysis of the content of Trp ox (+16 Da) byproducts in the crude exenatide from the cleavage using 4-MOBM as scavenger

EIC-MS peaks identified as Trp ox (+16 Da) byproducts: upper EIC-MS using DTT in the cleavage, lower EIC-MS using 4-MOBM in the cleavage

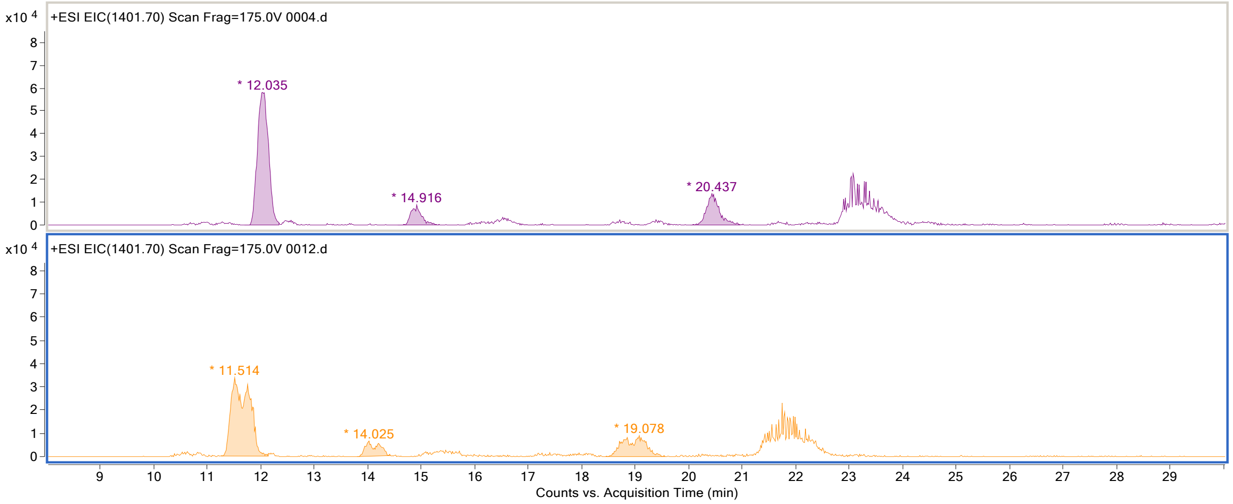


Summary of EIC areas for the identified Trp ox (+16 Da) byproducts

| **Peak** | **Rt** | **EIC area** |
|---|---|---|
| 1 | 14,03 | 111557,04 |
| 2 | 19,08 | 258321,82 |
| | | |
| ∑ | | **369878,86** |

### 6.4.10 EIC-MS analysis of the content of Trp ox (+16 Da) byproducts in the crude exenatide from the cleavage using TPMT as scavenger

EIC-MS peaks identified as Trp ox (+16 Da) byproducts: upper EIC-MS using DTT in the cleavage, lower EIC-MS using TPMT in the cleavage

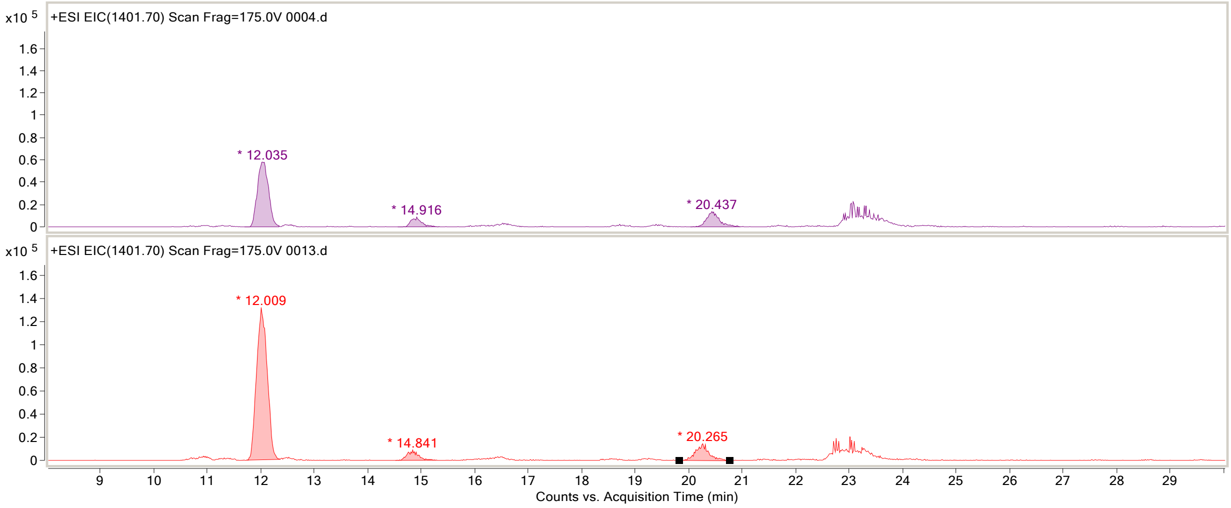


Summary of EIC areas for the identified Trp ox (+16 Da) byproducts

| Peak | Rt | EIC area |
|---|---|---|
| 1 | 14,84 | 118491,16 |
| 2 | 20,265 | 245113,79 |
| | | |
| Σ | | **363604,95** |

### 6.4.11 EIC-MS analysis of the content of Trp ox (+16 Da) byproducts in the crude exenatide from the cleavage using 2,4-DMOT as scavenger

EIC-MS peaks identified as Trp ox (+16 Da) byproducts: upper EIC-MS using DTT in the cleavage, lower EIC-MS using 2,4-DMOT in the cleavage

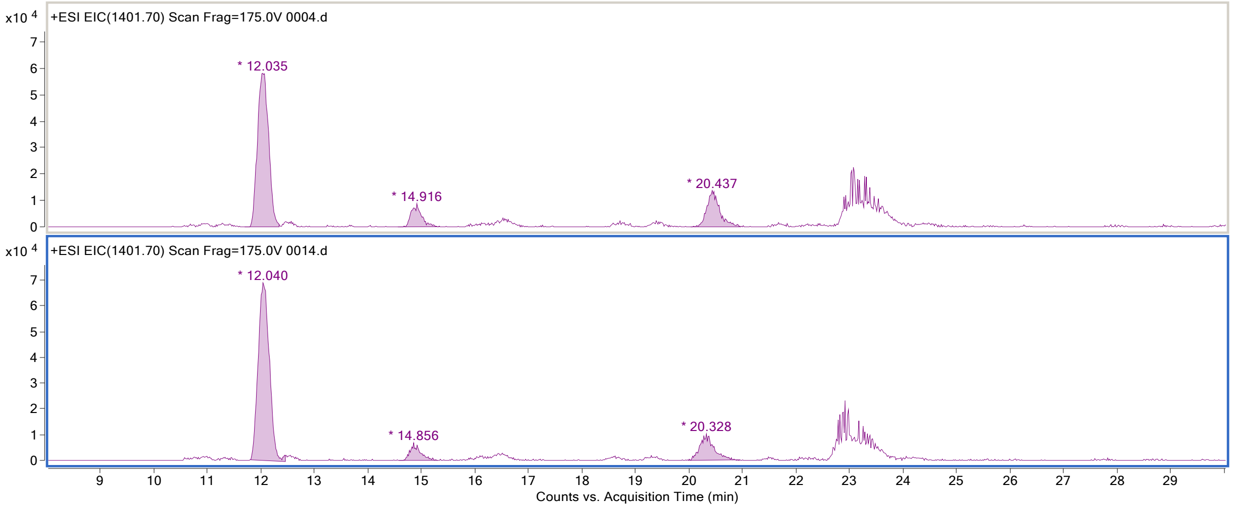


Summary of EIC areas for the identified Trp ox (+16 Da) byproducts

| Peak | Rt | EIC area |
|---|---|---|
| 1 | 14,86 | 85765,16 |
| 2 | 20,33 | 179692,21 |
| Σ | | **265457,37** |

### 6.4.12 EIC-MS analysis of the content of Trp ox (+16 Da) byproducts in the crude exenatide from the cleavage not using a thiol scavenger

EIC-MS peaks identified as Trp ox (+16 Da) byproducts: upper EIC-MS using DTT in the cleavage, lower EIC-MS not using a thiol in the cleavage

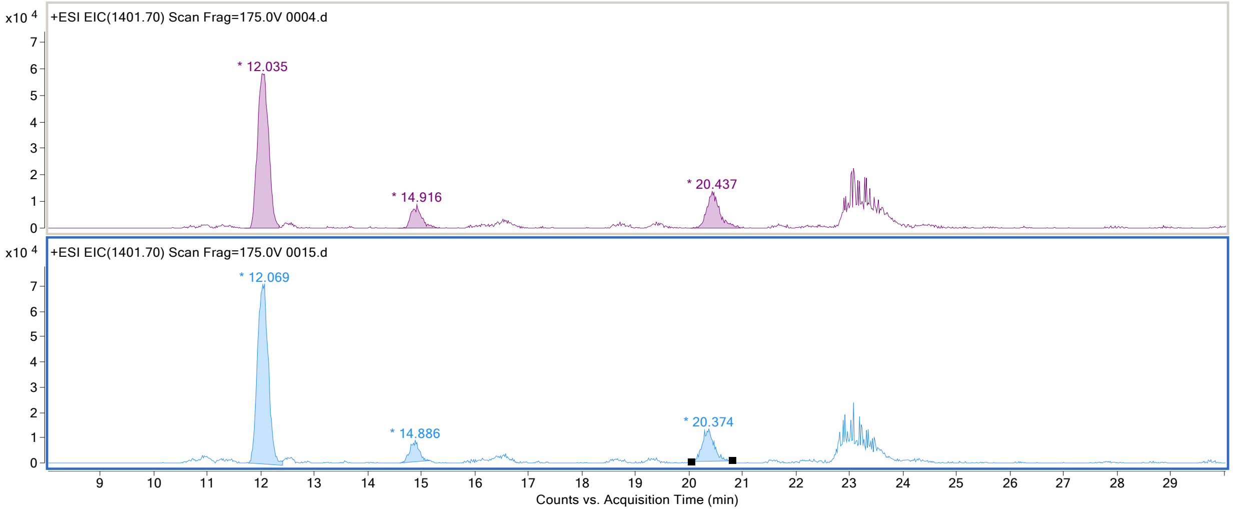


Summary of EIC areas for the identified Trp ox (+16 Da) byproducts

| Peak | Rt | EIC area |
|---|---|---|
| 1 | 14,89 | 99698,30 |
| 2 | 20,37 | 188972,13 |
| | | |
| **Σ** | | **288670,43** |

### 6.5 EIC-MS analysis of the content of Met to HCys (-14 Da) byproducts

The original chromatograms were inspected at the m/z values corresponding to the Met to HCys impurities: 4171,0 (z=+1) and the most abundant 1410,4 (z=+3).

### 6.5.1 EIC-MS analysis of the content of Met to HCys (-14 Da) byproducts in the crude exenatide from the cleavage using DTT as scavenger

EIC-MS peaks identified as Met to HCys (-14 Da) byproducts

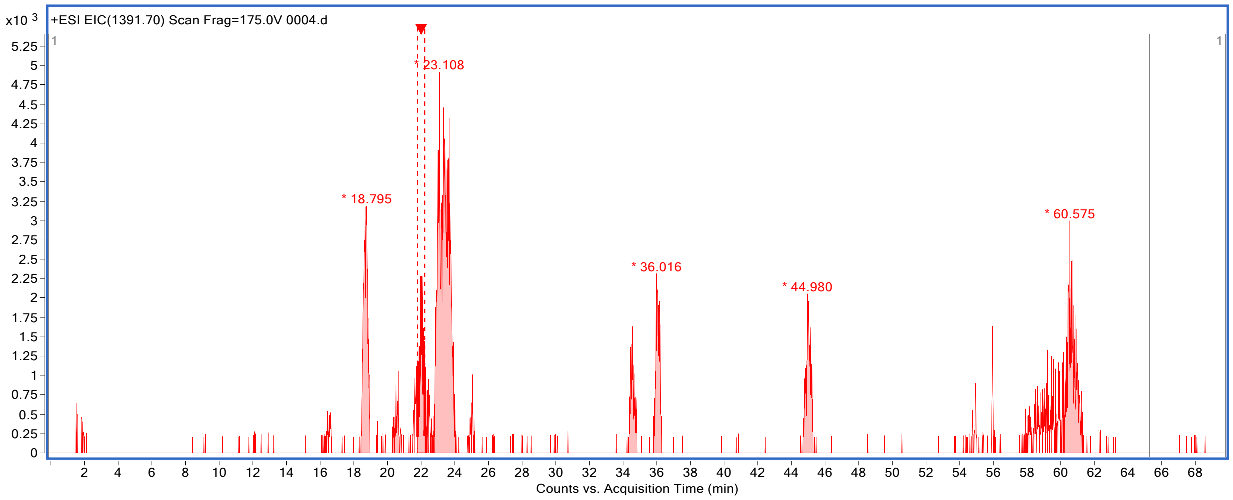


Summary of EIC areas for the identified Met to HCys (-14 Da) byproducts

| Peak | Rt | EIC area |
|---|---|---|
| 1 | 22,06 | 30282,47 |

**6.5.2 EIC-MS analysis of the content of Met to HCys (-14 Da) byproducts in the crude exenatide from the cleavage using EDT as scavenger**

EIC-MS peaks identified as Met to HCys (-14 Da) byproducts: upper EIC-MS using DTT in the cleavage, lower EIC-MS using EDT in the cleavage

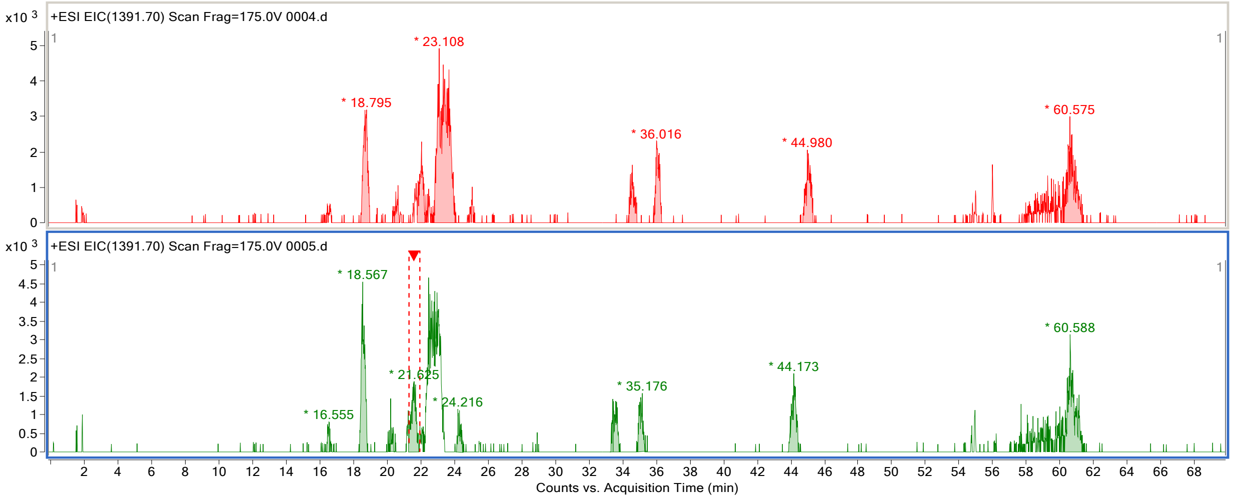


Summary of EIC areas for the identified Met to HCys (-14 Da) byproducts

| Peak | Rt | EIC area |
|---|---|---|
| 1 | 21,63 | 36498,09 |

**6.5.3 EIC-MS analysis of the content of Met to HCys (-14 Da) byproducts in the crude exenatide from the cleavage using DODT as scavenger**

EIC-MS peaks identified as Met to HCys (-14 Da) byproducts: upper EIC-MS using DTT in the cleavage, lower EIC-MS using DODT in the cleavage

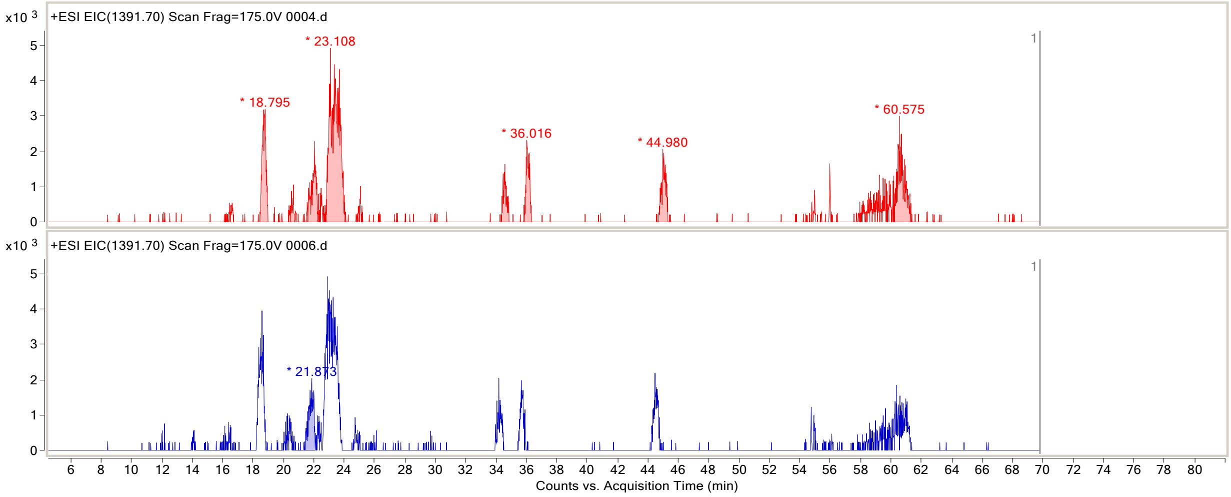


Summary of EIC areas for the identified Met to HCys (-14 Da) byproducts

| Peak | Rt | EIC area |
|---|---|---|
| 1 | 21,87 | 34914,38 |

### 6.5.4 EIC-MS analysis of the content of Met to HCys (-14 Da) byproducts in the crude exenatide from the cleavage using 1,4-BDMT as scavenger

EIC-MS peaks identified as Met to HCys (-14 Da) byproducts: upper EIC-MS using DTT in the cleavage, lower EIC-MS using 1,4-BDMT in the cleavage

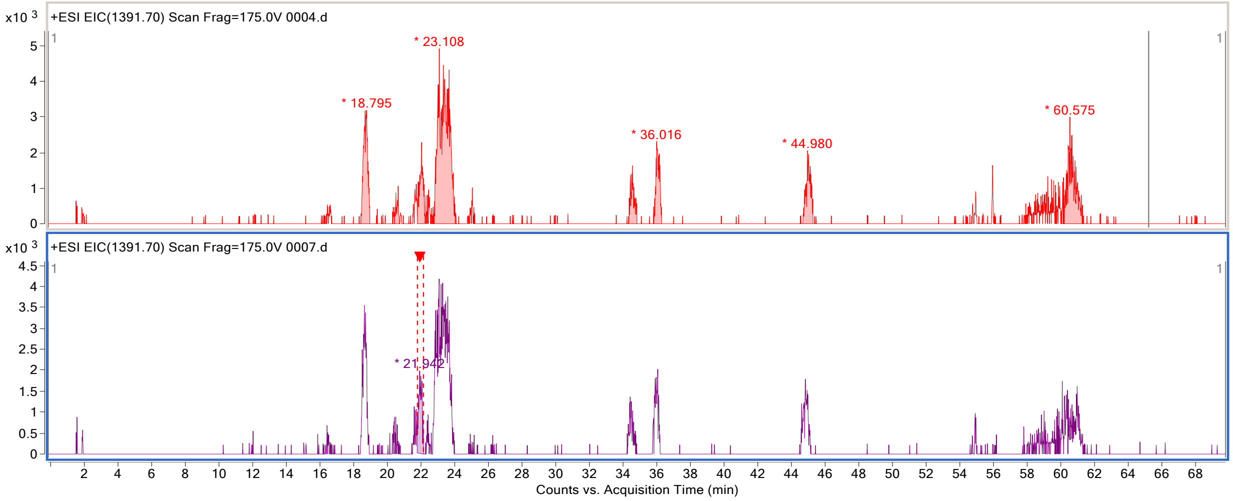


Summary of EIC areas for the identified Met to HCys (-14 Da) byproducts

| Peak | Rt | EIC area |
|---|---|---|
| 1 | 21,94 | 26909,28 |

**6.5.5 EIC-MS analysis of the content of Met to HCys (-14 Da) byproducts in the crude exenatide from the cleavage using 1,3-BDMT as scavenger**

EIC-MS peaks identified as Met to HCys (-14 Da) byproducts: upper EIC-MS using DTT in the cleavage, lower EIC-MS using 1,3-BDMT in the cleavage

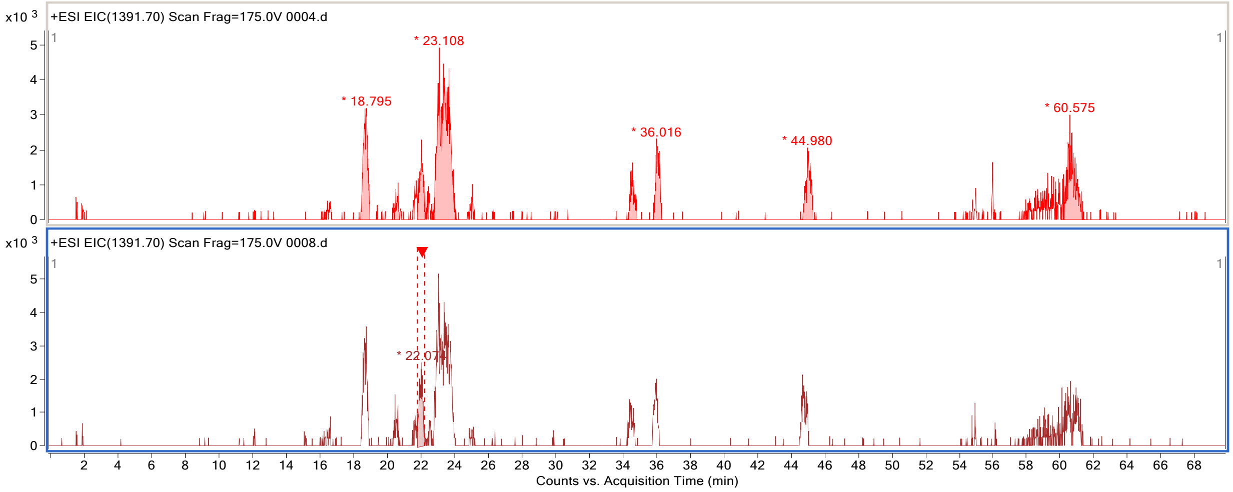


Summary of EIC areas for the identified Met to HCys (-14 Da) byproducts

| Peak | Rt | EIC area |
|---|---|---|
| 1 | 22,07 | 33866,66 |

**6.5.6 EIC-MS analysis of the content of Met to HCys (-14 Da) byproducts in the crude exenatide from the cleavage using 1,2-BDMT as scavenger**

EIC-MS peaks identified as Met to HCys (-14 Da) byproducts: upper EIC-MS using DTT in the cleavage, lower EIC-MS using 1,2-BDMT in the cleavage

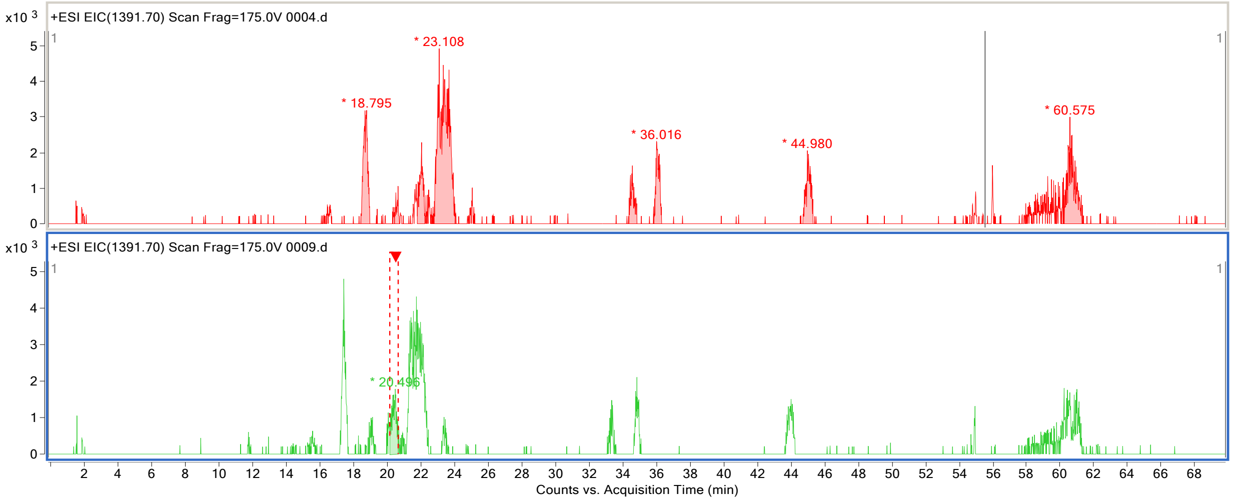


Summary of EIC areas for the identified Met to HCys (-14 Da) byproducts

| Peak | Rt | EIC area |
|---|---|---|
| 1 | 20,50 | 31607,13 |

### 6.5.7 EIC-MS analysis of the content of Met to HCys (-14 Da) byproducts in the crude exenatide from the cleavage using 4,4'-BMMB as scavenger

EIC-MS peaks identified as Met to HCys (-14 Da) byproducts: upper EIC-MS using DTT in the cleavage, lower EIC-MS using 4,4'-BMMB in the cleavage

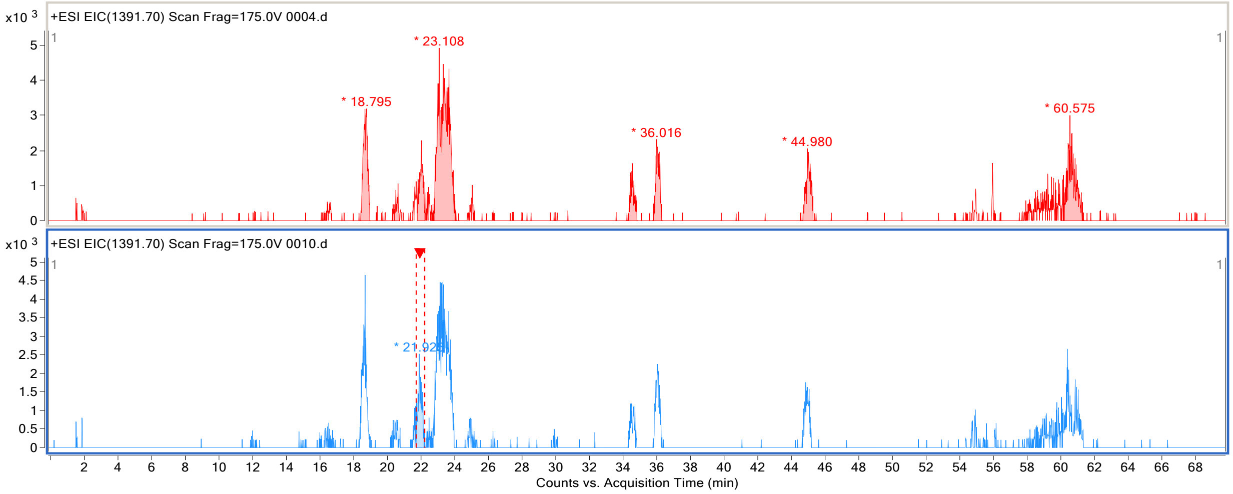


Summary of EIC areas for the identified Met to HCys (-14 Da) byproducts

| Peak | Rt | EIC area |
|---|---|---|
| 1 | 21,92 | 32503,72 |

**6.5.8 EIC-MS analysis of the content of Met to HCys (-14 Da) byproducts in the crude exenatide from the cleavage using 2,4-DCBM as scavenger**

EIC-MS peaks identified as Met to HCys (-14 Da) byproducts: upper EIC-MS using DTT in the cleavage, lower EIC-MS using 2,4-DCBM in the cleavage

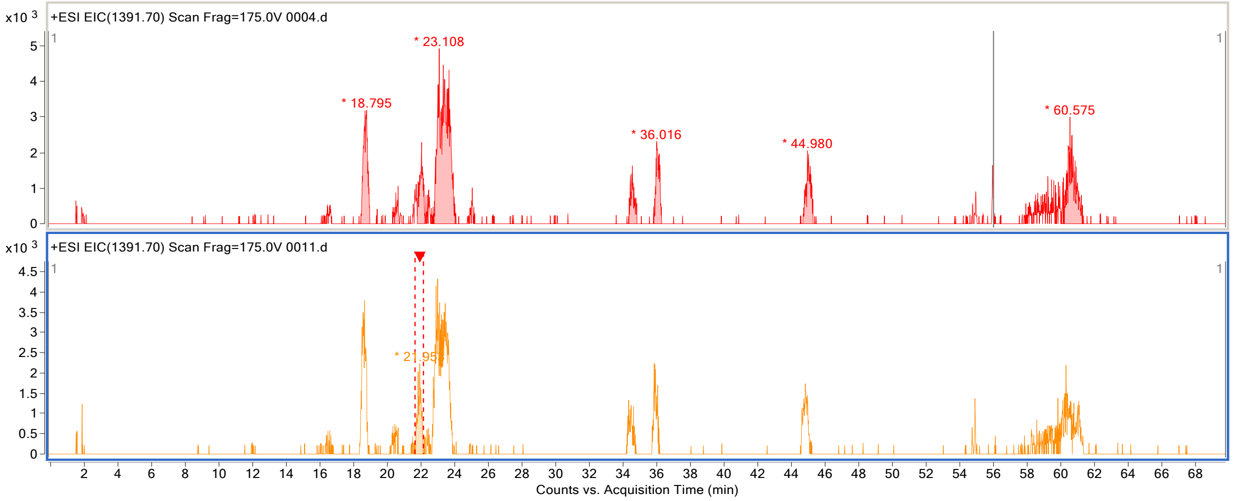


Summary of EIC areas for the identified Met to HCys (-14 Da) byproducts

| Peak | Rt | EIC area |
|---|---|---|
| 1 | 21,95 | 29732,65 |

**6.5.9 EIC-MS analysis of the content of Met to HCys (-14 Da) byproducts in the crude exenatide from the cleavage using 4-MOBM as scavenger**

EIC-MS peaks identified as Met to HCys (-14 Da) byproducts: upper EIC-MS using DTT in the cleavage, lower EIC-MS using 4-MOBM in the cleavage

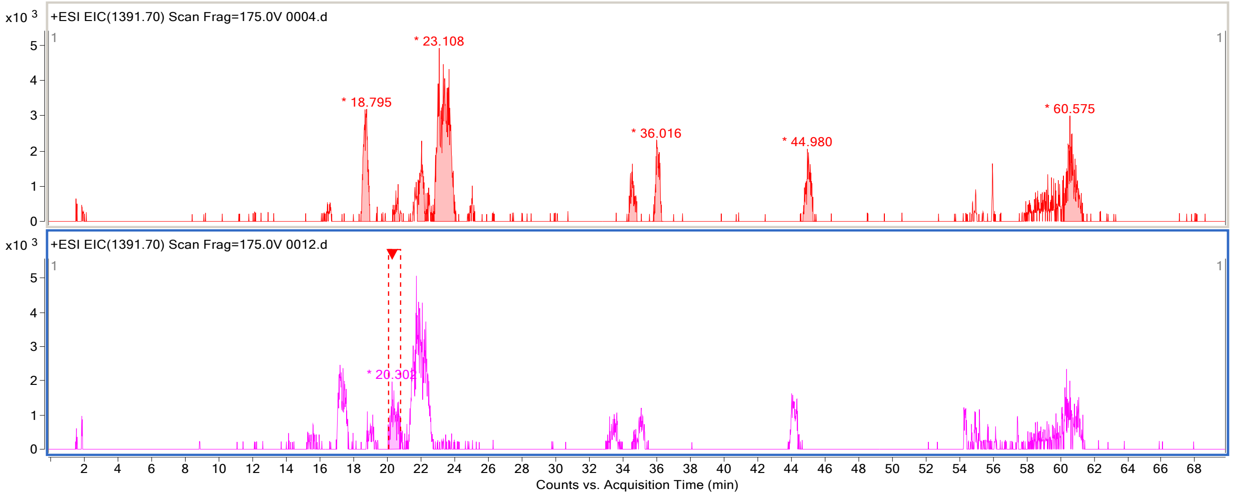


Summary of EIC areas for the identified Met to HCys (-14 Da) byproducts

| Peak | Rt | EIC area |
|---|---|---|
| 1 | 20,30 | 36976,19 |

**6.5.10 EIC-MS analysis of the content of Met to HCys (-14 Da) byproducts in the crude exenatide from the cleavage using TPMT as scavenger**

EIC-MS peaks identified as Met to HCys (-14 Da) byproducts: upper EIC-MS using DTT in the cleavage, lower EIC-MS using TPMT in the cleavage

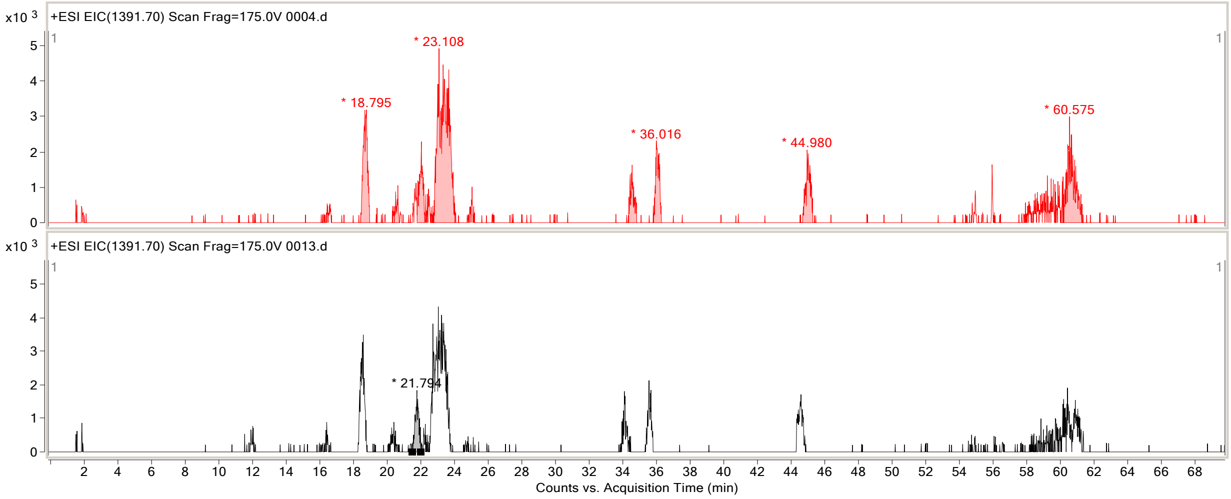


Summary of EIC areas for the identified Met to HCys (-14 Da) byproducts

| Peak | Rt | EIC area |
|---|---|---|
| 1 | 21,79 | 24865,73 |

**6.5.11 EIC-MS analysis of the content of Met to HCys (-14 Da) byproducts in the crude exenatide from the cleavage using 2,4-DMOT as scavenger**

EIC-MS peaks identified as Met to HCys (-14 Da) byproducts: upper EIC-MS using DTT in the cleavage, lower EIC-MS using 2,4-DMOT in the cleavage

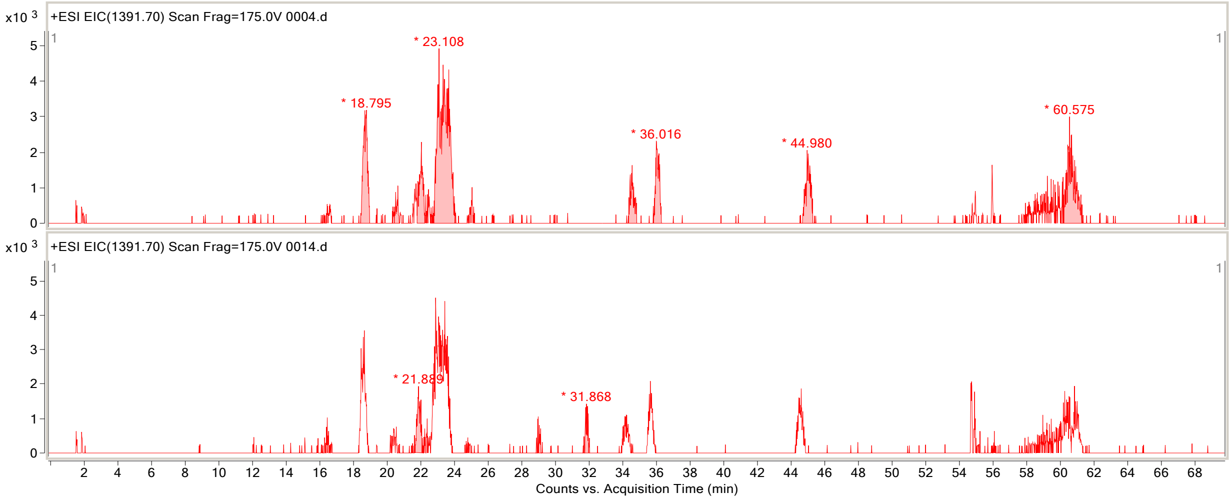


Summary of EIC areas for the identified Met to HCys (-14 Da) byproducts

| Peak | Rt | EIC area |
|---|---|---|
| 1 | 21,89 | 25125,48 |

### 6.5.12 EIC-MS analysis of the content of Met to HCys (-14 Da) byproducts in the crude exenatide from the cleavage not using a thiol scavenger

EIC-MS peaks identified as Met to HCys (-14 Da) byproducts: upper EIC-MS using DTT in the cleavage, lower EIC-MS not using a thiol in the cleavage

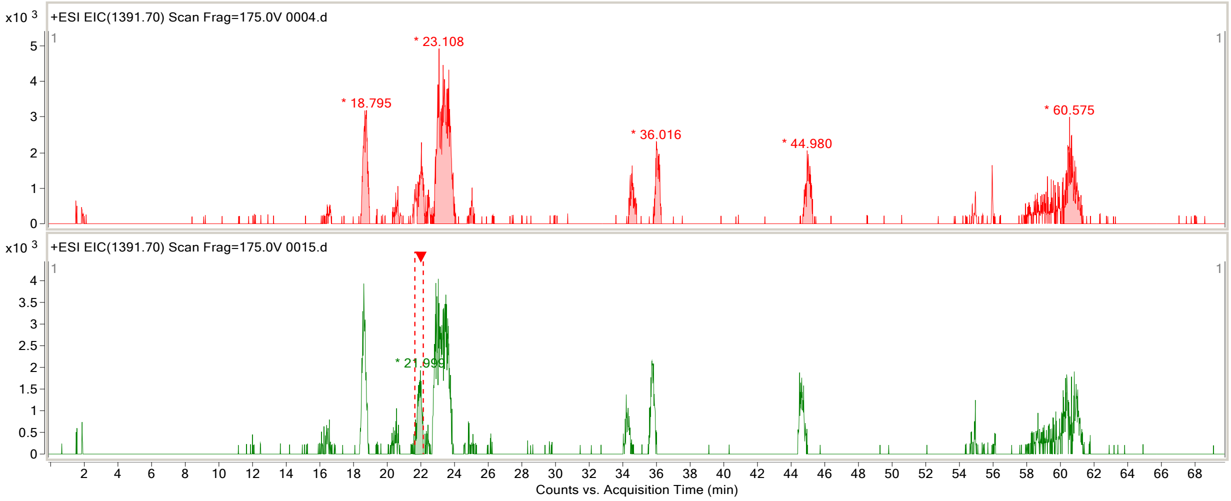


Summary of EIC areas for the identified Met to HCys (-14 Da) byproducts

| Peak | Rt | EIC area |
|---|---|---|
| 1 | 22,00 | 31681,52 |

## 6.6 EIC-MS analysis of the content of Met to Met(O) (+16 Da) byproducts

The original chromatograms were inspected at the m/z values corresponding to the Met to Met(O) impurities: 4201,0 (z=+1) and the most abundant 1401,7 (z=+3). The Met to Met(O) byproducts have the same molecular weight as the Trp oxidants (see section 6.4 in this Supporting Information) albeit the Met to Met(O) oxidant impurity elutes much earlier (Rt = ~12 min) than the Trp oxidants do (Rt ≥14,9 min). The identity of the Met to Met(O) impurity was confirmed by spiking a crude exenatide material with the authentic Met(O)-exenatide reference compound.

### 6.6.1 EIC-MS analysis of the content of Met to Met(O) (+16 Da) byproducts in the crude exenatide from the cleavage using DTT as scavenger

EIC-MS peaks identified as Met to Met(O) (+16 Da) byproducts: upper EIC-MS using DTT in the cleavage, lower EIC-MS using 2,4-DMOT in the cleavage

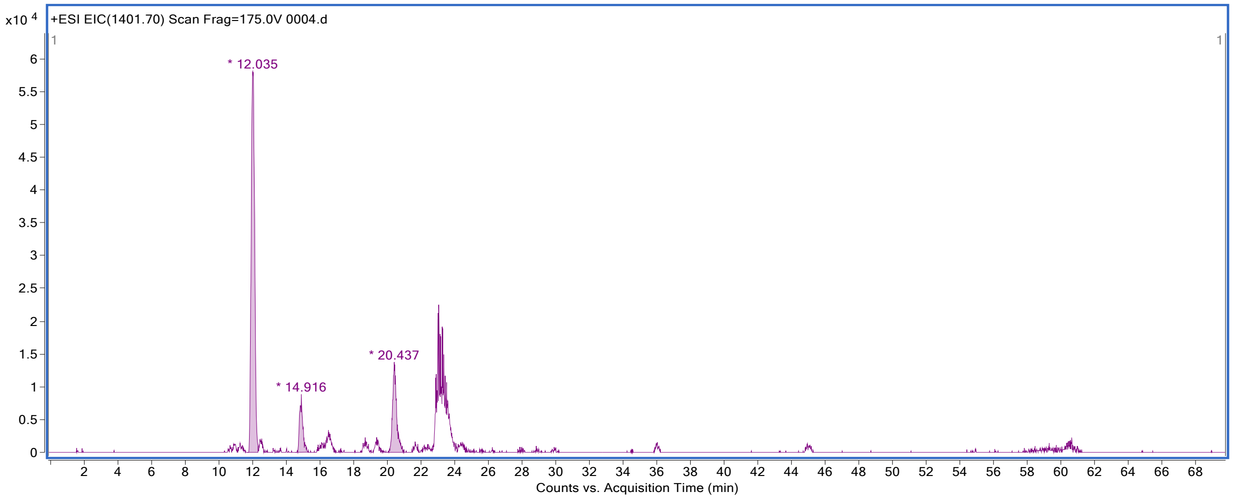


Summary of EIC areas for the identified Met to Met(O) (+16 Da) byproducts

| Peak | Rt | EIC area |
|---|---|---|
| 1 | 12,04 | 846138 |

### 6.6.2 EIC-MS analysis of the content of Met to Met(O) (+16 Da) byproducts in the crude exenatide from the cleavage using EDT as scavenger

EIC-MS peaks identified as Met to Met(O) (+16 Da) byproducts: upper EIC-MS using DTT in the cleavage, lower EIC-MS using EDT in the cleavage

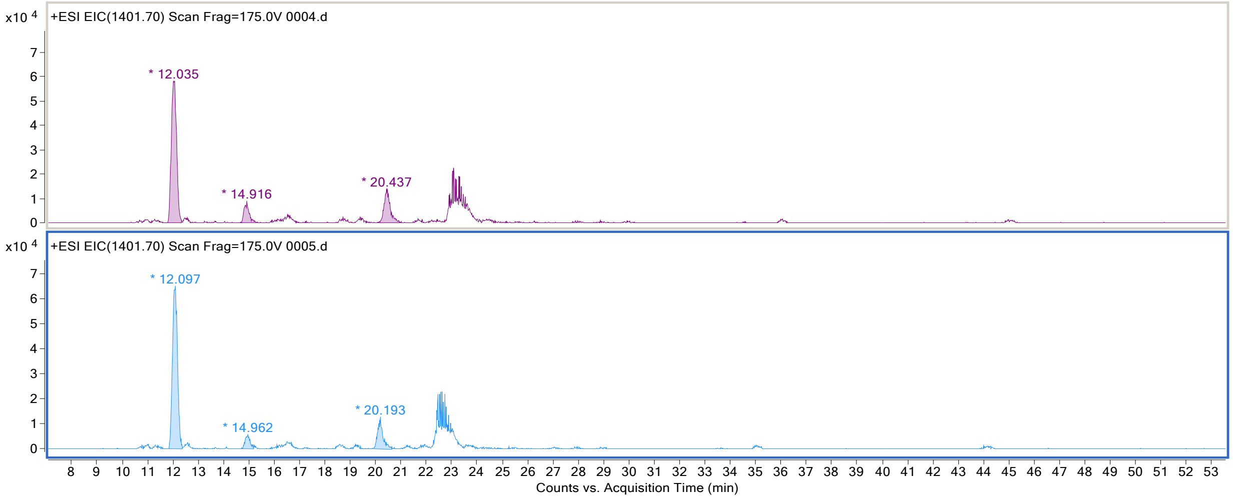


Summary of EIC areas for the identified Met to Met(O) (+16 Da) byproducts

| Peak | Rt | EIC area |
|---|---|---|
| 1 | 12,10 | 905439 |

**6.6.3 EIC-MS analysis of the content of Met to Met(O) (+16 Da) byproducts in the crude exenatide from the cleavage using DODT as scavenger**

EIC-MS peaks identified as Met to Met(O) (+16 Da) byproducts: upper EIC-MS using DTT in the cleavage, lower EIC-MS using DODT in the cleavage

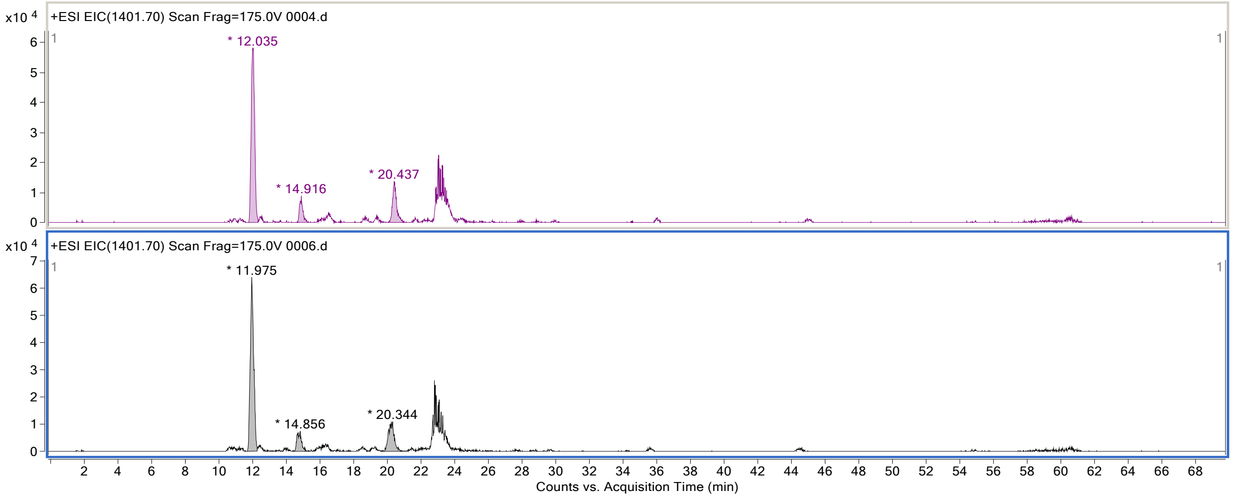


Summary of EIC areas for the identified Met to Met(O) (+16 Da) byproducts

| Peak | Rt | EIC area |
|---|---|---|
| 1 | 11,98 | 906423 |

**6.6.4 EIC-MS analysis of the content of Met to Met(O) (+16 Da) byproducts in the crude exenatide from the cleavage using 1,4-BDMT as scavenger**

EIC-MS peaks identified as Met to Met(O) (+16 Da) byproducts: upper EIC-MS using DTT in the cleavage, lower EIC-MS using 1,4-BDMT in the cleavage

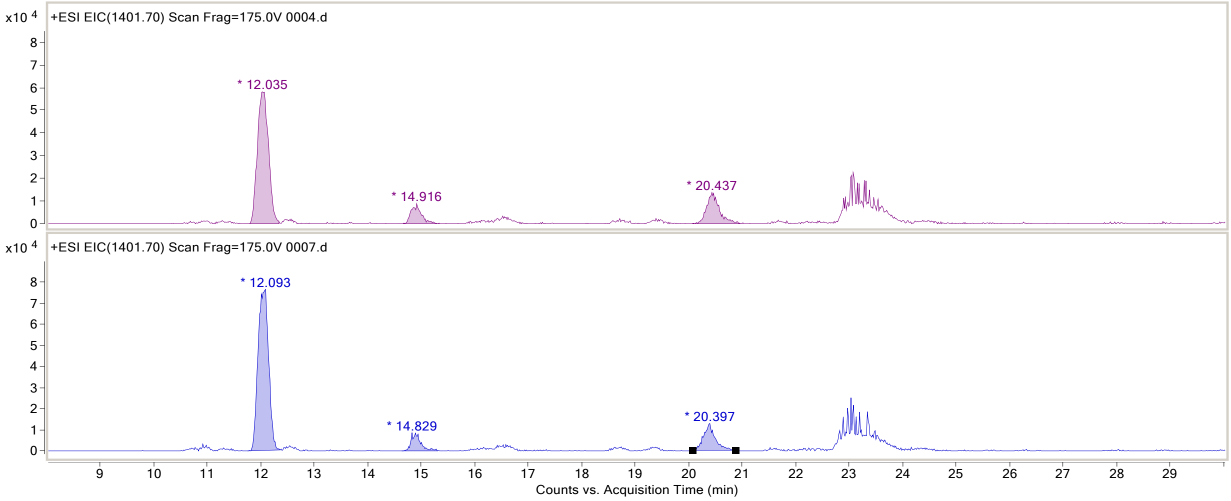


Summary of EIC areas for the identified Met to Met(O) (+16 Da) byproducts

| Peak | Rt | EIC area |
|---|---|---|
| 1 | 12,09 | 1057812 |

**6.6.5 EIC-MS analysis of the content of Met to Met(O) (+16 Da) byproducts in the crude exenatide from the cleavage using 1,3-BDMT as scavenger**

EIC-MS peaks identified as Met to Met(O) (+16 Da) byproducts: upper EIC-MS using DTT in the cleavage, lower EIC-MS using 1,3-BDMT in the cleavage

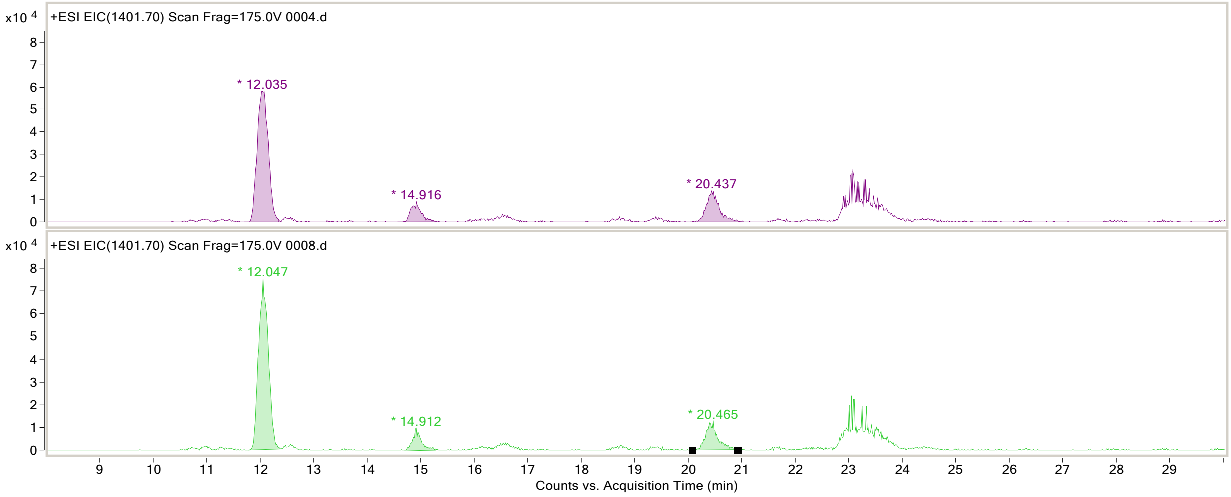


Summary of EIC areas for the identified Met to Met(O) (+16 Da) byproducts

| Peak | Rt | EIC area |
|---|---|---|
| 1 | 12,05 | 949976 |

**6.6.6 EIC-MS analysis of the content of Met to Met(O) (+16 Da) byproducts in the crude exenatide from the cleavage using 1,2-BDMT as scavenger**

EIC-MS peaks identified as Met to Met(O) (+16 Da) byproducts: upper EIC-MS using DTT in the cleavage, lower EIC-MS using 1,2-BDMT in the cleavage

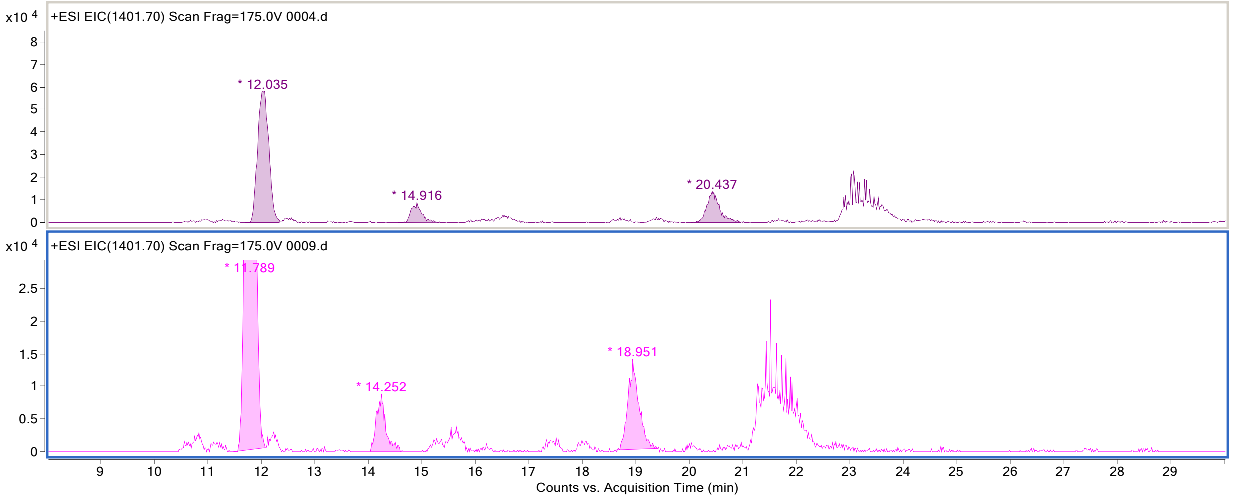


Summary of EIC areas for the identified Met to Met(O) (+16 Da) byproducts

| Peak | Rt | EIC area |
| --- | --- | --- |
| 1 | 11,79 | 1084083 |

### 6.6.7 EIC-MS analysis of the content of Met to Met(O) (+16 Da) byproducts in the crude exenatide from the cleavage using 4,4'-BMMB as scavenger

EIC-MS peaks identified as Met to Met(O) (+16 Da) byproducts: upper EIC-MS using DTT in the cleavage, lower EIC-MS using 4,4'-BMMB in the cleavage

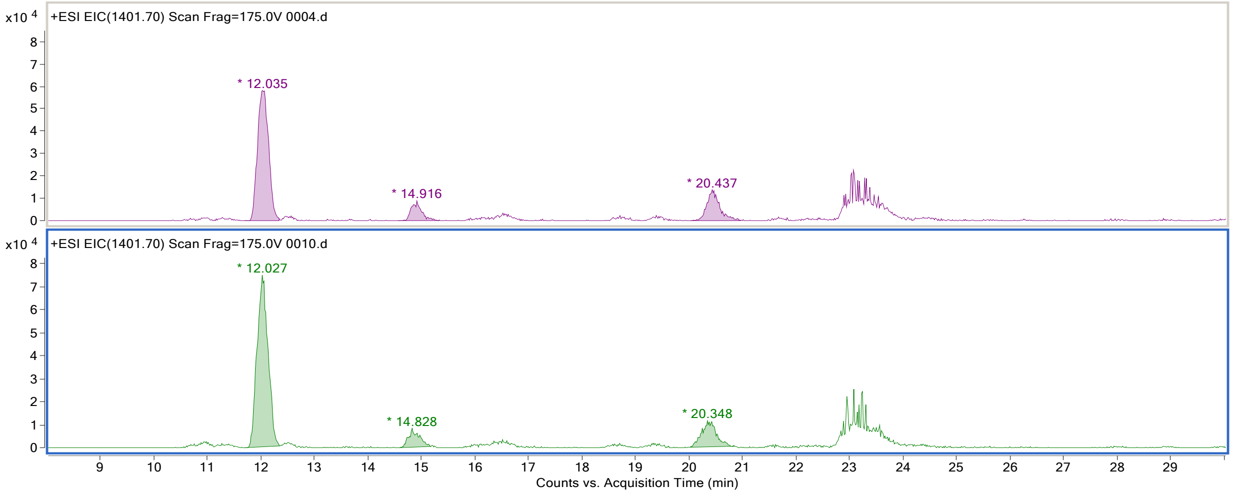


Summary of EIC areas for the identified Met to Met(O) (+16 Da) byproducts

| Peak | Rt | EIC area |
|---|---|---|
| 1 | 12,03 | 1066376 |

**6.6.8 EIC-MS analysis of the content of Met to Met(O) (+16 Da) byproducts in the crude exenatide from the cleavage using 2,4-DCBM as scavenger**

EIC-MS peaks identified as Met to Met(O) (+16 Da) byproducts: upper EIC-MS using DTT in the cleavage, lower EIC-MS using 2,4-DCBM in the cleavage

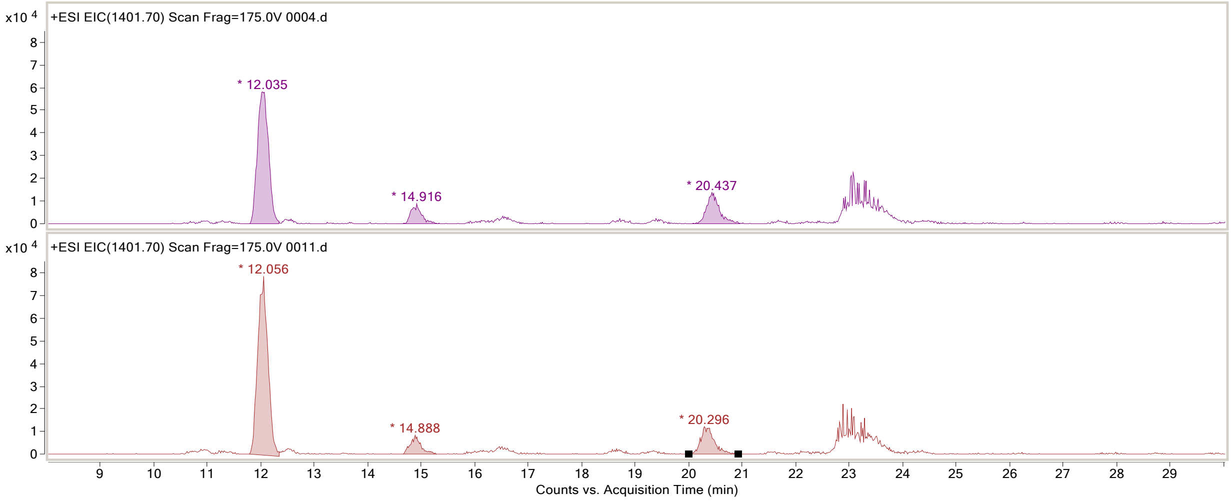


Summary of EIC areas for the identified Met to Met(O) (+16 Da) byproducts

| Peak | Rt | EIC area |
|---|---|---|
| 1 | 12,06 | 1049161 |

### 6.6.9 EIC-MS analysis of the content of Met to Met(O) (+16 Da) byproducts in the crude exenatide from the cleavage using 4-MOBM as scavenger

EIC-MS peaks identified as Met to Met(O) (+16 Da) byproducts: upper EIC-MS using DTT in the cleavage, lower EIC-MS using 4-MOBM in the cleavage

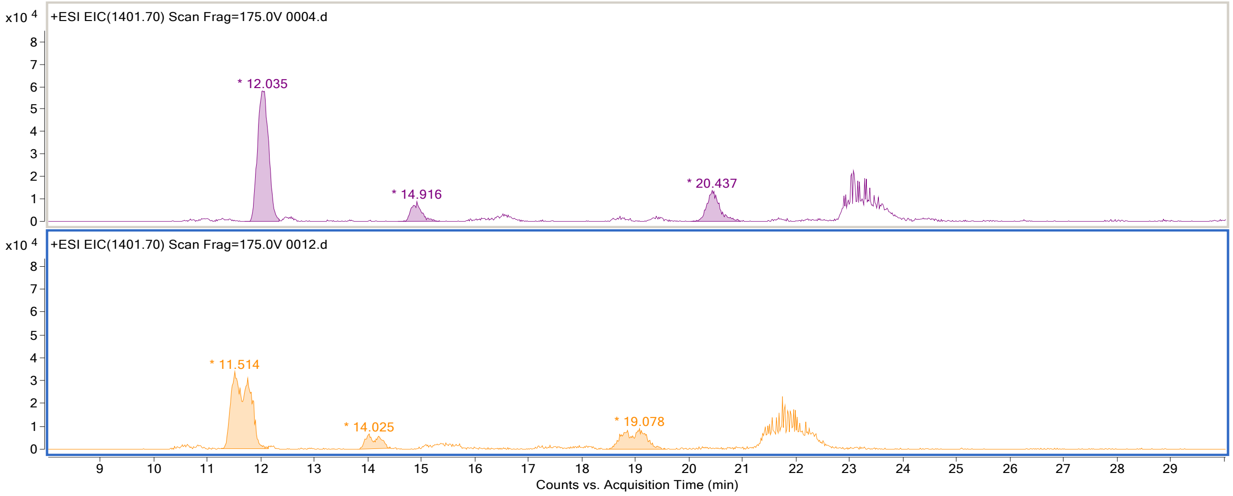


Summary of EIC areas for the identified Met to Met(O) (+16 Da) byproducts

| Peak | Rt | EIC area |
|---|---|---|
| 1 | 11,51 | 790535 |

**6.6.10 EIC-MS analysis of the content of Met to Met(O) (+16 Da) byproducts in the crude exenatide from the cleavage using TPMT as scavenger**

EIC-MS peaks identified as Met to Met(O) (+16 Da) byproducts: upper EIC-MS using DTT in the cleavage, lower EIC-MS using TPMT in the cleavage

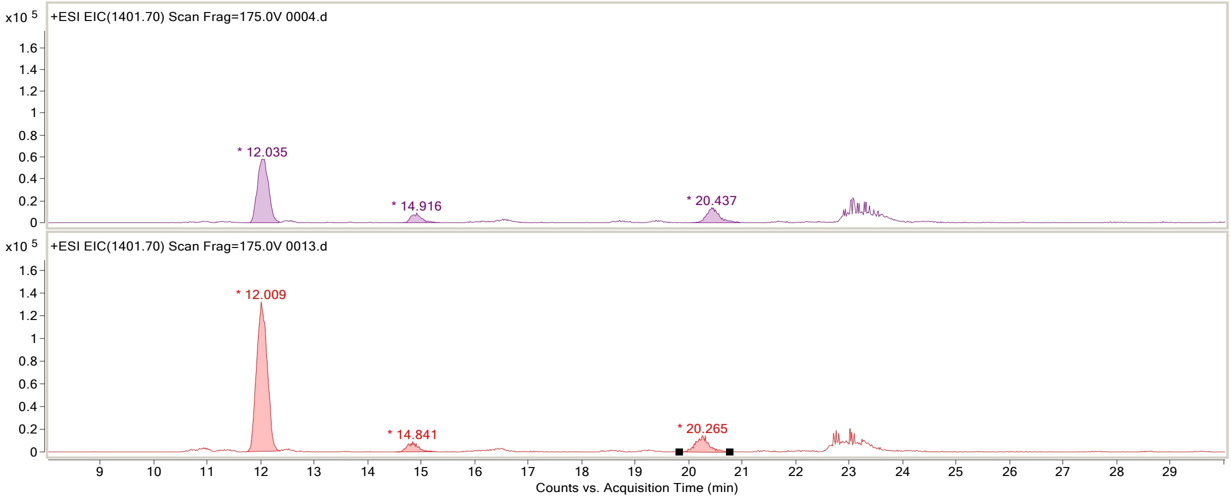


Summary of EIC areas for the identified Met to Met(O) (+16 Da) byproducts

| Peak | Rt | EIC area |
|---|---|---|
| 1 | 12,01 | 1832764 |

**6.6.11 EIC-MS analysis of the content of Met to Met(O) (+16 Da) byproducts in the crude exenatide from the cleavage using 2,4-DMOT as scavenger**

EIC-MS peaks identified as Met to Met(O) (+16 Da) byproducts: upper EIC-MS using DTT in the cleavage, lower EIC-MS using 2,4-DMOT in the cleavage

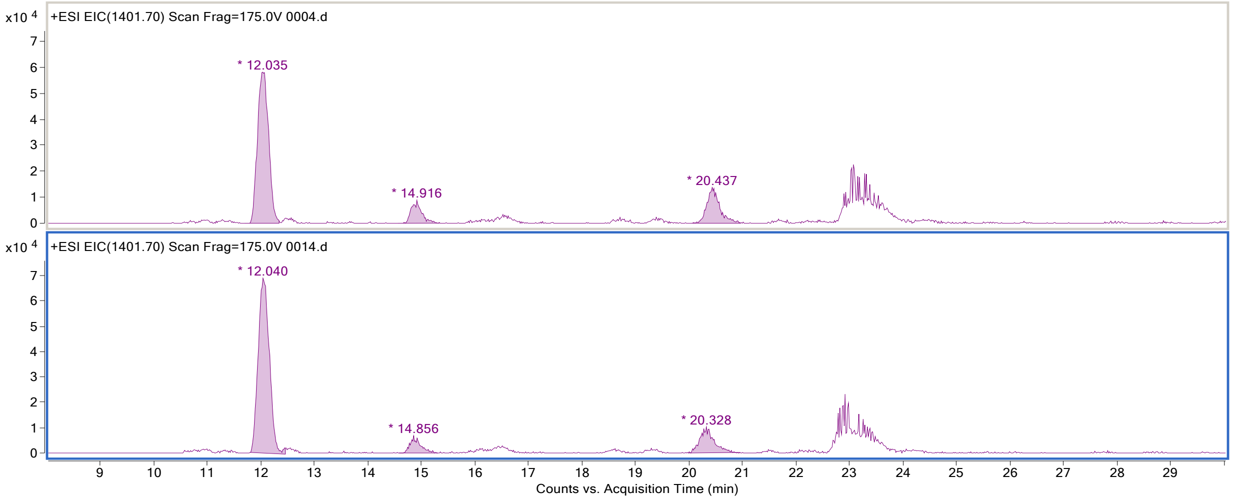


Summary of EIC areas for the identified Met to Met(O) (+16 Da) byproducts

| Peak | Rt | EIC area |
|---|---|---|
| 1 | 12,04 | 1008422 |

### 6.6.12 EIC-MS analysis of the content of Met to Met(O) (+16 Da) byproducts in the crude exenatide from the cleavage not using a thiol scavenger

EIC-MS peaks identified as Met to Met(O) (+16 Da) byproducts: upper EIC-MS using DTT in the cleavage, lower EIC-MS not using a thiol in the cleavage

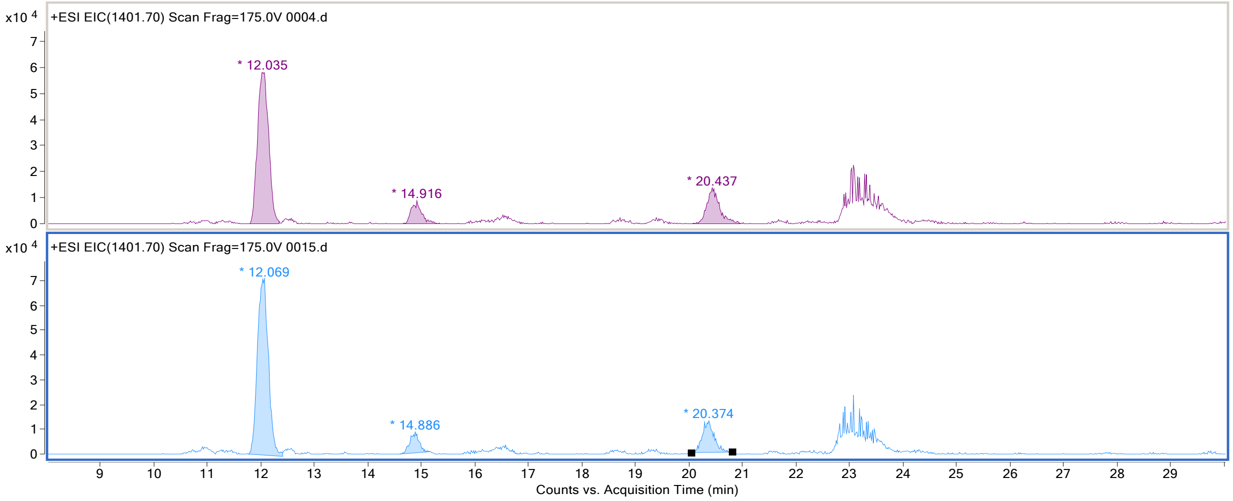


Summary of EIC areas for the identified Met to Met(O) (+16 Da) byproducts

| Peak | Rt | EIC area |
|---|---|---|
| 1 | 12,07 | 1017662 |

## 6.7 EIC-MS analysis of the content of scavenger adduct byproducts

The expected molecular weights of the scavenger adduct by product were calculated as (MW of exenatide) + (MW of scavenger) – 2 Da. The 2 Da were subtracted to account for loss of a proton from both exenatide and the scavenger during the formation of the adduct. The original chromatograms were inspected at the m/z values corresponding to scavenger adduct impurities, these were as follows: i) DTT as scavenger (+152 Da): 4337,0 (z=+1) and the most abundant 1447.1 (z=+3); ii) EDT as scavenger (+92 Da): 4277,0 (z=+1) and the most abundant 1427,1 (z=+3); iii) DODT as scavenger (+180 Da): 4365,2 (z=+1) and the most abundant 1456,4 (z=+3); iv) 1,2-, 1,3- and 1,4-BDMT as scavenger (+ 168 Da): 4353,1 (z=+1) and the most abundant 1452,4 (z=+3); v) 4,4'BMMB as scavenger (+244 Da): 4429,1 (z=+1) and the most abundant 1477,7 (z=+3); vi) 2,4-DCBM as scavenger (+190 Da): 4375,3 (z=+1) and the most abundant 1460,1 (z=+3); vii) 4-MOBM as scavenger (+152 Da): 4337,0 (z=+1) and the most abundant 1447,1 (z=+3); viii) TPMT as scavenger (+274 Da): 4459,1 (z=+1) and the most abundant 1487,7 (z=+3); viii) 2,4-DMOT as scavenger (+168 Da): 4353,2 (z=+1) and the most abundant 1452,4 (z=+3).

### 6.7.1 EIC-MS analysis of the content of scavenger adduct byproducts (+152 Da) in the crude exenatide from the cleavage using DTT as scavenger

EIC-MS peaks identified as DTT adduct (+152 Da) byproduct:

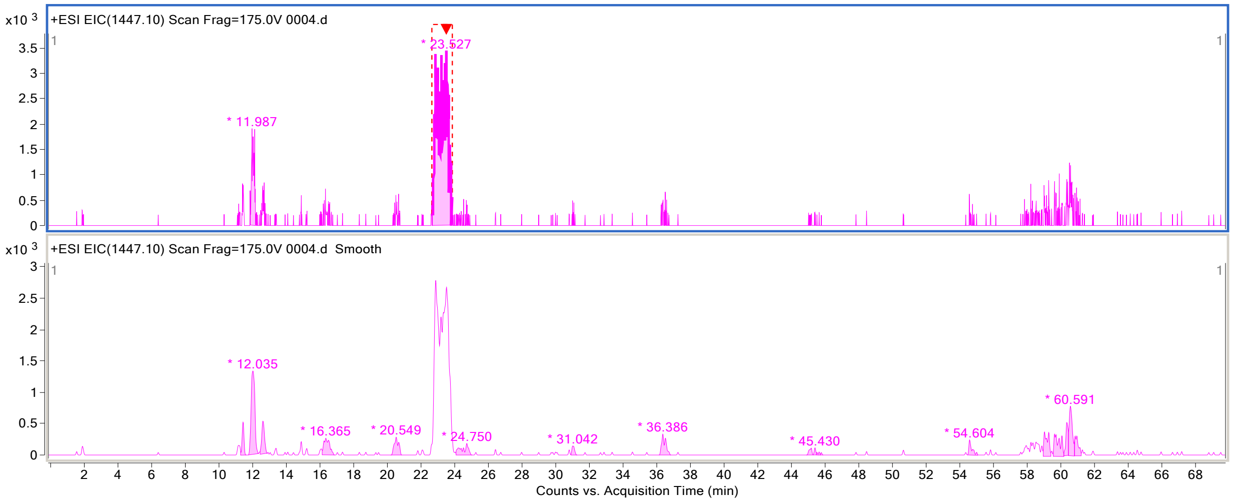


Summary of EIC areas for the identified DTT adduct (+152 Da) byproducts

No match for DTT adduct found

### 6.7.2 EIC-MS analysis of the content of scavenger adduct byproducts (+92 Da) in the crude exenatide from the cleavage using EDT as scavenger

EIC-MS peaks identified as EDT adduct (+92 Da) byproduct:

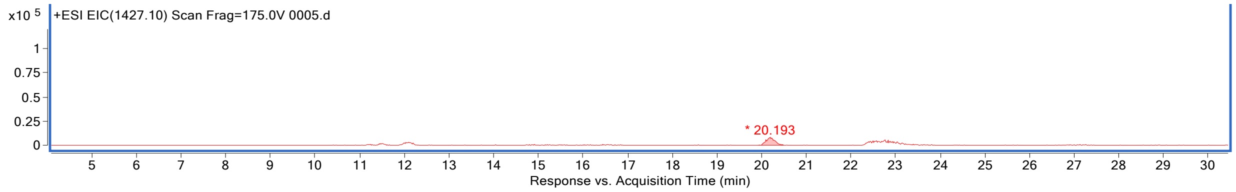


Summary of EIC areas for the identified EDT adduct (+92 Da) byproducts

| Peak | Rt | EIC area |
|---|---|---|
| 1 | 20.19 | 109821 |

### 6.7.3 EIC-MS analysis of the content of scavenger adduct byproducts (+180 Da) in the crude exenatide from the cleavage using DODT as scavenger

EIC-MS peaks identified as DODT adduct (+180 Da) byproduct:

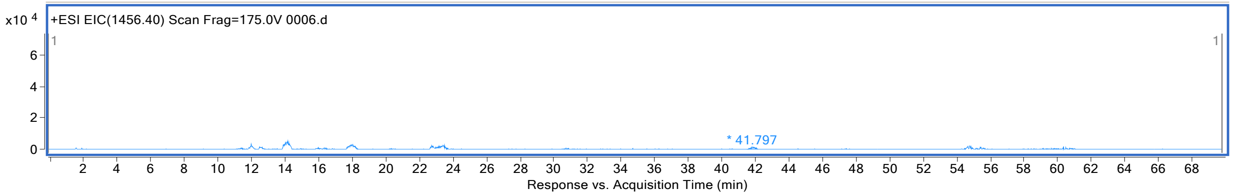


Summary of EIC areas for the identified DODT adduct (+180 Da) byproducts

| Peak | Rt | EIC area |
|---|---|---|
| 1 | 41.80 | 21.827 |

**6.7.4 EIC-MS analysis of the content of scavenger adduct byproducts (+ 168 Da) in the crude exenatide from the cleavage using 1,4-BDMT as scavenger**

EIC-MS peaks identified as 1,4-BDMT adduct (+168 Da) byproduct:

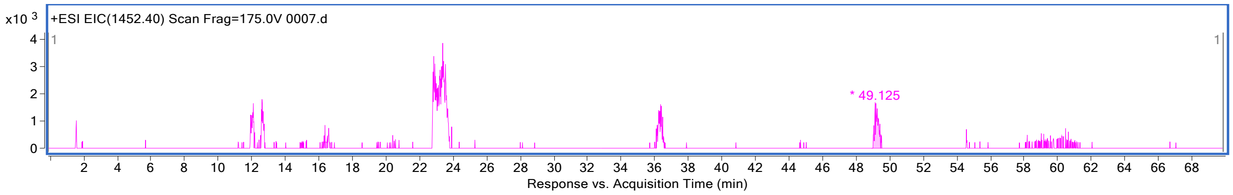


Summary of EIC areas for the identified 1,4-BDMT adduct (+168 Da) byproducts

| Peak | Rt | EIC area |
|---|---|---|
| 1 | 49.13 | 23454 |

**6.7.5 EIC-MS analysis of the content of scavenger adduct byproducts (+ 168 Da) in the crude exenatide from the cleavage using 1,3-BDMT as scavenger**

EIC-MS peaks identified as 1,3-BDMT adduct (+168 Da) byproduct:

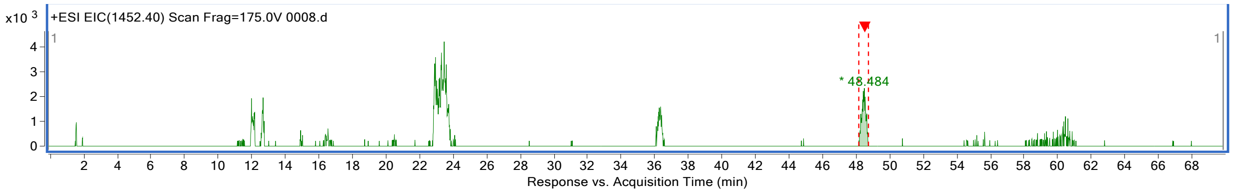


Summary of EIC areas for the identified 1,3-BDMT adduct (+168 Da) byproducts

| Peak | Rt | EIC area |
|---|---|---|
| 1 | 48.48 | 37953 |

### 6.7.6 EIC-MS analysis of the content of scavenger adduct byproducts (+ 168 Da) in the crude exenatide from the cleavage using 1,2-BDMT as scavenger

EIC-MS peaks identified as 1,2-BDMT adduct (+168 Da) byproduct:

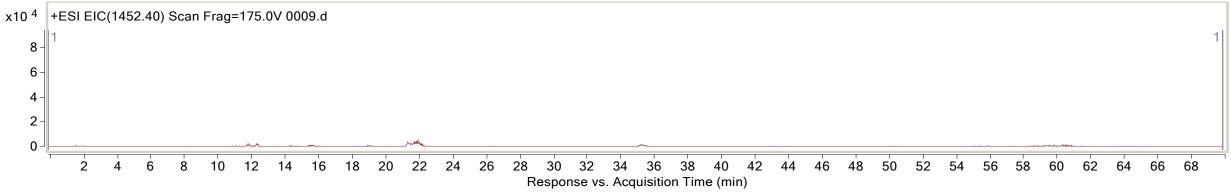


Summary of EIC areas for the identified 1,2-BDMT adduct (+168 Da) byproducts

No match for 1,2-BDMT adduct found

**6.7.7 EIC-MS analysis of the content of scavenger adduct byproducts (+244 Da) in the crude exenatide from the cleavage using 4,4’-BMMB as scavenger**

EIC-MS peaks identified as 4,4’-BMMB adduct (+244 Da) byproduct:

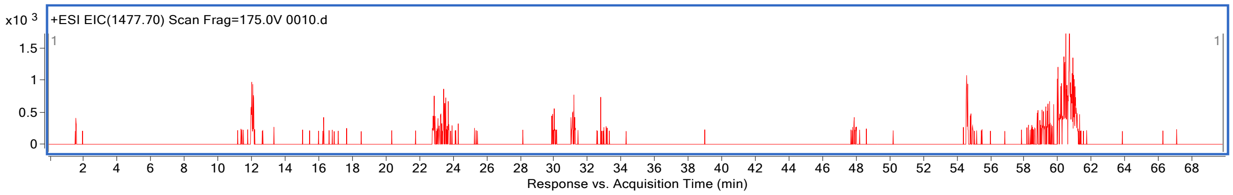


Summary of EIC areas for the identified 4,4’-BMMB adduct (+244 Da) byproducts

No match for 4,4’-BMMB adduct found

### 6.7.8 EIC-MS analysis of the content of scavenger adduct byproducts (+190 Da) in the crude exenatide from the cleavage using 2,4-DCBM as scavenger

EIC-MS peaks identified as 2,4-DCBM adduct (+152 Da) byproduct:

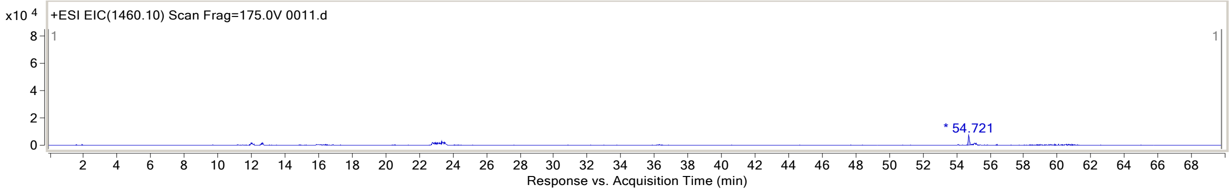


Summary of EIC areas for the identified 2,4-DCBM adduct (+152 Da) byproducts

| Peak | Rt | EIC area |
|---|---|---|
| 1 | 54.72 | 38425,76 |

**6.7.9 EIC-MS analysis of the content of scavenger adduct byproducts (+152 Da) in the crude exenatide from the cleavage using 4-MOBM as scavenger**

EIC-MS peaks identified as 4-MOBM adduct (+152 Da) byproducts:

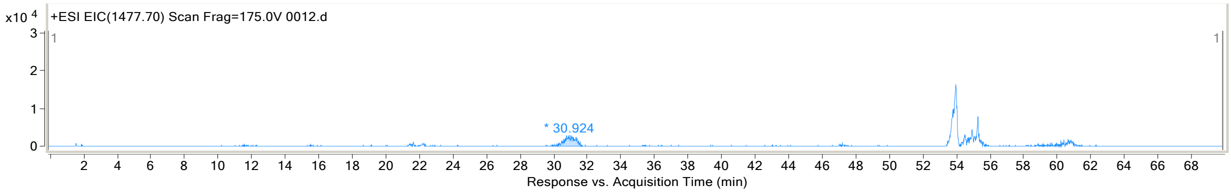


Summary of EIC areas for the identified 4-MOBM adduct (+152 Da) byproducts

| Peak | Rt | EIC area |
|---|---|---|
| 1 | 30.92 | 121191 |

### 6.7.10 EIC-MS analysis of the content of scavenger adduct byproducts (+274 Da) in the crude exenatide from the cleavage using TPMT as scavenger

EIC-MS peaks identified as TPMT adduct (+274 Da) byproducts:

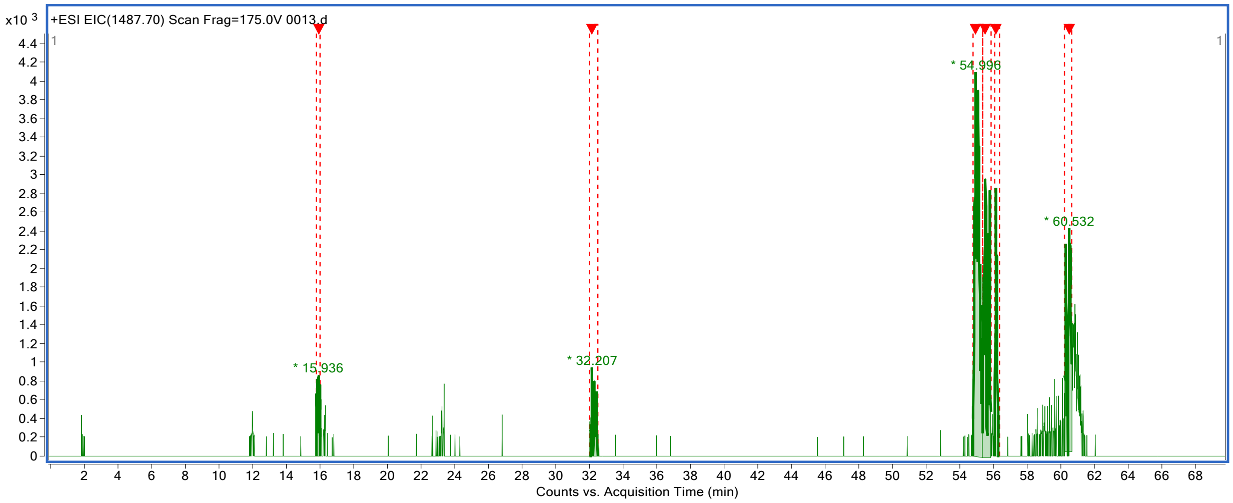


Summary of EIC areas for the identified TPMT adduct (+274 Da) byproducts

No match for TPMT adduct found

**6.7.11 EIC-MS analysis of the content of scavenger adduct byproducts (+168 Da) in the crude exenatide from the cleavage using 2,4-DMOT as scavenger**

EIC-MS peaks identified as 2,4-DMOT adduct (+168 Da) byproducts:

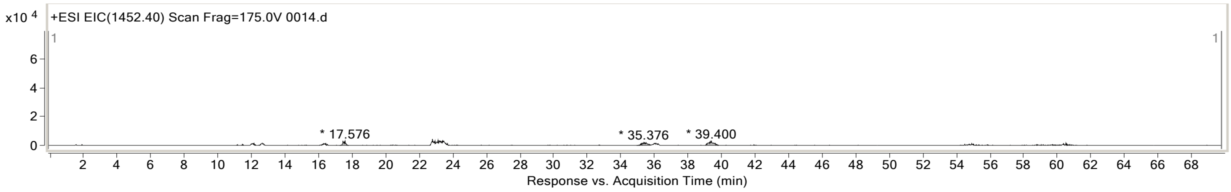


Summary of EIC areas for the identified 2,4-DMOT adduct (+168 Da) byproducts

| Peak | Rt | EIC area |
|---|---|---|
| 1 | 39,48 | 58500,5 |

## 6.8 Summary of EIC areas for all impurities and recalculation to the UV% areas

**Table S27.** Overview of EIC areas for all specific impurities investigated by EIC-MS.

| Scavenger | t-Bu adducts | Pbf adducts | $SO_3$ adducts | Met to HCys | Met to Met(O) | Trp ox | Add on scavenger |
|---|---|---|---|---|---|---|---|
| DTT | 988140 | 47472 | 169201 | 30282 | 846138 | 340459 | 0 |
| EDT | 548872 | 22773 | 20629 | 36498 | 905439 | 254874 | 109821 |
| DODT | 2257437 | 58184 | 198012 | 34914 | 906423 | 389419 | 21827 |
| 1,4-BDMT | 487183 | 20463 | 17421 | 26909 | 1057812 | 290926 | 23454 |
| 1,3-BDMT | 634170 | 25357 | 51596 | 33867 | 949976 | 298323 | 37953 |
| 1,2-BDMT | 592958 | 22892 | 43851 | 31607 | 1084083 | 299510 | 0 |
| 4,4'-BMMB | 1750046 | 88518 | 766828 | 32504 | 1066376 | 324497 | 0 |
| 2,4-DCBM | 758792 | 46671 | 196341 | 29733 | 1049161 | 319037 | 38426 |
| 4-MOBM | 1115826 | 0 | 67538 | 36976 | 790535 | 369879 | 121191 |
| TPMT | 670159 | 27138 | 375455 | 24866 | 1832764 | 363605 | 0 |
| 2,4-DMOT | 1431168 | 0 | 176792 | 25125 | 1008422 | 265457 | 58500,5 |
| none | 1708883 | 79840 | 775882 | 31682 | 1017662 | 288670 | 0 |

**Table S28.** Overview of UV% areas for all specific impurities investigated by EIC-MS. Met to Met(O) in the EDT crude (905439 in EIC = 1% in UV) was used for the EIC->UV% recalculation.

| Scavenger | t-Bu adducts | Pbf adductss | $SO_3$ adducts | Met to HCys | Met to Met(O) | Trp ox | Add on scavenger |
|---|---|---|---|---|---|---|---|
| DTT | 1,09 | 0,05 | 0,19 | 0,03 | 0,93 | 0,38 | 0,00 |
| EDT | 0,61 | 0,03 | 0,02 | 0,04 | 1,00 | 0,28 | 0,12 |
| DODT | 2,49 | 0,06 | 0,22 | 0,04 | 1,00 | 0,43 | 0,02 |
| 1,4-BDMT | 0,54 | 0,02 | 0,02 | 0,03 | 1,17 | 0,32 | 0,03 |
| 1,3-BDMT | 0,70 | 0,03 | 0,06 | 0,04 | 1,05 | 0,33 | 0,04 |
| 1,2-BDMT | 0,65 | 0,03 | 0,05 | 0,03 | 1,20 | 0,33 | 0,00 |
| 4,4'-BMMB | 1,93 | 0,10 | 0,85 | 0,04 | 1,18 | 0,36 | 0,00 |
| 2,4-DCBM | 0,84 | 0,05 | 0,22 | 0,03 | 1,16 | 0,35 | 0,04 |
| 4-MOBM | 1,23 | 0,00 | 0,07 | 0,04 | 0,87 | 0,41 | 0,13 |
| TPMT | 0,74 | 0,03 | 0,41 | 0,03 | 2,02 | 0,40 | 0,00 |
| 2,4-DMOT | 1,58 | 0,00 | 0,20 | 0,03 | 1,11 | 0,29 | 0,06 |
| none | 1,89 | 0,09 | 0,86 | 0,03 | 1,12 | 0,32 | 0,00 |

## 7. MS-MS analyses of exenatide from TFA cleavage using 2,4-DCBM as scavenger

LC-MS/MS was performed on the crude exenatide sample from TFA cleavage using 2,4-DCBM (DCBM) as scavenger to determine where the DCBM moiety is situated in the peptide chain.LC-HRMS method and instrumentation: see section 5.MS/MS: Using CID at 70V and 80V. Collision induced dissociation mainly gives N terminal b-fragments and C-terminal y-fragments. MS/MS was performed on the main exenatide peak and the add on DCBM peak (+190 Da).

**Table S29.** Overview of fragment ions for exenatide

| Most abundant | | | a | N-Terminal Ion Series | | | b | N-Terminal Ion Series | | | | |
|---|---|---|---|---|---|---|---|---|---|---|---|---|
| Exenatide | | | Ion | $a^{(1+)}$ | $a^{(2+)}$ | $a^{(3+)}$ | Ion | $b^{(1+)}$ | $b^{(2+)}$ | $b^{(3+)}$ | $b^{(4+)}$ | $b^{(5+)}$ |
| 1 | His | 137,06 | 0 | | | | 0 | | | | | |
| 2 | Gly | 57,02 | a1 | 110,07 | 55,54 | 37,36 | b1 | 138,07 | 69,54 | 46,69 | 35,27 | 28,42 |
| 3 | Glu | 129,04 | a2 | 167,09 | 84,05 | 56,37 | b2 | 195,09 | 98,05 | 65,70 | 49,53 | 39,82 |
| 4 | Gly | 57,02 | a3 | 296,14 | 148,57 | 99,38 | b3 | 324,13 | 162,57 | 108,71 | 81,79 | 65,63 |
| 5 | Thr | 101,05 | a4 | 353,16 | 177,08 | 118,39 | b4 | 381,15 | 191,08 | 127,72 | 96,04 | 77,04 |
| 6 | Phe | 147,07 | a5 | 454,20 | 227,61 | 152,07 | b5 | 482,20 | 241,60 | 161,40 | 121,31 | 97,25 |
| 7 | Thr | 101,05 | a6 | 601,27 | 301,14 | 201,10 | b6 | 629,27 | 315,14 | 210,43 | 158,07 | 126,66 |
| 8 | Ser | 87,03 | a7 | 702,32 | 351,66 | 234,78 | b7 | 730,32 | 365,66 | 244,11 | 183,33 | 146,87 |
| 9 | Asp | 115,03 | a8 | 789,35 | 395,18 | 263,79 | b8 | 817,35 | 409,18 | 273,12 | 205,09 | 164,28 |
| 10 | Leu | 113,08 | a9 | 904,38 | 452,69 | 302,13 | b9 | 932,37 | 466,69 | 311,46 | 233,85 | 187,28 |
| 11 | Ser | 87,03 | a10 | 1 017,46 | 509,24 | 339,83 | b10 | 1 045,46 | 523,23 | 349,16 | 262,12 | 209,90 |
| 12 | Lys | 128,09 | a11 | 1 104,50 | 552,75 | 368,84 | b11 | 1 132,49 | 566,75 | 378,17 | 283,88 | 227,30 |
| 13 | Gln | 128,06 | a12 | 1 232,59 | 616,80 | 411,54 | b12 | 1 260,59 | 630,80 | 420,87 | 315,90 | 252,92 |
| 14 | Met | 131,04 | a13 | 1 360,65 | 680,83 | 454,22 | b13 | 1 388,64 | 694,83 | 463,55 | 347,92 | 278,53 |
| 15 | Glu | 129,04 | a14 | 1 491,69 | 746,35 | 497,90 | b14 | 1 519,68 | 760,35 | 507,23 | 380,68 | 304,74 |
| 16 | Glu | 129,04 | a15 | 1 620,73 | 810,87 | 540,92 | b15 | 1 648,73 | 824,87 | 550,25 | 412,94 | 330,55 |
| 17 | Glu | 129,04 | a16 | 1 749,77 | 875,39 | 583,93 | b16 | 1 777,77 | 889,39 | 593,26 | 445,20 | 356,36 |
| 18 | Ala | 71,04 | a17 | 1 878,82 | 939,91 | 626,94 | b17 | 1 906,81 | 953,91 | 636,28 | 477,46 | 382,17 |
| 19 | Val | 99,07 | a18 | 1 949,85 | 975,43 | 650,62 | b18 | 1 977,85 | 989,43 | 659,95 | 495,22 | 396,38 |
| 20 | Arg | 156,10 | a19 | 2 048,92 | 1 024,97 | 683,65 | b19 | 2 076,92 | 1 038,96 | 692,98 | 519,98 | 416,19 |
| 21 | Leu | 113,08 | a20 | 2 205,02 | 1 103,02 | 735,68 | b20 | 2 233,02 | 1 117,01 | 745,01 | 559,01 | 447,41 |
| 22 | Phe | 147,07 | a21 | 2 318,11 | 1 159,56 | 773,37 | b21 | 2 346,10 | 1 173,56 | 782,71 | 587,28 | 470,03 |
| 23 | Ile | 113,08 | a22 | 2 465,18 | 1 233,09 | 822,40 | b22 | 2 493,17 | 1 247,09 | 831,73 | 624,05 | 499,44 |
| 24 | Glu | 129,04 | a23 | 2 578,26 | 1 289,63 | 860,09 | b23 | 2 606,26 | 1 303,63 | 869,42 | 652,32 | 522,06 |
| 25 | Trp | 186,08 | a24 | 2 707,30 | 1 354,16 | 903,11 | b24 | 2 735,30 | 1 368,15 | 912,44 | 684,58 | 547,87 |
| 26 | Leu | 113,08 | a25 | 2 893,38 | 1 447,19 | 965,13 | b25 | 2 921,38 | 1 461,19 | 974,46 | 731,10 | 585,08 |
| 27 | Lys | 128,09 | a26 | 3 006,47 | 1 503,74 | 1 002,83 | b26 | 3 034,46 | 1 517,73 | 1 012,16 | 759,37 | 607,70 |
| 28 | Asn | 114,04 | a27 | 3 134,56 | 1 567,78 | 1 045,53 | b27 | 3 162,56 | 1 581,78 | 1 054,86 | 791,39 | 633,32 |
| 29 | Gly | 57,02 | a28 | 3 248,60 | 1 624,81 | 1 083,54 | b28 | 3 276,60 | 1 638,80 | 1 092,87 | 819,91 | 656,13 |
| 30 | Gly | 57,02 | a29 | 3 305,63 | 1 653,32 | 1 102,55 | b29 | 3 333,62 | 1 667,31 | 1 111,88 | 834,16 | 667,53 |
| 31 | Pro | 97,05 | a30 | 3 362,65 | 1 681,83 | 1 121,55 | b30 | 3 390,64 | 1 695,82 | 1 130,89 | 848,42 | 678,93 |
| 32 | Ser | 87,03 | a31 | 3 459,70 | 1 730,35 | 1 153,90 | b31 | 3 487,70 | 1 744,35 | 1 163,24 | 872,68 | 698,34 |
| 33 | Ser | 87,03 | a32 | 3 546,73 | 1 773,87 | 1 182,92 | b32 | 3 574,73 | 1 787,87 | 1 192,25 | 894,44 | 715,75 |
| 34 | Gly | 57,02 | a33 | 3 633,76 | 1 817,39 | 1 211,93 | b33 | 3 661,76 | 1 831,38 | 1 221,26 | 916,20 | 733,16 |
| 35 | Ala | 71,04 | a34 | 3 690,79 | 1 845,90 | 1 230,93 | b34 | 3 718,78 | 1 859,89 | 1 240,27 | 930,45 | 744,56 |
| 36 | Pro | 97,05 | a35 | 3 761,82 | 1 881,42 | 1 254,61 | b35 | 3 789,82 | 1 895,41 | 1 263,94 | 948,21 | 758,77 |
| 37 | Pro | 97,05 | a36 | 3 858,88 | 1 929,94 | 1 286,96 | b36 | 3 886,87 | 1 943,94 | 1 296,30 | 972,47 | 778,18 |
| 38 | Pro | 97,05 | a37 | 3 955,93 | 1 978,47 | 1 319,31 | b37 | 3 983,92 | 1 992,47 | 1 328,65 | 996,74 | 797,59 |
| 39 | Ser | 87,03 | a38 | 4 052,98 | 2 026,99 | 1 351,67 | b38 | 4 080,98 | 2 040,99 | 1 361,00 | 1 021,00 | 817,00 |
| 40 | NH2 | 17,03 | a39 | 4 140,01 | 2 070,51 | 1 380,68 | b39 | 4 168,01 | 2 084,51 | 1 390,01 | 1 042,76 | 834,41 |

| | | y | C-Terminal Ion Series | | | | | c | N-Terminal Ion Series | | | z | C-Terminal Ion Series | | |
|---|---|---|---|---|---|---|---|---|---|---|---|---|---|---|---|
| | Exenatide | Ion | $y^{(1+)}$ | $y^{(2+)}$ | $y^{(3+)}$ | $y^{(4+)}$ | $y^{(5+)}$ | Ion | $c^{(1+)}$ | $c^{(2+)}$ | $c^{(3+)}$ | Ion | $z^{(1+)}$ | $z^{(2+)}$ | $z^{(3+)}$ |
| 1 | His | | 4 185,03 | 2 093,02 | 1 395,68 | 1 047,01 | 837,81 | | | | | | 4 169,02 | 2 085,01 | 1 390,34 |
| 2 | Gly | y40 | 4 047,98 | 2 024,49 | 1 350,00 | 1 012,75 | 810,40 | c1 | 154,07 | 77,54 | 52,03 | z40 | 4 031,96 | 2 016,48 | 1 344,66 |
| 3 | Glu | y39 | 3 990,95 | 1 995,98 | 1 330,99 | 998,49 | 799,00 | c2 | 211,10 | 106,05 | 71,04 | z39 | 3 974,94 | 1 987,97 | 1 325,65 |
| 4 | Gly | y38 | 3 861,91 | 1 931,46 | 1 287,98 | 966,23 | 773,19 | c3 | 340,14 | 170,57 | 114,05 | z38 | 3 845,89 | 1 923,45 | 1 282,64 |
| 5 | Thr | y37 | 3 804,89 | 1 902,95 | 1 268,97 | 951,98 | 761,78 | c4 | 397,16 | 199,08 | 133,06 | z37 | 3 788,87 | 1 894,94 | 1 263,63 |
| 6 | Phe | y36 | 3 703,84 | 1 852,42 | 1 235,29 | 926,72 | 741,57 | c5 | 498,21 | 249,61 | 166,74 | z36 | 3 687,82 | 1 844,42 | 1 229,95 |
| 7 | Thr | y35 | 3 556,77 | 1 778,89 | 1 186,26 | 889,95 | 712,16 | c6 | 645,28 | 323,14 | 215,76 | z35 | 3 540,76 | 1 770,88 | 1 180,92 |
| 8 | Ser | y34 | 3 455,73 | 1 728,37 | 1 152,58 | 864,69 | 691,95 | c7 | 746,32 | 373,67 | 249,45 | z34 | 3 439,71 | 1 720,36 | 1 147,24 |
| 9 | Asp | y33 | 3 368,69 | 1 684,85 | 1 123,57 | 842,93 | 674,54 | c8 | 833,36 | 417,18 | 278,46 | z33 | 3 352,68 | 1 676,84 | 1 118,23 |
| 10 | Leu | y32 | 3 253,67 | 1 627,34 | 1 085,23 | 814,17 | 651,54 | c9 | 948,38 | 474,69 | 316,80 | z32 | 3 237,65 | 1 619,33 | 1 079,89 |
| 11 | Ser | y31 | 3 140,58 | 1 570,80 | 1 047,53 | 785,90 | 628,92 | c10 | 1 061,47 | 531,24 | 354,49 | z31 | 3 124,56 | 1 562,79 | 1 042,19 |
| 12 | Lys | y30 | 3 053,55 | 1 527,28 | 1 018,52 | 764,14 | 611,52 | c11 | 1 148,50 | 574,75 | 383,50 | z30 | 3 037,53 | 1 519,27 | 1 013,18 |
| 13 | Gln | y29 | 2 925,46 | 1 463,23 | 975,82 | 732,12 | 585,90 | c12 | 1 276,59 | 638,80 | 426,20 | z29 | 2 909,44 | 1 455,22 | 970,48 |
| 14 | Met | y28 | 2 797,40 | 1 399,20 | 933,14 | 700,10 | 560,29 | c13 | 1 404,65 | 702,83 | 468,89 | z28 | 2 781,38 | 1 391,19 | 927,80 |
| 15 | Glu | y27 | 2 666,36 | 1 333,68 | 889,46 | 667,34 | 534,08 | c14 | 1 535,69 | 768,35 | 512,57 | z27 | 2 650,34 | 1 325,67 | 884,12 |
| 16 | Glu | y26 | 2 537,31 | 1 269,16 | 846,44 | 635,08 | 508,27 | c15 | 1 664,74 | 832,87 | 555,58 | z26 | 2 521,30 | 1 261,15 | 841,10 |
| 17 | Glu | y25 | 2 408,27 | 1 204,64 | 803,43 | 602,82 | 482,46 | c16 | 1 793,78 | 897,39 | 598,60 | z25 | 2 392,25 | 1 196,63 | 798,09 |
| 18 | Ala | y24 | 2 279,23 | 1 140,12 | 760,41 | 570,56 | 456,65 | c17 | 1 922,82 | 961,91 | 641,61 | z24 | 2 263,21 | 1 132,11 | 755,08 |
| 19 | Val | y23 | 2 208,19 | 1 104,60 | 736,74 | 552,80 | 442,44 | c18 | 1 993,86 | 997,43 | 665,29 | z23 | 2 192,17 | 1 096,59 | 731,40 |
| 20 | Arg | y22 | 2 109,12 | 1 055,07 | 703,71 | 528,04 | 422,63 | c19 | 2 092,93 | 1 046,97 | 698,31 | z22 | 2 093,10 | 1 047,06 | 698,37 |
| 21 | Leu | y21 | 1 953,02 | 977,02 | 651,68 | 489,01 | 391,41 | c20 | 2 249,03 | 1 125,02 | 750,35 | z21 | 1 937,00 | 969,01 | 646,34 |
| 22 | Phe | y20 | 1 839,94 | 920,47 | 613,98 | 460,74 | 368,79 | c21 | 2 362,11 | 1 181,56 | 788,04 | z20 | 1 823,92 | 912,46 | 608,64 |
| 23 | Ile | y19 | 1 692,87 | 846,94 | 564,96 | 423,97 | 339,38 | c22 | 2 509,18 | 1 255,09 | 837,06 | z19 | 1 676,85 | 838,93 | 559,62 |
| 24 | Glu | y18 | 1 579,79 | 790,40 | 527,27 | 395,70 | 316,76 | c23 | 2 622,26 | 1 311,64 | 874,76 | z18 | 1 563,77 | 782,39 | 521,93 |
| 25 | Trp | y17 | 1 450,74 | 725,88 | 484,25 | 363,44 | 290,95 | c24 | 2 751,31 | 1 376,16 | 917,77 | z17 | 1 434,72 | 717,87 | 478,91 |
| 26 | Leu | y16 | 1 264,66 | 632,84 | 422,23 | 316,92 | 253,74 | c25 | 2 937,39 | 1 469,20 | 979,80 | z16 | 1 248,65 | 624,83 | 416,89 |
| 27 | Lys | y15 | 1 151,58 | 576,29 | 384,53 | 288,65 | 231,12 | c26 | 3 050,47 | 1 525,74 | 1 017,49 | z15 | 1 135,56 | 568,28 | 379,19 |
| 28 | Asn | y14 | 1 023,49 | 512,25 | 341,83 | 256,63 | 205,50 | c27 | 3 178,56 | 1 589,79 | 1 060,19 | z14 | 1 007,47 | 504,24 | 336,49 |
| 29 | Gly | y13 | 909,44 | 455,22 | 303,82 | 228,12 | 182,69 | c28 | 3 292,61 | 1 646,81 | 1 098,21 | z13 | 893,42 | 447,22 | 298,48 |
| 30 | Gly | y12 | 852,42 | 426,71 | 284,81 | 213,86 | 171,29 | c29 | 3 349,63 | 1 675,32 | 1 117,21 | z12 | 836,40 | 418,70 | 279,47 |
| 31 | Pro | y11 | 795,40 | 398,20 | 265,80 | 199,61 | 159,89 | c30 | 3 406,65 | 1 703,83 | 1 136,22 | z11 | 779,38 | 390,19 | 260,47 |
| 32 | Ser | y10 | 698,35 | 349,68 | 233,45 | 175,34 | 140,48 | c31 | 3 503,70 | 1 752,36 | 1 168,57 | z10 | 682,33 | 341,67 | 228,11 |
| 33 | Ser | y9 | 611,31 | 306,16 | 204,44 | 153,58 | 123,07 | c32 | 3 590,74 | 1 795,87 | 1 197,58 | z9 | 595,30 | 298,15 | 199,10 |
| 34 | Gly | y8 | 524,28 | 262,64 | 175,43 | 131,83 | 105,66 | c33 | 3 677,77 | 1 839,39 | 1 226,59 | z8 | 508,26 | 254,64 | 170,09 |
| 35 | Ala | y7 | 467,26 | 234,13 | 156,43 | 117,57 | 94,26 | c34 | 3 734,79 | 1 867,90 | 1 245,60 | z7 | 451,24 | 226,12 | 151,09 |
| 36 | Pro | y6 | 396,22 | 198,62 | 132,75 | 99,81 | 80,05 | c35 | 3 805,83 | 1 903,42 | 1 269,28 | z6 | 380,21 | 190,61 | 127,41 |
| 37 | Pro | y5 | 299,17 | 150,09 | 100,40 | 75,55 | 60,64 | c36 | 3 902,88 | 1 951,94 | 1 301,63 | z5 | 283,15 | 142,08 | 95,06 |
| 38 | Pro | y4 | 202,12 | 101,56 | 68,04 | 51,29 | 41,23 | c37 | 3 999,93 | 2 000,47 | 1 333,98 | z4 | 186,10 | 93,55 | 62,70 |
| 39 | Ser | y3 | 105,07 | 53,04 | 35,69 | 27,02 | 21,82 | c38 | 4 096,98 | 2 049,00 | 1 366,33 | z3 | 89,05 | 45,03 | 30,35 |
| 40 | NH2 | y2 | 18,03 | 9,52 | 6,68 | 5,26 | 4,41 | | | | | | | | |

**Table S30.** Overview of fragment ions for add on DCBM exenatide

| Most abundant | | | a | N-Terminal Ion Series | | | b | N-Terminal Ion Series | | | | | y | C-Terminal Ion Series | | | | | c | N-Terminal Ion Series | | | z | C-Terminal Ion Series | | |
|---|---|---|---|---|---|---|---|---|---|---|---|---|---|---|---|---|---|---|---|---|---|---|---|---|---|---|
| Exenatide | | | Ion | $a^{(1+)}$ | $a^{(2+)}$ | $a^{(3+)}$ | Ion | $b^{(1+)}$ | $b^{(2+)}$ | $b^{(3+)}$ | $b^{(4+)}$ | $b^{(5+)}$ | Ion | $y^{(1+)}$ | $y^{(2+)}$ | $y^{(3+)}$ | $y^{(4+)}$ | $y^{(5+)}$ | Ion | $c^{(1+)}$ | $c^{(2+)}$ | $c^{(3+)}$ | Ion | $z^{(1+)}$ | $z^{(2+)}$ | $z^{(3+)}$ |
| 1 | His | 137,06 | 0 | | | | 0 | | | | | | | 4 374,98 | 2 187,99 | 1 459,00 | 1 094,50 | 875,80 | | | | | | 4 358,96 | 2 179,98 | 1 453,66 |
| 2 | Gly | 57,02 | a1 | 110,07 | 55,54 | 37,36 | b1 | 138,07 | 69,54 | 46,69 | 35,27 | 28,42 | y40 | 4 237,92 | 2 119,46 | 1 413,31 | 1 060,23 | 848,39 | c1 | 154,07 | 77,54 | 52,03 | z40 | 4 221,90 | 2 111,45 | 1 407,97 |
| 3 | Glu | 129,04 | a2 | 167,09 | 84,05 | 56,37 | b2 | 195,09 | 98,05 | 65,70 | 49,53 | 39,82 | y39 | 4 180,90 | 2 090,95 | 1 394,30 | 1 045,98 | 836,98 | c2 | 211,10 | 106,05 | 71,04 | z39 | 4 164,88 | 2 082,94 | 1 388,96 |
| 4 | Gly | 57,02 | a3 | 296,14 | 148,57 | 99,38 | b3 | 324,13 | 162,57 | 108,71 | 81,79 | 65,63 | y38 | 4 051,85 | 2 026,43 | 1 351,29 | 1 013,72 | 811,18 | c3 | 340,14 | 170,57 | 114,05 | z38 | 4 035,83 | 2 018,42 | 1 345,95 |
| 5 | Thr | 101,05 | a4 | 353,16 | 177,08 | 118,39 | b4 | 381,15 | 191,08 | 127,72 | 96,04 | 77,04 | y37 | 3 994,83 | 1 997,92 | 1 332,28 | 999,46 | 799,77 | c4 | 397,16 | 199,08 | 133,06 | z37 | 3 978,81 | 1 989,91 | 1 326,94 |
| 6 | Phe | 147,07 | a5 | 454,20 | 227,61 | 152,07 | b5 | 482,20 | 241,60 | 161,40 | 121,31 | 97,25 | y36 | 3 893,78 | 1 947,40 | 1 298,60 | 974,20 | 779,56 | c5 | 498,21 | 249,61 | 166,74 | z36 | 3 877,76 | 1 939,39 | 1 293,26 |
| 7 | Thr | 101,05 | a6 | 601,27 | 301,14 | 201,10 | b6 | 629,27 | 315,14 | 210,43 | 158,07 | 126,66 | y35 | 3 746,72 | 1 873,86 | 1 249,58 | 937,43 | 750,15 | c6 | 645,28 | 323,14 | 215,76 | z35 | 3 730,70 | 1 865,85 | 1 244,24 |
| 8 | Ser | 87,03 | a7 | 702,32 | 351,66 | 234,78 | b7 | 730,32 | 365,66 | 244,11 | 183,33 | 146,87 | y34 | 3 645,67 | 1 823,34 | 1 215,89 | 912,17 | 729,94 | c7 | 746,32 | 373,67 | 249,45 | z34 | 3 629,65 | 1 815,33 | 1 210,55 |
| 9 | Asp | 115,03 | a8 | 789,35 | 395,18 | 263,79 | b8 | 817,35 | 409,18 | 273,12 | 205,09 | 164,28 | y33 | 3 558,64 | 1 779,82 | 1 186,88 | 890,41 | 712,53 | c8 | 833,36 | 417,18 | 278,46 | z33 | 3 542,62 | 1 771,81 | 1 181,54 |
| 10 | Leu | 113,08 | a9 | 904,38 | 452,69 | 302,13 | b9 | 932,37 | 466,69 | 311,46 | 233,85 | 187,28 | y32 | 3 443,61 | 1 722,31 | 1 148,54 | 861,66 | 689,53 | c9 | 948,38 | 474,69 | 316,80 | z32 | 3 427,59 | 1 714,30 | 1 143,20 |
| 11 | Ser | 87,03 | a10 | 1 017,46 | 509,24 | 339,83 | b10 | 1 045,46 | 523,23 | 349,16 | 262,12 | 209,90 | y31 | 3 330,52 | 1 665,77 | 1 110,85 | 833,39 | 666,91 | c10 | 1 061,47 | 531,24 | 354,49 | z31 | 3 314,51 | 1 657,76 | 1 105,51 |
| 12 | Lys | 128,09 | a11 | 1 104,50 | 552,75 | 368,84 | b11 | 1 132,49 | 566,75 | 378,17 | 283,88 | 227,30 | y30 | 3 243,49 | 1 622,25 | 1 081,84 | 811,63 | 649,50 | c11 | 1 148,50 | 574,75 | 383,50 | z30 | 3 227,47 | 1 614,24 | 1 076,50 |
| 13 | Gln | 128,06 | a12 | 1 232,59 | 616,80 | 411,54 | b12 | 1 260,59 | 630,80 | 420,87 | 315,90 | 252,92 | y29 | 3 115,40 | 1 558,20 | 1 039,14 | 779,60 | 623,89 | c12 | 1 276,59 | 638,80 | 426,20 | z29 | 3 099,38 | 1 550,19 | 1 033,80 |
| 14 | Met | 131,04 | a13 | 1 360,65 | 680,83 | 454,22 | b13 | 1 388,64 | 694,83 | 463,55 | 347,92 | 278,53 | y28 | 2 987,34 | 1 494,17 | 996,45 | 747,59 | 598,27 | c13 | 1 404,65 | 702,83 | 468,89 | z28 | 2 971,32 | 1 486,16 | 991,11 |
| 15 | Glu | 129,04 | a14 | 1 491,69 | 746,35 | 497,90 | b14 | 1 519,68 | 760,35 | 507,23 | 380,68 | 304,74 | y27 | 2 856,30 | 1 428,65 | 952,77 | 714,83 | 572,07 | c14 | 1 535,69 | 768,35 | 512,57 | z27 | 2 840,28 | 1 420,64 | 947,43 |
| 16 | Glu | 129,04 | a15 | 1 620,73 | 810,87 | 540,92 | b15 | 1 648,73 | 824,87 | 550,25 | 412,94 | 330,55 | y26 | 2 727,26 | 1 364,13 | 909,76 | 682,57 | 546,26 | c15 | 1 664,74 | 832,87 | 555,58 | z26 | 2 711,24 | 1 356,12 | 904,42 |
| 17 | Glu | 129,04 | a16 | 1 749,77 | 875,39 | 583,93 | b16 | 1 777,77 | 889,39 | 593,26 | 445,20 | 356,36 | y25 | 2 598,21 | 1 299,61 | 866,74 | 650,31 | 520,45 | c16 | 1 793,78 | 897,39 | 598,60 | z25 | 2 582,19 | 1 291,60 | 861,40 |
| 18 | Ala | 71,04 | a17 | 1 878,82 | 939,91 | 626,94 | b17 | 1 906,81 | 953,91 | 636,28 | 477,46 | 382,17 | y24 | 2 469,17 | 1 235,09 | 823,73 | 618,05 | 494,64 | c17 | 1 922,82 | 961,91 | 641,61 | z24 | 2 453,15 | 1 227,08 | 818,39 |
| 19 | Val | 99,07 | a18 | 1 949,85 | 975,43 | 650,62 | b18 | 1 977,85 | 989,43 | 659,95 | 495,22 | 396,38 | y23 | 2 398,13 | 1 199,57 | 800,05 | 600,29 | 480,43 | c18 | 1 993,86 | 997,43 | 665,29 | z23 | 2 382,11 | 1 191,56 | 794,71 |
| 20 | Arg | 156,10 | a19 | 2 048,92 | 1 024,97 | 683,65 | b19 | 2 076,92 | 1 038,96 | 692,98 | 519,98 | 416,19 | y22 | 2 299,07 | 1 150,04 | 767,03 | 575,52 | 460,62 | c19 | 2 092,93 | 1 046,97 | 698,31 | z22 | 2 283,05 | 1 142,03 | 761,69 |
| 21 | Leu | 113,08 | a20 | 2 205,02 | 1 103,02 | 735,68 | b20 | 2 233,02 | 1 117,01 | 745,01 | 559,01 | 447,41 | y21 | 2 142,96 | 1 071,99 | 714,99 | 536,50 | 429,40 | c20 | 2 249,03 | 1 125,02 | 750,35 | z21 | 2 126,94 | 1 063,98 | 709,65 |
| 22 | Phe | 147,07 | a21 | 2 318,11 | 1 159,56 | 773,37 | b21 | 2 346,10 | 1 173,56 | 782,71 | 587,28 | 470,03 | y20 | 2 029,88 | 1 015,44 | 677,30 | 508,23 | 406,78 | c21 | 2 362,11 | 1 181,56 | 788,04 | z20 | 2 013,86 | 1 007,43 | 671,96 |
| 23 | Ile | 113,08 | a22 | 2 465,18 | 1 233,09 | 822,40 | b22 | 2 493,17 | 1 247,09 | 831,73 | 624,05 | 499,44 | y19 | 1 882,81 | 941,91 | 628,28 | 471,46 | 377,37 | c22 | 2 509,18 | 1 255,09 | 837,06 | z19 | 1 866,79 | 933,90 | 622,94 |
| 24 | Glu | 129,04 | a23 | 2 578,26 | 1 289,63 | 860,09 | b23 | 2 606,26 | 1 303,63 | 869,42 | 652,32 | 522,06 | y18 | 1 769,73 | 885,37 | 590,58 | 443,19 | 354,75 | c23 | 2 622,26 | 1 311,64 | 874,76 | z18 | 1 753,71 | 877,36 | 585,24 |
| 25 | Trp(DCBM) | 376,02 | a24 | 2 707,30 | 1 354,16 | 903,11 | b24 | 2 735,30 | 1 368,15 | 912,44 | 684,58 | 547,87 | y17 | 1 640,68 | 820,85 | 547,57 | 410,93 | 328,94 | c24 | 2 751,31 | 1 376,16 | 917,77 | z17 | 1 624,67 | 812,84 | 542,23 |
| 26 | Leu | 113,08 | a25 | 3 083,32 | 1 542,17 | 1 028,45 | b25 | 3 111,32 | 1 556,16 | 1 037,78 | 778,59 | 623,07 | y16 | 1 264,66 | 632,84 | 422,23 | 316,92 | 253,74 | c25 | 3 127,33 | 1 564,17 | 1 043,11 | z16 | 1 248,65 | 624,83 | 416,89 |
| 27 | Lys | 128,09 | a26 | 3 196,41 | 1 598,71 | 1 066,14 | b26 | 3 224,40 | 1 612,70 | 1 075,47 | 806,86 | 645,69 | y15 | 1 151,58 | 576,29 | 384,53 | 288,65 | 231,12 | c26 | 3 240,41 | 1 620,71 | 1 080,81 | z15 | 1 135,56 | 568,28 | 379,19 |
| 28 | Asn | 114,04 | a27 | 3 324,50 | 1 662,75 | 1 108,84 | b27 | 3 352,50 | 1 676,75 | 1 118,17 | 838,88 | 671,31 | y14 | 1 023,49 | 512,25 | 341,83 | 256,63 | 205,50 | c27 | 3 368,51 | 1 684,76 | 1 123,51 | z14 | 1 007,47 | 504,24 | 336,49 |
| 29 | Gly | 57,02 | a28 | 3 438,55 | 1 719,78 | 1 146,85 | b28 | 3 466,54 | 1 733,77 | 1 156,19 | 867,39 | 694,11 | y13 | 909,44 | 455,22 | 303,82 | 228,12 | 182,69 | c28 | 3 482,55 | 1 741,78 | 1 161,52 | z13 | 893,42 | 447,22 | 298,48 |
| 30 | Gly | 57,02 | a29 | 3 495,57 | 1 748,29 | 1 165,86 | b29 | 3 523,56 | 1 762,28 | 1 175,19 | 881,65 | 705,52 | y12 | 852,42 | 426,71 | 284,81 | 213,86 | 171,29 | c29 | 3 539,57 | 1 770,29 | 1 180,53 | z12 | 836,40 | 418,70 | 279,47 |
| 31 | Pro | 97,05 | a30 | 3 552,59 | 1 776,80 | 1 184,87 | b30 | 3 580,58 | 1 790,80 | 1 194,20 | 895,90 | 716,92 | y11 | 795,40 | 398,20 | 265,80 | 199,61 | 159,89 | c30 | 3 596,59 | 1 798,80 | 1 199,54 | z11 | 779,38 | 390,19 | 260,47 |
| 32 | Ser | 87,03 | a31 | 3 649,64 | 1 825,32 | 1 217,22 | b31 | 3 677,64 | 1 839,32 | 1 226,55 | 920,16 | 736,33 | y10 | 698,35 | 349,68 | 233,45 | 175,34 | 140,48 | c31 | 3 693,64 | 1 847,33 | 1 231,89 | z10 | 682,33 | 341,67 | 228,11 |
| 33 | Ser | 87,03 | a32 | 3 736,67 | 1 868,84 | 1 246,23 | b32 | 3 764,67 | 1 882,84 | 1 255,56 | 941,92 | 753,74 | y9 | 611,31 | 306,16 | 204,44 | 153,58 | 123,07 | c32 | 3 780,68 | 1 890,84 | 1 260,90 | z9 | 595,30 | 298,15 | 199,10 |
| 34 | Gly | 57,02 | a33 | 3 823,71 | 1 912,36 | 1 275,24 | b33 | 3 851,70 | 1 926,35 | 1 284,57 | 963,68 | 771,15 | y8 | 524,28 | 262,64 | 175,43 | 131,83 | 105,66 | c33 | 3 867,71 | 1 934,36 | 1 289,91 | z8 | 508,26 | 254,64 | 170,09 |
| 35 | Ala | 71,04 | a34 | 3 880,73 | 1 940,87 | 1 294,25 | b34 | 3 908,72 | 1 954,86 | 1 303,58 | 977,94 | 782,55 | y7 | 467,26 | 234,13 | 156,43 | 117,57 | 94,26 | c34 | 3 924,73 | 1 962,87 | 1 308,91 | z7 | 451,24 | 226,12 | 151,09 |
| 36 | Pro | 97,05 | a35 | 3 951,76 | 1 976,39 | 1 317,93 | b35 | 3 979,76 | 1 990,38 | 1 327,26 | 995,70 | 796,76 | y6 | 396,22 | 198,62 | 132,75 | 99,81 | 80,05 | c35 | 3 995,77 | 1 998,39 | 1 332,59 | z6 | 380,21 | 190,61 | 127,41 |
| 37 | Pro | 97,05 | a36 | 4 048,82 | 2 024,91 | 1 350,28 | b36 | 4 076,81 | 2 038,91 | 1 359,61 | 1 019,96 | 816,17 | y5 | 299,17 | 150,09 | 100,40 | 75,55 | 60,64 | c36 | 4 092,82 | 2 046,91 | 1 364,94 | z5 | 283,15 | 142,08 | 95,06 |
| 38 | Pro | 97,05 | a37 | 4 145,87 | 2 073,44 | 1 382,63 | b37 | 4 173,86 | 2 087,44 | 1 391,96 | 1 044,22 | 835,58 | y4 | 202,12 | 101,56 | 68,04 | 51,29 | 41,23 | c37 | 4 189,87 | 2 095,44 | 1 397,30 | z4 | 186,10 | 93,55 | 62,70 |
| 39 | Ser | 87,03 | a38 | 4 242,92 | 2 121,96 | 1 414,98 | b38 | 4 270,92 | 2 135,96 | 1 424,31 | 1 068,48 | 854,99 | y3 | 105,07 | 53,04 | 35,69 | 27,02 | 21,82 | c38 | 4 286,92 | 2 143,97 | 1 429,65 | z3 | 89,05 | 45,03 | 30,35 |
| 40 | NH2 | 17,03 | a39 | 4 329,95 | 2 165,48 | 1 443,99 | b39 | 4 357,95 | 2 179,48 | 1 453,32 | 1 090,24 | 872,40 | y2 | 18,03 | 9,52 | 6,68 | 5,26 | 4,41 | | | | | | | | |

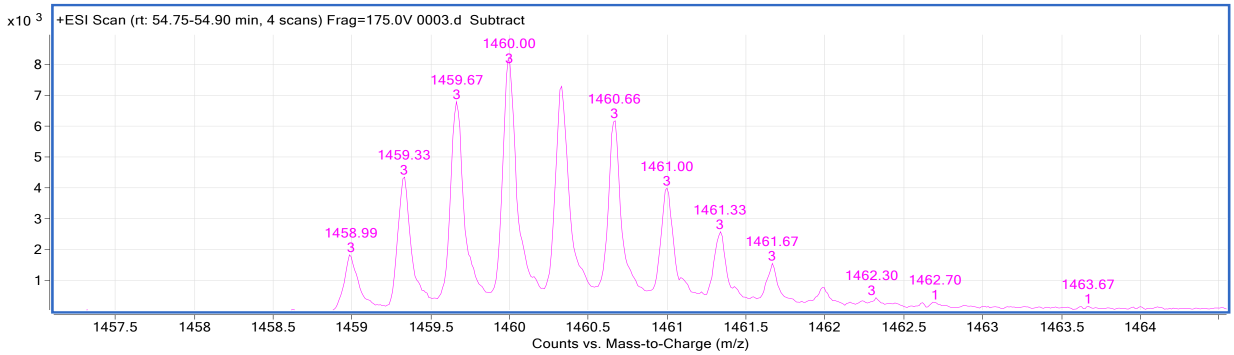


**Figure S27.** MS spectrum for the add on DCBM impurity

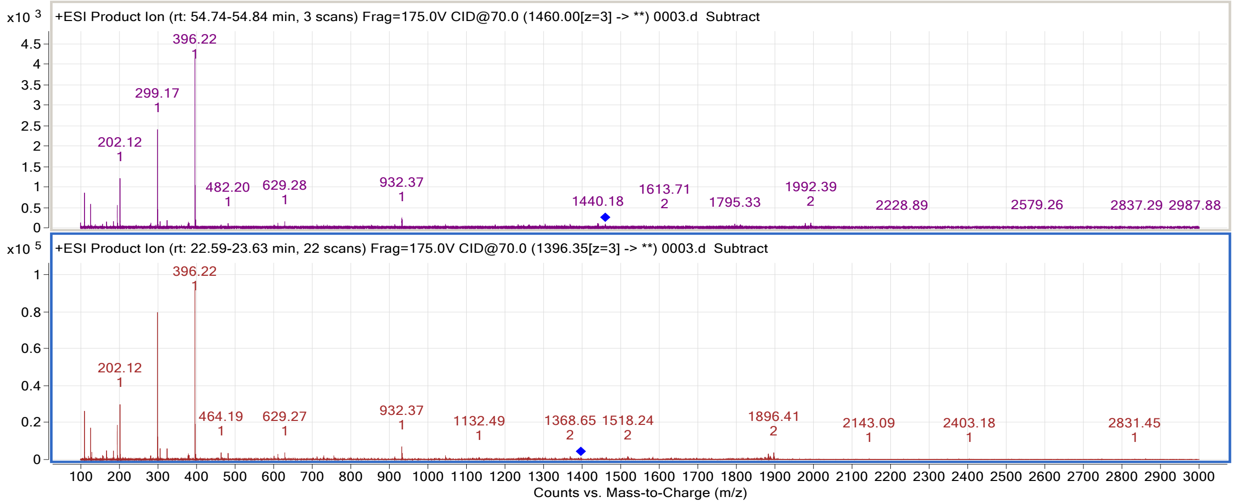


**Figure S28.** MS-MS spectra overview (exenatide and add on DCBM). Evaluating the fragments the same b5-b12, b21-b24, y4-6 and also some a-fragments a1-a2 are present in both main peak and add on DCBM. This points towards the hypothesis that DCBM is situated on Trp$^{25}$. In the main peak the b25 and b26 fragments 1461.19 and 1517.73 are present but not in add on DCBM. See Figure S29 and S30. In add on DCMB the b26 fragment 1612.75 is found but not in the main peak. See Figure S31.This confirms the position of add on DCBM on Trp$^{25}$.

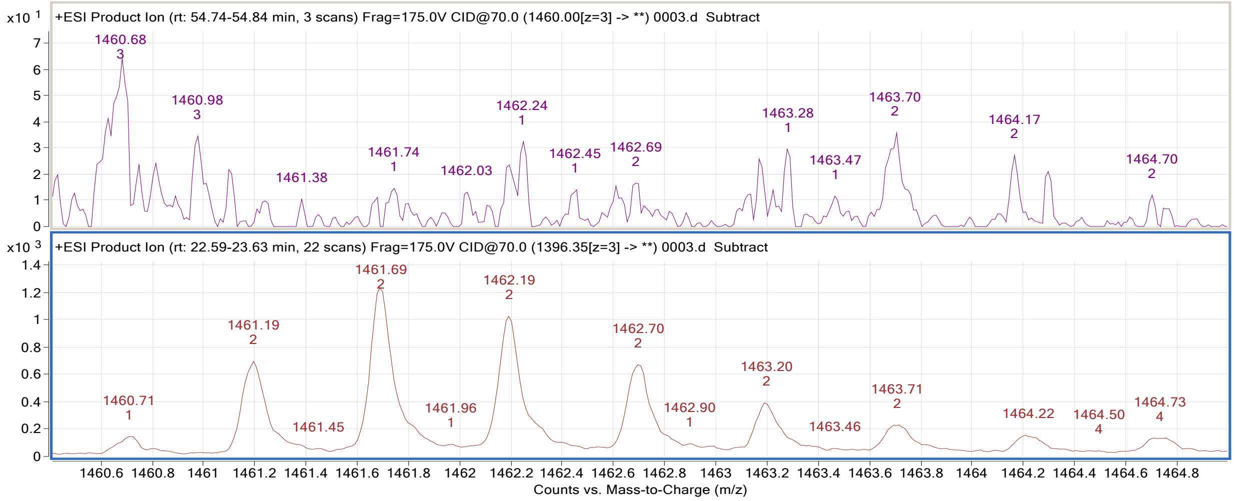


**Figure S29.** Fragment 1461.2. Overlay upper "add on DCBM, lower main peak.

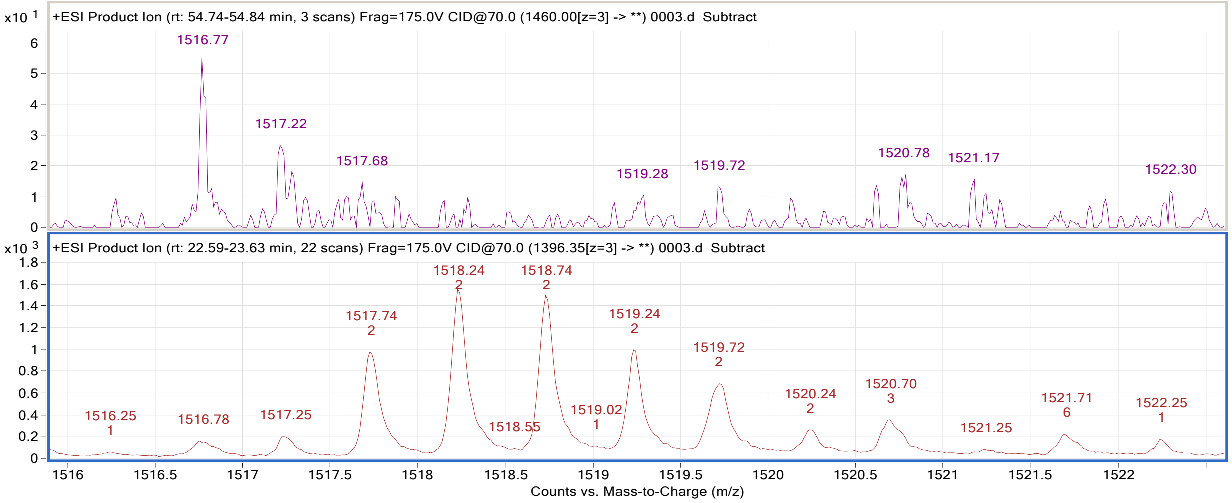


**Figure S30.** Fragment 1517.73. Overlay upper add on DCBM, lower main peak.

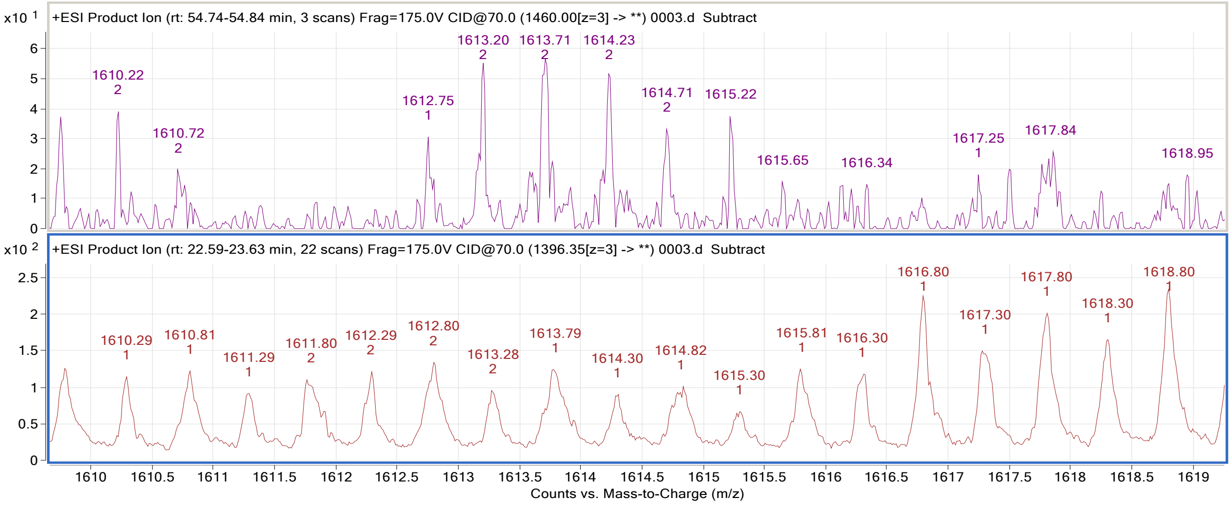


**Figure S31.** Fragment 1612.75. Overlay upper add on DCBM, lower main peak.

## 8. Assessment of thiol stability in TFA/TIS/$H_2O$

Following HPLC method was used for all analyses: column: Waters XSelect CSH130 C18 2.5µm 4.6x150mm; column temperature: 45°C; injection volume: 10 µL; sampler temperature:10°C; flow: 1.0 ml/min; mobile phase A: 0.1 % TFA in water, mobile phase B: 0.08 % TFA in 90% MeCN/10 %water. Gradient (Time(min), %B): 0, 1; 10, 100; 13, 100; 14, 100; 19, 1; 20, 1. The results for stability of thiols detailed in sections 8.1. – 8.4 are summarized in Fig. 5 of this paper.

### 8.1. Assessment of DTT stability in TFA/TIS/$H_2O$

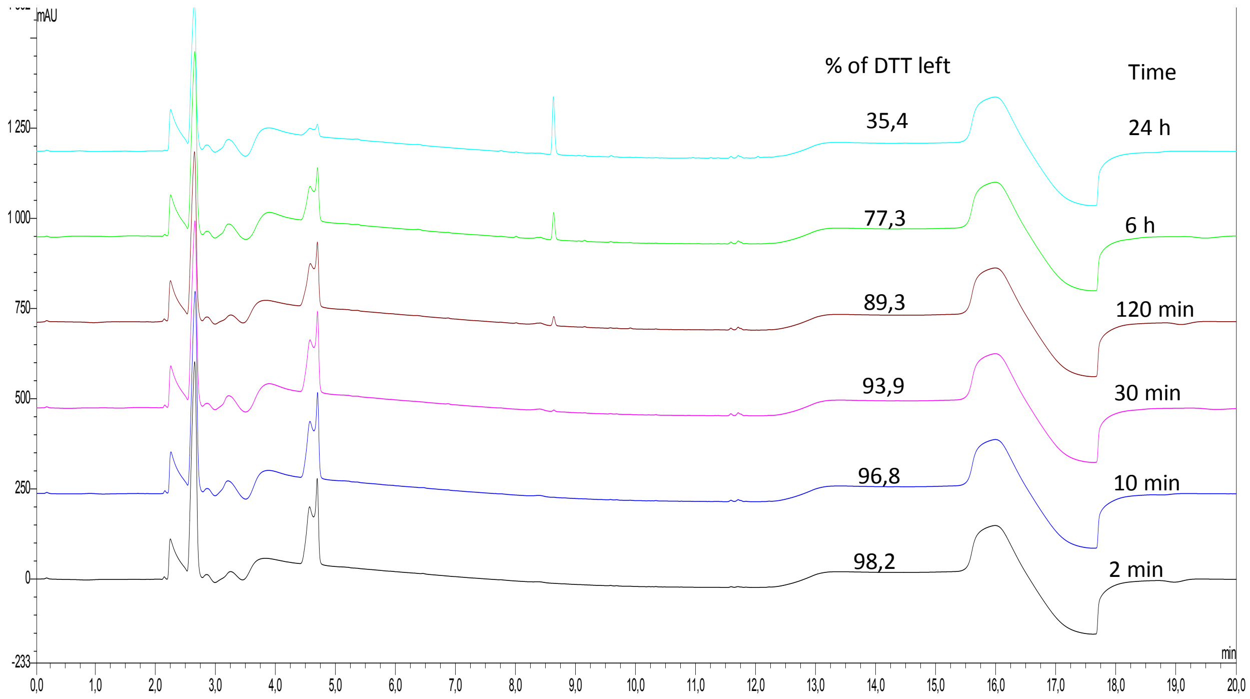


**Figure S32**. HPLC overlay for analyses of DTT stability in TFA/TIS/$H_2O$.

## 8.2. Assessment of DODT stability in TFA/TIS/$H_2O$

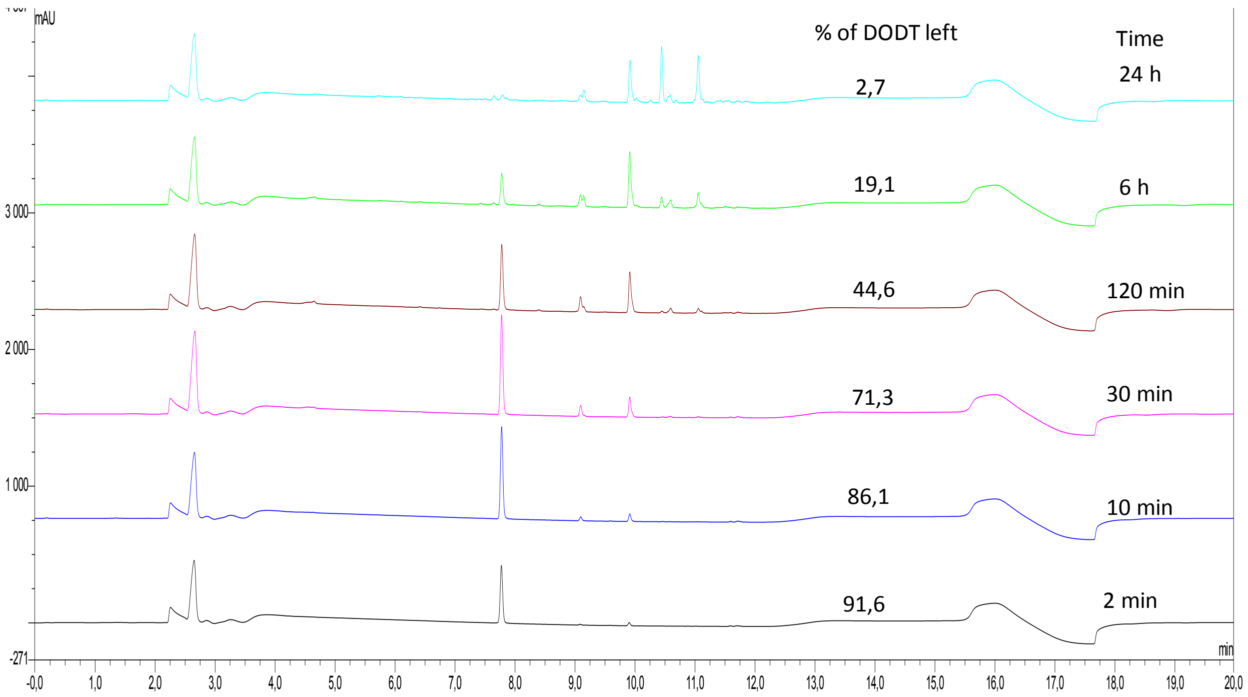


**Figure S33**. HPLC overlay for analyses of DODT stability in TFA/TIS/$H_2O$.

## 8.3. Assessment of 1,2-BDMT stability in TFA/TIS/$H_2O$

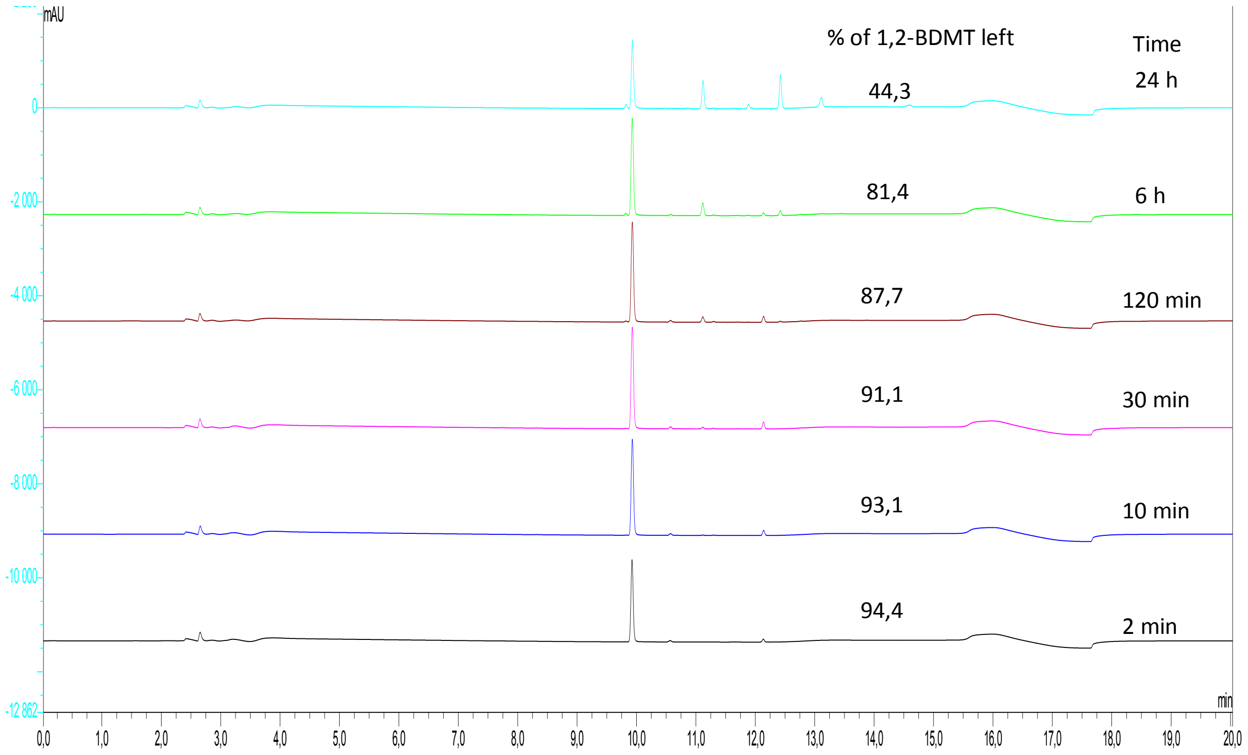


**Figure S34**. HPLC overlay for analyses of 1,2-BDMT stability in TFA/TIS/$H_2O$.

## 8.4. Assessment of 1,4-BDMT stability in TFA/TIS/$H_2O$

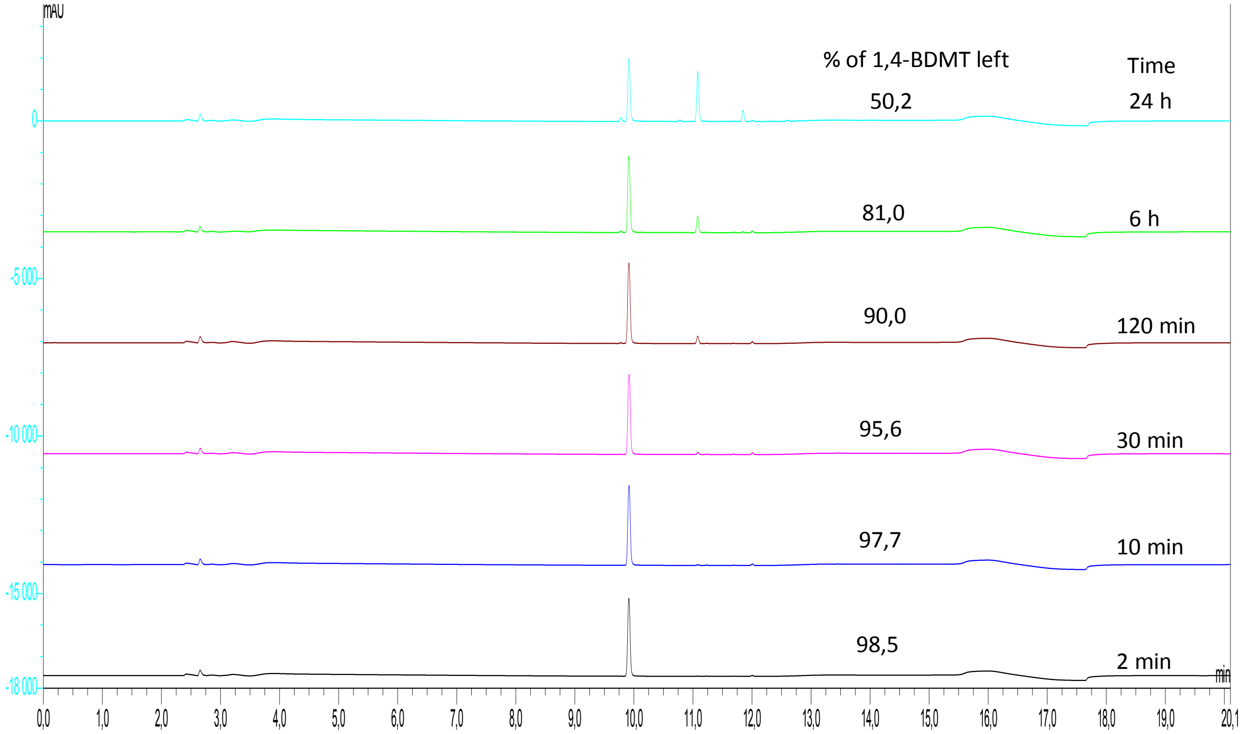


**Figure S35**. HPLC overlay for analyses of 1,4-BDMT stability in TFA/TIS/$H_2O$.

## 9. Assessment of precipitation during TFA cleavages using BTs as scavengers

TFA cleavages of exenatide peptide resin were carried out according to the protocol described in section 3 of this Electronic Supporting Information using 1,2-BDMT, 13-BDMT and 1,4-BDMT as scavengers. After 2 h of cleavage time the supernate containing the crude peptide was filtered off, leaving the spent resin and the precipitate formed during the cleavage. This residual material was rinsed with TFA (2 mL) after which the precipitate was dissolved in 5 mL dichloromethane (DCM) and the spent resin was filtered off. The volatiles were removed *en vacuo* affording the precipitates from the TFA cleavages as off-white solids. Following amounts of the precipitates were isolated: i) 1,2-BDMT as scavenger: 8 mg; ii) 1,3-BDMT as scavenger: 8 mg, iii) 1,4-BDMT as scavenger: 12 mg. The precipitates thus obtained were analyzed by HPLC (Fig. S36) using the analytical system employed in Section 8 of this Supporting Information. These analyses showed that the precipates observed were mostly formed during the TFA cleavages and the benzylthiol scavengers per se. Furthermore, LC-HRMS analysis of a precipitate from TFA cleavage of exenatide peptide resin using 1,4-BDMT was carried out (Figures S37 – S38). Based on this LC-HRMS assessment we propose that the precipitated material is based on formation of adducts of 1,4-BDMT with protectecting groups (PGs) such as the *t*-Bu functions. The formation of such scavenger-PG adducts is in keeping with the previously reported[9] formation of Di-*t*-Bu-DTT adducts formed during TFA cleavages of peptide resins using DTT as the scavenger.

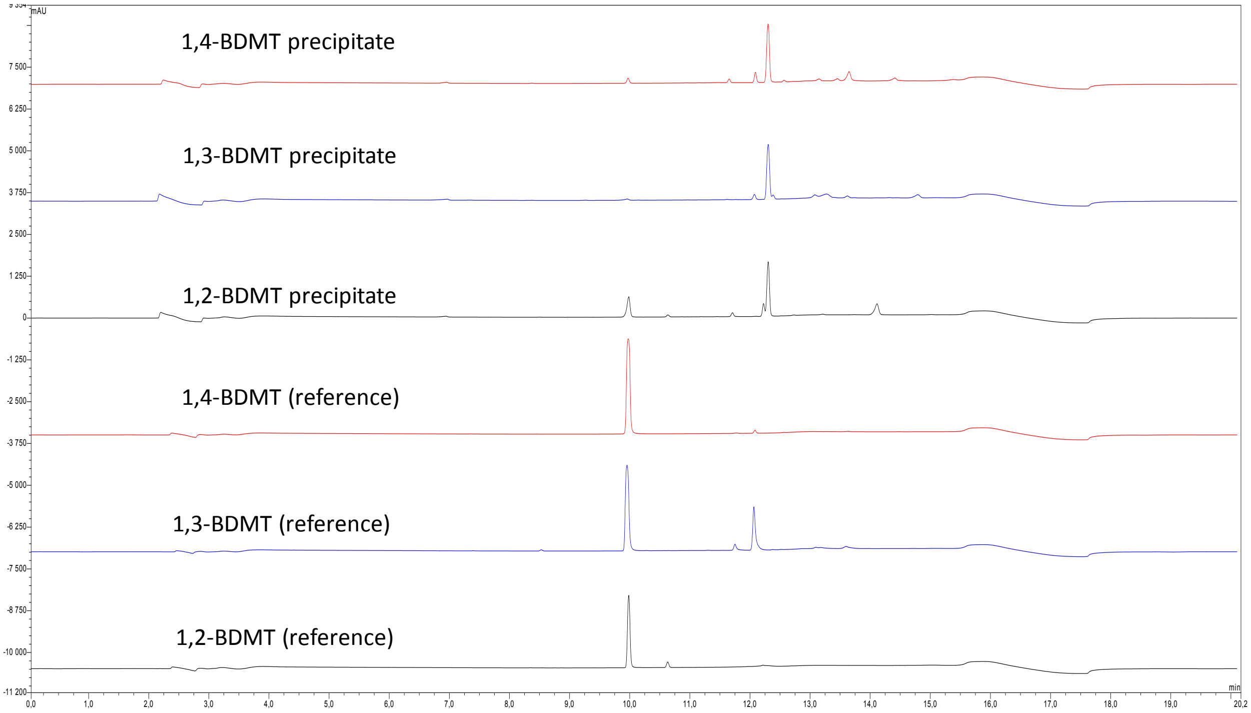


**Figure S36**. HPLC overlay for analyses of 1,2-, 1,3- and 1,4-BDMT scavengers vs precipitates formed during TFA cleavages of exenatide peptide resin using 1,2-, 1,3- and 1,4-BDMT as scavengers.

LC-HRMS assessment of the precipitate formed during the TFA cleavage of exenatide peptide resin using 1,4-BDMT as a scavenger. Experimental conditions: column: Waters peptide CSH C18, 2.1x150mm, 1.7um, 130Å; column temperature: 55°C; injection volume: 4 µL; sampler temperature: 10°C; MS mode: positive 50-3200; DAD: 220 nm; data rate: 5Hz; detector cell: standard cell 1uL; flow: 0.2 ml/min; jet weaver: v380 mixer; mobile phase A: 0.1 % TFA in water, mobile phase B: 0.10 % TFA in MeCN. Gradient (Time(min), %B): 0, 11; 1, 11; 30, 90; 32, 90; 32.1, 11; 45, 11.

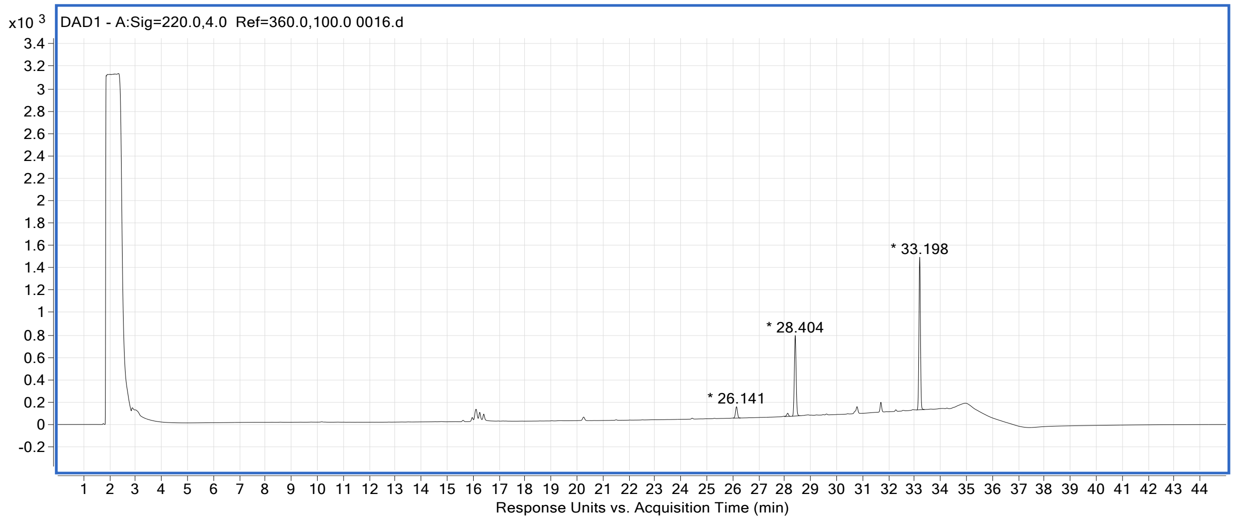


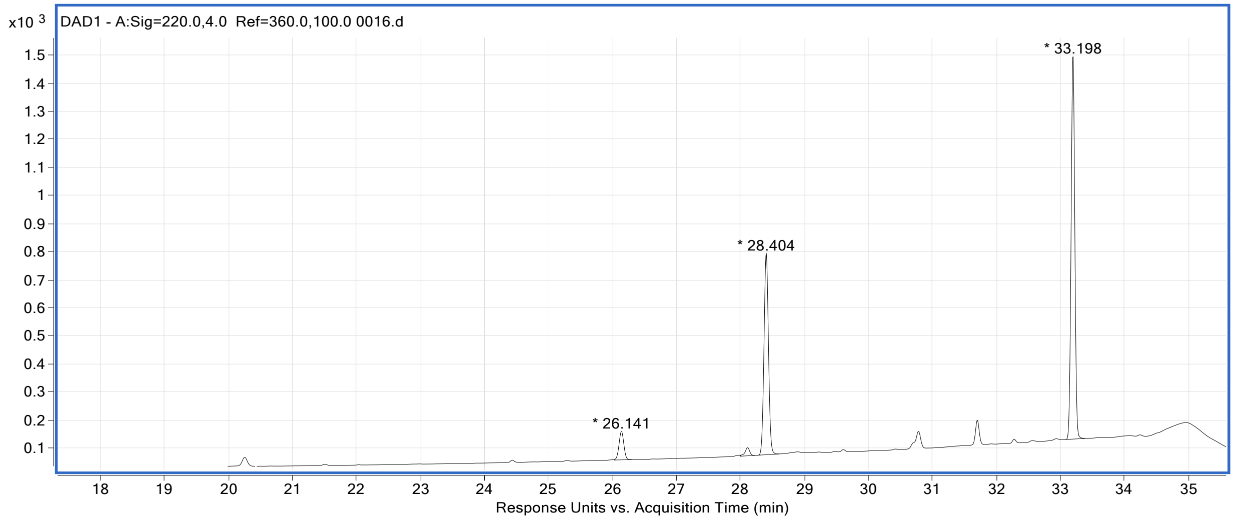


**Figure S37**. LC-HRMS analysis of precipitate formed during the TFA cleavage of exenatide peptide resin using 1,4-BDMT as a scavenger. Rt of = 20.3 min. Rt of the two main components of the precipitate: 28.4 min and 33.2 min.

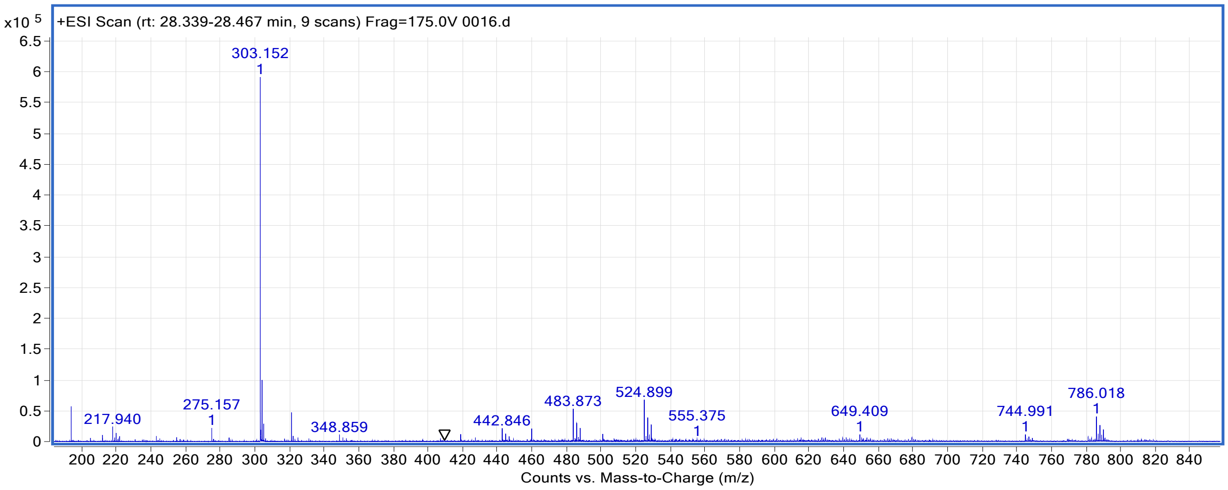


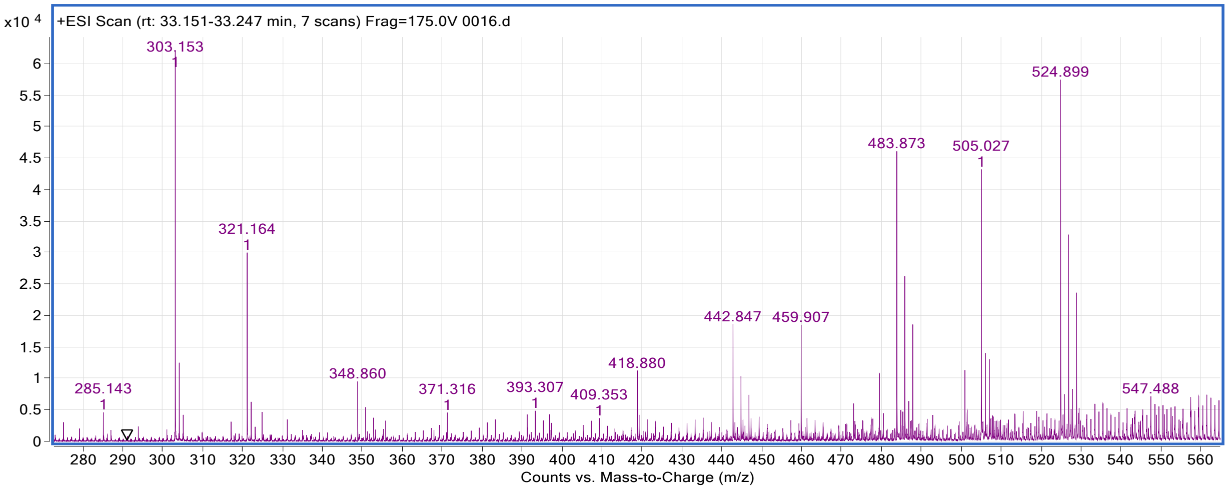


**Figure S38**. MS spectra of two main components (Rt 28.4 min and 33.2 min) of precipitate formed during TFA cleavage of exenatide peptide resin using 1,4-BDMT as a scavenger. The tentative assigned based on these MS spectra is that these species are conceivably based on the formation of adducts between 1,4-BDMT and protecting groups employed in the SPPS. For example, 1,4-BDMT-di-*t* two *t*-Bu groups, m/z 282.15; m/z 303.12 (+Na-2H), m/z 321.11 (+K).

## 10. Assessment of UV visibility (220 nm) of DTT and 1,4-BDMT

Samples of DTT and 1,4-BDMT in MeCN were analyzed using the following HPLC method: column: Phenomenex Kinetex C18 100A 2.6µm 4.6x50mm; column temperature: 45°C; injection volume: 10 µL; sampler temperature:10°C; flow: 1.0 ml/min; mobile phase A: 0.1 % TFA in water, mobile phase B: 0.08 % TFA in 90% MeCN/10 %water. Gradient (Time(min), %B): 0, 1; 10, 100; 13, 100; 14, 100; 19, 1; 20, 1.

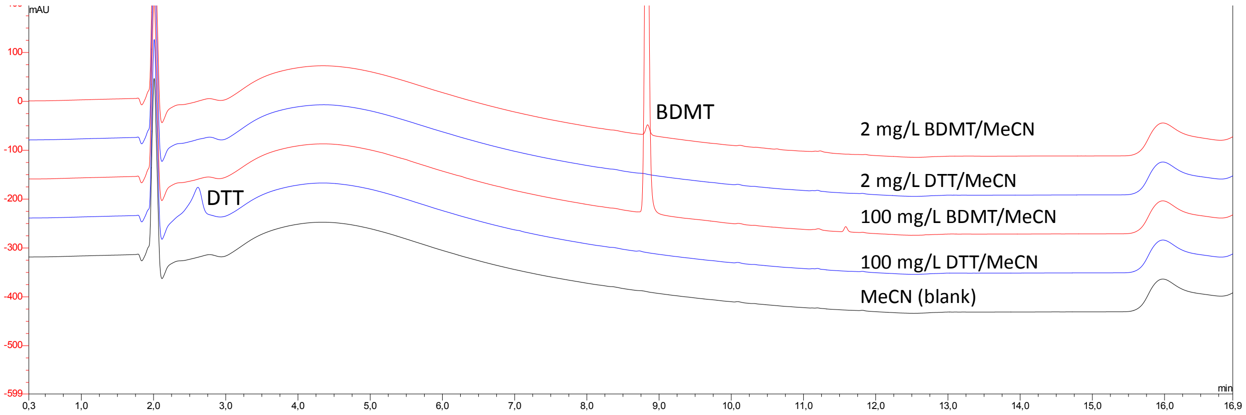


**Figure S39**. HPLC overlay of DTT and 1,4-BDMT at 100 mg/L and 2 mg/L respectively. At the lower concentration the aliphatic DTT is not detectable while the benzylthiol 1,4-BDMT is still easily detected.

---

[1] Aminomethyl (AM) functionalized copolymer of styrene and di(ethylene glycol) dimethacrylate (DEGDMA), see http://www.tjhecheng.com/index.php?m=content&c=index&a=show&catid=8&id=69.

[2] J. C. Sheehan, G. P. Hess, *J. Am. Chem. Soc.* 1955, **77**, 1067.

[3] N. L. Benoiton, F. M. F. Chen, *J. Chem. Soc. Chem. Commun.* 1981, 543.

[4] R. Ramage, S. L. Irving, C. McInnes, *Tetrahedron Lett*. 1993, **34**, 6599.

[5] E. Kaiser, R. L. Colescott, C. D. Bossinger, P. I. Cook, *Anal.Biochem.* 1970, **34**, 595.

[6] T. Vojkovsky, *Pept. Res*. 1995, **8**, 236.

[7] H. Franzén, L. Grehn, U. Ragnarsson, *J. Chem. Soc. Chem. Commun.* 1984, 1699.

[8] T. Todorovski, M. Fedorova, L. Hennig, R. Hoffmann, *J. Pept. Sci.* 2011, **17**, 256.

[9] For a report on a DTT-di-*t*-Bu adduct formed in peptide resin TFA cleavages see J. Pawlas, S. Hansen, T. Svensson, G. Stærkær, Poster presentation at EuroTIDES, November 2012, Berlin, Germany, https://www.polypeptide.com/web/upload/medias/15627436995d259393ac186.pdf.
